# Regioselective C3–H Alkylation of Imidazopyridines with Donor–Acceptor Cyclopropanes

**DOI:** 10.1021/acs.joc.3c01122

**Published:** 2023-08-03

**Authors:** Oguzhan Dalkilic, Ozge Turbedaroglu, Ferruh Lafzi, Haydar Kilic

**Affiliations:** Department of Chemistry, Faculty of Sciences, Atatürk University, Erzurum 25240, Türkiye

## Abstract

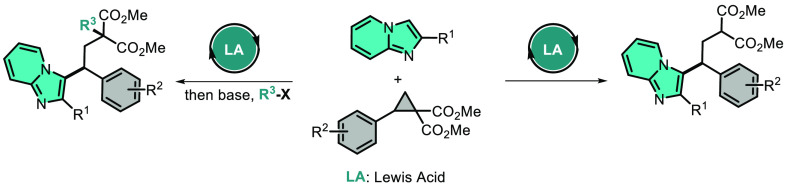

Alkylated imidazopyridines are crucial structures for medicinal chemistry. Here, an efficient method for the C3–H alkylation of imidazopyridines was devised. Under Lewis acid-catalyzed conditions, reactions of imidazopyridines with different donor–acceptor cyclopropanes were carried out. Finally, 1,3-bisfunctionalizaton of donor–acceptor cyclopropanes was performed. In addition, synthesized C3-alkylated imidazopyridines are amenable for diverse synthetic applications.

## Introduction

**Figure 1:**
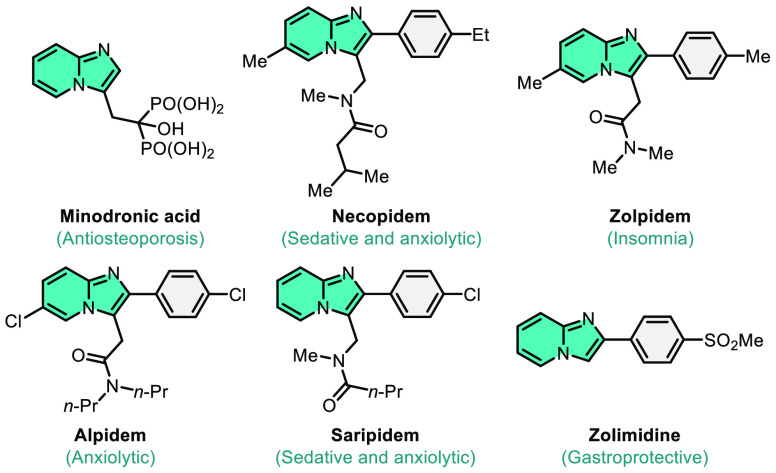
Drug molecules contain the imidazo[1,2-*a*]pyridine framework.

Imidazo[1,2-*a*]pyridine, which is found in numerous natural products, is regarded as the most significant derivative in the imidazopyridine family due to its many noteworthy biological activities.^1−5^ These compounds display a wide range of intriguing properties, including anticancer, antifungal, analgesic, antibacterial, antipyretic, anti-infective, anti-inflammatory, antiprotozoal, as well as pain-relieving and sedative effects.^1,2,6−9^ Zolpidem (insomnia drug),^10^ alpidem (anxiolytic drug),^11^ minodronic acid (antiosteoporosis drug),^12^ saripidem (sedative and anxiolytic drug),^13^ necopidem (sedative and anxiolytic drug),^14^ and zolimidine (gastroprotective drug)^15^ are some of the marketed drugs containing imidazo[1,2-*a*]pyridine core (Figure 1). Also, this functional group has demonstrated significant importance in material chemistry as it is capable of exhibiting excited state intramolecular proton transfer.^16^ The electron-rich C3 position of imidazo[1,2-*a*]pyridine has been the focus of numerous studies aimed at developing fascinating methods for functionalizing this position, including the formation of carbon–sulfur bonds, carbon–phosphorus bonds, carbon–carbon bonds, and carbon–halogen bonds.^17−30^ The significant attention given in the last few decades to the functionalization of imidazopyridines at various positions by different groups is attributed to these reasons.^31^

Donor–acceptor (DA) cyclopropanes are versatile building blocks used in chemical synthesis. They are a class of organic compounds that contain a cyclopropane ring flanked by two groups that are both electron-donating and electron-withdrawing. The electron-donating and electron-withdrawing groups create a “push-pull” effect that polarizes the cyclopropane ring, making it more reactive than a nonactivated cyclopropane. DA-cyclopropanes have been successfully employed to create a variety of alkylation products. These three-membered basic structures have been used to synthesize a number of important compounds. The strain energy in cyclopropane is stated to be 115 kJ/mol and this energy drives ring-opening and cycloaddition processes.^32^ The push-pull mechanism of DA-cyclopropanes, which are usually referred to as 1,3-dipolar zwitterionic synthons, makes them one of the most sought after building blocks for a number of reactions, including ring-opening events, cycloadditions, and rearrangement procedures.^33−50^

**Scheme 1:**
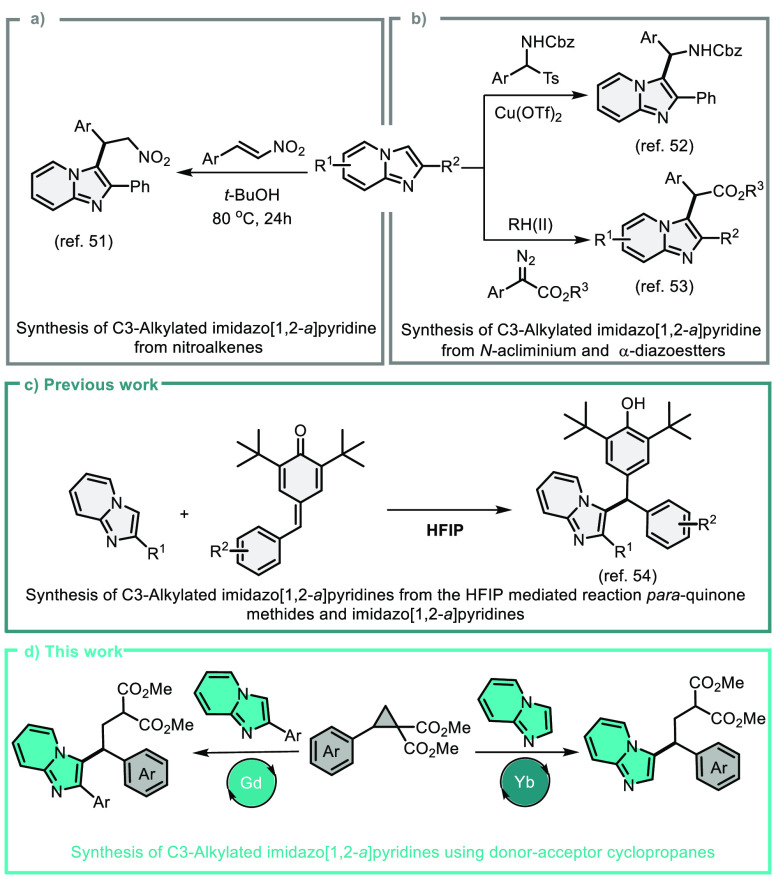
Development of C3–H Alkylation of Imidazo[1,2-*a*]pyridines and Overview of This Work

Because of the relevance and use of imidazopyridines, substantial advancement in their direct C3–H alkylation has been accomplished in recent years. Despite these advances, selective alkylation of imidazo[1,2-*a*]pyridines with donor–acceptor cyclopropanes has not been reported. (Scheme 1a–c).^51−54^ Herein, we report the nucleophilic ring-opening reaction of DA-cyclopropanes with imidazo[1,2-*a*]pyridines (Scheme 1d). We also investigated three component 1,3-bisfunctionalizations of DA-cyclopropanes.

## Results and Discussion

We commenced the study with imidazo[1,2-*a*]pyridine (**1**) and dimethyl 2-(4-methoxyphenyl)cyclopropane-1,1-dicarboxylate (**2a**) as the model substrate (Table 1). Initially, the reaction was performed with Zn(OTf)_2_ in the presence of 1,2-dichloroethane (DCE) as a solvent; unfortunately, the reaction did not yield the product under these conditions (Table 1, entry 1). Moreover, other triflate catalysts, Sc(OTf)_3_, Cu(OTf)_2_, Bi(OTf)_3_, LiOTf, AgOTf, Sn(OTf)_2_, and Gd(OTf)_3_, were ineffective in catalyzing the reaction and no product was formed (Table 1, entries 2–8). Then, Zn(TFA)_2_ and BF_3_^.^Et_2_O were tested as catalysts in DCE and at room temperature (rt), and the ring-opening product could not obtained using those catalysts (Table 1, entries 9 and 10). Next, we examined Yb(OTf)_3_ as a catalyst in DCE. When 10% of Yb(OTf)_3_ was utilized in DCE at rt, eventually the reaction afforded ring-opening product **3a** in a yield of 30% (Table 1, entry 11). Following this, the solvent was replaced by CH_3_CN and the yield of **3a** slightly increased to 45% (Table 1, entry 12). Increasing the catalyst ratio to 20% improved the product yield up to 53% (Table 1, entry 13). At the same time, altering the temperature to 100 °C resulted in a satisfactory increase in efficiency up to 89% yield (Table 1, entry 14). Finally, increasing the amount of the catalyst to 25 mol % gave **3a** in 96% yield (Table 1, entry 15). Thus, optimized reaction conditions were found: 1.0 mmol **1**, 1.0 mmol **2a**, 25 mol % Yb(OTf)_3_, 3 mL CH_3_CN, and 100 °C for 24 h.

**Table 1:**
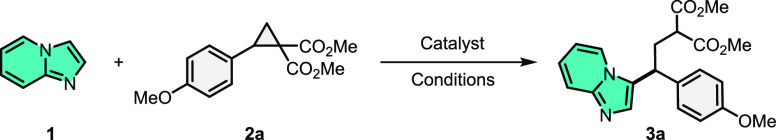
Optimization of Reaction Conditions^a^^,^^b^

entry	catalyst	mol (%)	solvent	time (h)	yield (%)^b^
1	Zn(OTf)_2_	10	DCE	12	ND
2	Sc(OTf)_3_	10	DCE	12	ND
3	Cu(OTf)_2_	10	DCE	12	ND
4	Bi(OTf)_3_	10	DCE	12	ND
5	LiOTf	10	DCE	12	ND
6	AgOTf	10	DCE	12	ND
7	Sn(OTf)_2_	10	DCE	12	ND
8	Gd(OTf)_3_	10	DCE	12	ND
9	Zn(TFA)_2_	10	DCE	12	ND
10	BF_3_^.^Et_2_O	10	DCE	12	ND
11	Yb(OTf)_3_	10	DCE	12	30
12	Yb(OTf)_3_	10	CH_3_CN	12	45
13	Yb(OTf)_3_	20	CH_3_CN	24	53
14^c^	Yb(OTf)_3_	20	CH_3_CN	24	89
15^c^	Yb(OTf)_3_	25	CH_3_CN	24	96

a General conditions: Imidazo[1,2-*a*]pyridine (1.0 mmol), donor–acceptor cyclopropane (1.0 mmol), catalyst (25 mol %), solvent (3.0 mL), rt.

b Isolated yields.

c Reactions were carried out at 100 °C (in an oil bath). ND = not determined.

**Scheme 2:**
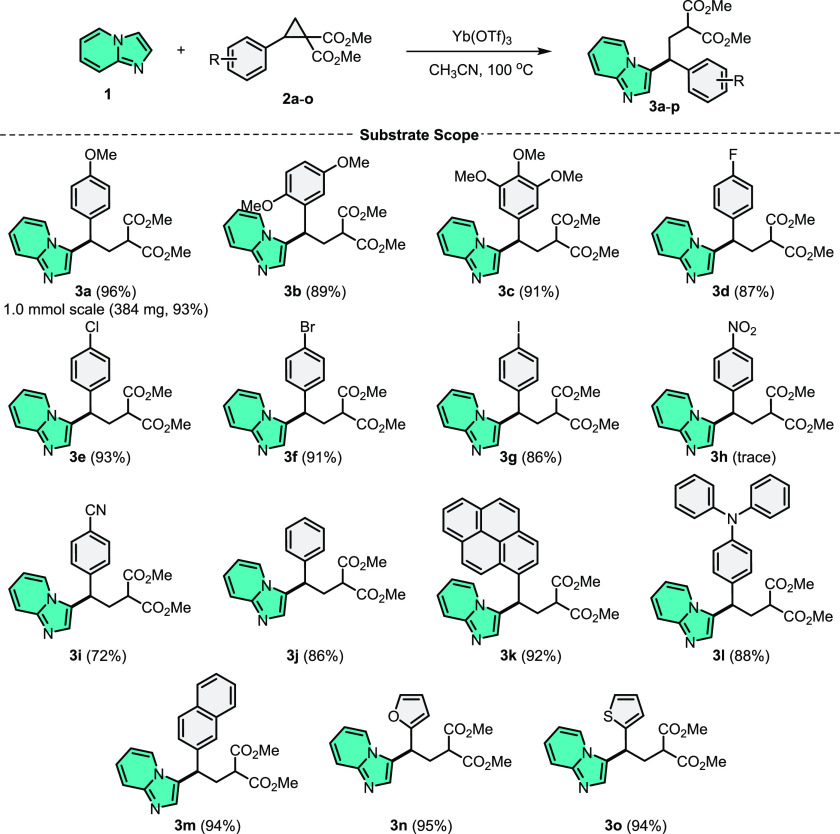
C3–H Alkylation of Imidazo[1,2-*a*]pyridine with Diverse DA-Cyclopropanes^,^ General conditions: Imidazo[1,2-*a*]pyridine (**1**) (0.5 mmol), DA-cyclopropane (0.5 mmol), Yb(OTf)_3_ (25 mol %), CH_3_CN (3.0 mL), and 100 °C (in an oil bath), 24 h. Isolated yields.

With these optimized conditions in hand, we investigated the substrate scope of DA-cyclopropanes (Scheme 2). DA-cyclopropanes bearing electron-donating groups gave the C3-alkylation products of imidazo[1,2-*a*]pyridine (**1**) (**3a**–**3c**) in very high yields (89–96%). Furthermore, *p*-halogenated DA-cyclopropanes provided the related alkylation products of imidazopyridine (**3d**–**3g**) in a satisfactory yield (86–93%). It was observed that the yield of alkylation products decreased in DA-cyclopropanes containing electron-withdrawing groups. When we evaluated DA-cyclopropane bearing −NO_2_ groups on the *para*-position, trace amounts of product **3h** were observed. Moreover, DA-cyclopropanes bearing the *p*-CN group yielded alkylation product **3i** in 72% yield. In addition, we observed that aryl-substituted DA-cyclopropanes had no remarkable difference compared to the optimized reaction, and the corresponding alkylation products (**3j**–**3m**) were obtained in quite high yields (86–94%). Finally, DA-cyclopropanes bearing furan and thiophene provided the corresponding products (**3n** and **3o**) in excellent yields (95% and 94%, respectively). When we scaled up the reaction to 1.0 mmol using the model substrates **1** and **2a**, we noticed only a minor reduction in yield (93%).

Within the scope of the study, we examined the nucleophilic ring-opening reactions of 2-phenylimidazopyridines with DA-cyclopropanes (Table 2). When we applied the optimum conditions for the reaction of 2-phenylimidazo[1,2-*a*]pyridine (**4**) and DA-cyclopropane **2a**, we obtained the C3-alkylation product **5a** in 85% yield (Table 2, entry 1). When the catalyst was replaced by Gd(OTf)_3_ under the same conditions, product **5a** was obtained in 87% yield with 5 mol % catalyst loading (Table 2, entry 2). Next, the reaction of 2-phenylimidazo[1,2-*a*]pyridine with DA-cyclopropane **2a** was performed with 15 mol % of Gd(OTf)_3_ and **5a** was obtained in 95% yield (Table 2, entry 3). Zn(OTf)_2_ and Bi(OTf)_3_ were not effective the reaction of 2-phenylimidazo[1,2-*a*]pyridine (**4**) and DA-cyclopropane **2a** (Table 2, entries 4 and 5). Furthermore, the reaction in Sc(OTf)_3_ (10 mol %) gave slightly less yield of the desired product (Table 2, entry 6).

**Table 2:**
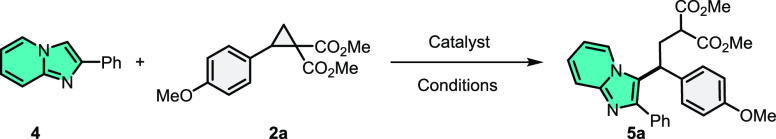
Optimization of Reaction Conditions^a^^,^^b^

entry	catalyst	mol (%)	solvent	time (h)	yield (%)^b^
1	Yb(OTf)_3_	25	CH_3_CN	24	85
2	Gd(OTf)_3_	5	DCE	24	87
3	Gd(OTf)_3_	15	DCE	24	95
4	Zn(OTf)_2_	10	DCE	12	ND
5	Bi(OTf)_3_	10	DCE	12	ND
6	Sc(OTf)_3_	10	DCE	12	30

a Conditions: 2-Phenylimidazo[1,2-*a*]pyridine (1.0 mmol), donor–acceptor cyclopropane (1.0 mmol), solvent (3.0 mL), 100 °C (in oil bath).

b Isolated yields. ND = not determined.

**Scheme 3:**
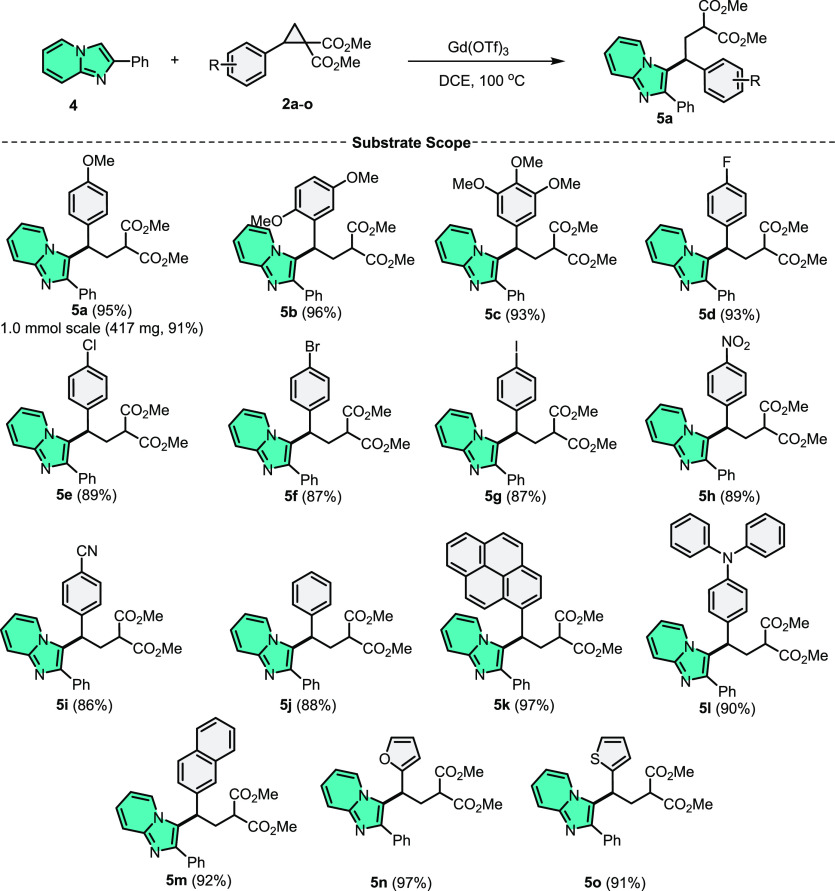
C3–H Alkylation of 2-Phenylimidazo[1,2-*a*]pyridine with Diverse DA-Cyclopropanes^,^ General conditions: 2-Phenylimidazo[1,2-*a*]pyridine (**4**) (0.5 mmol), DA-cyclopropane (0.5 mmol), Gd(OTf)_3_ (15 mol %), CH_3_CN (3.0 mL), and 100 °C (in an oil bath), 24 h. Isolated yields.

We then examined the scope of the reactions for different DA-cyclopropanes using these improved settings (Scheme 3). The reaction of 2-phenylimidazo[1,2-*a*]pyridine (**4**) with electron-donating groups bearing DA-cyclopropanes yielded C3-alkylated compounds (**5a**–**c**) in excellent yields (93–95%). *p*-Halogenated DA-cyclopropanes were produced in high yields (87–93%). When the reactions were carried out with *p*-NO_2_ and *p*-CN DA-cyclopropane, the C3-alkylation products **5h** and **5i** were obtained in 89% and 86%, respectively. Then, we investigated aryl-substituted DA-cyclopropanes. The reaction of 2-phenylimidazo[1,2-*a*]pyridine (**4**) with aryl DA-cyclopropanes yielded the intended products (**5j**–**m**) in high yields (88–92%). DA-cyclopropanes containing furan and thiophene provided the relevant products (**5n** and **5o**) in excellent yields (97% and 91%, respectively). In scaling up the reaction to 1.0 mmol using the substrates **4** and **2a**, we observed only a slight decrease in yield to 91%, highlighting the practicability of the current method.

**Scheme 4:**
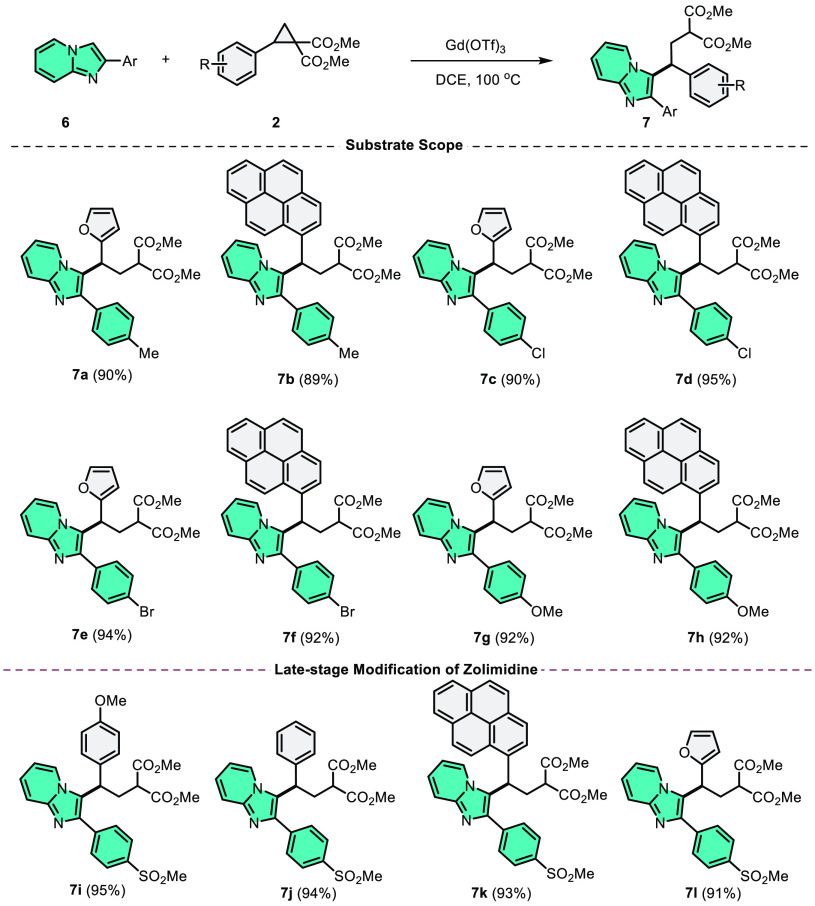
C3–H Alkylation of 2-Arylimidazo[1,2-*a*]pyridines and Late-Stage C3–H Alkylation of Zolimidine with Diverse DA-Cyclopropanes^,^ General conditions: 2-Arylimidazo[1,2-*a*]pyridine (**6**) (0.5 mmol), DA-cyclopropane (0.5 mmol), Gd(OTf)_3_ (15 mol %), DCE (3.0 mL), and 100 °C (in an oil bath), 24 h. Isolated yields.

At this point, after establishing the scope of DA-cyclopropanes with 2-phenylimidazo[1,2-*a*]pyridine, our focus shifted toward investigating the ring-opening scope of 2-arylimidazo[1,2-*a*]pyridines (Scheme 4). Gratifyingly, the reactions of various 2-arylimidazo[1,2-*a*]pyridines with DA-cyclopropanes under optimized conditions showed similar results. Ring-opening products **7a**–**7h** were obtained in high yields (89–95%) using DA-cyclopropanes containing *p*-Cl, *p*-Br, *p*-Me, and *p*-OMe. The late-stage modification of medicinal molecules such as zolimidine, a medication for peptic ulcer and gastroesophageal reflux illness, with DA-cyclopropanes, might be performed using a straightforward and effective catalytic method. As a result of these reactions, the ring-opening products of zolimidine with DA-cyclopropanes were precisely synthesized in high yields (91–95%).

**Scheme 5:**
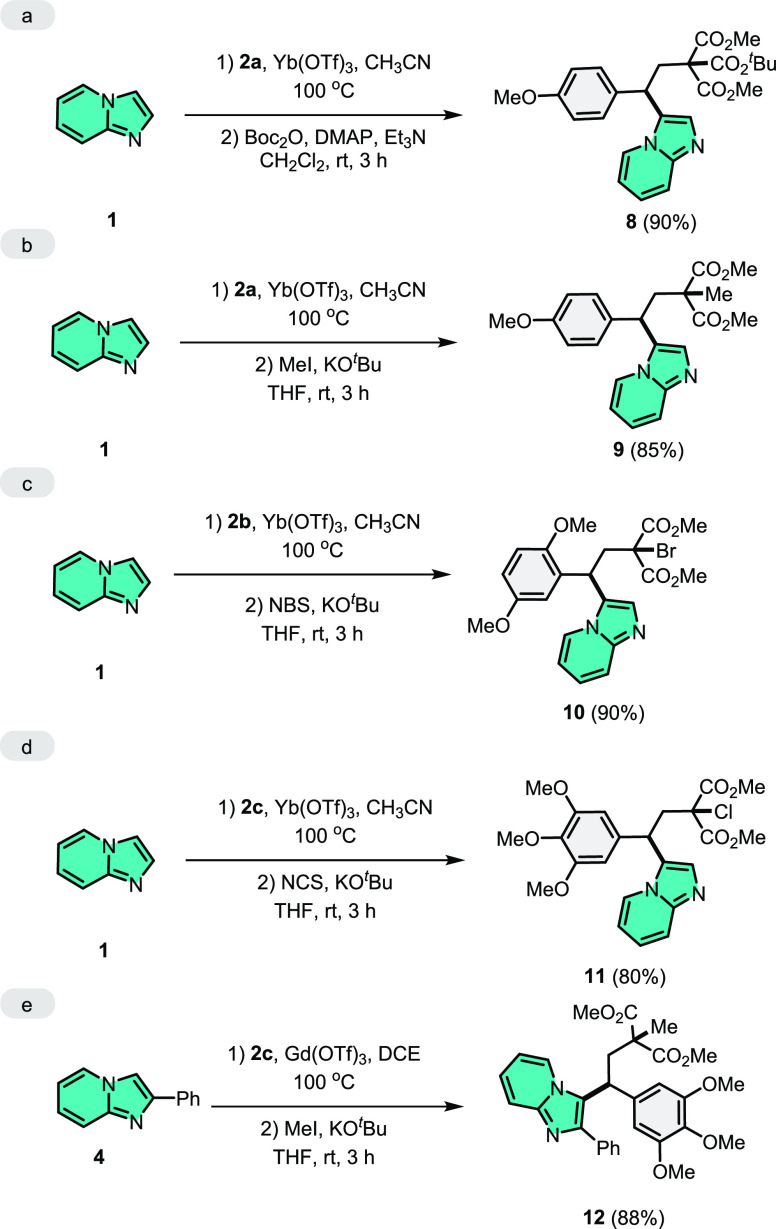
1,3-Bisfunctionalization of DA-Cyclopropanes

In the last part of this study, ring-opening 1,3-bisfunctionalization transformations were investigated (Scheme 5). First of all, 1,3-bisfuctionalized triester **8** was easily obtained from imidazo[1,2-*a*]pyridine (**1**) under the optimum reaction conditions and then in the presence of Boc_2_O and DMAP in 90% yield (Scheme 5a). Next, 1,3-bisfunctionalized methylation product **9** was readily generated in 85% yield (Scheme 5b). The halogenation products in the malonate moiety were obtained using *N*-bromosuccinimide (NBS) and *N*-chlorosuccinimide (NCS) in 90 and 80% yields, respectively (Scheme 5c,d). Finally, the 1,3-bisfunctionalized reaction is not restricted to imidazo[1,2-*a*]pyridine as the nucleophilic component. When the methylation reaction was carried out in the presence of 2-phenylimidazo[1,2-*a*]pyridine (**4**), the 1,3-bisfunctioanalization product **12** was formed in 88% yield (Scheme 5e).

**Scheme 6:**
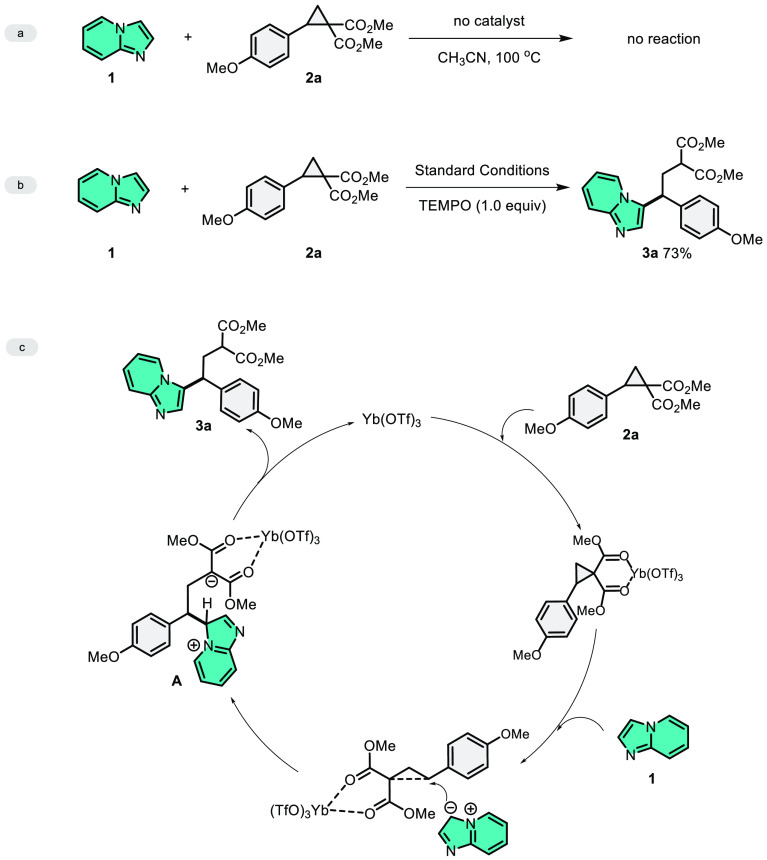
Control Experiments and the Proposed Mechanism of the Cascade Reactions

We did control reactions to understand the mechanism. In the absence of Yb(OTf)_3_, the reaction of imidazo[1,2-*a*]pyridine (**1**) and DA-cyclopropane **2a** did not provided any product (Scheme 6a). In the presence of TEMPO, the yield of product **3a** was decreased to 73% but was not completely quenched (Scheme 6b). This result confirms that the reaction is going to occur with the ionic mechanism. A plausible mechanism was proposed with experimental results and the precedent report previously.^34,36^ We hypothesize that in the first stage, an interaction occurs between DA-cyclopropane and Lewis acid, and a partial positive charge is formed on the bonded carbon of the donor group, and a partial negative charge is formed on the carbon to which the acceptor groups are attached. We hypothesize that following the formation of these interactions, ring opening occurs when the imidazo[1,2-*a*]pyridine attacks from the C3 position to the carbon atom to which the donor group of DA-cyclopropane is attached, followed by the formation of C3-alkylated imidazo[1,2-*a*]pyridine **3a**.

## Conclusions

In summary, we developed a new method for C3–H alkylation of imidazo[1,2-*a*]pyridines using DA-cyclopropanes via nucleophilic ring opening. The current methodology is suitable for both imidazo[1,2-*a*]pyridine and 2-arylimidazo[1,2-*a*]pyridines. DA-cyclopropanes with various substitution patterns yielded the corresponding C3-alkylated products in good-to-excellent yields. We also performed the 1,3-bisfunctionalization of DA-cyclopropanes. To the best of our knowledge, this is the first report for C3–H alkylation of imidazo[1,2-*a*]pyridines using DA-cyclopropanes.

## Experimental Section

### General Experimental Methods

^1^H NMR and ^13^C NMR experiments were performed on either 400 MHz Varian or 400 MHz Bruker Avance II instruments using CDCl_3_, DMSO-*d_6_*, MeOD, and acetone-*d_6_* as the solvent with tetramethylenesilane (TMS) as the internal standard at room temperature, and the coupling constants *J* are given in hertz. The multiplicity is designated as s (singlet), d (doublet) t (triplet) q (quartet), dd (doublet of doublets), and m (multiplet). All products were further characterized by high-resolution mass spectra (HRMS). HRMS were obtained using a QTOF (quadrupole time-of-flight) spectrometry device. IR spectra were recorded using a Shimadzu IRSpirit FT-IR spectrophotometer. Column chromatography and thin layer chromatography (TLC) were performed using silica gel high-purity grade (7734), pore size 60 Å, 70–230 mesh (Sigma-Aldrich). The imidazo[1,2-*a*]pyridine (**1**) commercially available reagent was purchased from Sigma-Aldrich. All donor–acceptor cyclopropanes (**2a**–**2o**) were synthesized via literature methods.^55^

### General Procedure 1 (GP1): Preparation of 3-Substitute Imidazo[1,2-*a*]pyridine (**3a–o**)

A solution of imidazo[1,2-*a*]pyridine (**1**) (0.5 mmol, 1.0 equiv), DA-cyclopropane (**2**) (0.5 mmol, 1.0 equiv), and Yb(OTf)_3_ (25 mol %) in CH_3_CN (3.0 mL) was charged into a sealed tube and heated to 100 °C in an oil bath for 24 h. The reaction mixture was cooled to room temperature and then the solvent was evaporated. The residue was purified using preparative thin layer chromatography on silica gel (TLC) over EtOAC:hexane then DCM:MeOH.

#### Dimethyl 2-(2-(Imidazo[1,2-*a*]pyridin-3-yl)-2-(4-methoxyphenyl)ethyl)malonate (**3a**)

TLC (EtOAC:hexane (1.5:8.5) then DCM:MeOH (9.5:0.5)) gave the product as a colorless oil (186 mg, 96% yield). ^1^H NMR (400 MHz, CDCl_3_): δ 8.94 (d, *J* = 6.7 Hz, 1H), 8.41 (s, 1H), 8.09 (d, *J* = 8.9 Hz, 1H), 7.94–7.88 (m, 2H), 7.47–7.35 (m, 2H), 6.92 (d, *J* = 8.9 Hz, 2H), 5.92 (t, *J* = 7.7 Hz, 1H), 3.78 (s, 3H), 3.71 (s, 3H), 3.67 (s, 3H), 3.39 (t, *J* = 7.0 Hz, 1H), 2.93 (t, *J* = 7.7 Hz, 2H). ^13^C{^1^H} NMR (100 MHz, CDCl_3_): δ 168.9, 168.6, 160.7, 138.6, 134.5, 130.4, 129.0, 126.3, 122.4, 118.0, 115.1, 111.2, 59.3, 55.4, 53.2, 53.1, 48.2, 32.8 (1C signal overlaps). FT-IR: ν 2930, 2373, 1735, 1653, 1559, 1255, 1228, 1161, 1029, 764, 635 cm^–1^. HRMS (ESI-TOF) *m*/*z*: [M + H]^+^ calcd for C_21_H_23_N_2_O_5_, 383.1601; found 383.1600.

#### Dimethyl 2-(2-(2,5-Dimethoxyphenyl)-2-(imidazo[1,2-*a*]pyridin-3-yl)ethyl)malonate (**3b**)

TLC (EtOAC:hexane (2:8) then DCM:MeOH (9.5:0.5)) gave the product as a light yellow oil (187 mg, 89% yield). ^1^H NMR (400 MHz, CDCl_3_): δ 8.94 (d, *J* = 6.7 Hz, 1H), 8.41 (d, *J* = 2.4 Hz, 1H), 8.10–7.85 (m, 2H), 7.46–7.38 (m, 1H), 7.04 (d, *J* = 2.4 Hz, 1H), 6.95–6.76 (m, 2H), 6.19 (t, *J* = 7.8 Hz, 1H), 3.75 (s, 3H), 3.69 (s, 3H), 3.67 (s, 3H), 3.65 (s, 3H), 3.42–3.30 (m, 1H), 3.06–2.91 (m, 2H). ^13^C{^1^H} NMR (100 MHz, CDCl_3_): δ 168.7, 168.6, 154.1, 151.1, 138.9, 134.4, 130.4, 123.5, 122.7, 117.9, 115.7, 113.7, 112.5, 111.0, 56.0 (2C), 53.7, 53.1, 53.0, 48.2, 31.6 (1C signal overlaps). FT-IR: ν 3103, 2926, 2322, 1733, 1653, 1559, 1506, 1305, 1142, 741 cm^–1^. HRMS (ESI-TOF) *m*/*z*: [M + H]^+^ calcd for C_22_H_25_N_2_O_6_, 413.1707; found 413.1709.

#### Dimethyl 2-(2-(Imidazo[1,2-*a*]pyridin-3-yl)-2-(3,4,5-trimethoxyphenyl)ethyl)malonate (**3c**)

TLC (EtOAC:hexane (2:8) then DCM:MeOH (9.5:0.5)) gave the product as a light yellow oil (204 mg, 91% yield). ^1^H NMR (400 MHz, CDCl_3_): δ 8.84 (d, *J* = 6.8 Hz, 1H), 8.35–8.32 (m, 1H), 8.21 (d, *J* = 9.3 Hz, 1H), 8.14–8.12 (m, 1H), 7.97–7.84 (m, 1H), 7.39 (t, *J* = 6.8 Hz, 1H), 6.71 (s, 1H), 5.91 (t, *J* = 7.8 Hz, 1H), 3.83 (s, 6H), 3.77 (s, 3H), 3.70 (s, 3H), 3.65 (s, 3H), 3.42 (t, *J* = 6.9 Hz, 1H), 2.95 (t, *J* = 7.3 Hz, 2H). ^13^C{^1^H} NMR (100 MHz, CDCl_3_): δ 168.8, 168.6, 154.1 (2C), 138.9, 138.8, 134.6, 130.3, 130.1, 122.7, 118.1, 111.4, 104.8, 60.7, 59.8, 56.5, 53.1 (2C), 48.3, 32.8. FT-IR: ν 3101, 2379, 1717, 1635, 1559, 1506, 1305, 1141, 1126, 898, 738, 706 cm^–1^. HRMS (ESI-TOF) *m*/*z*: [M + H]^+^ calcd for C_23_H_27_N_2_O_7_, 443.1813; found 443.1810.

#### Dimethyl 2-(2-(4-Fluorophenyl)-2-(imidazo[1,2-*a*]pyridin-3-yl)ethyl)malonate (**3d**)

TLC (EtOAC:hexane (2:8) then DCM:MeOH (9.5:0.5)) gave the product as a light yellow oil (164 mg, 87% yield). ^1^H NMR (400 MHz, CDCl_3_): δ 8.95 (d, *J* = 6.8 Hz, 1H), 8.44 (s, 1H), 8.15–8.09 (m, 2H), 7.99–7.84 (m, 1H), 7.51 (dd, *J* = 8.5, 5.1 Hz, 1H), 7.41 (t, *J* = 6.8 Hz, 1H), 7.05 (t, *J* = 8.5 Hz, 2H), 6.05 (t, *J* = 7.7 Hz, 1H), 3.65 (s, 3H), 3.63 (s, 3H), 3.41 (t, *J* = 7.0 Hz, 1H), 3.03–2.89 (m, 2H). ^13^C{^1^H} NMR (100 MHz, CDCl_3_): δ 168.9, 168.7, 163.4 (d, *J* = 250.0 Hz), 139.0 (2C), 135.0, 131.3 (d, *J* = 3.2 Hz), 130.6, 129.9 (d, *J* = 8.4 Hz), 122.7, 118.3, 116.9 (d, *J* = 21.8 Hz), 111.5, 59.2, 59.1, 53.3, 48.5, 33.1. FT-IR: ν 3123, 2954, 2322, 1733, 1508, 1255, 1248, 1152, 1029, 842, 735, 639 cm^–1^. HRMS (ESI-TOF) *m*/*z*: [M + H]^+^ calcd for C_20_H_20_FN_2_O_4_, 371.1402; found 371.1400.

#### Dimethyl 2-(2-(4-Chlorophenyl)-2-(imidazo[1,2-*a*]pyridin-3-yl)ethyl)malonate (**3e**)

TLC (EtOAC:hexane (2:8) then DCM:MeOH (9.5:0.5)) gave the product as a light yellow oil (183 mg, 93% yield). ^1^H NMR (400 MHz, CDCl_3_): δ 9.07 (d, *J* = 6.3 Hz, 1H), 8.57 (s, 1H), 8.11–7.99 (m, 2H), 7.97–7.85 (m, 1H), 7.45–7.38 (m, 4H), 6.06 (t, *J* = 7.6 Hz, 1H), 3.71 (s, 3H), 3.68 (s, 3H), 3.42 (t, *J* = 6.9 Hz, 1H), 2.98 (t, *J* = 7.1 Hz, 2H). ^13^C{^1^H} NMR (100 MHz, CDCl_3_): δ 168.7, 168.5, 138.8, 135.8, 134.8, 133.8, 130.5, 129.9, 129.0, 122.6, 118.1, 111.2, 58.9, 53.1 (2C), 48.2, 32.7 (1C signal overlaps). FT-IR: ν 3127, 2958, 1730, 1645, 1519, 1253, 1223, 1149, 1028, 756, 636 cm^–1^. HRMS (ESI-TOF) *m*/*z*: [M + H]^+^ calcd for C_20_H_20_ClN_2_O_4_, 387.1106; found 387.1102.

#### Dimethyl 2-(2-(4-Bromophenyl)-2-(imidazo[1,2-*a*]pyridin-3-yl)ethyl)malonate (**3f**)

TLC (EtOAC:hexane (2:8) then DCM:MeOH (9.5:0.5)) gave the product as a colorless oil (199 mg, 91% yield). ^1^H NMR (400 MHz, CDCl_3_): δ 9.00 (d, *J* = 6.6 Hz, 1H), 8.49 (s, 1H), 8.11–8.06 (m, 2H), 7.99–7.86 (m, 1H), 7.52 (d, *J* = 8.4 Hz, 2H), 7.44–7.38 (m, 2H), 6.05 (t, *J* = 7.7 Hz, 1H), 3.69 (s, 3H), 3.66 (s, 3H), 3.42 (t, *J* = 7.0 Hz, 1H), 2.98 (t, *J* = 8.2 Hz, 2H). ^13^C{^1^H} NMR (100 MHz, CDCl_3_): δ 168.7, 168.50, 138.8, 134.7, 134.2 (2C), 132.9, 130.6, 129.2, 124.2, 122.6, 118.1, 111.2, 59.0, 53.2, 53.1, 48.1, 32.7. FT-IR: ν 3124, 2923, 1732, 1645, 1520, 1255, 1223, 1152, 1028, 756, 636 cm^–1^. HRMS (ESI-TOF) *m*/*z*: [M + H]^+^ calcd for C_20_H_20_BrN_2_O_4_, 431.0601; found 431.0598.

#### Dimethyl 2-(2-(Imidazo[1,2-*a*]pyridin-3-yl)-2-(4-iodophenyl)ethyl)malonate (**3g**)

TLC (EtOAC:hexane (2:8) then DCM:MeOH (9.5:0.5)) gave the product as a light yellow oil (210 mg, 86% yield). ^1^H NMR (400 MHz, CDCl_3_): δ 8.94 (d, *J* = 6.7 Hz, 1H), 8.42 (d, *J* = 1.9 Hz, 1H), 8.12–8.08 (m, 2H), 7.98–7.90 (m, 1H), 7.74 (d, *J* = 8.3 Hz, 2H), 7.45 (t, *J* = 6.7 Hz, 1H), 7.28 (d, *J* = 2.5 Hz, 1H), 6.02 (t, *J* = 7.7 Hz, 1H), 3.72 (s, 3H), 3.69 (s, 3H), 3.44 (t, *J* = 7.0 Hz, 1H), 3.06–2.97 (m, 2H). ^13^C{^1^H} NMR (100 MHz, CDCl_3_): δ 168.7, 168.5, 138.9, 138.8, 134.8 (2C), 134.7, 130.4, 129.3, 122.6, 118.2, 111.2, 96.0, 59.1, 53.2 (2C), 48.1, 32.6. FT-IR: ν 3128, 2923, 1730, 1645, 1518, 1252, 1223, 1151, 1028, 1006, 756, 636 cm^–1^. HRMS (ESI-TOF) *m*/*z*: [M + H]^+^ calcd for C_20_H_20_IN_2_O_4_, 479.0462; found 479.0466.

#### Dimethyl 2-(2-(4-Cyanophenyl)-2-(imidazo[1,2-*a*]pyridin-3-yl)ethyl)malonate (**3i**)

TLC (EtOAC:hexane (3:7) then DCM:MeOH (9.5:0.5)) gave the product as a light yellow oil (138 mg, 72% yield). ^1^H NMR (400 MHz, CDCl_3_): δ 8.94 (d, *J* = 6.7 Hz, 1H), 8.44 (s, 1H), 8.26 (s, 1H), 8.03 (d, *J* = 9.2 Hz, 1H), 7.97–7.83 (m, 1H), 7.74–7.62 (m, 3H), 7.46 (t, *J* = 6.7 Hz, 1H), 6.18 (t, *J* = 7.6 Hz, 1H), 3.69 (s, 3H), 3.67 (s, 3H), 3.43 (t, *J* = 6.9 Hz, 1H), 3.09–3.02 (m, 2H). ^13^C{^1^H} NMR (100 MHz, CDCl_3_): δ 168.6, 168.4, 140.4, 139.0, 134.9, 133.5, 130.6, 128.3, 122.8, 118.3, 117.8, 116.9, 113.8, 111.1, 59.0, 53.2 (2C), 48.0, 32.6. FT-IR: ν 3126, 2957, 2230, 1732, 1646, 1520, 1252, 1223, 1152, 1028, 758, 636 cm^–1^. HRMS (ESI-TOF) *m*/*z*: [M + H]^+^ calcd for C_21_H_20_N_3_O_4_, 378.1448; found 378.1445.

#### Dimethyl 2-(2-(Imidazo[1,2-*a*]pyridin-3-yl)-2-phenylethyl)malonate (**3j**)

TLC (EtOAC:hexane (2:8) then DCM:MeOH (9.5:0.5)) gave the product as a light yellow oil (154 mg, 86% yield). ^1^H NMR (400 MHz, CDCl_3_): δ 8.95 (d, *J* = 6.8 Hz, 1H), 8.44 (d, *J* = 2.1 Hz, 1H), 8.05 (d, *J* = 9.2 Hz, 1H), 7.99 (d, *J* = 2.1 Hz, 1H), 7.94–7.84 (m, 1H), 7.50–7.32 (m, 5H), 5.99 (t, *J* = 7.7 Hz, 1H), 3.70 (s, 3H), 3.66 (s, 3H), 3.42 (t, *J* = 7.0 Hz, 1H), 2.98 (t, *J* = 7.4 Hz, 2H). ^13^C{^1^H} NMR (100 MHz, CDCl_3_): δ 168.8, 168.6, 138.8, 135.0 (2C), 134.7, 130.5, 130.0, 129.8, 127.4, 122.5, 118.1, 111.2, 59.7, 53.1 (2C), 48.2, 32.7. FT-IR: ν 3128, 2956, 1730, 1646, 1520, 1253, 1223, 1148, 1028, 756, 635 cm^–1^. HRMS (ESI-TOF) *m*/*z*: [M + H]^+^ calcd for C_20_H_21_N_2_O_4_, 353.1496; found 353.1495.

#### Dimethyl 2-(2-(Imidazo[1,2-*a*]pyridin-3-yl)-2-(pyren-1-yl)ethyl)malonate (**3k**)

TLC (EtOAC:hexane (3:7) then DCM:MeOH (9.5:0.5)) gave the product as a light yellow oil (219 mg, 92% yield). ^1^H NMR (400 MHz, CDCl_3_): δ 8.86 (d, *J* = 6.7 Hz, 1H), 8.38 (d, *J* = 2.0 Hz, 1H), 8.32 (d, *J* = 9.4 Hz, 1H), 8.26–8.16 (m, 4H), 8.11–7.94 (m, 5H), 7.77–7.71 (m, 1H), 7.29–7.26 (m, 1H), 7.13 (t, *J* = 7.6 Hz, 1H), 3.68 (s, 3H), 3.66 (s, 3H), 3.63–3.58 (m, 1H), 3.34–3.22 (m, 2H). ^13^C{^1^H} NMR (100 MHz, CDCl_3_): δ 169.0, 168.7, 138.8, 134.7, 132.2, 131.1, 130.5, 130.3, 130.1, 128.9, 128.8, 127.2, 127.1, 126.7, 126.5, 126.1, 125.7, 125.0, 124.3, 123.6, 122.9, 120.4, 118.0, 110.8, 55.6, 53.2, 53.1, 48.2, 33.4 (1C signal overlaps). FT-IR: ν 3127, 2923, 1733, 1646, 1516, 1253, 1223, 1151, 1028, 846, 755, 721, 635 cm^–1^. HRMS (ESI-TOF) *m*/*z*: [M + H]^+^ calcd for C_30_H_25_N_2_O_4_, 477.1809; found 477.1816.

#### Dimethyl 2-(2-(4-(Diphenylamino)phenyl)-2-(imidazo[1,2-*a*]pyridin-3-yl)ethyl)malonate (**3l**)

TLC (EtOAC:hexane (3:7) then DCM:MeOH (9.5:0.5)) gave the product as a light yellow oil (231 mg, 88% yield). ^1^H NMR (400 MHz, CDCl_3_): δ 8.96 (d, *J* = 6.8 Hz, 1H), 8.44 (s, 1H), 8.03 (d, *J* = 9.2 Hz, 1H), 7.95–7.82 (m, 1H), 7.80 (d, *J* = 2.1 Hz, 1H), 7.37 (t, *J* = 6.8 Hz, 1H), 7.26–7.10 (m, 6H), 7.10–6.89 (m, 7H), 5.82 (t, *J* = 7.6 Hz, 1H), 3.66 (s, 3H), 3.63 (s, 3H), 3.38 (t, *J* = 7.0 Hz, 1H), 2.85 (t, *J* = 7.6 Hz, 2H). ^13^C{^1^H} NMR (100 MHz, CDCl_3_): δ 168.9, 168.6, 149.5, 146.8, 138.6, 134.5, 130.8, 129.6, 128.4, 126.2, 125.4, 124.1, 122.4, 122.2, 118.0, 110.9, 59.3, 53.2, 53.1, 48.2, 32.7 (1C signal overlaps). FT-IR: ν 3133, 2953, 1733, 1589, 1508, 1489, 1253, 1225, 1159, 1028, 755, 696, 638 cm^–1^. HRMS (ESI-TOF) *m*/*z*: [M + H]^+^ calcd for C_32_H_30_N_3_O_4_, 520.2231; found 520.2236.

#### Dimethyl 2-(2-(Imidazo[1,2-*a*]pyridin-3-yl)-2-(naphthalen-2-yl)ethyl)malonate (**3m**)

TLC (EtOAC:hexane (3:7) then DCM:MeOH (9.5:0.5)) gave the product as a light yellow oil (192 mg, 94% yield). ^1^H NMR (400 MHz, CDCl_3_): δ 8.90 (d, *J* = 6.7 Hz, 1H), 8.42 (d, *J* = 2.0 Hz, 1H), 8.13 (d, *J* = 9.3 Hz, 1H), 8.06 (d, *J* = 2.0 Hz, 1H), 8.02 (s, 1H), 7.91–7.78 (m, 3H), 7.77–7.70 (m, 1H), 7.48–7.40 (m, 2H), 7.31 (t, *J* = 6.9 Hz, 1H), 6.17 (t, *J* = 7.8 Hz, 1H), 3.66 (s, 3H), 3.59 (s, 3H), 3.50–3.43 (m, 1H), 3.12–3.02 (m, 2H). ^13^C{^1^H} NMR (100 MHz, CDCl_3_): δ 168.8, 168.6, 138.8, 134.6, 133.5 (2C), 133.1, 132.3, 130.4, 130.0, 128.4, 127.7, 127.4, 127.1, 123.9, 122.6, 118.0, 116.5, 111.3, 59.8, 53.1 (2C), 48.3, 32.7. FT-IR: ν 3126, 2359, 1733, 1646, 1518, 1253, 1222, 1151, 1028, 869, 755, 635 cm^–1^. HRMS (ESI-TOF) *m*/*z*: [M + H]^+^ calcd for C_24_H_23_N_2_O_4_, 403.1652; found 403.1638.

#### Dimethyl 2-(2-(Furan-2-yl)-2-(imidazo[1,2-*a*]pyridin-3-yl)ethyl)malonate (**3n**)

TLC (EtOAC:hexane (2:8) then DCM:MeOH (9.7:0.3)) gave the product as a light yellow oil (165 mg, 95% yield). ^1^H NMR (400 MHz, CDCl_3_): δ 8.95 (d, *J* = 6.7 Hz, 1H), 8.41 (s, 1H), 8.21 (d, *J* = 9.2 Hz, 1H), 8.03–7.92 (m, 1H), 7.83 (d, *J* = 2.2 Hz, 1H), 7.45–7.44 (m, 1H), 6.75 (d, *J* = 3.3 Hz, 1H), 6.40–6.38 (m, 1iH), 6.24–6.12 (m, 1H), 3.67 (s, 6H), 3.47–3.43 (m, 1H), 3.00–2.92 (m, 1H), 2.90–2.79 (m, 1H). ^13^C{^1^H} NMR (100 MHz, CDCl_3_): δ 168.7, 168.4, 147.1, 144.5, 138.6, 134.9, 130.5, 122.9, 118.2, 111.8, 111.2, 111.1, 53.4, 53.2 (2C), 48.0, 31.8 (1C signal overlaps). FT-IR: ν 3131, 2954, 1733, 1646, 1522, 1250, 1223, 1151, 1028, 755, 635 cm^–1^. HRMS (ESI-TOF) *m*/*z*: [M + H]^+^ calcd for C_18_H_19_N_2_O_5_, 343.1288; found 343.1286.

#### Dimethyl 2-(2-(Imidazo[1,2-*a*]pyridin-3-yl)-2-(thiophen-2-yl)ethyl)malonate (**3o**)

TLC (EtOAC:hexane (2:8) then DCM:MeOH (9.7:0.3)) gave the product as a light yellow oil (172 mg, 94% yield). ^1^H NMR (400 MHz, CDCl_3_): δ 9.01 (d, *J* = 6.8 Hz, 1H), 8.48 (s, 1H), 8.23 (d, *J* = 9.2 Hz, 1H), 8.02–7.92 (m, 1H), 7.86 (d, *J* = 2.0 Hz, 1H), 7.43 (t, *J* = 6.8 Hz, 1H), 7.40–7.33 (m, 1H), 7.01–6.99 (m, 1H), 6.37 (t, *J* = 7.7 Hz, 1H), 3.66 (s, 3H), 3.65 (s, 3H), 3.50 (t, *J* = 7.0 Hz, 1H), 2.95 (t, *J* = 7.3 Hz, 2H). ^13^C{^1^H} NMR (100 MHz, CDCl_3_): δ 168.7, 168.4, 138.6, 137.2, 134.9, 130.6, 128.7, 128.0, 127.7, 122.3, 118.2, 111.2, 55.1, 53.2 (2C), 48.3, 34.2 (1C signal overlaps). FT-IR: ν 3124, 2956, 1733, 1646, 1518, 1252, 1223, 1151, 1028, 853, 756, 715, 635 cm^–1^. HRMS (ESI-TOF) *m*/*z*: [M + H]^+^ calcd for C_18_H_19_N_2_O_4_S, 359.1060; found 359.1058.

### General Procedure 2 (GP2): Preparation of 3-Substitute 2-Phenylimidazo[1,2-*a*]pyridine (**5a–o**)

A solution of 2-phenylimidazo[1,2-*a*]pyridine (**4**) (0.5 mmol, 1.0 equiv), DA-cyclopropane (**2**) (0.5 mmol, 1.0 equiv), and Gd(OTf)_3_ (15 mol %) in DCE (3.0 mL) was charged into a sealed tube and heated to 100 °C in an oil bath for 24 h. The reaction mixture was cooled to room temperature and then the solvent was evaporated. The residue was purified using preparative thin layer chromatography on silica gel (TLC) over EtOAC:hexane.

#### Dimethyl 2-(2-(4-Methoxyphenyl)-2-(2-phenylimidazo[1,2-*a*]pyridin-3-yl)ethyl)malonate (**5a**)

TLC (EtOAC:hexane (3:7)) gave the product as a light yellow oil (220 mg, 95% yield). ^1^H NMR (400 MHz, CDCl_3_) δ 7.66–7.56 (m, 4H), 7.39–7.33 (m, 2H), 7.30 (d, *J* = 7.4 Hz, 1H), 7.18 (d, *J* = 8.9 Hz, 2H), 7.14–7.06 (m, 1H), 6.81 (d, *J* = 8.9 Hz, 2H), 6.58 (t, *J* = 6.8 Hz, 1H), 4.85–4.77 (m, 1H), 3.73 (s, 3H), 3.38 (s, 3H), 3.29 (s, 3H), 2.93–2.78 (m, 2H), 2.75–2.64 (m, 1H). ^13^C{^1^H} NMR (100 MHz, CDCl_3_): δ 169.2, 168.9, 158.6, 145.6, 145.3, 134.3, 131.0, 129.0, 128.6, 128.1, 128.0, 124.9, 124.6, 119.0, 117.7, 114.5, 112.2, 55.3, 52.6, 52.5, 49.8, 37.2, 29.5. FT-IR: ν 2953, 2926, 2838, 2364, 1749, 1733, 1508, 1246, 1176, 1028, 755, 742, 704 cm^–1^. HRMS (ESI-TOF) *m*/*z*: [M + H]^+^ calcd for C_27_H_27_N_2_O_5_, 459.1914; found 459.1907.

#### Dimethyl 2-(2-(2,5-Dimethoxyphenyl)-2-(2-phenylimidazo[1,2-*a*]pyridin-3-yl)ethyl)malonate (**5b**)

TLC (EtOAC:hexane (3:7)) gave the product as a light yellow oil (237 mg, 96% yield). ^1^H NMR (400 MHz, CDCl_3_): δ 8.01 (d, *J* = 6.9 Hz, 1H), 7.71–7.54 (m, 3H), 7.43–7.31 (m, 3H), 7.21–7.08 (m, 1H), 6.81–6.64 (m, 4H), 5.04–4.88 (m, 1H), 3.68 (s, 3H), 3.64 (s, 3H), 3.51 (s, 3H), 3.50 (s, 3H), 3.14 (t, *J* = 7.2 Hz, 1H), 2.76–2.60 (m, 2H). ^13^C{^1^H} NMR (100 MHz, CDCl_3_): δ 169.4, 169.1, 153.6, 151.5, 144.7, 135.4, 129.6, 129.3, 128.1, 127.7, 124.3, 123.9, 119.8, 117.7, 114.7, 112.03, 112.01, 111.6, 55.7 (2C), 52.5 (2C), 49.7, 33.6, 30.4 (1C signal overlaps). FT-IR: ν 2953, 2926, 2838, 2361, 1749, 1733, 1496, 1436, 1216, 1149, 1024, 736, 699 cm^–1^. HRMS (ESI-TOF) *m*/*z*: [M + H]^+^ calcd for C_28_H_29_N_2_O_6_, 489.2020; found 489.2042.

#### Dimethyl 2-(2-(2-Phenylimidazo[1,2-*a*]pyridin-3-yl)-2-(3,4,5-trimethoxyphenyl)ethyl)malonate (**5c**)

TLC (EtOAC:hexane (3:7)) gave the product as a light yellow oil (244 mg, 93% yield). ^1^H NMR (400 MHz, CDCl_3_): δ 7.72 (d, *J* = 7.0 Hz, 1H), 7.66 (d, *J* = 7.0 Hz, 3H), 7.42 (t, *J* = 7.4 Hz, 2H), 7.36 (d, *J* = 7.3 Hz, 1H), 7.23–7.15 (m, 1H), 6.74–6.66 (m, 1H), 6.51 (s, 2H), 4.84 (dd, *J* = 11.2, 5.2 Hz, 1H), 3.83 (s, 3H), 3.73 (s, 6H), 3.46 (s, 3H), 3.35 (s, 3H), 3.01–2.80 (m, 2H), 2.80–2.69 (m, 1H). ^13^C{^1^H} NMR (100 MHz, CDCl_3_): δ 169.2, 168.9, 153.7, 145.7, 145.3, 137.2, 134.9, 134.3, 129.0, 128.6, 128.1, 124.9, 124.8, 118.7, 117.7, 112.3, 104.2, 60.9, 56.2, 52.6, 52.5, 49.7, 38.1, 29.7 (3C signal overlaps). FT-IR: ν 2953, 2920, 2850, 2361, 1733, 1508, 1239, 1123, 1006, 742, 699 cm^–1^. HRMS (ESI-TOF) *m*/*z*: [M + H]^+^ calcd for C_29_H_31_N_2_O_7_, 519.2126; found 519.2157.

#### Dimethyl 2-(2-(4-Fluorophenyl)-2-(2-phenylimidazo[1,2-*a*]pyridin-3-yl)ethyl)malonate (**5d**)

TLC (EtOAC:hexane (2:8)) gave the product as a light yellow oil (210 mg, 93% yield). ^1^H NMR (400 MHz, CDCl_3_): δ 7.68–7.59 (m, 4H), 7.42 (t, *J* = 7.3 Hz, 2H), 7.39–7.34 (m, 1H), 7.30 (dd, *J* = 8.0, 5.2 Hz, 2H), 7.21–7.15 (m, 1H), 7.07–6.99 (m, 2H), 6.69–6.64 (m, 1H), 4.88 (dd, *J* = 11.4, 4.2 Hz, 1H), 3.44 (s, 3H), 3.34 (s, 3H), 3.00–2.85 (m, 2H), 2.82–2.68 (m, 1H). ^13^C{^1^H} NMR (100 MHz, CDCl_3_) δ 169.3, 169.1, 162.1 (d, *J* = 246.0 Hz), 146.2, 145.7, 135.2 (d, *J* = 3.1 Hz), 134.6, 129.2 (d, *J* = 7.6 Hz), 129.0, 128.7, 125.0, 124.8, 118.6, 118.2, 116.5, 116.1 (d, *J* = 21.7 Hz), 112.5, 52.9, 52.8, 49.7, 37.5, 29.8. FT-IR: ν 2956, 2916, 2850, 2346, 1750, 1719, 1506, 1250, 1220, 1149, 744, 704 cm^–1^. HRMS (ESI-TOF) *m*/*z*: [M + H]^+^ calcd for C_26_H_24_FN_2_O_4_, 447.1715; found 447.1732.

#### Dimethyl 2-(2-(4-Chlorophenyl)-2-(2-phenylimidazo[1,2-*a*]pyridin-3-yl)ethyl)malonate (**5e**)

TLC (EtOAC:hexane (2:8)) gave the product as a light yellow oil (208 mg, 89% yield). ^1^H NMR (400 MHz, CDCl_3_): δ 7.66–7.52 (m, 4H), 7.44–7.23 (m, 5H), 7.20 (d, *J* = 8.2 Hz, 2H), 7.17–7.10 (m, 1H), 6.67–6.57 (m, 1H), 4.82 (dd, *J* = 11.5, 4.7 Hz, 1H), 3.39 (s, 3H), 3.29 (s, 3H), 2.91–2.78 (m, 2H), 2.75–2.63 (m, 1H). ^13^C{^1^H} NMR (100 MHz, CDCl_3_): δ 169.0, 168.8, 145.9, 145.4, 137.8, 134.2, 133.2, 129.3, 128.9, 128.6, 128.4, 128.1, 124.8, 124.6, 118.1, 117.9, 112.4, 52.6, 52.5, 49.6, 37.5, 29.4. FT-IR: ν 2954, 2916, 2844, 2360, 1749, 1717, 1653, 1559, 1249, 1143, 1006, 742, 702 cm^–1^. HRMS (ESI-TOF) *m*/*z*: [M + H]^+^ calcd for C_26_H_24_ClN_2_O_4_, 463.1419; found 463.1421.

#### Dimethyl 2-(2-(4-Bromophenyl)-2-(2-phenylimidazo[1,2-*a*]pyridin-3-yl)ethyl)malonate (**5f**)

TLC (EtOAC:hexane (2:8)) gave the product as a light yellow oil (223 mg, 87% yield). ^1^H NMR (400 MHz, CDCl_3_): δ 7.71–7.59 (m, 4H), 7.50–7.34 (m, 5H), 7.21 (d, *J* = 8.1 Hz, 3H), 6.74–6.66 (m, 1H), 4.86 (dd, *J* = 11.5, 4.6 Hz, 1H), 3.45 (s, 3H), 3.35 (s, 3H), 2.98–2.85 (m, 2H), 2.81–2.70 (m, 1H). ^13^C{^1^H} NMR (100 MHz, CDCl_3_): δ 169.0, 168.8, 145.8, 145.3, 138.3, 134.0, 132.3, 129.0, 128.8, 128.7, 128.2, 125.0, 124.6, 121.3, 118.1, 117.8, 112.5, 52.6, 52.5, 49.6, 37.6, 29.3. FT-IR: ν 2954, 2917, 2850, 2360, 1747, 1717, 1559, 1248, 1149, 1005, 742, 704 cm^–1^. HRMS (ESI-TOF) *m*/*z*: [M + H]^+^ calcd for C_26_H_24_BrN_2_O_4_, 507.0914; found 507.0929.

#### Dimethyl 2-(2-(4-Iodophenyl)-2-(2-phenylimidazo[1,2-*a*]pyridin-3-yl)ethyl)malonate (**5g**)

TLC (EtOAC:hexane (2:8)) gave the product as a light yellow oil (243 mg, 87% yield). ^1^H NMR (400 MHz, CDCl_3_): δ 7.73–7.55 (m, 6H), 7.43 (t, *J* = 7.3 Hz, 2H), 7.39–7.34 (m, 1H), 7.23–7.15 (m, 1H), 7.08 (d, *J* = 8.2 Hz, 2H), 6.68 (t, *J* = 6.6 Hz, 1H), 4.86 (dd, *J* = 11.5, 4.7 Hz, 1H), 3.45 (s, 3H), 3.35 (s, 3H), 2.99–2.83 (m, 2H), 2.82–2.68 (m, 1H). ^13^C{^1^H} NMR (100 MHz, CDCl_3_): δ 169.0, 168.8, 146.2, 145.5, 139.2, 138.2, 134.3, 129.1, 128.9, 128.6, 128.1, 124.7, 124.6, 118.0, 112.4, 92.7, 52.6, 52.5, 49.6, 37.7, 29.2 (1C signal overlaps). FT-IR: ν 2954, 2920, 2846, 2371, 1733, 1653, 1559, 1508, 1458, 1155, 929, 741 cm^–1^. HRMS (ESI-TOF) *m*/*z*: [M + 2H]^+^ calcd for C_26_H_25_IN_2_O_4_, 556.0854; found 556.0850.

#### Dimethyl 2-(2-(4-Nitrophenyl)-2-(2-phenylimidazo[1,2-*a*]pyridin-3-yl)ethyl)malonate (**5h**)

TLC (EtOAC:hexane (3:7)) gave the product as a light yellow oil (212 mg, 89% yield). ^1^H NMR (400 MHz, CDCl_3_): δ 8.22 (d, *J* = 8.9 Hz, 2H), 7.70 (d, *J* = 8.9 Hz, 1H), 7.62 (d, *J* = 6.9 Hz, 2H), 7.57 (d, *J* = 6.9 Hz, 1H), 7.52 (d, *J* = 8.3 Hz, 2H), 7.48–7.38 (m, 3H), 7.24–7.21 (m, 1H), 6.74–6.62 (m, 1H), 5.04–4.94 (m, 1H), 3.48 (s, 3H), 3.37 (s, 3H), 3.02–2.91 (m, 2H), 2.88–2.74 (m, 1H). ^13^C{^1^H} NMR (100 MHz, CDCl_3_): δ 168.9, 168.6, 147.2, 146.4, 145.6, 134.0, 128.9, 128.7, 128.3, 128.1, 125.0, 124.3, 124.1, 118.2, 117.3, 112.7, 52.7, 52.6, 49.5, 38.1, 29.4 (1C signal overlaps). FT-IR: ν 2953, 2920, 2848, 2354, 1733, 1516, 1345, 1226, 1151, 1012, 852, 739, 698 cm^–1^. HRMS (ESI-TOF) *m*/*z*: [M + H]^+^ calcd for C_26_H_24_N_3_O_6_, 474.1660; found 474.1673.

#### Dimethyl 2-(2-(4-Cyanophenyl)-2-(2-phenylimidazo[1,2-*a*]pyridin-3-yl)ethyl)malonate (**5i**)

TLC (EtOAC:hexane (3:7)) gave the product as a light yellow oil (197 mg, 86% yield). ^1^H NMR (400 MHz, CDCl_3_): δ 7.73–7.53 (m, 7H), 7.50–7.36 (m, 4H), 7.26–7.19 (m, 1H), 6.74–6.68 (m, 1H), 5.00–4.91 (m, 1H), 3.47 (s, 3H), 3.36 (s, 3H), 2.99–2.88 (m, 2H), 2.84–2.74 (m, 1H). ^13^C{^1^H} NMR (100 MHz, CDCl_3_): δ 168.9, 168.6, 145.5, 145.1, 132.9, 128.9, 128.7, 128.3, 127.9, 127.3, 126.4, 125.1, 124.2, 118.4, 118.1, 117.4, 112.8, 111.4, 52.7, 52.6, 49.5, 38.2, 29.7. FT-IR: ν 2953, 2921, 2850, 2376, 2229, 1733, 1653, 1559, 1508, 1233, 1151, 1018, 741, 699 cm^–1^. HRMS (ESI-TOF) *m*/*z*: [M + H]^+^ calcd for C_27_H_24_N_3_O_4_, 454.1761; found 454.1763.

#### Dimethyl 2-(2-Phenyl-2-(2-phenylimidazo[1,2-*a*]pyridin-3-yl)ethyl)malonate (**5j**)

TLC (EtOAC:hexane (2:8)) gave the product as a light yellow oil (190 mg, 88% yield). ^1^H NMR (400 MHz, CDCl_3_): δ 7.71–7.63 (m, 4H), 7.46–7.41 (m, 3H), 7.39–7.33 (m, 5H), 7.21–7.15 (m, 1H), 6.71–6.58 (m, 1H), 5.01–4.87 (m, 1H), 3.45 (s, 3H), 3.36 (s, 3H), 3.03–2.91 (m, 2H), 2.87–2.70 (m, 1H). ^13^C{^1^H} NMR (100 MHz, CDCl_3_): δ 169.1, 168.9, 145.5, 145.2, 139.2, 134.1, 129.2, 129.0, 128.6, 128.1, 127.3, 127.0, 124.8, 118.8, 117.7, 112.3, 52.6, 52.5, 49.7, 37.9, 29.4. (1C signal overlaps). FT-IR: ν 2953, 2916, 2850, 2374, 1750, 1719, 1559, 1436, 1249, 1143, 871, 742, 701 cm^–1^. HRMS (ESI-TOF) *m*/*z*: [M + H]^+^ calcd for C_26_H_25_N_2_O_4_, 429.1809; found 429.1832.

#### Dimethyl 2-(2-(2-Phenylimidazo[1,2-*a*]pyridin-3-yl)-2-(pyren-1-yl)ethyl)malonate (**5k**)

TLC (EtOAC:hexane (2:8)) gave the product as a light yellow oil (271 mg, 97% yield). ^1^H NMR (400 MHz, CDCl_3_): δ 8.46 (d, *J* = 9.3 Hz, 1H), 8.23–8.15 (m, 3H), 8.09–8.00 (m, 5H), 7.92 (d, *J* = 8.0 Hz, 1H), 7.75–7.67 (m, 3H), 7.41–7.37 (m, 3H), 7.24–7.15 (m, 1H), 6.73–6.68 (m, 1H), 5.78 (dd, *J* = 10.1, 6.7 Hz, 1H), 3.63 (s, 3H), 3.50 (s, 3H), 3.36–3.32 (m, 1H), 3.16–2.97 (m, 2H). ^13^C{^1^H} NMR (100 MHz, CDCl_3_): δ 169.6, 169.1, 145.3, 145.2, 135.0, 133.4, 131.3, 130.8, 130.7, 129.6, 128.9, 128.5, 128.4, 128.1, 127.6, 127.3, 126.2, 125.5, 125.4, 124.9, 124.8, 124.5, 124.4, 124.2, 122.4, 119.8, 118.0, 112.6, 52.7, 52.6, 49.5, 36.9, 31.3 (1C signal overlaps). FT-IR: ν 2951, 2917, 2850, 2322, 1750, 1717, 1653, 1559, 1436, 1248, 1143, 846, 742, 701 cm^–1^. HRMS (ESI-TOF) *m*/*z*: [M + H]^+^ calcd for C_36_H_29_N_2_O_4_, 553.2122; found 553.2145.

#### Dimethyl 2-(2-(4-(Diphenylamino)phenyl)-2-(2-phenylimidazo[1,2-*a*]pyridin-3-yl)ethyl)malonate (**5l**)

TLC (EtOAC:hexane (3:7)) gave the product as a light yellow oil (270 mg, 90% yield). ^1^H NMR (400 MHz, CDCl_3_): δ 7.70 (d, *J* = 7.2 Hz, 1H), 7.63–7.59 (m, 3H), 7.36 (t, *J* = 7.4 Hz, 2H), 7.30 (d, *J* = 7.2 Hz, 1H), 7.22–7.07 (m, 7H), 7.04–6.90 (m, 8H), 6.67–6.62 (m, 1H), 4.80 (dd, *J* = 11.3, 5.1 Hz, 1H), 3.38 (s, 3H), 3.28 (s, 3H), 2.93–2.89 (m, 1H), 2.86–2.78 (m, 1H), 2.75–2.66 (m, 1H). ^13^C{^1^H} NMR (100 MHz, CDCl_3_): δ 169.2, 169.0, 147.5, 146.8, 145.7, 145.3, 134.4, 132.8, 129.3, 129.0, 128.5, 128.0, 127.8, 124.9, 124.5, 124.4, 123.8, 123.1, 119.0, 117.8, 112.2, 52.6, 52.5, 49.8, 37.4, 29.5. FT-IR: ν 2953, 2917, 285, 2313, 1735, 1559, 1506, 1270, 1228, 1143, 751, 696 cm^–1^. HRMS (ESI-TOF) *m*/*z*: [M + H]^+^ calcd for C_38_H_34_N_3_O_4_, 596.2544; found 596.2545.

#### Dimethyl 2-(2-(Naphthalen-2-yl)-2-(2-phenylimidazo[1,2-*a*]pyridin-3-yl)ethyl)malonate (**5m**)

TLC (EtOAC:hexane (2:8)) gave the product as a light yellow oil (222 mg, 92% yield). ^1^H NMR (400 MHz, CDCl_3_): δ 7.82 (q, *J* = 7.0 Hz, 4H), 7.75 (d, *J* = 7.1 Hz, 2H), 7.68 (d, *J* = 9.0 Hz, 2H), 7.53–7.34 (m, 6H), 7.20–7.13 (m, 1H), 6.59 (t, *J* = 7.3 Hz, 1H), 5.10 (dd, *J* = 11.5, 4.7 Hz, 1H), 3.47 (s, 3H), 3.38 (s, 3H), 3.17–2.98 (m, 2H), 2.96–2.83 (m, 1H). ^13^C{^1^H} NMR (100 MHz, CDCl_3_): δ 169.2, 168.9, 146.1, 145.5, 136.9, 134.5, 133.5, 132.5, 129.0 (2C), 128.6, 128.0 (2C), 127.6, 126.5, 126.2, 125.5, 125.3, 124.8, 124.6, 118.6, 117.8, 112.2, 52.6, 52.5, 49.8, 38.2, 29.4. FT-IR: ν 2951, 2918, 2850, 2322, 1735, 1559, 1506, 1269, 1225, 1169, 1039, 746, 699 cm^–1^. HRMS (ESI-TOF) *m*/*z*: [M + H]^+^ calcd for C_30_H_27_N_2_O_4_, 479.1965; found 479.1992.

#### Dimethyl 2-(2-(Furan-2-yl)-2-(2-phenylimidazo[1,2-*a*]pyridin-3-yl)ethyl)malonate (**5n**)

TLC (EtOAC:hexane (2:8)) gave the product as a light yellow oil (204 mg, 97% yield). ^1^H NMR (400 MHz, CDCl_3_): δ 7.98 (d, *J* = 7.0 Hz, 1H), 7.70 (d, *J* = 7.0 Hz, 2H), 7.65 (d, *J* = 9.1 Hz, 1H), 7.45 (t, *J* = 7.5 Hz, 2H), 7.38 (d, *J* = 7.0 Hz, 2H), 7.23–7.14 (m, 1H), 6.74 (t, *J* = 6.8 Hz, 1H), 6.37 (dd, *J* = 3.1, 1.9 Hz, 1H), 6.23 (d, *J* = 3.1 Hz, 1H), 4.90 (dd, *J* = 10.6, 5.7 Hz, 1H), 3.44 (s, 3H), 3.37 (s, 3H), 3.03 (t, *J* = 7.3 Hz, 1H), 2.90–2.71 (m, 2H). ^13^C{^1^H} NMR (100 MHz, CDCl_3_): δ 168.9, 168.7, 152.7, 145.1, 142.4, 133.7, 129.2, 128.6, 128.1, 125.0, 124.9, 117.6, 117.1, 112.5, 110.5, 106.9, 52.6, 52.5, 49.4, 33.8, 28.3 (1C signal overlaps). FT-IR: ν 2954, 2923, 2850, 2322, 1749, 1733, 1506, 1230, 1146, 1009, 738, 699 cm^–1^. HRMS (ESI-TOF) *m*/*z*: [M + H]^+^ calcd for C_24_H_23_N_2_O_5_, 419.1601; found 419.1605.

#### Dimethyl 2-(2-(2-Phenylimidazo[1,2-*a*]pyridin-3-yl)-2-(thiophen-2-yl)ethyl)malonate (**5o**)

TLC (EtOAC:hexane (2:8)) gave the product as a light yellow oil (200 mg, 91% yield). ^1^H NMR (400 MHz, CDCl_3_): δ 7.80–7.67 (m, 4H), 7.45 (t, *J* = 7.3 Hz, 2H), 7.39 (d, *J* = 7.2 Hz, 1H), 7.29–7.20 (m, 2H), 7.06–6.99 (m, 1H), 6.98–6.97 (m, 1H), 6.73 (t, *J* = 6.8 Hz, 1H), 5.05 (dd, *J* = 9.9, 5.3 Hz, 1H), 3.43 (s, 3H), 3.34 (s, 3H), 2.99–2.83 (m, 3H). ^13^C{^1^H} NMR (100 MHz, CDCl_3_): δ 168.8, 168.6, 145.3, 143.8, 129.0, 128.7, 128.3, 127.4, 125.2, 125.1, 125.0, 124.4, 118.2, 117.6, 112.5, 52.6, 52.5, 49.6, 35.2, 30.3. FT-IR: ν 2950, 2917, 2851, 2320, 1729, 1559, 1508, 1232, 1168, 1028, 751, 715, 698 cm^–1^ (2C signal overlaps). HRMS (ESI-TOF) *m*/*z*: [M + H]^+^ calcd for C_24_H_23_N_2_O_4_S, 435.1373; found 435.1388.

#### Dimethyl 2-(2-(Furan-2-yl)-2-(2-(*p*-tolyl)imidazo[1,2-*a*]pyridin-3-yl)ethyl)malonate (**7a**)

TLC (EtOAC:hexane (3:7)) gave the product as a light yellow oil (197 mg, 90% yield). ^1^H NMR (400 MHz, CDCl_3_): δ 7.89 (d, *J* = 7.0 Hz, 1H), 7.58 (d, *J* = 9.1 Hz, 1H), 7.51 (d, *J* = 8.0 Hz, 2H), 7.31 (d, *J* = 1.2 Hz, 1H), 7.21–7.16 (m, 2H), 7.15–7.06 (m, 1H), 6.68–6.64 (m, 1H), 6.30 (dd, *J* = 3.2, 1.9 Hz, 1H), 6.15 (d, *J* = 3.3 Hz, 1H), 4.83 (dd, *J* = 10.5, 5.7 Hz, 1H), 3.36 (s, 3H), 3.33 (s, 3H), 2.96 (t, *J* = 7.2 Hz, 1H), 2.82–2.63 (m, 2H), 2.32 (s, 3H). ^13^C{^1^H} NMR (100 MHz, CDCl_3_): δ 169.0, 168.7, 152.9, 145.7, 145.3, 142.2, 137.7, 131.2, 129.3, 129.1, 124.9, 124.4, 117.7, 116.8, 112.1, 110.5, 106.8, 52.6, 52.5, 49.5, 33.9, 28.3, 21.3. FT-IR: ν 2954, 2920, 2848, 2310, 1749, 1733, 1559, 1506, 1226, 1143, 1011, 826, 735 cm^–1^. HRMS (ESI-TOF) *m*/*z*: [M + H]^+^ calcd for C_25_H_25_N_2_O_5_, 433.1758; found 433.1768.

#### Dimethyl 2-(2-(Pyren-1-yl)-2-(2-(*p*-tolyl)imidazo[1,2-*a*]pyridin-3-yl)ethyl)malonate (**7b**)

TLC (EtOAC:hexane (3:7)) gave the product as a light yellow oil (255 mg, 89% yield). ^1^H NMR (400 MHz, CDCl_3_): δ 8.46 (d, *J* = 9.2 Hz, 1H), 8.18 (dd, *J* = 16.3, 8.3 Hz, 3H), 8.09–7.98 (m, 5H), 7.93 (d, *J* = 8.0 Hz, 1H), 7.71 (d, *J* = 9.2 Hz, 1H), 7.60 (d, *J* = 8.0 Hz, 2H), 7.22–7.13 (m, 3H), 6.72–6.62 (m, 1H), 5.78 (dd, *J* = 10.2, 6.6 Hz, 1H), 3.63 (s, 3H), 3.50 (s, 3H), 3.35 (dd, *J* = 8.7, 6.0 Hz, 1H), 3.16–2.99 (m, 2H), 2.39 (s, 3H). ^13^C{^1^H} NMR (100 MHz, CDCl_3_): δ 169.6, 169.1, 145.3, 145.2, 137.8, 133.5, 132.0, 131.4, 130.8, 130.7, 129.4, 129.1, 128.9, 128.5, 127.5, 127.3, 126.2, 125.52, 125.49, 125.4, 124.9, 124.8, 124.6, 124.3, 124.1, 122.5, 119.6, 117.9, 112.5, 52.7, 52.5, 49.5, 37.0, 31.3, 21.3. FT-IR: ν 2951, 2928, 2846, 2322, 1747, 1733, 1559, 1508, 1226, 1148, 846, 719 cm^–1^. HRMS (ESI-TOF) *m*/*z*: [M + H]^+^ calcd for C_37_H_31_N_2_O_4_, 567.2278; found 567.2293.

#### Dimethyl 2-(2-(2-(4-Chlorophenyl)imidazo[1,2-*a*]pyridin-3-yl)-2-(furan-2-yl)ethyl)malonate (**7c**)

TLC (EtOAC:hexane (2:8)) gave the product as a light yellow oil (205 mg, 90% yield). ^1^H NMR (400 MHz, CDCl_3_): δ 7.99 (d, *J* = 7.0 Hz, 1H), 7.70 (d, *J* = 9.1 Hz, 1H), 7.64 (d, *J* = 8.4 Hz, 2H), 7.43 (d, *J* = 8.4 Hz, 2H), 7.38 (d, *J* = 1.3 Hz, 1H), 7.25–7.22 (m, 1H), 6.79 (t, *J* = 6.8 Hz, 1H), 6.37 (dd, *J* = 3.2, 1.9 Hz, 1H), 6.25 (d, *J* = 3.1 Hz, 1H), 4.83 (dd, *J* = 10.4, 5.8 Hz, 1H), 3.45 (s, 3H), 3.43 (s, 3H), 3.02 (t, *J* = 7.2 Hz, 1H), 2.88–2.70 (m, 2H). ^13^C{^1^H} NMR (100 MHz, CDCl_3_): δ 168.8, 168.6, 152.3, 145.0, 143.6, 142.5, 134.3, 132.1, 130.5, 128.9, 125.4, 124.9, 117.6, 117.4, 112.8, 110.5, 107.0, 52.7, 52.6, 49.4, 33.8, 28.3. FT-IR: ν 2954, 2312, 1749, 1733, 1559, 1506, 1223, 1143, 1089, 1013, 909, 836, 726 cm^–1^. HRMS (ESI-TOF) *m*/*z*: [M + H]^+^ calcd for C_24_H_22_ClN_2_O_5_, 453.1212; found 453.1229.

#### Dimethyl 2-(2-(2-(4-Chlorophenyl)imidazo[1,2-*a*]pyridin-3-yl)-2-(pyren-1-yl)ethyl)malonate (**7d**)

TLC (EtOAC:hexane (2:8)) gave the product as a light yellow oil (279 mg, 95% yield). ^1^H NMR (400 MHz, CDCl_3_): δ 8.28 (d, *J* = 9.1 Hz, 1H), 8.13–8.03 (m, 4H), 8.00–7.89 (m, 4H), 7.78 (d, *J* = 8.2 Hz, 1H), 7.69 (d, *J* = 9.1 Hz, 1H), 7.48 (d, *J* = 8.3 Hz, 2H), 7.24 (d, *J* = 8.2 Hz, 2H), 7.17 (t, *J* = 7.7 Hz, 1H), 6.69 (t, *J* = 6.7 Hz, 1H), 5.65 (dd, *J* = 9.5, 7.0 Hz, 1H), 3.58 (s, 3H), 3.44 (s, 3H), 3.28–3.22 (m, 1H), 3.06–2.87 (m, 2H). ^13^C{^1^H} NMR (100 MHz, CDCl_3_): δ 169.5, 169.1, 144.7, 142.9, 134.4, 132.6, 132.4, 131.3, 131.0, 130.9, 130.6, 129.0, 128.7, 128.6, 127.7, 127.3, 126.3, 125.7, 125.5, 124.9, 124.7, 124.33, 124.26, 122.1, 120.4, 117.6, 113.3, 52.8, 52.7, 49.3, 36.7, 31.2 (2C signal overlaps). FT-IR: ν 2953, 2921, 2850, 2322, 1733, 1653, 1559, 1508, 1225, 1151, 1013, 842, 718 cm^–1^. HRMS (ESI-TOF) *m*/*z*: [M + H]^+^ calcd for C_36_H_28_ClN_2_O_4_, 587.1732; found 587.1756.

#### Dimethyl 2-(2-(2-(4-Bromophenyl)imidazo[1,2-*a*]pyridin-3-yl)-2-(furan-2-yl)ethyl)malonate (**7e**)

TLC (EtOAC:hexane (3:7)) gave the product as a light yellow oil (235 mg, 94% yield). ^1^H NMR (400 MHz, CDCl_3_): δ 7.97 (d, *J* = 6.9 Hz, 1H), 7.67 (d, *J* = 9.1 Hz, 1H), 7.57 (s, 4H), 7.38 (s, 1H), 7.25–7.16 (m, 1H), 6.78 (t, *J* = 6.7 Hz, 1H), 6.38–6.32 (m, 1H), 6.24 (d, *J* = 3.0 Hz, 1H), 4.82 (dd, *J* = 10.4, 5.8 Hz, 1H), 3.45 (s, 3H), 3.43 (s, 3H), 3.01 (t, *J* = 7.2 Hz, 1H), 2.91–2.63 (m, 2H). ^13^C{^1^H} NMR (100 MHz, CDCl_3_): δ 168.8, 168.6, 152.2, 144.8, 143.2, 142.5, 132.1, 131.9, 130.8, 125.7, 125.0, 122.7, 117.5, 117.4, 113.1, 110.6, 107.1, 52.69, 52.67, 49.4, 33.7, 28.3. FT-IR: ν 2954, 2314, 1733, 1506, 1232, 1143, 1009, 908, 833, 726 cm^–1^. HRMS (ESI-TOF) *m*/*z*: [M + H]^+^ calcd for C_24_H_22_BrN_2_O_5_, 497.0707; found 497.0707.

#### Dimethyl 2-(2-(2-(4-Bromophenyl)imidazo[1,2-*a*]pyridin-3-yl)-2-(pyren-1-yl)ethyl)malonate (**7f**)

TLC (EtOAC:hexane (3:7)) gave the product as a light yellow oil (290 mg, 92% yield). ^1^H NMR (400 MHz, CDCl_3_): δ 8.34 (d, *J* = 9.4 Hz, 1H), 8.22–8.19 (m, 2H), 8.15 (d, *J* = 9.0 Hz, 2H), 8.09–7.97 (m, 4H), 7.87 (d, *J* = 8.0 Hz, 1H), 7.81 (d, *J* = 9.0 Hz, 1H), 7.49 (q, *J* = 8.6 Hz, 4H), 7.29 (d, *J* = 7.2 Hz, 1H), 6.80 (t, *J* = 6.8 Hz, 1H), 5.77–5.70 (m, 1H), 3.68 (s, 3H), 3.54 (s, 3H), 3.41–3.30 (m, 1H), 3.15–2.95 (m, 2H). ^13^C{^1^H} NMR (100 MHz, CDCl_3_): δ 169.4, 169.1, 144.4, 142.4, 132.6, 132.2, 131.6, 131.3, 131.1, 131.0, 130.6, 129.0, 128.8, 127.8, 127.3, 126.3, 125.9, 125.7, 125.53, 125.51, 124.9, 124.7, 124.33, 124.30, 122.9, 122.0, 120.6, 117.4, 113.6, 52.84, 52.76, 49.3, 36.7, 31.2. FT-IR: ν 2953, 2926, 2847, 2376, 1733, 1559, 1436, 1263, 1225, 1148, 1009, 843, 731 cm^–1^. HRMS (ESI-TOF) *m*/*z*: [M + H]^+^ calcd for C_36_H_28_BrN_2_O_4_, 631.1227; found 631.1243.

#### Dimethyl 2-(2-(Furan-2-yl)-2-(2-(4-methoxyphenyl)imidazo[1,2-*a*]pyridin-3-yl)ethyl)malonate (**7g**)

TLC (EtOAC:hexane (3:7)) gave the product as a light yellow oil (208 mg, 92% yield). ^1^H NMR (400 MHz, CDCl_3_): δ 7.98 (d, *J* = 6.9 Hz, 1H), 7.71 (d, *J* = 9.0 Hz, 1H), 7.63 (d, *J* = 8.6 Hz, 2H), 7.38 (s, 1H), 7.26–7.18 (m, 1H), 6.99 (d, *J* = 8.6 Hz, 2H), 6.78 (t, *J* = 6.7 Hz, 1H), 6.37 (dd, *J* = 3.1, 1.9 Hz, 1H), 6.23 (d, *J* = 3.2 Hz, 1H), 4.87 (dd, *J* = 10.4, 5.8 Hz, 1H), 3.84 (s, 3H), 3.44 (s, 3H), 3.42 (s, 3H), 3.03 (t, *J* = 7.2 Hz, 1H), 2.88–2.71 (m, 2H). ^13^C{^1^H} NMR (100 MHz, CDCl_3_): δ 168.9, 168.7, 159.8, 152.5, 142.4, 130.5, 125.5, 124.9, 117.1, 116.8, 114.2, 112.9, 110.5, 107.0, 55.3, 52.7, 52.6, 49.5, 33.8, 28.2 (3C signal overlaps). FT-IR: ν 2954, 2920, 2848, 2310, 1733, 1653, 1559, 1508, 1248, 1173, 1025, 836, 736 cm^–1^. HRMS (ESI-TOF) *m*/*z*: [M + H]^+^ calcd for C_25_H_25_N_2_O_6_, 449.1707; found 449.1737.

#### Dimethyl 2-(2-(2-(4-Methoxyphenyl)imidazo[1,2-*a*]pyridin-3-yl)-2-(pyren-1-yl)ethyl)malonate (**7h**)

TLC (EtOAC:hexane (3:7)) gave the product as a light yellow oil (270 mg, 92% yield). ^1^H NMR (400 MHz, CDCl_3_): δ 8.44 (d, *J* = 9.1 Hz, 1H), 8.23–8.13 (m, 3H), 8.11–7.97 (m, 5H), 7.91 (d, *J* = 8.0 Hz, 1H), 7.73 (d, *J* = 9.1 Hz, 1H), 7.61 (d, *J* = 8.4 Hz, 2H), 7.23–7.15 (m, 1H), 6.91 (d, *J* = 8.4 Hz, 2H), 6.71 (t, *J* = 6.8 Hz, 1H), 5.75 (dd, *J* = 9.9, 6.7 Hz, 1H), 3.81 (s, 3H), 3.65 (s, 3H), 3.51 (s, 3H), 3.35 (dd, *J* = 8.4, 6.1 Hz, 1H), 3.18–2.98 (m, 2H). ^13^C{^1^H} NMR (100 MHz, CDCl_3_): δ 169.6, 169.1, 159.7, 144.9, 144.6, 133.3, 131.3, 130.83, 130.78, 130.7, 128.9, 128.5, 127.6, 127.3, 126.9, 126.2, 125.5, 125.4, 124.9, 124.8, 124.6, 124.5, 124.1, 122.4, 119.5, 117.6, 113.8, 112.7, 55.3, 52.7, 52.5, 49.5, 36.9, 31.3 (1C signal overlaps). FT-IR: ν 2956, 2926, 2841, 2320, 1733, 1559, 1508, 1246, 1173, 1032, 842, 729 cm^–1^. HRMS (ESI-TOF) *m*/*z*: [M + 2H]^+^ calcd for C_37_H_32_N_2_O_5_, 584.2306; found 584.2331.

#### Dimethyl 2-(2-(4-Methoxyphenyl)-2-(2-(4-(methylsulfonyl)phenyl)imidazo[1,2-*a*]pyridin-3-yl)ethyl)malonate (**7i**)

TLC (EtOAC:hexane (3:7)) gave the product as a yellow solid (256 mg, 95% yield; mp 78.1–79.1 °C). ^1^H NMR (400 MHz, CDCl_3_) δ 7.93 (d, *J* = 8.4 Hz, 2H), 7.81 (d, *J* = 8.4 Hz, 2H), 7.67 (d, *J* = 7.0 Hz, 1H), 7.63–7.52 (m, 1H), 7.25–7.07 (m, 3H), 6.84–6.77 (m, 2H), 6.65 (td, *J* = 7.0, 1.1 Hz, 1H), 4.75 (dd, *J* = 10.8, 5.2 Hz, 1H), 3.72 (s, 3H), 3.38 (s, 3H), 3.35 (s, 3H), 3.01 (s, 3H), 2.95–2.80 (m, 2H), 2.77–2.64 (m, 1H). ^13^C{^1^H} NMR (100 MHz, CDCl_3_) δ 169.0, 168.7, 158.8, 145.6, 143.3, 140.3, 139.7, 130.3, 129.9, 128.0, 127.6, 125.3, 125.0, 120.5, 117.9, 114.6, 112.7, 55.3, 52.6, 49.8, 44.6, 37.4, 29.6. FT-IR: ν 2954, 2924, 2851, 2313, 1733, 1559, 1508, 1308, 1248, 1143, 1032, 956, 772 cm^–1^. HRMS (ESI-TOF) *m*/*z*: [M + 2H]^+^ calcd for C_28_H_30_N_2_O_7_S, 538.1768; found 538.1770.

#### Dimethyl 2-(2-(2-(4-(Methylsulfonyl)phenyl)imidazo[1,2-*a*]pyridin-3-yl)-2-phenylethyl)malonate (**7j**)

TLC (EtOAC:hexane (2:8)) gave the product as a yellow solid (240 mg, 94% yield; mp 79.2–80.2 °C). ^1^H NMR (400 MHz, CDCl_3_) δ 7.94 (d, *J* = 8.4 Hz, 2H), 7.83 (d, *J* = 8.4 Hz, 2H), 7.65 (d, *J* = 7.0 Hz, 1H), 7.61 (d, *J* = 9.1 Hz, 1H), 7.37–7.07 (m, 6H), 6.65 (td, *J* = 7.0, 1.2 Hz, 1H), 4.83 (dd, *J* = 11.0, 4.7 Hz, 1H), 3.39 (s, 3H), 3.36 (s, 3H), 3.02 (s, 3H), 2.98–2.86 (m, 2H), 2.78–2.67 (m, 1H).^13^C{^1^H} NMR (100 MHz, CDCl_3_) δ 169.0, 168.7, 145.6, 143.5, 140.2, 139.7, 138.5, 129.9, 129.3, 127.6, 127.5, 127.0, 125.4, 124.9, 120.2, 118.0, 112.8, 52.7 (2C), 49.8, 44.6, 38.1 29.4. FT-IR: ν 2928, 2923, 2322, 1723, 1559, 1508, 1299, 1236, 1148, 1033, 972, 852, 748, 776 cm^–1^. HRMS (ESI-TOF) *m*/*z*: [M + 2H]^+^ calcd for C_27_H_28_N_2_O_6_S, 508.1663; found 508.1681.

#### Dimethyl 2-(2-(2-(4-(Methylsulfonyl)phenyl)imidazo[1,2-*a*]pyridin-3-yl)-2-(pyren-4-yl)ethyl)malonate (**7k**)

TLC (EtOAC:hexane (2:8)) gave the product as a light yellow solid (294 mg, 93% yield; mp 131.2–132.2 °C). ^1^H NMR (400 MHz, CDCl_3_) δ 8.18 (d, *J* = 6.8 Hz, 1H), 8.12 (d, *J* = 9.3 Hz, 1H), 8.08 (d, *J* = 7.5 Hz, 2H), 8.01 (d, *J* = 9.3 Hz, 1H), 7.99–7.81 (m, 4H), 7.76 (d, *J* = 8.0 Hz, 1H), 7.70 (d, *J* = 7.5 Hz, 2H), 7.65–7.45 (m, 3H), 7.16 (t, *J* = 7.8 Hz, 1H), 6.73 (t, *J* = 6.7 Hz, 1H), 5.64 (t, *J* = 8.1 Hz, 1H), 3.57 (s, 3H), 3.44 (s, 3H), 3.29 (t, *J* = 7.2 Hz, 1H), 3.14–2.70 (m, 5H). ^13^C{^1^H} NMR (100 MHz, CDCl_3_) δ 169.4, 169.1, 145.2, 142.8, 140.5, 139.6, 131.8, 131.3, 130.9, 130.5, 130.3, 129.1, 128.8, 127.8, 127.2, 127.1, 126.4, 125.8, 125.6, 125.5, 125.3 (2C), 124.8, 124.6, 124.3, 121.9, 121.5, 118.0, 113.4, 52.9, 52.8, 49.3, 44.5, 36.6, 31.1. FT-IR: ν 2951, 2924, 2322, 1733, 1653, 1559, 1508, 1312, 1151, 955, 843, 719, 541 cm^–1^. HRMS (ESI-TOF) *m*/*z*: [M + 2H]^+^ calcd for C_37_H_32_N_2_O_6_S, 632.1976; found 632.1985.

#### Dimethyl 2-(2-(Furan-2-yl)-2-(2-(4-(methylsulfonyl)phenyl)imidazo[1,2-*a*]pyridin-3-yl)ethyl)malonate (**7l**)

TLC (EtOAC:hexane (2:8)) gave the product as a light yellow solid (228 mg, 91% yield; mp 75.2–76.2 °C). ^1^H NMR (400 MHz, CDCl_3_) δ 8.09–7.99 (m, 1H), 7.96 (d, *J* = 8.3 Hz, 2H), 7.85 (d, *J* = 8.3 Hz, 2H), 7.62 (d, *J* = 9.1 Hz, 1H), 7.32 (d, *J* = 1.6 Hz, 1H), 7.20–7.12 (m, 1H), 6.75 (td, *J* = 6.9, 1.1 Hz, 1H), 6.31 (dd, *J* = 3.2, 1.9 Hz, 1H), 6.20 (d, *J* = 3.2 Hz, 1H), 4.78 (dd, *J* = 9.8, 6.2 Hz, 1H), 3.39 (s, 3H), 3.38 (s, 3H), 3.03 (s, 3H), 2.98 (t, *J* = 7.2 Hz, 1H), 2.86–2.63 (m, 2H). ^13^C{^1^H} NMR (100 MHz, CDCl_3_) δ 168.8, 168.6, 152.0, 145.6, 143.0, 142.6, 139.8, 139.7, 130.1, 127.6, 125.5, 125.0, 118.4, 117.9, 113.0, 110.6, 107.2, 52.7 (2C), 49.5, 44.5, 33.9, 28.4. FT-IR: ν 2954, 2926, 2850, 2377, 1733, 1559, 15,008, 1308, 1143, 1013, 956, 772, 736, 538 cm^–1^. HRMS (ESI-TOF) *m*/*z*: [M + 2H]^+^ calcd for C_25_H_26_N_2_O_7_S, 498.1455; found 498.1457.

#### 1-(*tert*-Butyl) 1,1-Dimethyl 3-(imidazo[1,2-*a*]pyridin-3-yl)-3-(4-methoxyphenyl)propane-1,1,1-tricarboxylate (**8**)

A solution of imidazo[1,2-*a*]pyridine (**1**) (62 mg, 0.52 mmol), dimethyl 2-(4-methoxyphenyl)cyclopropane-1,1-dicarboxylate (**2a**) (139 mg, 0.52 mmol), and Yb(OTf)_3_ (25 mol %) in CH_3_CN (3 mL) was charged into a sealed tube and heated to 100 °C in an oil bath for 24 h. The reaction mixture was cooled to room temperature and then the solvent was evaporated, the reaction mixture was diluted with EtOAC, filtered through a short pad of silica gel, and eluted with DCM:MeOH. The solvent was evaporated, and the crude product was dissolved in DCM (7 mL), followed by the addition of di-*tert* butyl dicarbonate (171 mg, 0.78 mmol), triethylamine (110 μL, 0.78 mmol), and 4-dimethylaminopyridine (9.6 mg, 0.07 mmol) at room temperature. The mixture was allowed to stir at room temperature for 3 h, the crude compound was purified by TLC (DCM:MeOH (9.7:0.3)), affording the desired compound **8** as light yellow oil (228 mg, 90% yield). ^1^H NMR (400 MHz, CDCl_3_): δ 9.09 (d, *J* = 6.7 Hz, 1H), 8.59 (d, *J* = 2.3 Hz, 1H), 7.98–7.88 (m, 2H), 7.45–7.40 (m, 1H), 7.36 (t, *J* = 5.8 Hz, 2H), 6.91 (d, *J* = 8.8 Hz, 2H), 6.52 (t, *J* = 6.7 Hz, 1H), 3.78 (s, 3H), 3.55 (s, 3H), 3.53 (s, 3H), 3.27–3.21 (m, 2H), 1.39 (s, 9H). ^13^C{^1^H} NMR (100 MHz, CDCl_3_): δ 166.8, 164.7, 160.5, 138.4, 134.3, 131.0, 128.6, 128.0, 123.2, 117.9, 114.9, 110.9, 84.9, 63.9, 57.4, 55.5, 53.4, 36.6, 27.5 (3C signal overlaps). FT-IR: ν 2958, 2322, 1735, 1653, 1559, 1516, 1255, 1223, 1151, 1029, 836, 732, 638 cm^–1^. HRMS (ESI-TOF) *m*/*z*: [M + H]^+^ calcd for C_26_H_31_N_2_O_7_, 483.2126; found 483.2136.

#### Dimethyl 2-(2-(Imidazo[1,2-*a*]pyridin-3-yl)-2-(4-methoxyphenyl)ethyl)-2-methylmalonate (**9**)

A solution of imidazo[1,2-*a*]pyridine (**1**) (62 mg, 0.52 mmol), dimethyl 2-(4-methoxyphenyl)cyclopropane-1,1-dicarboxylate (**2a**) (139 mg, 0.52 mmol), and Yb(OTf)_3_ (25 mol %) in CH_3_CN (3.0 mL) was charged into a sealed tube and heated to 100 °C in an oil bath for 24 h. The reaction mixture was cooled to room temperature and then the solvent was evaporated, the reaction mixture was diluted with EtOAC, filtered through a short pad of silica gel, and eluted with DCM:MeOH. The solvent was evaporated, and the crude product was dissolved in THF (7 mL), followed by the addition of potassium *tert*-butoxide (65 mg, 0.57 mmol) and methyl iodide (49 μL, 0.78 mmol) at room temperature, respectively. After stirring at room temperature for 3 h, solvent was evaporated on reduced pressure. The crude compound was purified by thin layer chromatography (DCM:MeOH (9.6:0.4)), affording the desired compound **9** as orange oil (177 mg, 85% yield). ^1^H NMR (400 MHz, CDCl_3_): δ 9.18 (d, *J* = 6.5 Hz, 1H), 8.66 (s, 1H), 8.25 (d, *J* = 9.2 Hz, 1H), 8.02–7.88 (m, 1H), 7.45–7.40 (m, 3H), 6.91 (d, *J* = 8.7 Hz, 2H), 6.25–6.08 (m, 1H), 3.77 (s, 3H), 3.55 (s, 3H), 3.38 (s, 3H), 3.04–2.81 (m, 2H), 1.51 (s, 3H). ^13^C{^1^H} NMR (100 MHz, CDCl_3_): δ 172.0, 171.3, 160.5, 138.3, 134.5, 130.6, 129.1, 127.4, 122.7, 117.9, 116.5, 114.9, 111.3, 58.5, 55.5, 53.0, 52.8, 52.2, 40.0, 21.9 (1C signal overlaps). FT-IR: ν 2954, 2917, 2850, 2310, 1733, 1516, 1252, 1156, 1109, 1029, 835, 755, 636 cm^–1^. HRMS (ESI-TOF) *m*/*z*: [M + H]^+^ calcd for C_22_H_25_N_2_O_5_, 397.1758; found 397.1776.

#### Dimethyl 2-Bromo-2-(2-(2,5-dimethoxyphenyl)-2-(imidazo[1,2-*a*]pyridin-3-yl)ethyl)malonate (**10**)

A solution of imidazo[1,2-*a*]pyridine (**1**) (57 mg, 0.48 mmol), dimethyl 2-(2,5-dimethoxyphenyl)cyclopropane-1,1-dicarboxylate (**2b**) (142 mg, 0.48 mmol), and Yb(OTf)_3_ (25 mol %) in CH_3_CN (3.0 mL) was charged into a sealed tube and heated to 100 °C in an oil bath for 24 h. The reaction mixture was cooled to room temperature, and then the solvent was evaporated; the reaction mixture was diluted with EtOAC, filtered through a short pad of silica gel, and eluted with DCM:MeOH. The solvent was evaporated, and the crude product was dissolved in THF (8 mL), followed by the addition of potassium *tert*-butoxide (60 mg, 0.53 mmol) and NBS (129 mg, 0.72 mmol) at room temperature, respectively. After stirring at room temperature for 3 h, the solvent was evaporated on reduced pressure. The crude compound was purified by thin layer chromatography (hexane:acetone (4:6)), affording the desired compound **10** as an orange oil (215 mg, 90% yield). ^1^H NMR (400 MHz, CDCl_3_): δ 9.07 (d, *J* = 6.8 Hz, 1H), 8.61 (d, *J* = 2.1 Hz, 1H), 8.04 (d, *J* = 9.2 Hz, 1H), 8.01–7.88 (m, 2H), 7.45 (t, *J* = 6.8 Hz, 1H), 7.14 (d, *J* = 2.8 Hz, 1H), 6.92–6.79 (m, 2H), 6.49 (dd, *J* = 8.4, 5.0 Hz, 1H), 3.83 (s, 3H), 3.72 (s, 3H), 3.68 (s, 3H), 3.50 (s, 3H), 3.44–3.31 (m, 2H). ^13^C{^1^H} NMR (100 MHz, CDCl_3_): δ 166.4, 166.0, 154.3, 150.9, 139.2, 134.3, 131.1, 124.4, 123.2, 118.1, 117.4, 114.4, 114.2, 112.6, 111.4, 60.3, 56.4, 56.2, 54.8, 53.5, 40.7, 29.9. FT-IR: ν 2957, 2917, 2850, 2323, 1735, 1506, 1222, 1223, 1155, 1028, 756, 636 cm^–1^. HRMS (ESI-TOF) *m*/*z*: [M + H]^+^ calcd for C_22_H_24_BrN_2_O_6_, 491.0812; found 491.0840.

#### Dimethyl 2-Chloro-2-(2-(imidazo[1,2-*a*]pyridin-3-yl)-2-(3,4,5-trimethoxyphenyl)ethyl)malonate (**11**)

A solution of imidazo[1,2-*a*]pyridine (**1**) (53 mg, 0.44 mmol), dimethyl 2-(3,4,5-trimethoxyphenyl)cyclopropane-1,1-dicarboxylate (**2c**) (145 mg, 0.44 mmol), and Yb(OTf)_3_ (25 mol %) in CH_3_CN (3.0 mL) was charged into a sealed tube and heated to 100 °C in an oil bath for 24 h. The reaction mixture was cooled to room temperature, and then the solvent was evaporated; the reaction mixture was diluted with EtOAC, filtered through a short pad of silica gel, and eluted with DCM:MeOH. The solvent was evaporated, and the crude product was dissolved in THF (8 mL), followed by the addition of potassium *tert*-butoxide (56 mg, 0.5 mmol) and NCS (90 mg, 0.68 mmol) at room temperature, respectively. After stirring at room temperature for 3 h, solvent was evaporated on reduced pressure. The crude compound was purified by thin layer chromatography (hexane:acetone (4:6)), affording the desired compound **11** as an orange oil (173 mg, 80% yield). ^1^H NMR (400 MHz, CDCl_3_): δ 8.97 (d, *J* = 6.7 Hz, 1H), 8.47 (d, *J* = 2.2 Hz, 1H), 8.37 (d, *J* = 2.2 Hz, 1H), 8.06 (d, *J* = 9.2 Hz, 1H), 8.03–7.89 (m, 1H), 7.45 (t, *J* = 7.0 Hz, 1H), 6.72 (s, 1H), 6.06 (t, *J* = 6.8 Hz, 1H), 3.89 (s, 6H), 3.80 (s, 3H), 3.65 (s, 3H), 3.61 (s, 3H), 3.55–3.41 (m, 2H). ^13^C{^1^H} NMR (100 MHz, CDCl_3_): δ 166.0, 165.9, 154.0, 139.2, 138.5, 134.6, 130.72, 130.65, 123.4, 118.0, 116.8, 110.7, 104.8, 68.0, 60.8, 58.4, 56.6, 54.4, 54.3, 40.8. FT-IR: ν 2956, 2913, 2848, 2353, 1749, 1508, 1250, 1155, 1123, 1029, 1003, 756, 636 cm^–1^. HRMS (ESI-TOF) *m*/*z*: [M + H]^+^ calcd for C_23_H_26_ClN_2_O_7_, 477.1423; found 477.1445.

#### Dimethyl 2-Methyl-2-(2-(2-phenylimidazo[1,2-*a*]pyridin-3-yl)-2-(3,4,5 trimethoxyphenyl)ethyl)malonate (**12**)

A solution of 2-phenylimidazo[1,2-*a*]pyridine (**4**) (60 mg, 0.3 mmol), dimethyl 2-(3,4,5-trimethoxyphenyl)cyclopropane-1,1-dicarboxylate (**2c**) (100 mg, 0.3 mmol), and Gd(OTf)_3_ (15 mol %) in DCE (3.0 mL) was charged into a sealed tube and heated to 100 °C in an oil bath for 24 h. The reaction mixture was cooled to room temperature, and then the solvent was evaporated; the reaction mixture was diluted with DCM, filtered through a short pad of silica gel, and eluted with EtOAC:hexane. The solvent was evaporated, and the crude product was dissolved in THF (6.0 mL), followed by the addition of potassium *tert*-butoxide (38 mg, 0.34 mmol) and methyl iodide (29 μL, 0.46 mmol) at room temperature, respectively. After stirring at room temperature for 3 h, solvent was evaporated on reduced pressure. The crude compound was purified by thin layer chromatography (EtOAC:hexane (2:8)), affording the desired compound **12** as an yellow oil (145 mg, 88% yield). ^1^H NMR (400 MHz, CDCl_3_) δ 7.77 (d, *J* = 6.8 Hz, 1H), 7.72–7.58 (m, 3H), 7.45 (t, *J* = 7.3 Hz, 4H), 7.38 (t, *J* = 7.3 Hz, 2H), 7.24–7.14 (m, 2H), 6.73 (t, *J* = 6.8 Hz, 1H), 6.54 (s, 2H), 5.05–4.71 (m, 1H), 3.84 (s, 3H), 3.73 (s, 6H), 3.45 (s, 3H), 3.12 (s, 3H), 3.08–2.94 (m, 1H), 2.86–2.68 (m, 1H). ^13^C{^1^H} NMR (100 MHz, CDCl_3_) δ 172.8, 171.1, 153.8, 145.6, 145.4, 137.3, 136.1, 134.8, 129.1, 128.9, 128.4, 125.5, 124.8, 119.5, 117.8, 112.2, 104.4, 61.1, 56.5, 53.3, 52.9, 52.5, 35.9, 35.6, 19.4. FT-IR: *ν* 2953, 2838, 2371, 1733, 1508, 1458, 1239, 1125, 1111, 1006, 734, 699 cm^–1^. HRMS (ESI-TOF) *m*/*z*: [M + H]^+^ calcd for C_30_H_33_N_2_O_7_, 533.2282; found 533.2281.

## Data Availability

The data underlying this study are available in the published article and its Supporting Information.

## Supporting Information Available

NMR spectra for all compounds (PDF)

## References

[ref1] Anaflous A.; Benchat N.; Mimouni M.; Abouricha S.; Ben-Hadda T.; El-Bali B.; Hakkou A.; Hacht B. Armed Imidazo [1,2-*a*] Pyrimidines (Pyridines): Evaluation of Antibacterial Activity. Lett. Drug Des. Discovery 2004, 1, 224–229. 10.2174/1570180043398885.

[ref2] Al-Tel T. H.; Al-Qawasmeh R. A. Post Groebke–Blackburn Multicomponent Protocol: Synthesis of New Polyfunctional Imidazo [1,2-a] Pyridine and Imidazo [1,2-*a*] Pyrimidine Derivatives as Potential Antimicrobial Agents. Eur. J. Med. Chem. 2010, 45, 5848–5855. 10.1016/j.ejmech.2010.09.049. 20934788

[ref3] Kurteva V. Recent Progress in Metal-Free Direct Synthesis of Imidazo [1,2-*a*] Pyridines. ACS Omega 2021, 6, 35173–35185. 10.1021/acsomega.1c03476. 34984250 PMC8717391

[ref4] Nguyen T. B.; Ermolenko L.; Retailleau P.; Al-Mourabit A. Molecular Iodine-Catalyzed Aerobic α, β-Diamination of Cyclohexanones with 2-Aminopyrimidine and 2-Aminopyridines. Org. Lett. 2016, 18, 2177–2179. 10.1021/acs.orglett.6b00823. 27088653

[ref5] Baviskar A. T.; Amrutkar S. M.; Trivedi N.; Chaudhary V.; Nayak A.; Guchhait S. K.; Banerjee U. C.; Bharatam P. V.; Kundu C. N. Switch in Site of Inhibition: A Strategy for Structure-Based Discovery of Human Topoisomerase IIα Catalytic Inhibitors. ACS Med. Chem. Lett. 2015, 6, 481–485. 10.1021/acsmedchemlett.5b00040. 25941559 PMC4416441

[ref6] Almirante L.; Polo L.; Mugnaini A.; Provinciali E.; Rugarli P.; Biancotti A.; Gamba A.; Murmann W. Derivatives of Imidazole. I. Synthesis and Reactions of Imidazo [1,2-α] Pyridines with Analgesic, Antiinflammatory, Antipyretic, and Anticonvulsant Activity. J. Med. Chem. 1965, 8, 305–312. 10.1021/jm00327a007. 14329509

[ref7] Gudmundsson K. S.; Williams J. D.; Drach J. C.; Townsend L. B. Synthesis and Antiviral Activity of Novel Erythrofuranosyl Imidazo [1,2-*a*] Pyridine C-Nucleosides Constructed via Palladium Coupling of Iodoimidazo [1,2-*a*] Pyridines and Dihydrofuran. J. Med. Chem. 2003, 46, 1449–1455. 10.1021/jm020339r. 12672244

[ref8] Hranjec M.; Kralj M.; Piantanida I.; Sedić M.; Šuman L.; Pavelić K.; Karminski-Zamola G. Novel Cyano-and Amidino-Substituted Derivatives of Styryl-2-Benzimidazoles and Benzimidazo [1,2-a] Quinolines. Synthesis, Photochemical Synthesis, DNA Binding, and Antitumor Evaluation, Part 3. J. Med. Chem. 2007, 50, 5696–5711. 10.1021/jm070876h. 17935309

[ref9] Lacerda R. B.; de Lima C. K. F.; da Silva L. L.; Romeiro N. C.; Miranda A. L. P.; Barreiro E. J.; Fraga C. A. M. Discovery of Novel Analgesic and Anti-Inflammatory 3-Arylamine-Imidazo [1,2-a] Pyridine Symbiotic Prototypes. Bioorg. Med. Chem. 2009, 17, 74–84. 10.1016/j.bmc.2008.11.018. 19059783

[ref10] Messineo L.; Eckert D. J.; Lim R.; Chiang A.; Azarbarzin A.; Carter S. G.; Carberry J. C. Zolpidem Increases Sleep Efficiency and the Respiratory Arousal Threshold without Changing Sleep Apnoea Severity and Pharyngeal Muscle Activity. J. Physiol. 2020, 598, 4681–4692. 10.1113/JP280173. 32864734

[ref11] Zivkovic B.; Morel E.; Joly D.; Perrault G.; Sanger D. J.; Lloyd K. G. Pharmacological and Behavioral Profile of Alpidem as an Anxiolytic. Pharmacopsychiatry 1990, 23, 108–113. 10.1055/s-2007-1014545. 1974069

[ref12] Kakimoto S.; Nagakura Y.; Tamura S.; Watabiki T.; Shibasaki K.; Tanaka S.; Mori M.; Sasamata M.; Okada M. Minodronic Acid, a Third-Generation Bisphosphonate, Antagonizes Purinergic P2X2/3 Receptor Function and Exerts an Analgesic Effect in Pain Models. Eur. J. Pharmacol. 2008, 589, 98–101. 10.1016/j.ejphar.2008.05.011. 18565509

[ref13] Dheer D.; Reddy K. R.; Rath S. K.; Sangwan P. L.; Das P.; Shankar R. Cu (I)-Catalyzed Double C–H Amination: Synthesis of 2-Iodo-Imidazo [1,2-*a*] Pyridines. RSC Adv. 2016, 6, 38033–38036. 10.1039/C6RA02953A.

[ref14] Goel R.; Luxami V.; Paul K. Imidazo [1, 2-*a*] Pyridines: Promising Drug Candidate for Antitumor Therapy. Curr. Top. Med. Chem. 2016, 16, 3590–3616. 10.2174/1568026616666160414122644. 27086790

[ref15] Bagdi A. K.; Rahman M.; Santra S.; Majee A.; Hajra A. Copper-Catalyzed Synthesis of Imidazo [1,2-*a*] Pyridines through Tandem Imine Formation-Oxidative Cyclization under Ambient Air: One-Step Synthesis of Zolimidine on a Gram-Scale. Adv. Synth. Catal. 2013, 355, 1741–1747. 10.1002/adsc.201300298.

[ref16] Stasyuk A. J.; Banasiewicz M.; Cyrański M. K.; Gryko D. T. Imidazo [1,2-*a*] Pyridines Susceptible to Excited State Intramolecular Proton Transfer: One-Pot Synthesis via an Ortoleva–King Reaction. J. Org. Chem. 2012, 77, 5552–5558. 10.1021/jo300643w. 22662878

[ref17] Sun K.; Mu S.; Liu Z.; Feng R.; Li Y.; Pang K.; Zhang B. Copper-Catalyzed C–N Bond Formation with Imidazo [1,2-*a*] Pyridines. Org. Biomol. Chem. 2018, 16, 6655–6658. 10.1039/C8OB01853G. 30183799

[ref18] Kaswan P.; Porter A.; Pericherla K.; Simone M.; Peters S.; Kumar A.; DeBoef B. Oxidative Cross-Coupling of Sp3-and Sp2-Hybridized C–H Bonds: Vanadium-Catalyzed Aminomethylation of Imidazo [1,2-*a*] Pyridines. Org. Lett. 2015, 17, 5208–5211. 10.1021/acs.orglett.5b02539. 26479446

[ref19] Lei S.; Mai Y.; Yan C.; Mao J.; Cao H. A Carbonylation Approach toward Activation of Csp2-H and Csp3-H Bonds: Cu-Catalyzed Regioselective Cross Coupling of Imidazo [1,2-*a*] Pyridines with Methyl Hetarenes. Org. Lett. 2016, 18, 3582–3585. 10.1021/acs.orglett.6b01588. 27388919

[ref20] Lu S.; Zhu X.; Li K.; Guo Y.-J.; Wang M.-D.; Zhao X.-M.; Hao X.-Q.; Song M.-P. Reactivity of P-Toluenesulfonylmethyl Isocyanide: Iron-Involved C–H Tosylmethylation of Imidazopyridines in Nontoxic Media. J. Org. Chem. 2016, 81, 8370–8377. 10.1021/acs.joc.6b01552. 27557624

[ref21] Samanta S.; Mondal S.; Santra S.; Kibriya G.; Hajra A. FeCl_3_-Catalyzed Cross-Dehydrogenative Coupling between Imidazoheterocycles and Oxoaldehydes. J. Org. Chem. 2016, 81, 10088–10093. 10.1021/acs.joc.6b02091. 27696879

[ref22] Li Z.; Hong J.; Zhou X. An Efficient and Clean CuI-Catalyzed Chalcogenylation of Aromatic Azaheterocycles with Dichalcogenides. Tetrahedron 2011, 67, 3690–3697. 10.1016/j.tet.2011.03.067.

[ref23] Ravi C.; Chandra Mohan D.; Adimurthy S. N-Chlorosuccinimide-Promoted Regioselective Sulfenylation of Imidazoheterocycles at Room Temperature. Org. Lett. 2014, 16, 2978–2981. 10.1021/ol501117z. 24838116

[ref24] Zhang J.; Zhan L.; Wei L.; Ning Y.; Zhong X.; Lai J.; Xu L.; Tang R. Metal-Free Thiolation of Imidazopyridines with Functionalized Haloalkanes Using Elemental Sulfur. Adv. Synth. Catal. 2018, 360, 533–543. 10.1002/adsc.201701190.

[ref25] Guo Y.-J.; Lu S.; Tian L.-L.; Huang E.-L.; Hao X.-Q.; Zhu X.; Shao T.; Song M.-P. Iodine-Mediated Difunctionalization of Imidazopyridines with Sodium Sulfinates: Synthesis of Sulfones and Sulfides. J. Org. Chem. 2018, 83, 338–349. 10.1021/acs.joc.7b02734. 29249143

[ref26] Yadav M.; Dara S.; Saikam V.; Kumar M.; Aithagani S. K.; Paul S.; Vishwakarma R. A.; Singh P. P. Regioselective Oxidative C–H Phosphonation of Imidazo [1, 2-a] Pyridines and Related Heteroarenes Mediated by Manganese (III) Acetate. Eur. J. Inorg. Chem. 2015, 2015, 6526–6533. 10.1002/ejoc.201500984.

[ref27] Dey A.; Singsardar M.; Sarkar R.; Hajra A. Environment-Friendly Protocol for the Chlorination of Imidazoheterocycles by Chloramine-T. ACS Omega 2018, 3, 3513–3521. 10.1021/acsomega.7b01844. 31458602 PMC6641228

[ref28] Mondal S.; Samanta S.; Singsardar M.; Mishra S.; Mitra S.; Hajra A. Zwitterionic-Type Molten Salt Catalyzed Iodination in Water: Synthesis of Iodoimidazoheterocycles. Synthesis 2016, 48, 4009–4015. 10.1055/s-0035-1562492.

[ref29] Liu P.; Gao Y.; Gu W.; Shen Z.; Sun P. Regioselective Fluorination of Imidazo [1, 2-a] Pyridines with Selectfluor in Aqueous Condition. J. Org. Chem. 2015, 80, 11559–11565. 10.1021/acs.joc.5b01961. 26523829

[ref30] Zhu Y.; Chu Q.; Li H.; Liu P.; Sun P. Electrochemical Primary Amination of Imidazopyridines with Azidotrimethylsilane under Mild Conditions. Green Chem. 2023, 296 10.1039/D2GC03703C.

[ref31] Tashrifi Z.; Mohammadi-Khanaposhtani M.; Larijani B.; Mahdavi M. C3-Functionalization of Imidazo [1, 2-a] Pyridines. Eur. J. Inorg. Chem. 2020, 2020, 269–284. 10.1002/ejoc.201901491.

[ref32] Schneider T. F.; Kaschel J.; Werz D. B. A New Golden Age for Donor–Acceptor Cyclopropanes. Angew. Chem., Int. Ed. 2014, 53, 5504–5523. 10.1002/anie.201309886. 24771660

[ref33] Boichenko M. A.; Andreev I. A.; Chagarovskiy A. O.; Levina I. I.; Zhokhov S. S.; Trushkov I. V.; Ivanova O. A. Ring Opening of Donor–Acceptor Cyclopropanes with Cyanide Ion and Its Surrogates. J. Org. Chem. 2019, 85, 1146–1157. 10.1021/acs.joc.9b03098. 31804074

[ref34] Singh K.; Bera T.; Jaiswal V.; Biswas S.; Mondal B.; Das D.; Saha J. Lewis Acid Catalyzed Nucleophilic Ring Opening and 1,3-Bisfunctionalization of Donor–Acceptor Cyclopropanes with Hydroperoxides: Access to Highly Functionalized Peroxy/(α-Heteroatom Substituted) Peroxy Compounds. J. Org. Chem. 2018, 84, 710–725. 10.1021/acs.joc.8b02561. 30565925

[ref35] Singh P.; Varshnaya R. K.; Dey R.; Banerjee P. Donor–Acceptor Cyclopropanes as an Expedient Building Block Towards the Construction of Nitrogen-Containing Molecules: An Update. Adv. Synth. Catal. 2020, 362, 1447–1484. 10.1002/adsc.201901332.

[ref36] Augustin A. U.; Jones P. G.; Werz D. B. Ring-Opening 1, 3-Aminochalcogenation of Donor–Acceptor Cyclopropanes: A Three-Component Approach. Chem. – Eur. J. 2019, 25, 11620–11624. 10.1002/chem.201902160. 31282001 PMC6771889

[ref37] Ivanov K. L.; Villemson E. V.; Budynina E. M.; Ivanova O. A.; Trushkov I. V.; Melnikov M. Y. Ring Opening of Donor–Acceptor Cyclopropanes with the Azide Ion: A Tool for Construction of N-Heterocycles. Chem. – Eur. J. 2015, 21, 4975–4987. 10.1002/chem.201405551. 25573783

[ref38] Xia Y.; Lin L.; Chang F.; Fu X.; Liu X.; Feng X. Asymmetric Ring-Opening of Cyclopropyl Ketones with Thiol, Alcohol, and Carboxylic Acid Nucleophiles Catalyzed by a Chiral N, N′-dioxide–Scandium (III) Complex. Angew. Chem., Int. Ed. 2015, 54, 13748–13752. 10.1002/anie.201506909. 26398505

[ref39] Irwin L. C.; Renwick C. R.; Kerr M. A. Nucleophilic Opening of Donor–Acceptor Cyclopropanes with Indoles via Hydrogen Bond Activation with 1,1,1,3,3,3-Hexafluoroisopropanol. J. Org. Chem. 2018, 83, 6235–6242. 10.1021/acs.joc.8b00894. 29757647

[ref40] Chagarovskiy A. O.; Budynina E. M.; Ivanova O. A.; Grishin Y. K.; Trushkov I. V.; Verteletskii P. V. Lewis Acid-Catalyzed Reactions of Donor–Acceptor Cyclopropanes with Furan Derivatives. Tetrahedron 2009, 65, 5385–5392. 10.1016/j.tet.2009.04.061.

[ref41] Richmond E.; Vuković V. D.; Moran J. Nucleophilic Ring Opening of Donor–Acceptor Cyclopropanes Catalyzed by a Brønsted Acid in Hexafluoroisopropanol. Org. Lett. 2018, 20, 574–577. 10.1021/acs.orglett.7b03688. 29345947

[ref42] Richmond E.; Yi J.; Vuković V. D.; Sajadi F.; Rowley C. N.; Moran J. Ring-Opening Hydroarylation of Monosubstituted Cyclopropanes Enabled by Hexafluoroisopropanol. Chem. Sci. 2018, 9, 6411–6416. 10.1039/C8SC02126K. 30310570 PMC6115651

[ref43] Kilic H.; Dalkilic O. The Reaction of Donor-Acceptor Cyclopropanes with 4, 7-dihydroindole: A New Protocol for the Synthesis of Divergent C2-alkylated Indoles. ChemistrySelect 2019, 4, 3737–3740. 10.1002/slct.201900268.

[ref44] Maloney T. P.; Murphy K. L.; Mainsah T. L.; Nolin K. A. Friedel-Crafts Alkylation of Benzo [b] Furan with Activated Cyclopropanes Catalyzed by a Calcium (II) Complex. Tetrahedron Lett. 2018, 59, 18–21. 10.1016/j.tetlet.2017.10.064.

[ref45] Von Köller H. F.; Jones P. G.; Werz D. B. A Widely Applicable and Versatile Method for the Ring-Opening 1,3-Carbocarbonation of Donor-Acceptor Cyclopropanes. Chem. – Eur. J. 2023, 29, e202203986 10.1002/chem.202203986. 36579656

[ref46] Das S.; Daniliuc C. G.; Studer A. Multicomponent 1,3-Bifunctionalization of Donor–Acceptor Cyclopropanes with Arenes and Nitrosoarenes. Org. Lett. 2016, 18, 5576–5579. 10.1021/acs.orglett.6b02815. 27809550

[ref47] Oliver G. A.; Werz D. B. Ring-Opening 1, 3-Sulfonylation-Fluorination of Donor–Acceptor Cyclopropanes: Three-Component Access to γ-Fluorosulfones. Org. Lett. 2023, 25, 3568–3572. 10.1021/acs.orglett.3c01208. 37159931

[ref48] Guin A.; Rathod T.; Gaykar R. N.; Roy T.; Biju A. T. Lewis Acid Catalyzed Ring-Opening 1,3-Aminothiolation of Donor–Acceptor Cyclopropanes Using Sulfenamides. Org. Lett. 2020, 22, 2276–2280. 10.1021/acs.orglett.0c00483. 32129074

[ref49] Deswal S.; Guin A.; Biju A. T. Benzotriazole-Triggered Three-Component Lewis Acid-Catalyzed Ring-Opening 1,3-Aminofunctionalization of Donor–Acceptor Cyclopropanes. Org. Lett. 2023, 25, 1643–1648. 10.1021/acs.orglett.3c00180. 36876870

[ref50] Wallbaum J.; Garve L. K. B.; Jones P. G.; Werz D. B. Ring-Opening Regio-, Diastereo-, and Enantioselective 1,3-Chlorochalcogenation of Cyclopropyl Carbaldehydes. Chem. – Eur. J. 2016, 22, 18756–18759. 10.1002/chem.201605265. 27868248 PMC6680189

[ref51] Li K.; Zhu X.; Lu S.; Zhou X.-Y.; Xu Y.; Hao X.-Q.; Song M.-P. Catalyst-Free Friedel–Crafts Alkylation of Imidazo [1,2-*a*] Pyridines. Synlett 2016, 27, 387–390. 10.1039/C5RA15678E.

[ref52] Park H. J.; Jun J. An Efficient Copper-catalyzed Nucleophilic Addition to N-Acyliminium Ions Derived from N-Benzyloxycarbonylamino Sulfones: A Novel Approach to C-3 Functionalization of 2-Phenylimidazo [1, 2-a] Pyridine. Bull. Korean Chem. Soc. 2017, 38, 1123–1128. 10.1002/bkcs.11228.

[ref53] Kim H.; Byeon M.; Jeong E.; Baek Y.; Jeong S. J.; Um K.; Han S. H.; Han G. U.; Ko G. H.; Maeng C.; Son J. Y.; Kim D.; Kim S. H.; Lee K.; Lee P. H. Rhodium (II)-Catalyzed Regioselective C3-Alkylation of 2-Arylimidazo [1,2-*a*] Pyridines with Aryl Diazoesters. Adv. Synth. Catal. 2019, 361, 2094–2106. 10.1002/adsc.201900092.

[ref54] Lafzi F.; Kilic H. Metal-and Additive-Free C3-Functionalization of Imidazo [1,2-*a*] Pyridines with *Para*-Quinone Methides. Asian J. Org. Chem. 2021, 10, 1814–1821. 10.1002/ajoc.202100313.

[ref55] Novikov R. A.; Tarasova A. V.; Korolev V. A.; Timofeev V. P.; Tomilov Y. V. A New Type of Donor–Acceptor Cyclopropane Reactivity: The Generation of Formal 1,2-and 1,4-Dipoles. Angew. Chem., Int. Ed. 2014, 53, 3187–3191. 10.1002/anie.201306186. 24554497

