# Supplementary material for: Regioselective C3–H Alkylation of Imidazopyridines with Donor–Acceptor Cyclopropanes
Source: J Org Chem. 2023 Aug 3;88(16):11834–46. doi: 10.1021/acs.joc.3c01122 (PMC10443042; doi:10.1021/acs.joc.3c01122)
Supplement: Supplementary file 1 — jo3c01122_si_001.pdf [file jo3c01122_si_001.pdf]

Supporting Information

Regioselective C3-H Alkylation of Imidazopyridines with Donor-Acceptor Cyclopropanes

Oguzhan Dalkilic[†], Ozge Turbedaroglu[†], Ferruh Lafzi, Haydar Kilic*

Department of Chemistry, Faculty of Sciences, Atatürk University, Erzurum 25240, Türkiye

Table of Contents

**General procedure for the synthesis of cyclopropanes 2a-o**	**S1-S2**
**References**	**S2-S3**
**NMR Spectra**	**S4-S57**

Email: hydk@atauni.edu.tr

[†]O.D. and O.T. contributed equally to this work.

General procedure for the synthesis of cyclopropanes 2a-o

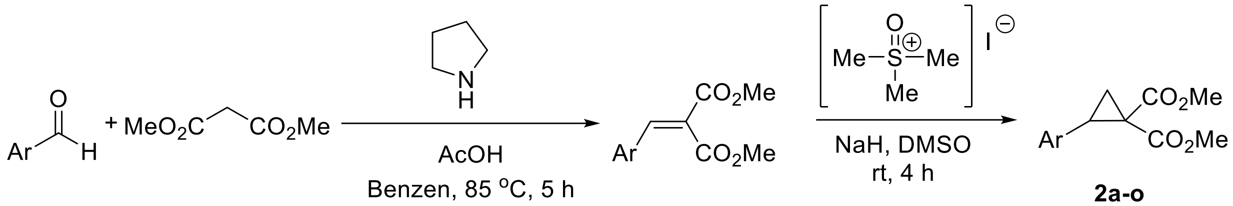

2a-o

The solution of aromatic aldehyde (1 equiv.), dimethylmalonate (1 equiv.), piperidine (0.1 equiv.) and acetic acid (0.02 equiv.) in 40 mL benzen was refluxed with a Dean-Stark attachment during 5 h until no water was extracted. The reaction mixture was washed with HCl (5% in water, 3×30 mL) and $NaHCO_3$ (5% in water, 3×30 mL). The organic layer was dried over Na_2SO_4 and the solvent was evaporated. The obtained arylidenemalonates were used in the next step without additional purification.

To a stirred suspension of NaH (1.1 equiv.) in dry DMSO (20 mL) in argon atmosphere was added trimethylsulfoxonium iodide (1.1 equiv.) under the same conditions. Then a solution of arylidenemalonate (1 equiv.) in dry DMSO (7 mL) was added in a single portion. The resulted mixture was stirred at room temperature, poured into H_2O–ice, and extracted with diethyl ether (3×40 mL). The combined organic layers were washed with water (3×40 mL), dried over Na_2SO_4, and concentrated in vacuo to give the pure cyclopropanes **2** in good yields.

Dimethyl 2-(4-methoxyphenyl)cyclopropane-1,1-dicarboxylate (**2a**)[1]: Column chromatography (EtOAC:hexane (0.5:9.5)) gave the product as a colorless oil. ^{1}H NMR (400 MHz, $CDCl_3$) δ 7.04 (d, *J* = 8.6 Hz, 2H), 6.73 (d, *J* = 8.6 Hz, 2H), 3.71 (s, 3H), 3.70 (s, 3H), 3.32 (s, 3H), 3.11 (t, *J* = 8.6 Hz, 1H), 2.08 (dd, *J* = 8.0, 5.2 Hz, 1H), 1.65 (dd, *J* = 9.3, 5.2 Hz, 1H).

Dimethyl 2-(2,5-dimethoxyphenyl)cyclopropane-1,1-dicarboxylate (**2b**)[2]: Column chromatography (EtOAC:hexane (0.5:9.5)) colorless oil. ^{1}H NMR (400 MHz, $CDCl_3$) δ 6.78 – 6.75 (m, 2H), 6.59 (d, *J* = 2.4 Hz, 1H), 3.81 (s, 3H), 3.80 (s, 3H), 3.75 (s, 3H), 3.40 (s, 3H), 3.33 (t, *J* = 8.9 Hz, 1H), 2.18 (dd, *J* = 8.4, 5.1 Hz, 1H), 1.75 (dd, *J* = 9.2, 5.1 Hz, 1H).

Dimethyl 2-(3,4,5-trimethoxyphenyl)cyclopropane-1,1-dicarboxylate (**2c**)[3]: Column chromatography (EtOAC:hexane (0.5:9.5)) colorless oil. ^{1}H NMR (400 MHz, $CDCl_3$) δ 6.43 (s, 2H), 3.85 (s, 6H), 3.83 (s, 3H), 3.81 (s, 3H), 3.47 (s, 3H), 3.20 (t, *J* = 8.6 Hz, 1H), 2.15 (dd, *J* = 7.9, 5.2 Hz, 1H), 1.75 (dd, *J* = 9.2, 5.2 Hz, 1H).

Dimethyl 2-(4-fluorophenyl)cyclopropane-1,1-dicarboxylate (**2d**)[1]: Column chromatography (EtOAC:hexane (0.3:9.7)) colorless oil. ^{1}H NMR (400 MHz, $CDCl_3$) δ 7.23 – 7.10 (m, 2H), 7.02 – 6.70 (m, 2H), 3.79 (s, 3H), 3.39 (s, 3H), 3.20 (t, *J* = 8.6 Hz, 1H), 2.15 (dd, *J* = 8.0, 5.3 Hz, 1H), 1.74 (dd, *J* = 9.3, 5.2 Hz, 1H).

Dimethyl 2-(4-chlorophenyl)cyclopropane-1,1-dicarboxylate (**2e**)[1]: Column chromatography (EtOAC:hexane (0.3:9.7)) colorless oil. ^{1}H NMR (400 MHz, $CDCl_3$) δ 7.17 (d, *J* = 8.4 Hz, 2H), 7.05 (d, *J* = 8.4 Hz, 2H), 3.72 (s, 3H), 3.33 (s, 3H), 3.11 (t, *J* = 8.6 Hz, 1H), 2.08 (dd, *J* = 7.9, 5.4 Hz, 1H), 1.67 (dd, *J* = 9.2, 5.4 Hz, 1H).

Dimethyl 2-(4-bromophenyl)cyclopropane-1,1-dicarboxylate (**2f**)[1]: Column chromatography (EtOAC:hexane (0.3:9.7)) Colorless oil**.** ^{1}H NMR (400 MHz, $CDCl_3$) δ 7.32 (d, *J* = 8.5 Hz, 2H), 7.00 (d, *J* = 8.5 Hz, 2H), 3.72 (s, 3H), 3.34 (s, 3H), 3.09 (t, *J* = 8.6 Hz, 1H), 2.07 (dd, *J* = 8.0, 5.3 Hz, 1H), 1.67 (dd, *J* = 9.2, 5.3 Hz, 1H).

Dimethyl 2-(4-iodophenyl)cyclopropane-1,1-dicarboxylate (**2g**)[4]: Column chromatography (EtOAC:hexane (0.3:9.7)) Colorless oil. ^{1}H NMR (400 MHz, $CDCl_3$) δ 7.59 (d, *J* = 8.4 Hz, 2H), 6.93 (d, *J* = 8.4 Hz, 2H), 3.78 (s, 3H), 3.40 (s, 3H), 3.21 – 3.09 (m, 1H), 2.13 (dd, *J* = 8.0, 5.3 Hz, 1H), 1.73 (dd, *J* = 9.2, 5.3 Hz, 1H).

Dimethyl 2-(4-nitrophenyl)cyclopropane-1,1-dicarboxylate (**2h**)[5]: Column chromatography (EtOAC:hexane (0.4:9.6)) white solid (mp 135.0-136.0 °C). ^{1}H NMR (400 MHz, $CDCl_3$) δ 8.17 (d, *J* = 8.7 Hz, 2H), 7.38 (d, *J* = 8.7 Hz, 2H), 3.83 (s, 3H), 3.44 (s, 3H), 3.30 (t, *J* = 8.6 Hz, 1H), 2.25 (dd, *J* = 8.0, 5.5 Hz, 1H), 1.86 (dd, *J* = 9.1, 5.5 Hz, 1H).

Dimethyl 2-(4-cyanophenyl)cyclopropane-1,1-dicarboxylate (**2i**)[5]: Column chromatography (EtOAC:hexane (0.3:9.7)) white solid (mp 114.0-115.0 °C). ^{1}H NMR (400 MHz, $CDCl_3$) δ 7.51 (d, *J* = 8.3 Hz, 2H), 7.23 (d, *J* = 8.3 Hz, 2H), 3.74 (s, 3H), 3.34 (s, 3H), 3.24 – 3.08 (m, 1H), 2.13 (dd, *J* = 8.0, 5.4 Hz, 1H), 1.73 (dd, *J* = 9.1, 5.4 Hz, 1H).

Dimethyl 2-phenylcyclopropane-1,1-dicarboxylate (**2j**)[2]: Column chromatography (EtOAC:hexane (0.2:9.8)) colorless oil. ^{1}H NMR (400 MHz, $CDCl_3$) δ 7.35 – 7.05 (m, 5H), 3.80 (s, 3H), 3.36 (s, 3H), 3.25 (t, *J* = 8.6 Hz, 1H), 2.21 (dd, *J* = 8.0, 5.3 Hz, 1H), 1.75 (dd, *J* = 9.2, 5.2 Hz, 1H).

Dimethyl 2-(pyren-2-yl)cyclopropane-1,1-dicarboxylate (**2k**)[2]: Column chromatography (EtOAC:hexane (0.3:9.7)) yellow solid (mp: 167.0-168.0 °C). ^{1}H NMR (400 MHz, $CDCl_3$) δ 8.43 (d, *J* = 9.1 Hz, 1H), 8.25 – 8.13 (m, 3H), 8.13 – 7.97 (m, 4H), 7.84 (d, *J* = 7.9 Hz, 1H), 3.99 (t, *J* = 8.6 Hz, 1H), 3.94 (s, 2H), 2.96 (s, 3H), 2.64 (dd, *J* = 7.8, 5.2 Hz, 1H), 2.00 (dd, *J* = 8.9, 5.0 Hz, 1H).

Dimethyl 2-(4-(diphenylamino)phenyl)cyclopropane-1,1-dicarboxylate (**2l**)[2]: Column chromatography (EtOAC:hexane (0.3:9.7)) yellow oil. ^{1}H NMR (400 MHz, $CDCl_3$) δ 7.31 – 7.22 (m, 4H), 7.12 – 6.98 (m, 10H), 3.82 (s, 3H), 3.48 (s, 3H), 3.22 (t, *J* = 8.6 Hz, 1H), 2.20 (dd, *J* = 8.0, 5.2 Hz, 1H), 1.77 (dd, *J* = 9.3, 5.2 Hz, 1H).

Dimethyl 2-(naphthalen-2-yl)cyclopropane-1,1-dicarboxylate (**2m**)[1]: Column chromatography (EtOAC:hexane (0.2:9.8)) colorless oil. ^{1}H NMR (400 MHz, $CDCl_3$) δ 7.90 – 7.72 (m, 3H), 7.66 (s, 1H), 7.48 (dd, *J* = 6.3, 2.4 Hz, 2H), 7.36 (d, *J* = 8.5 Hz, 1H), 3.84 (s, 3H), 3.41 (t, *J* = 8.6 Hz, 1H), 3.32 (s, 3H), 2.36 (dd, *J* = 8.0, 5.3 Hz, 1H), 1.86 (dd, *J* = 9.2, 5.3 Hz, 1H).

Dimethyl 2-(furan-2-yl)cyclopropane-1,1-dicarboxylate (**2n**)[6]: Column chromatography (EtOAC:hexane (0.2:9.8)) yellow oil. ^{1}H NMR (400 MHz, $CDCl_3$) δ 7.31 (d, *J* = 1.1 Hz, 1H), 6.56 – 6.20 (m, 1H), 6.14 (d, *J* = 3.2 Hz, 1H), 3.79 (s, 3H), 3.56 (s, 3H), 3.20 – 3.02 (m, 1H), 2.10 (dd, *J* = 7.7, 5.1 Hz, 1H), 1.80 (dd, *J* = 9.5, 5.1 Hz, 1H).

Dimethyl 2-(thiophen-2-yl)cyclopropane-1,1-dicarboxylate (**2o**)[6]: Column chromatography (EtOAC:hexane (0.2:9.8)) yellow oil. ^{1}H NMR (400 MHz, $CDCl_3$) δ 7.18 (dd, *J* = 5.1, 1.1 Hz, 1H), 6.92 (dd, *J* = 5.1, 3.5 Hz, 1H), 6.86 (d, *J* = 3.5 Hz, 1H), 3.81 (s, 3H), 3.50 (s, 3H), 3.31 (t, *J* = 8.5 Hz, 1H), 2.17 (dd, *J* = 7.7, 5.1 Hz, 1H), 1.85 (dd, *J* = 9.3, 5.1 Hz, 1H).

References

1. Novikov, R. A.; Tarasova, A. V; Korolev, V. A.; Timofeev, V. P.; Tomilov, Y. V. A New Type of Donor–Acceptor Cyclopropane Reactivity: The Generation of Formal 1,2-and 1,4-Dipoles. *Angew. Chem. Int. Ed.* ***2014***, 53, 3187 –3191.

2. Kilic, H.; Dalkilic, O. The Reaction of Donor-Acceptor Cyclopropanes with 4, 7-dihydroindole: A New Protocol for the Synthesis of Divergent C2-alkylated Indoles. *ChemistrySelect,* **2019**, *4*, 3737–3740.

3. Chagarovskiy, A. O., Kuznetsov, V. V., Ivanova, O. A., Goloveshkin, A. S., Levina, I. I., Makhova, N. N., Trushkov, I. V. Synthesis of 1-Substituted Pyrazolines by Reaction of Donor-Acceptor Cyclopropanes with 1, 5-Diazabicyclo [3.1. 0] hexanes. *Eur. J. Org. Chem.* **2019***,* (31-32), 5475-5485.

4. Xiong, H., Xu, H., Liao, S., Xie, Z., Tang, Y. Copper-catalyzed highly enantioselective cyclopentannulation of indoles with donor–acceptor cyclopropanes. *J. Am. Chem. Soc.* **2013**, 135, 7851-7854.

5. Richmond, E., Vuković, V. D., Moran, J. Nucleophilic ring opening of Donor–Acceptor cyclopropanes catalyzed by a Brønsted Acid in hexafluoroisopropanol. *Org. let.* **2018**, *20*, 574-577.

6. Talukdar, R., Tiwari, D. P., Saha, A., Ghorai, M. K. Diastereoselective Synthesis of Functionalized Tetrahydrocarbazoles via a Domino-Ring Opening–Cyclization of Donor–Acceptor Cyclopropanes with Substituted 2-Vinylindoles. *Org. let.* **2014**, 16, 3954-3957.

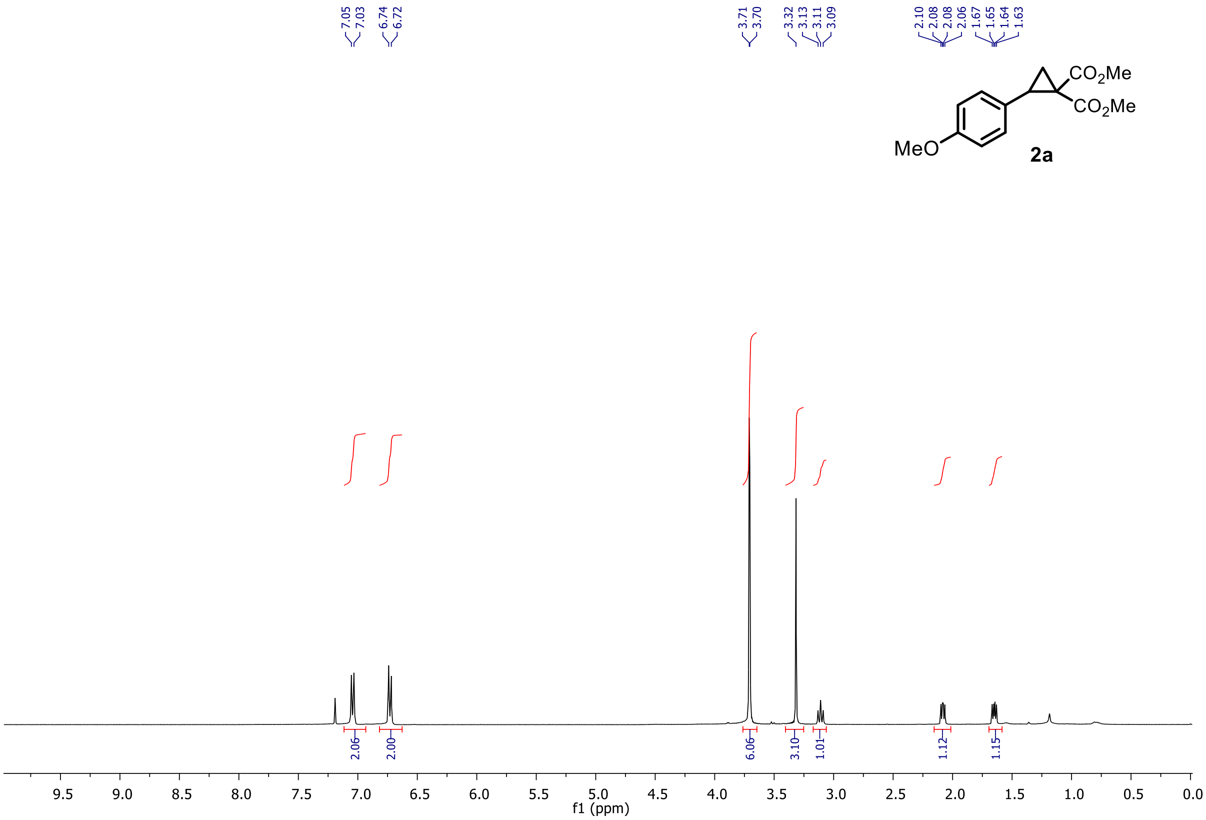

^{1}H NMR (400 MHz) spectra of **2a** ($CDCl_3$).

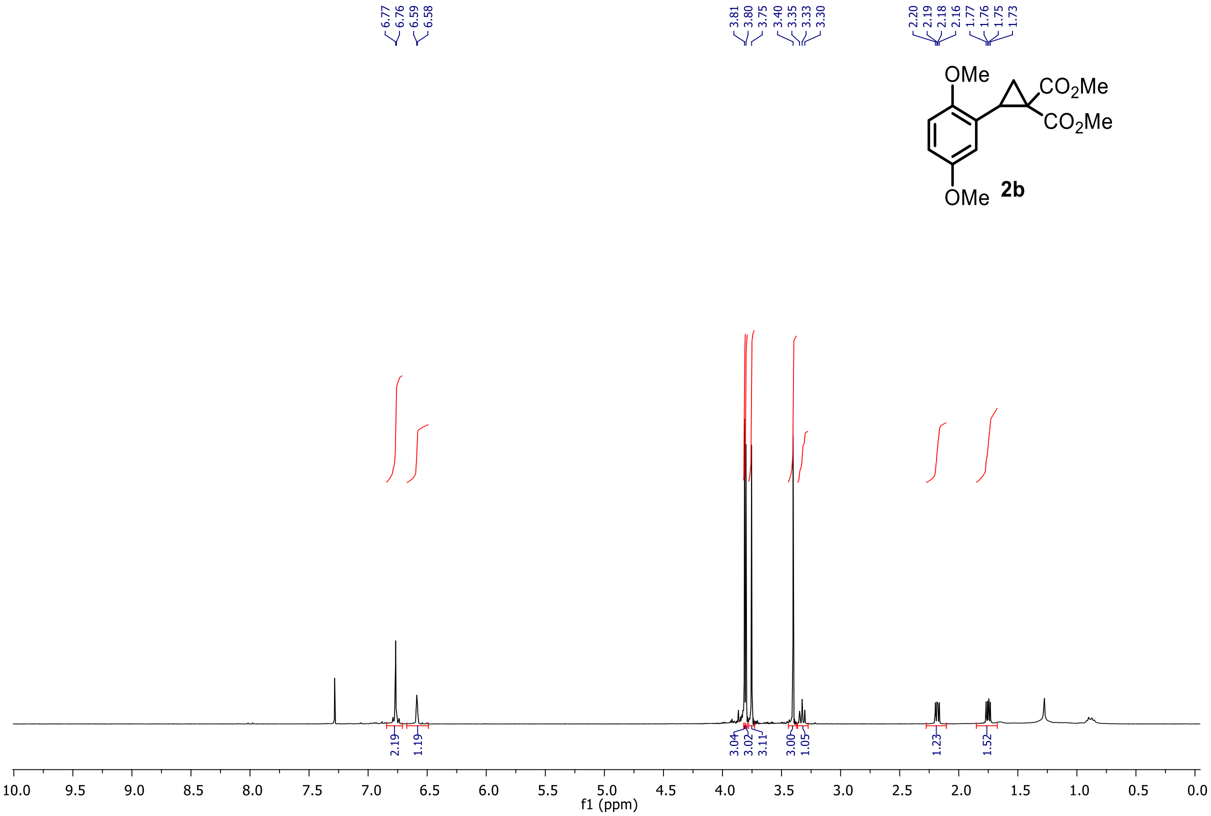

^{1}H NMR (400 MHz) spectra of **2b** ($CDCl_3$).

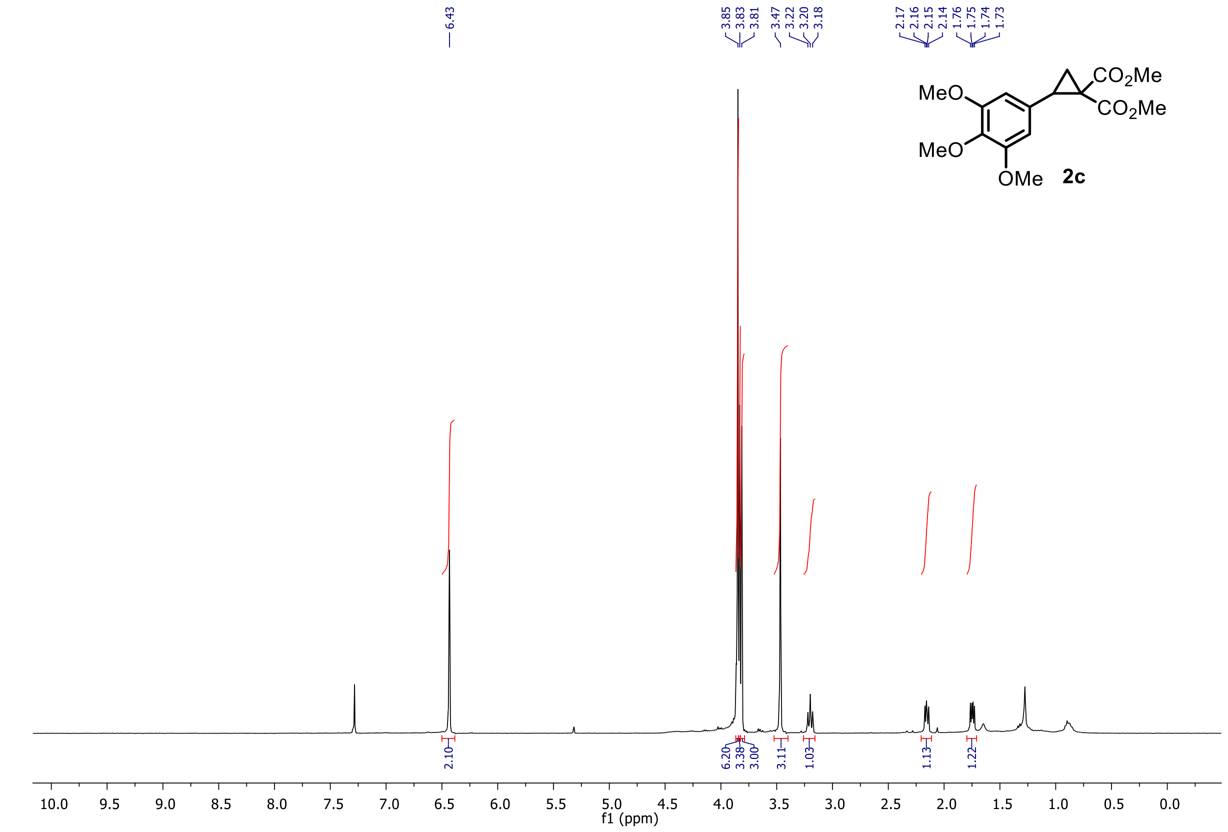

^{1}H NMR (400 MHz) spectra of **2c** ($CDCl_3$).

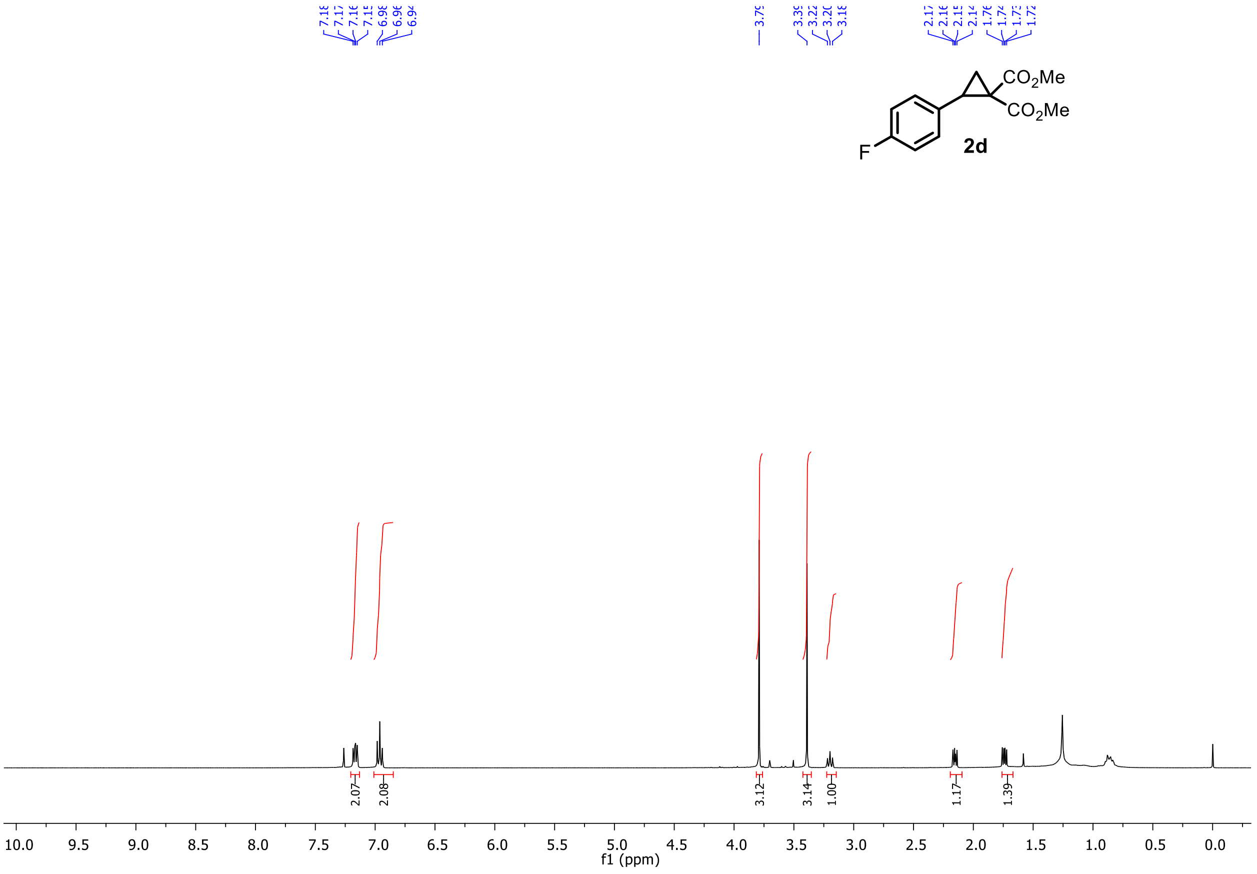

^{1}H NMR (400 MHz) spectra of **2d** ($CDCl_3$).

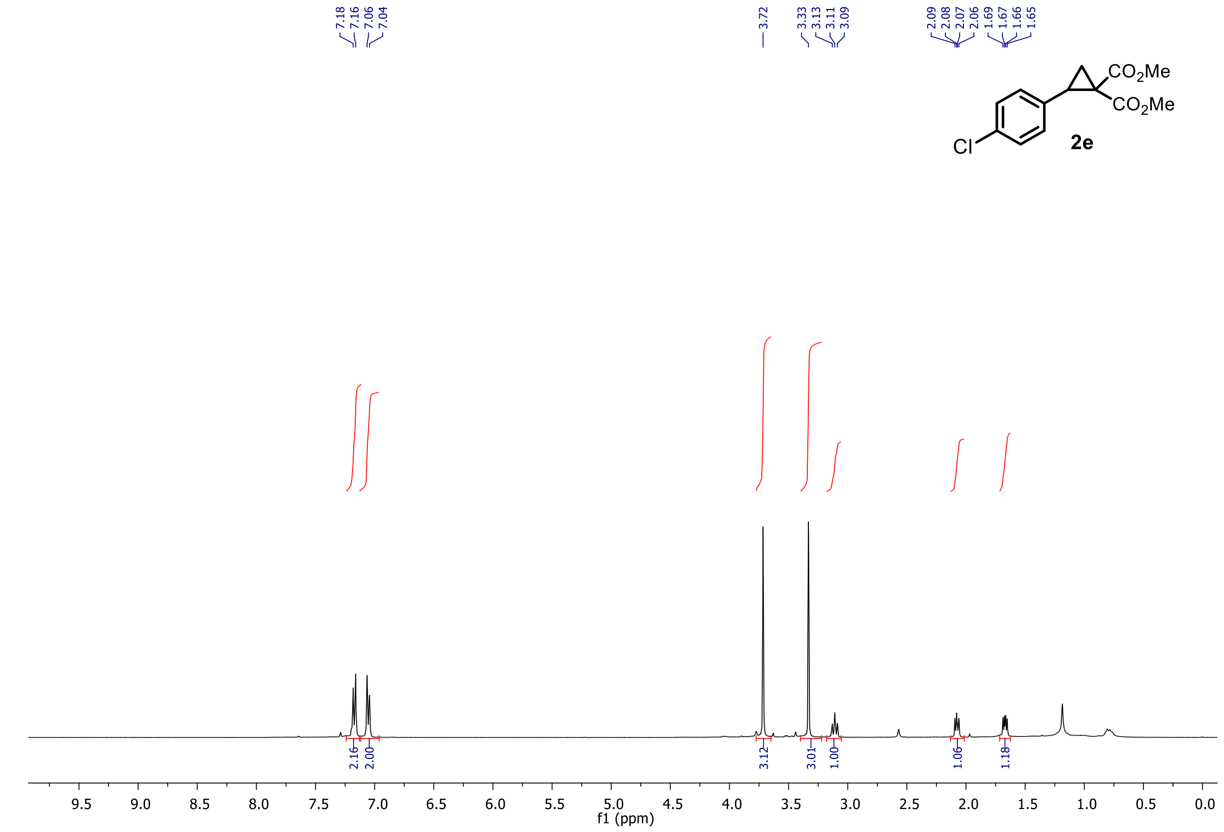

^{1}H NMR (400 MHz) spectra of **2e** ($CDCl_3$).

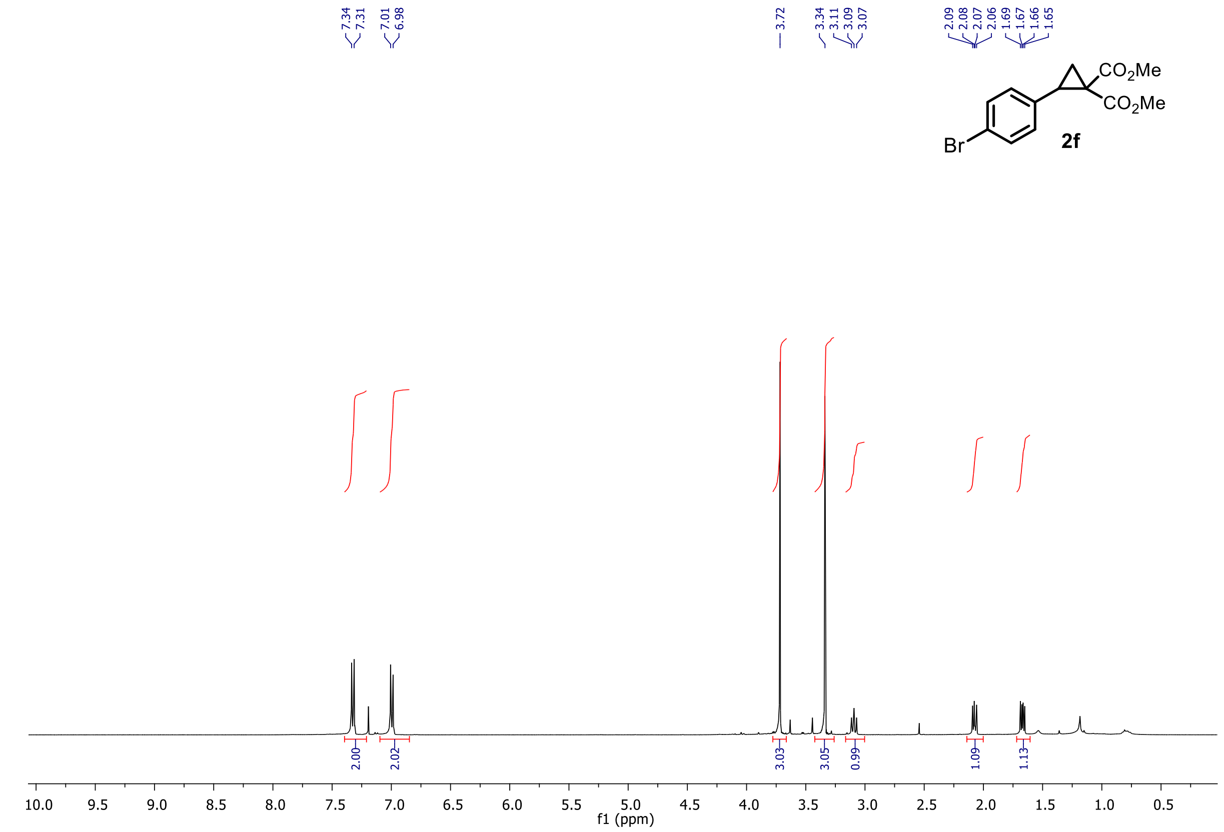

^{1}H NMR (400 MHz) spectra of **2f** ($CDCl_3$).

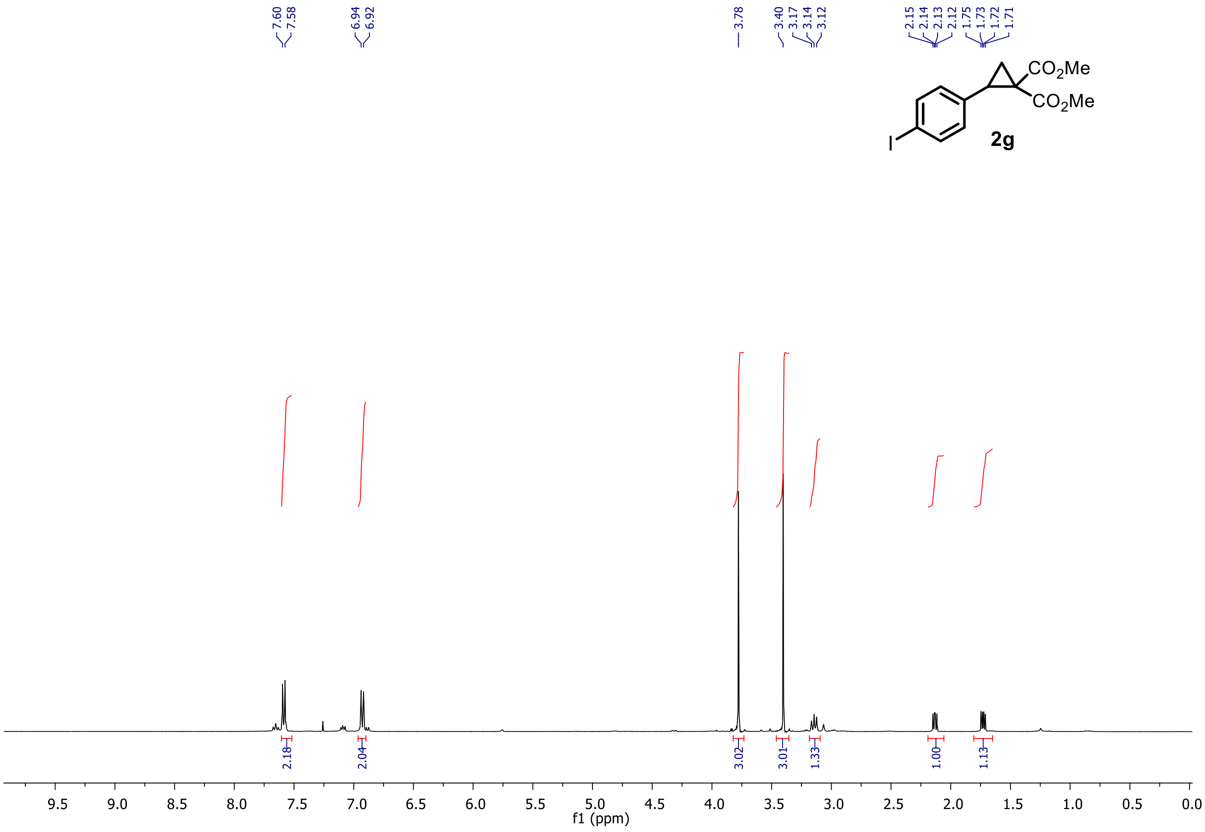

^{1}H NMR (400 MHz) spectra of **2g** ($CDCl_3$).

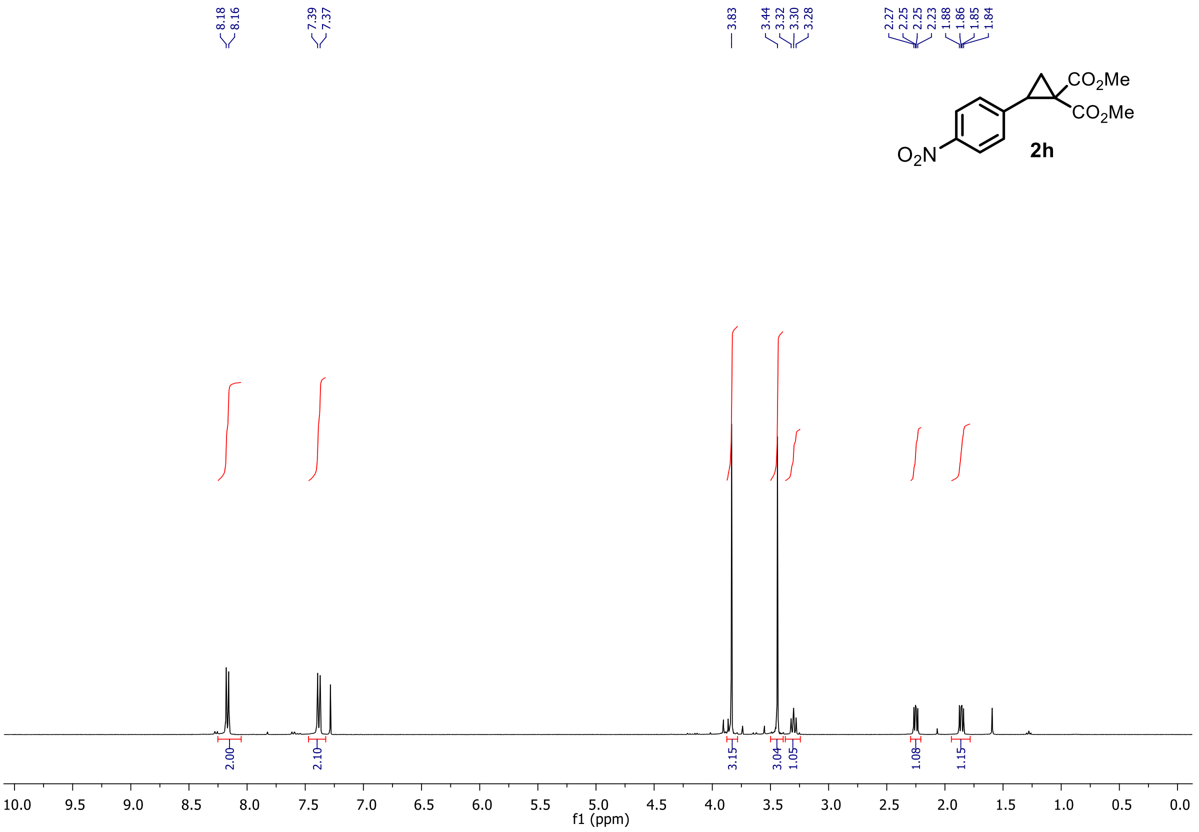

^{1}H NMR (400 MHz) spectra of **2h** ($CDCl_3$).

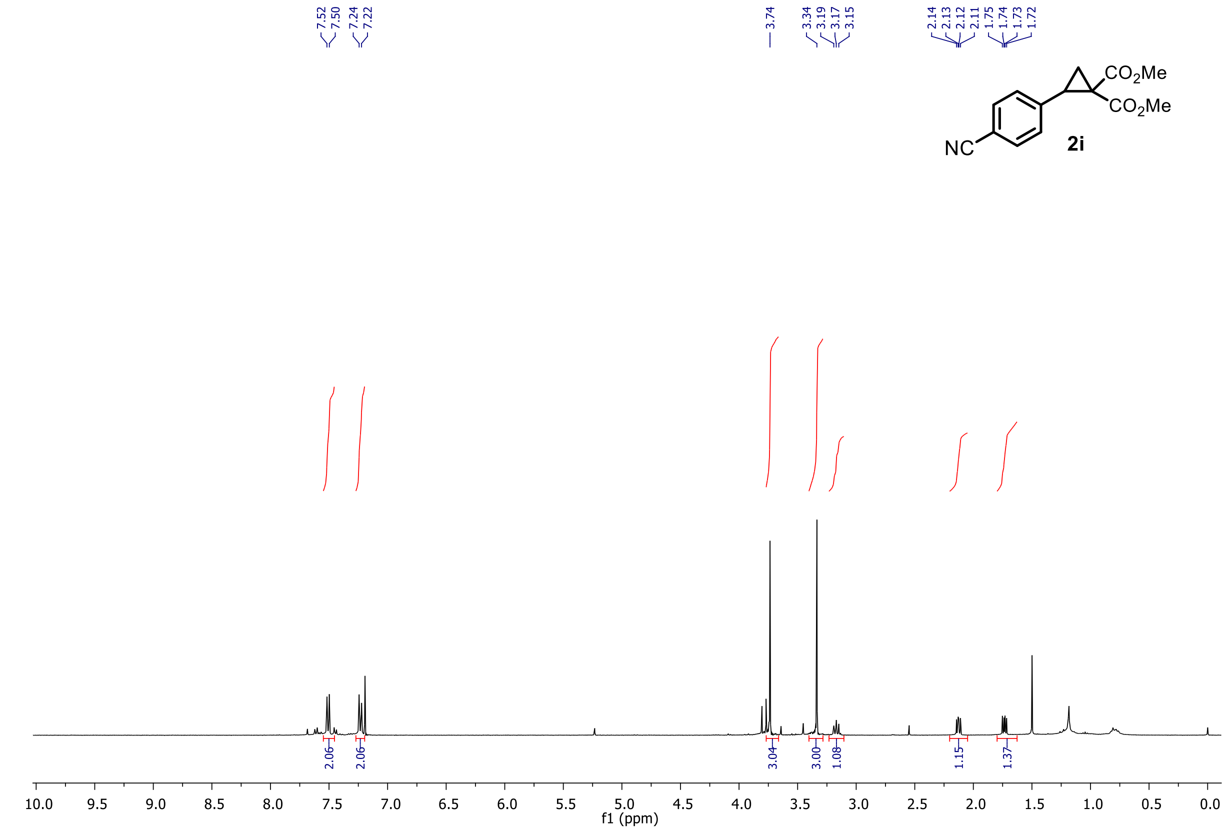

^{1}H NMR (400 MHz) spectra of **2i** ($CDCl_3$).

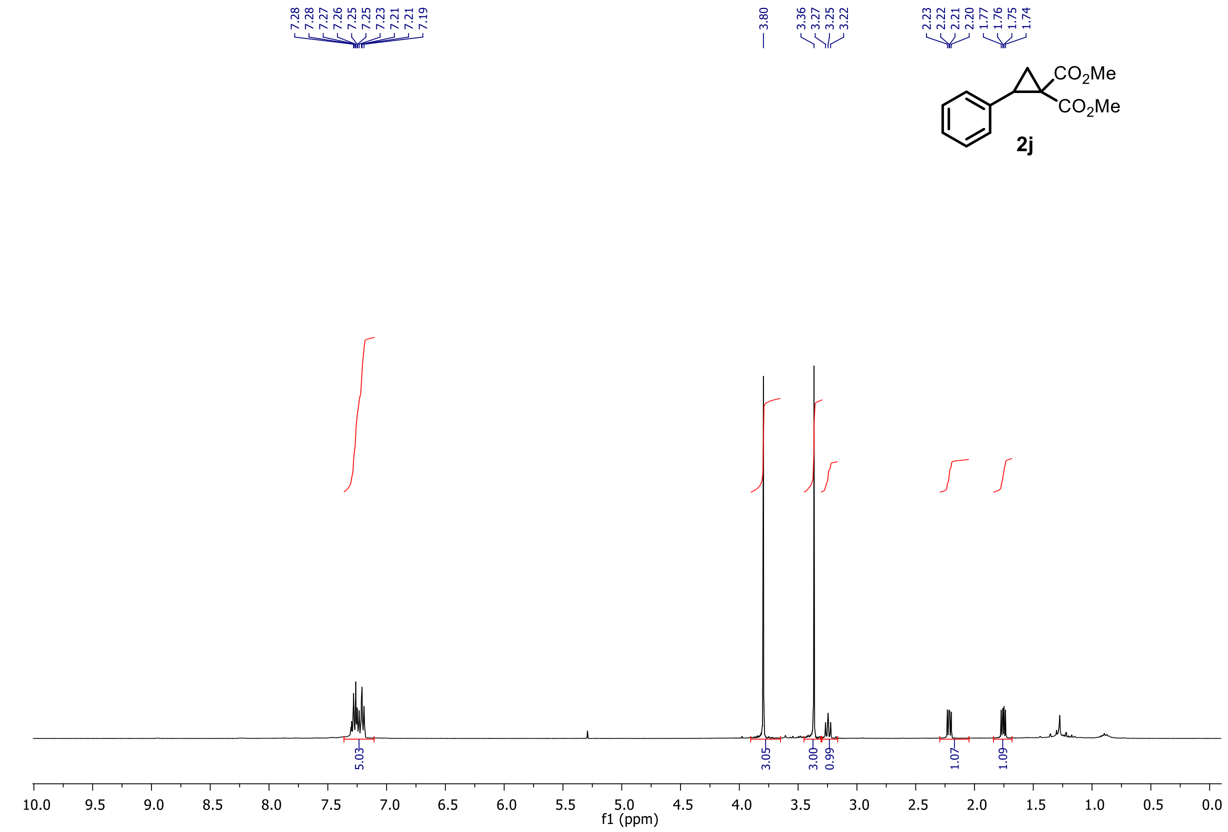

^{1}H NMR (400 MHz) spectra of **2j** ($CDCl_3$).

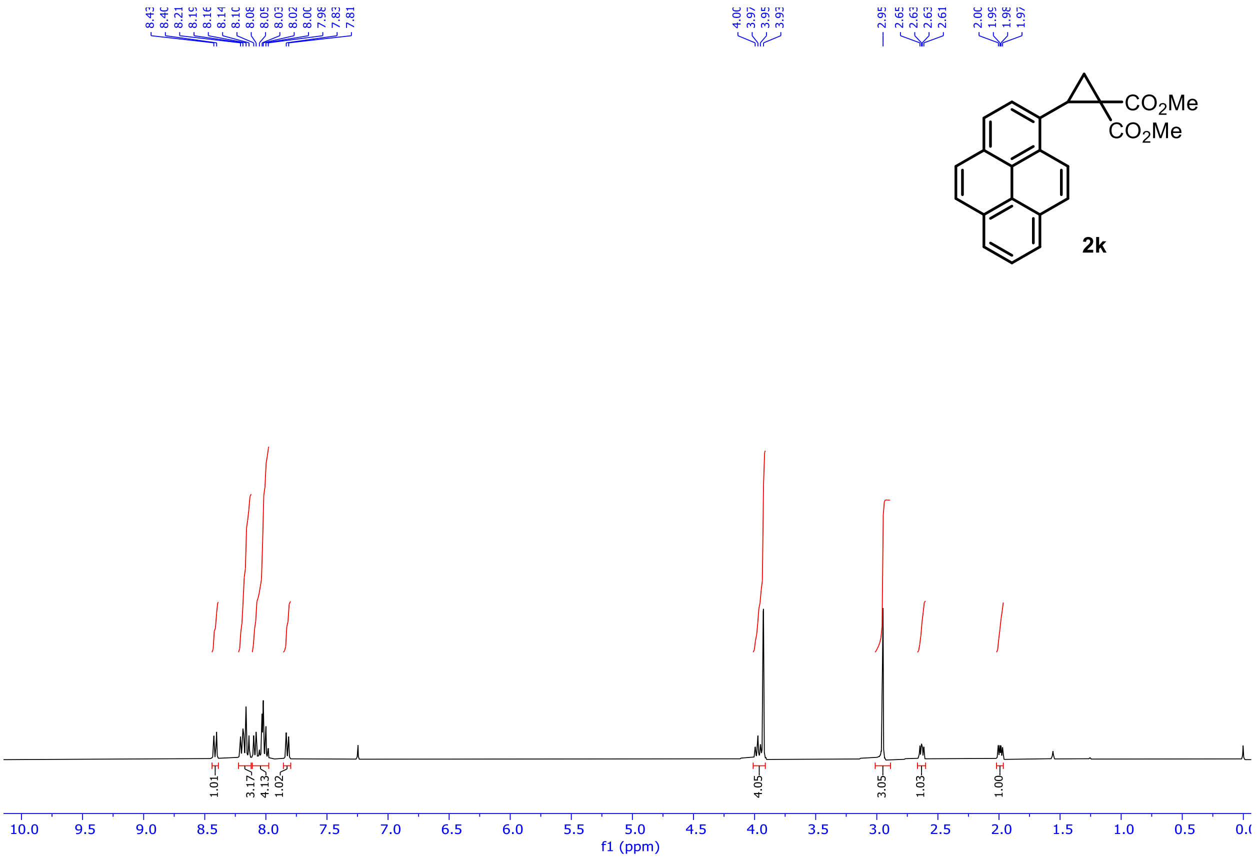

^{1}H NMR (400 MHz) spectra of **2k** ($CDCl_3$).

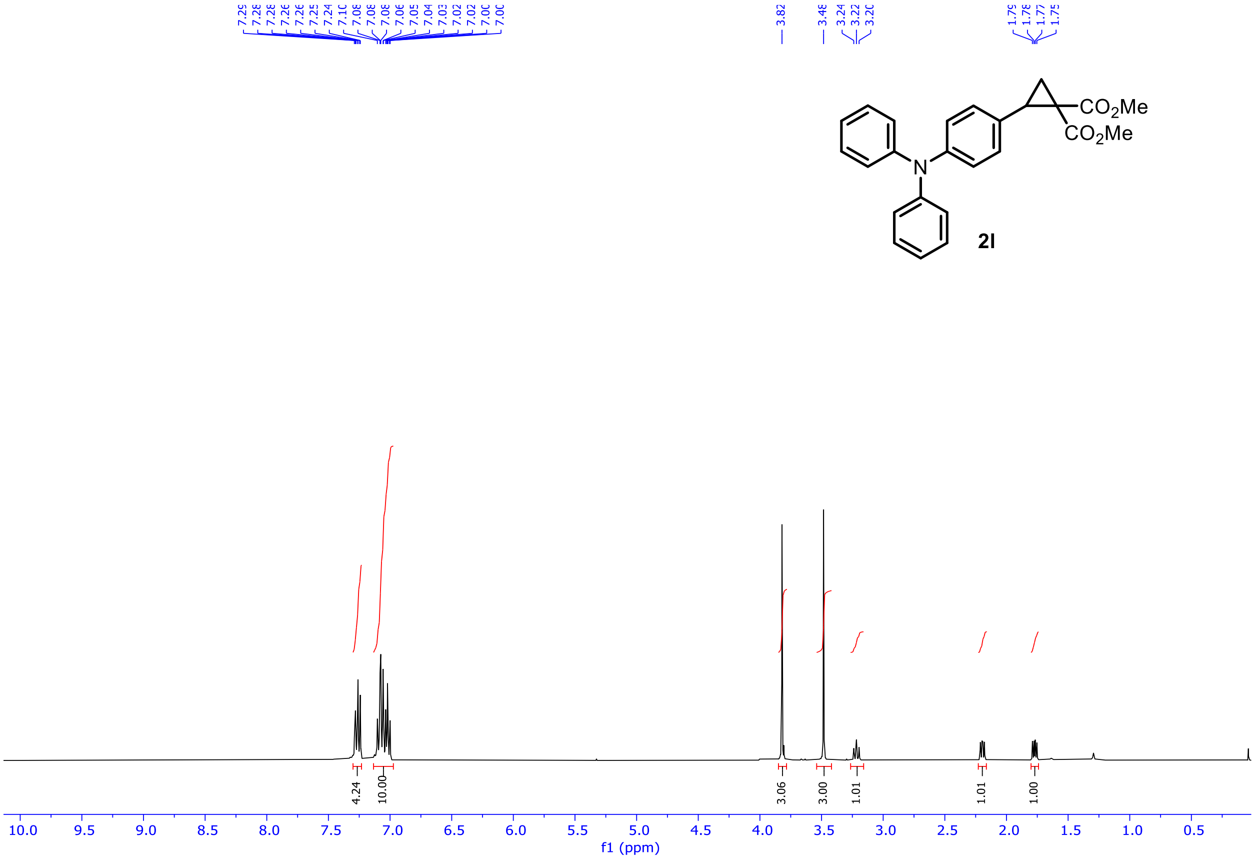

^{1}H NMR (400 MHz) spectra of **2l** ($CDCl_3$).

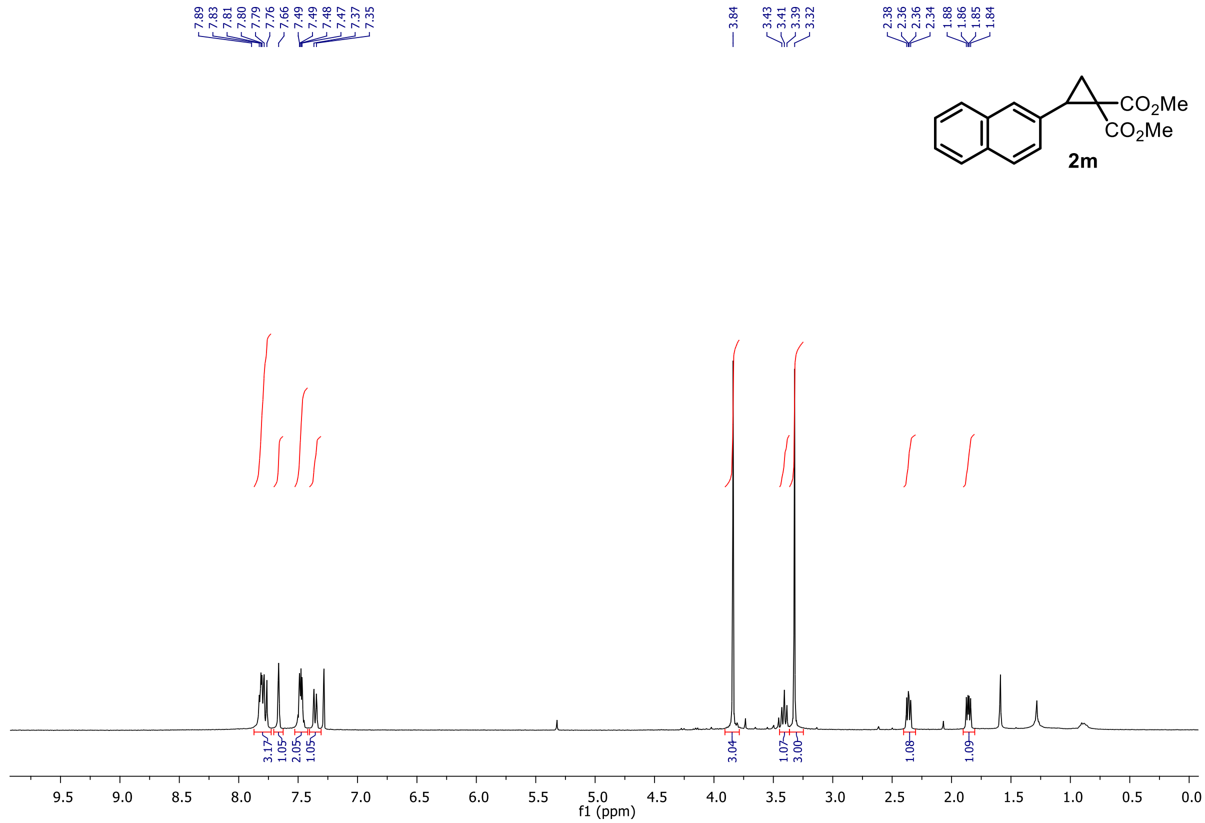

^{1}H NMR (400 MHz) spectra of **2m** ($CDCl_3$).

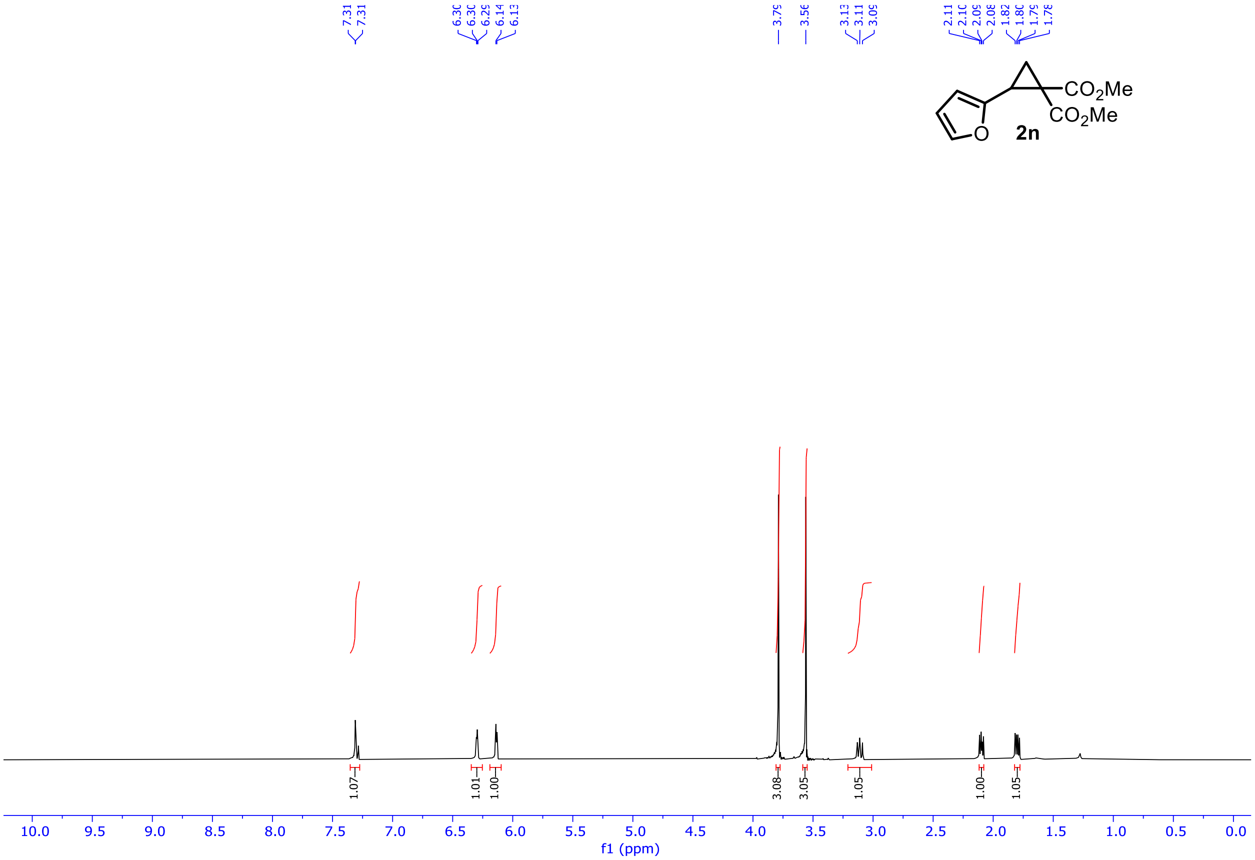

^{1}H NMR (400 MHz) spectra of **2n** ($CDCl_3$).

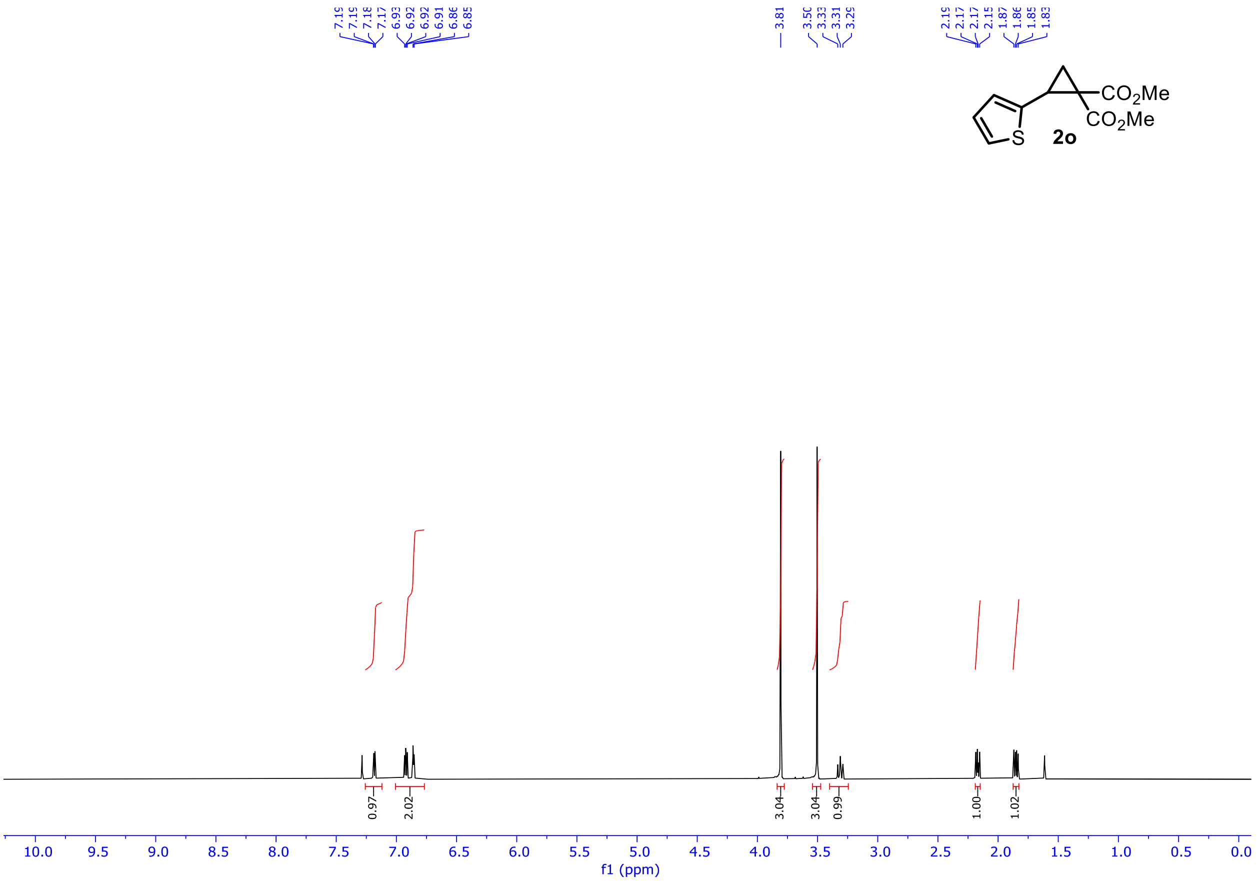

^{1}H NMR (400 MHz) spectra of **2o** ($CDCl_3$).

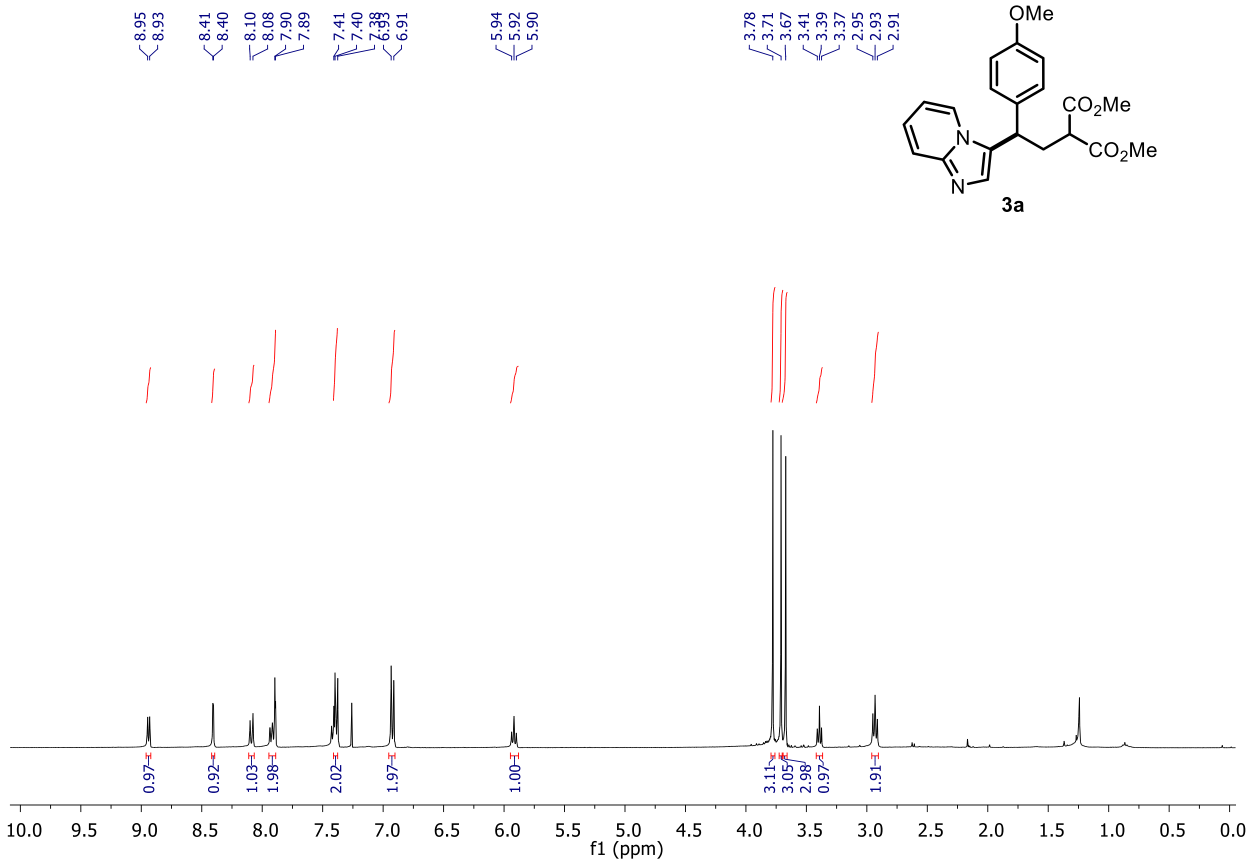

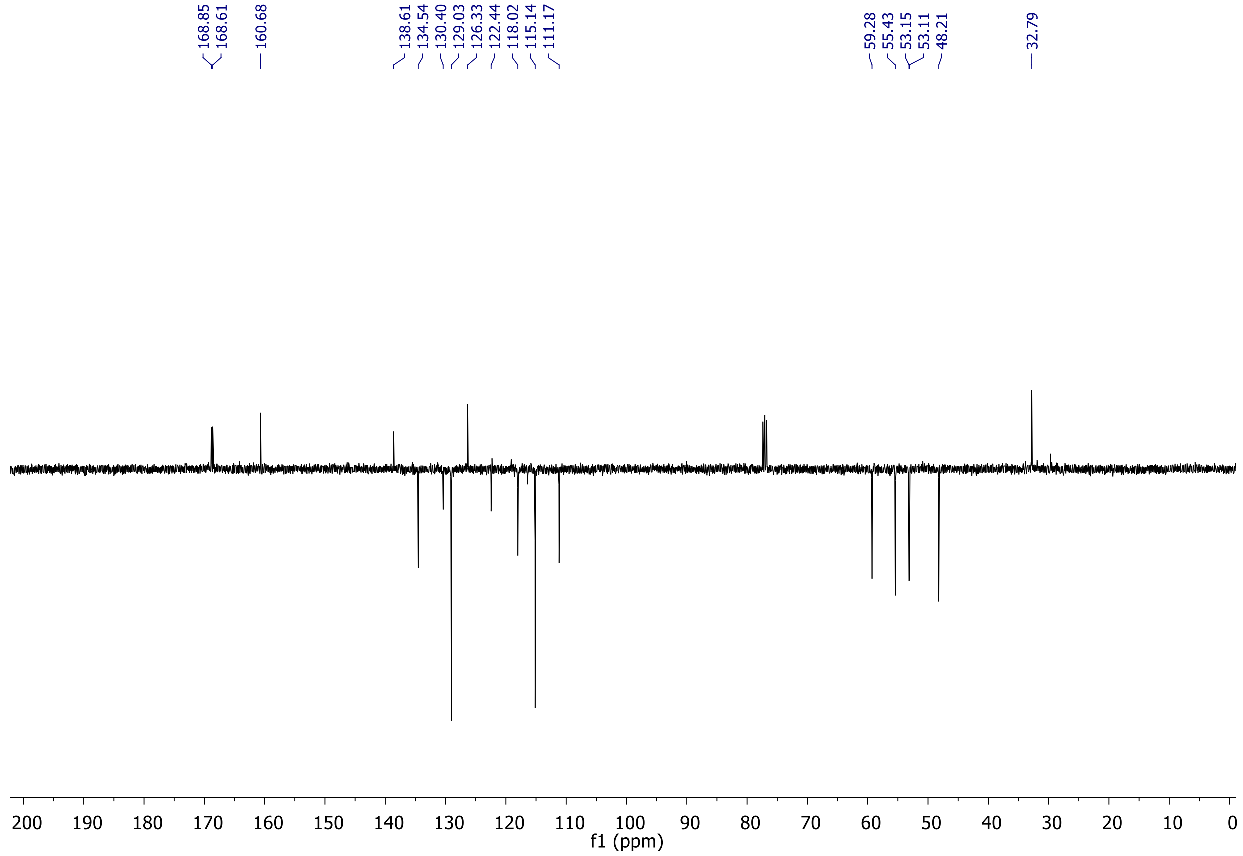

^{1}H NMR (400 MHz) and $^{13}C\{^{1}H\}$ APT NMR (100 MHz) spectra of **3a** ($CDCl_3$).

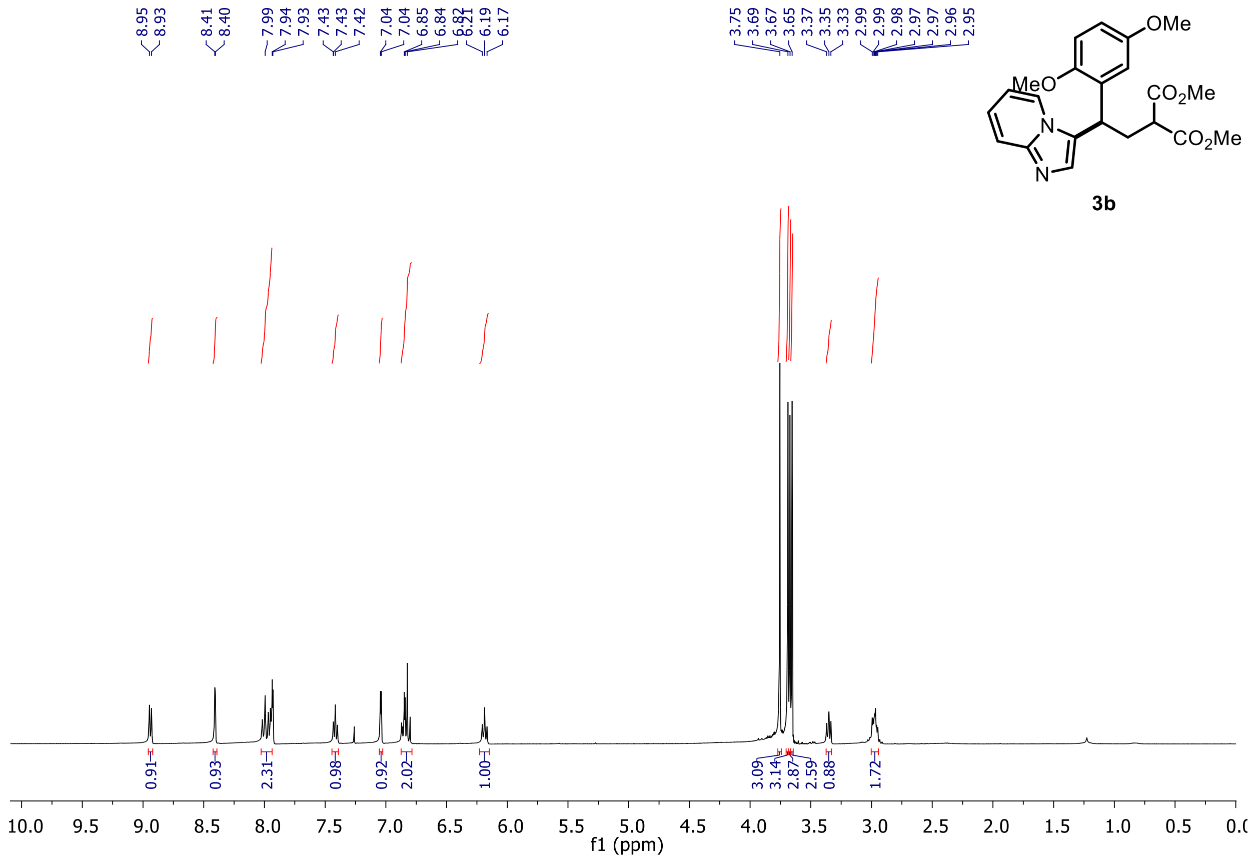

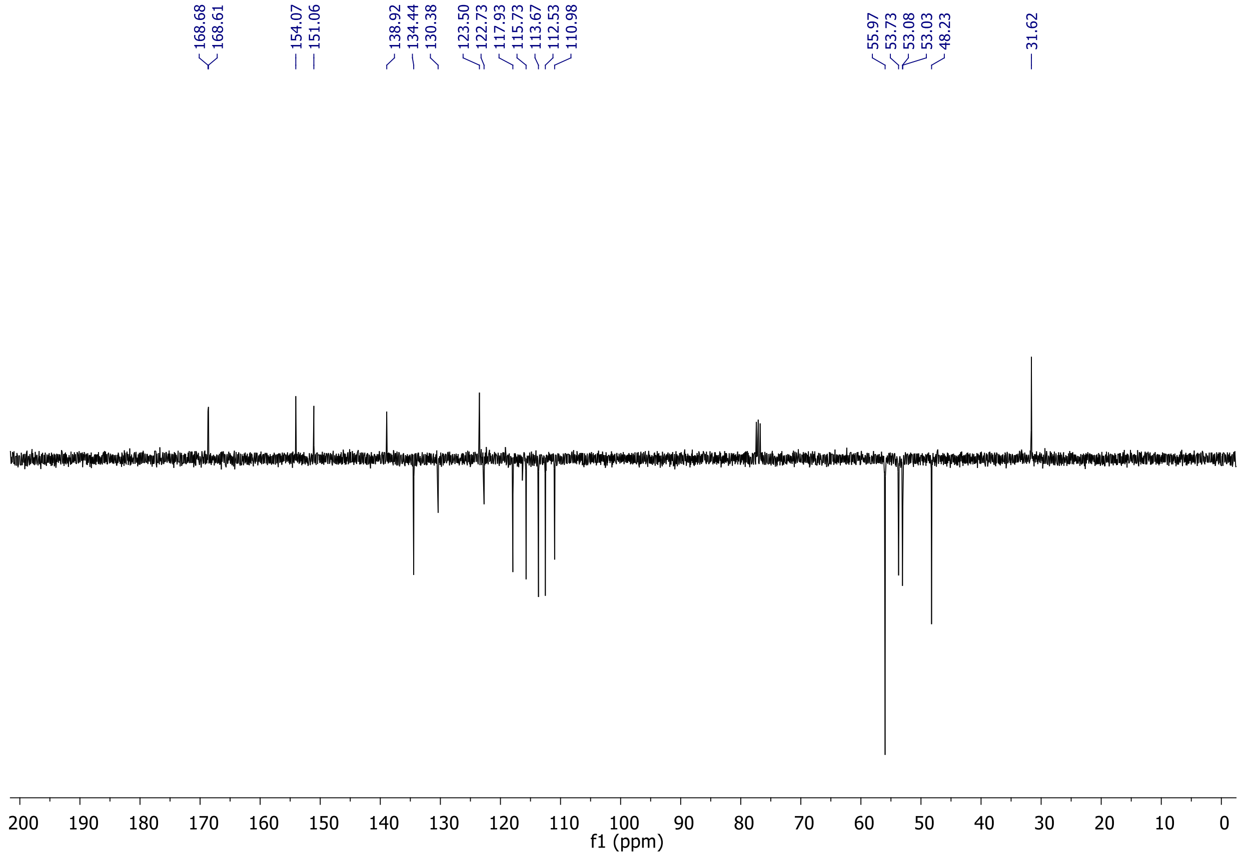

^1H NMR (400 MHz) and $^{13}C\{^1H\}$ APT NMR (100 MHz) spectra of **3b** ($CDCl_3$).

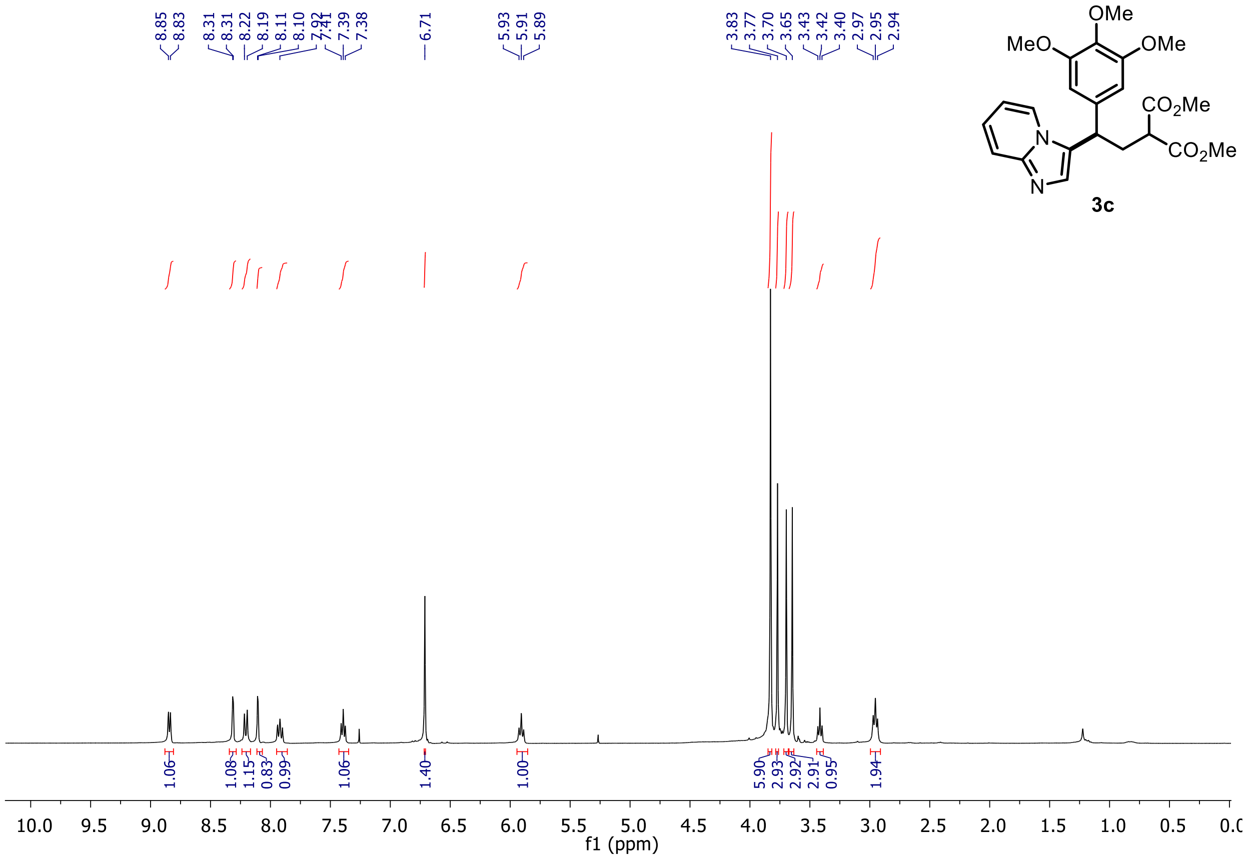

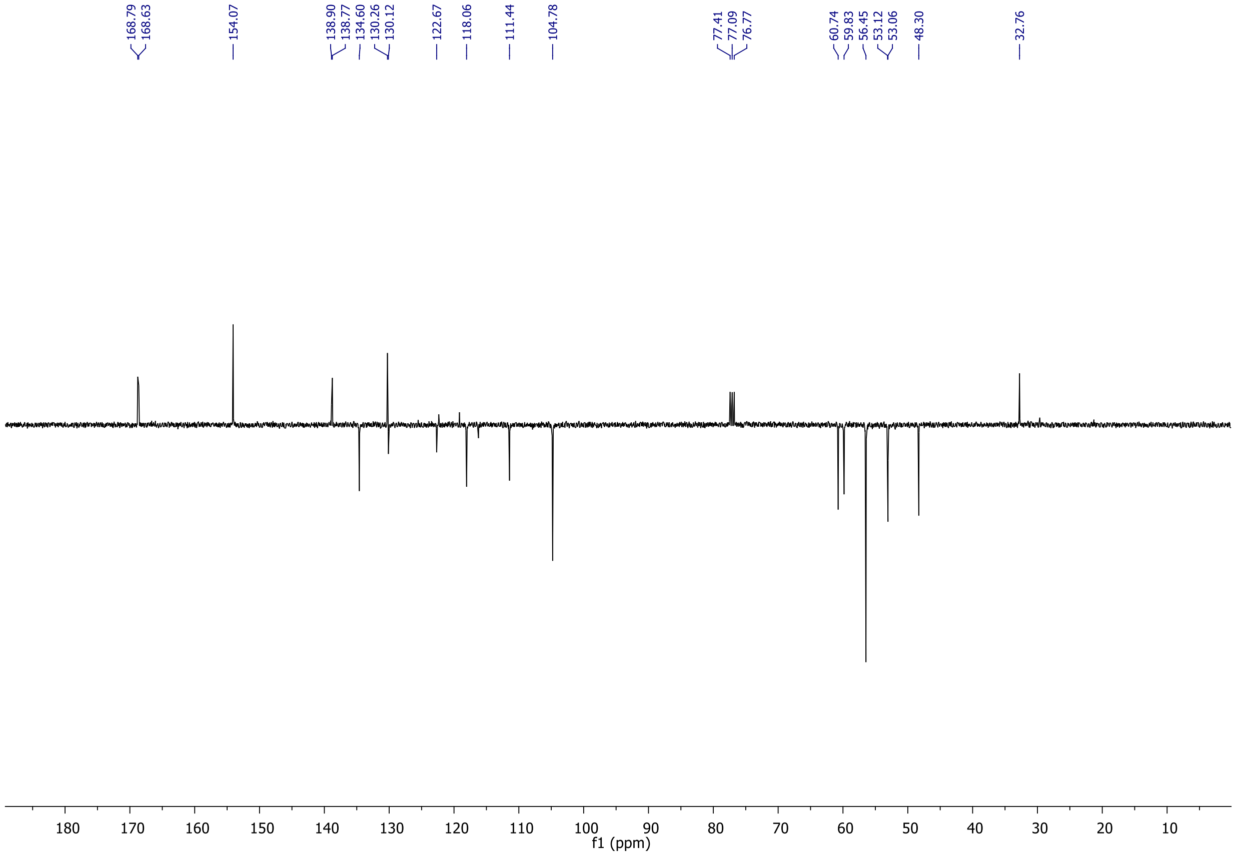

^{1}H NMR (400 MHz) and ^{13}C{^{1}H} APT NMR (100 MHz) spectra of **3c** ($CDCl_3$).

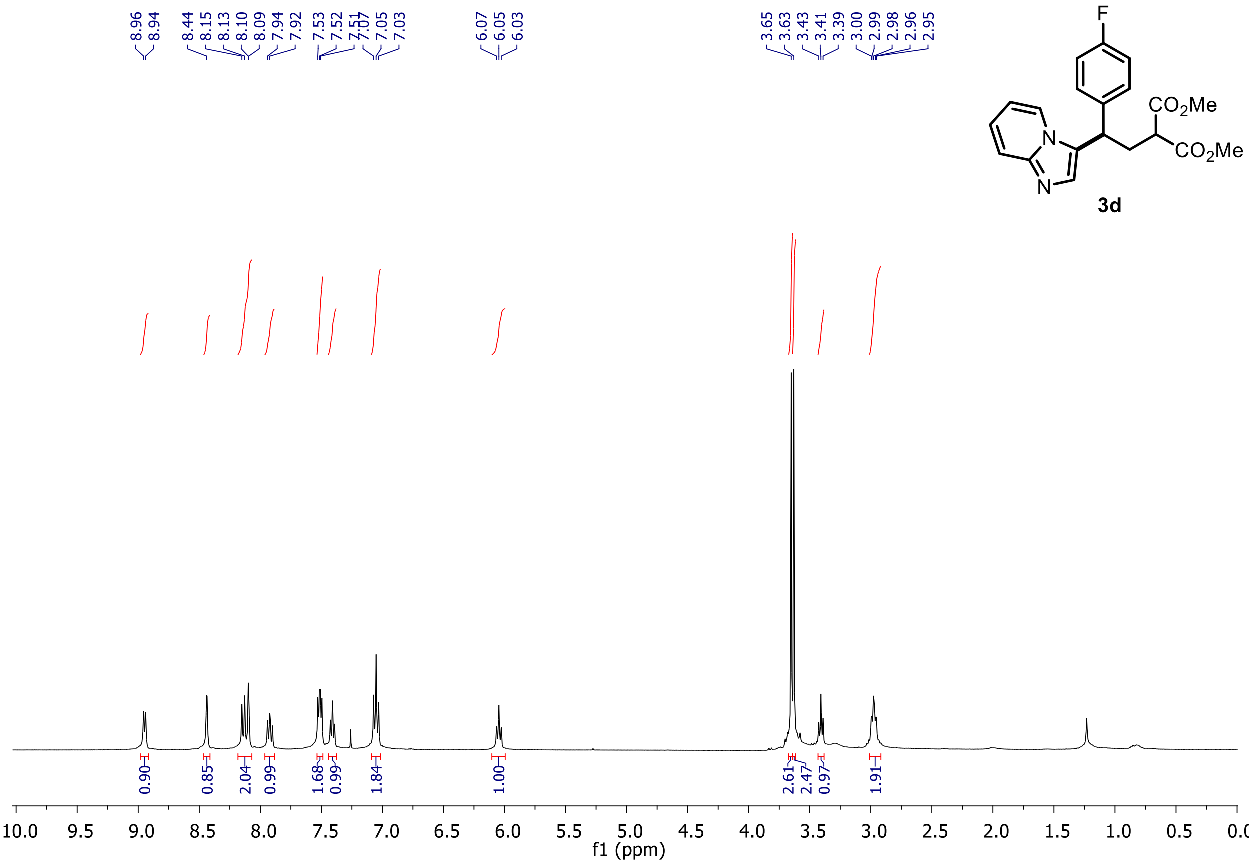

3d

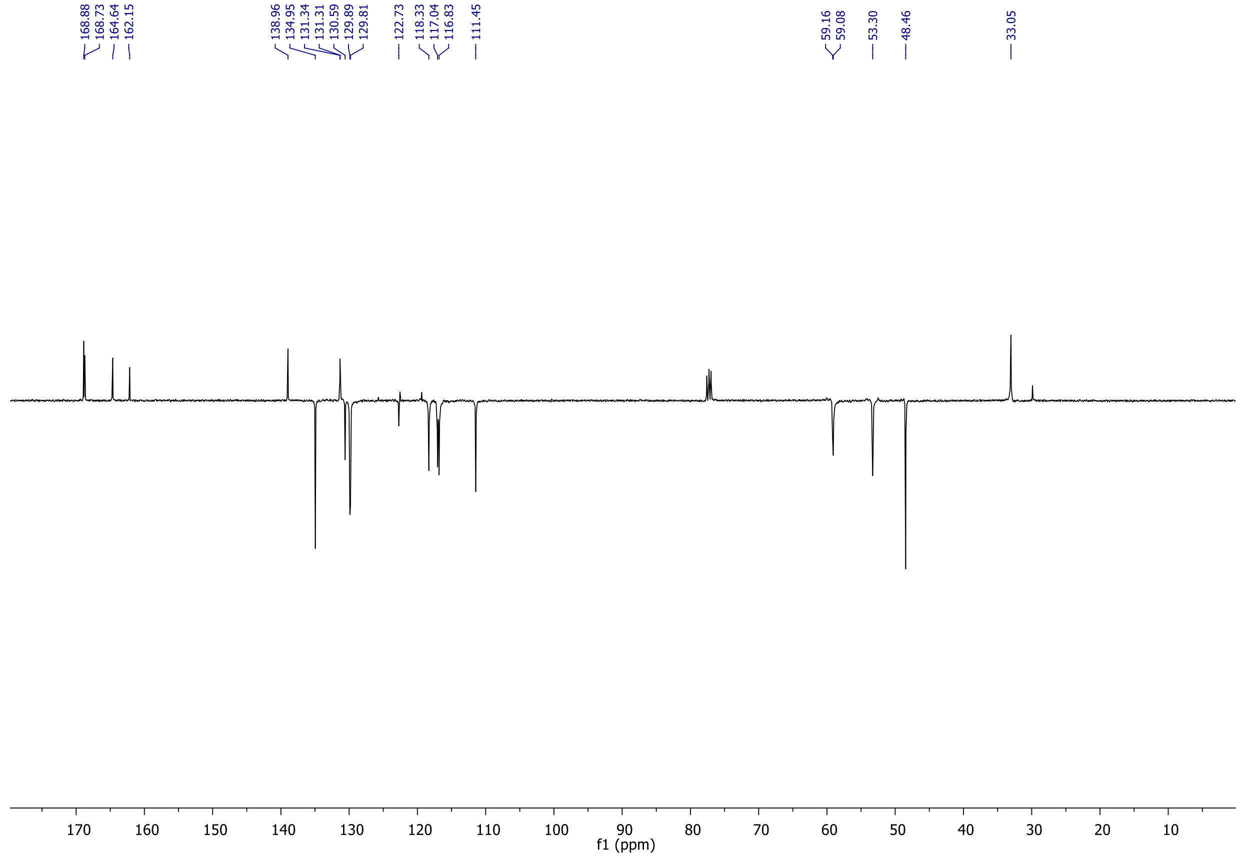

^{1}H NMR (400 MHz) and ^{13}C{^{1}H} APT NMR (100 MHz) spectra of **3d** ($CDCl_3$).

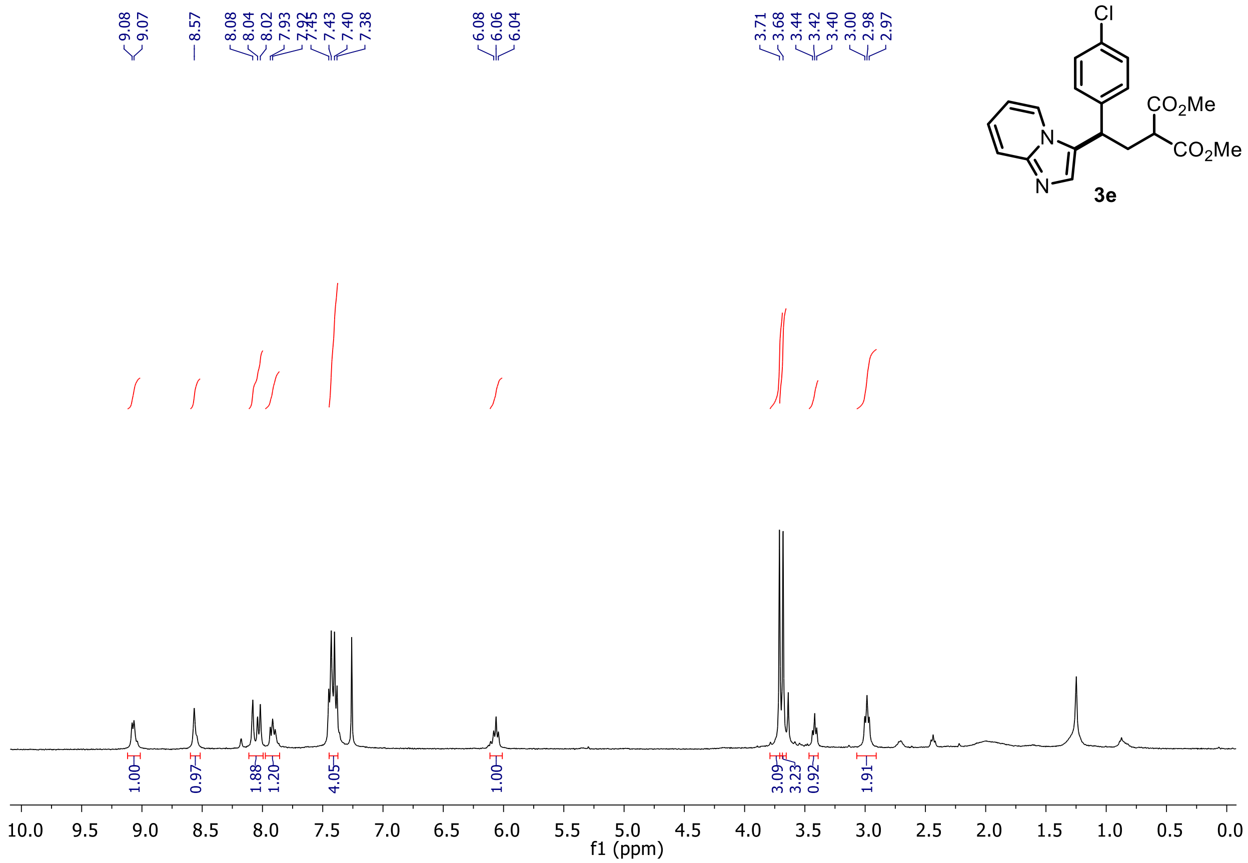

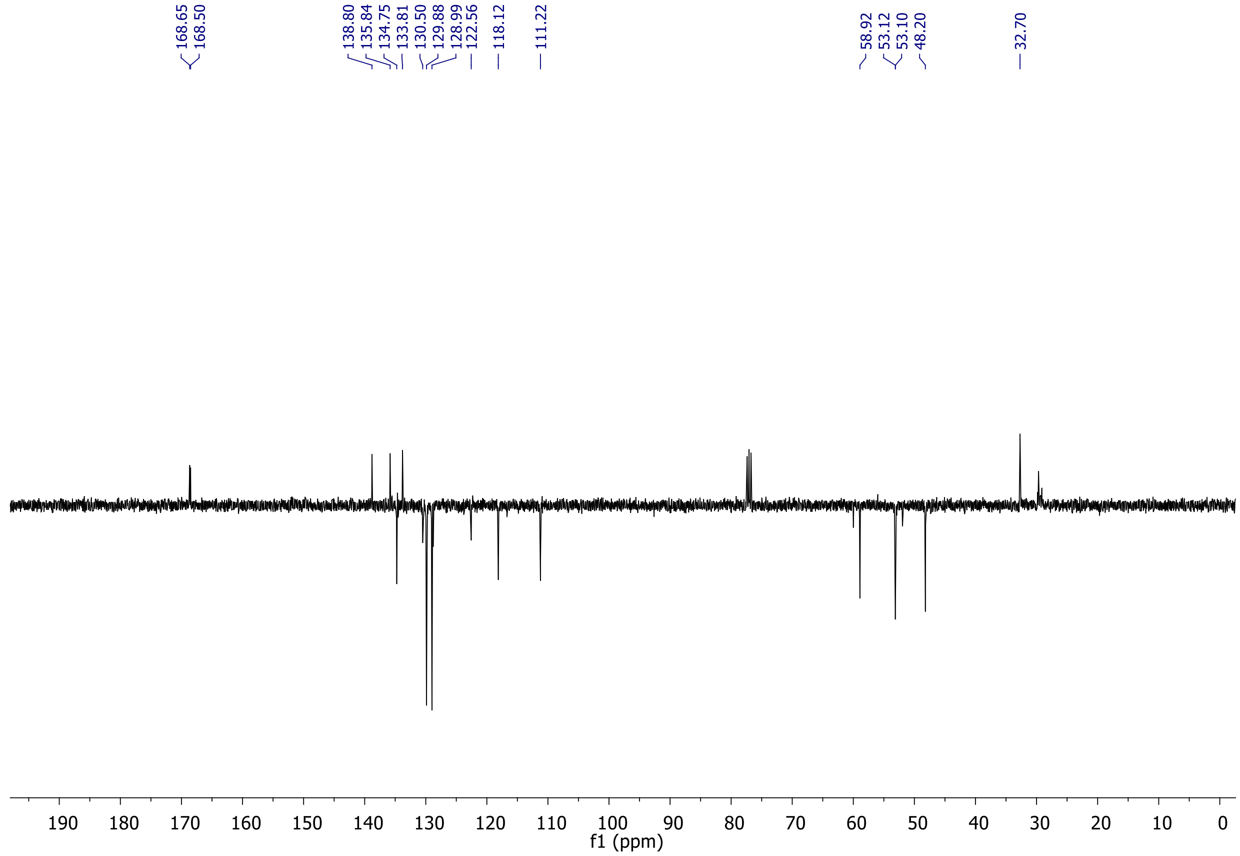

^{1}H NMR (400 MHz) and ^{13}C{^{1}H} APT NMR (100 MHz) spectra of **3e** ($CDCl_3$).

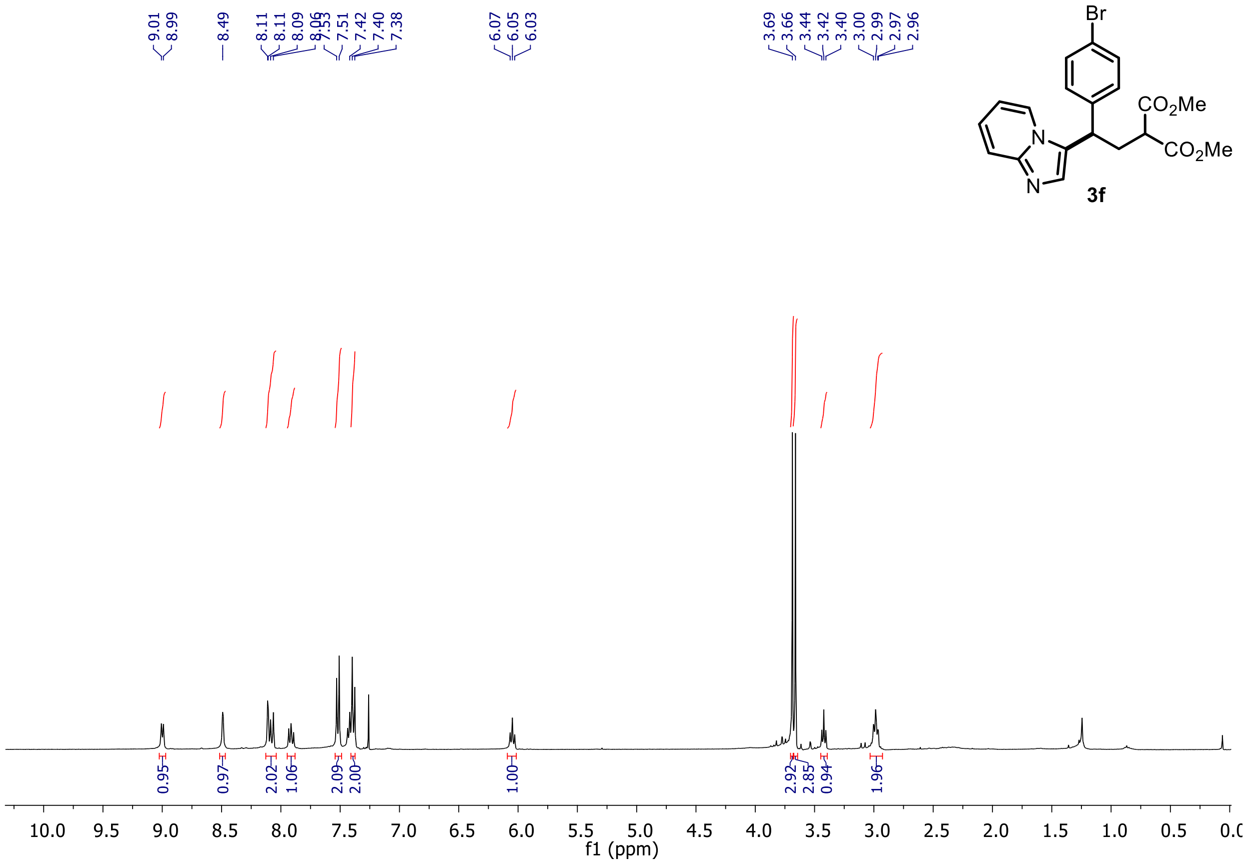

3f

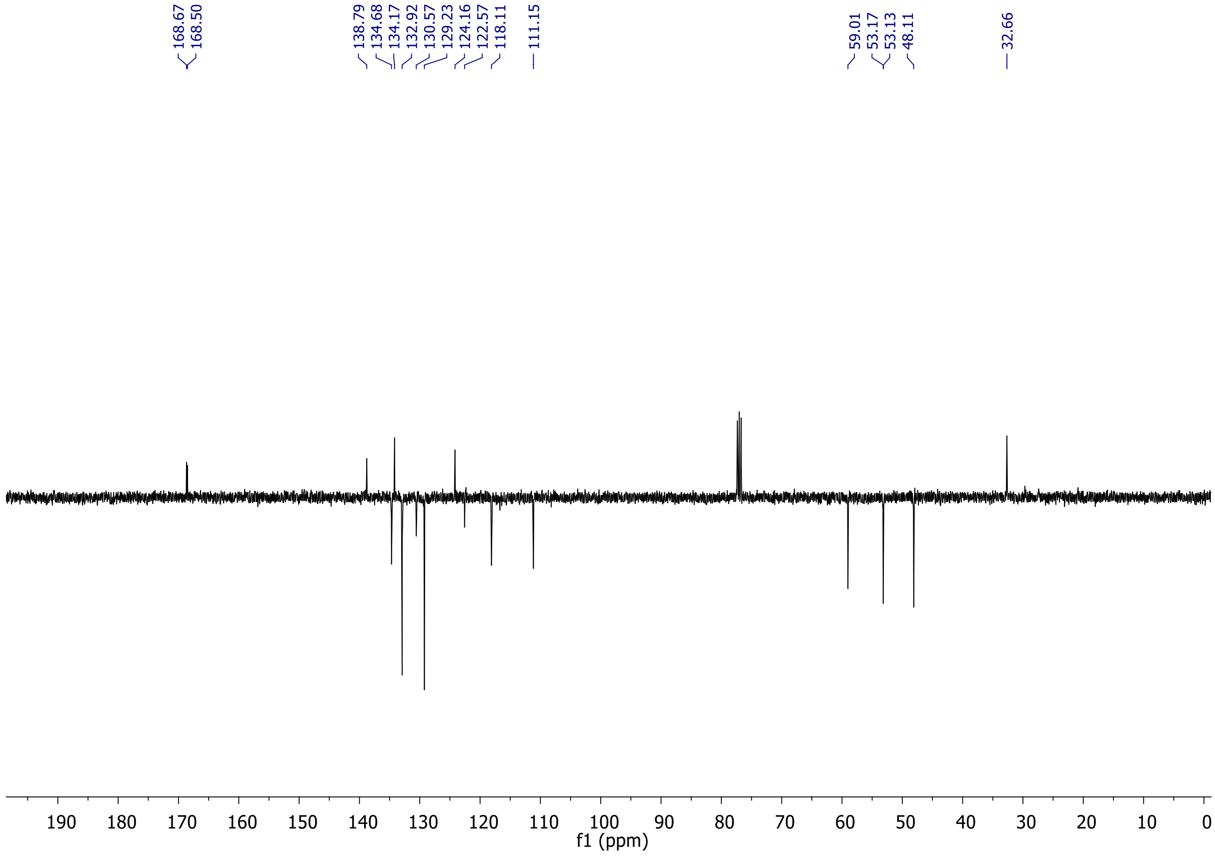

^{1}H NMR (400 MHz) and $^{13}C\{^{1}H\}$ APT NMR (100 MHz) spectra of **3f** ($CDCl_3$).

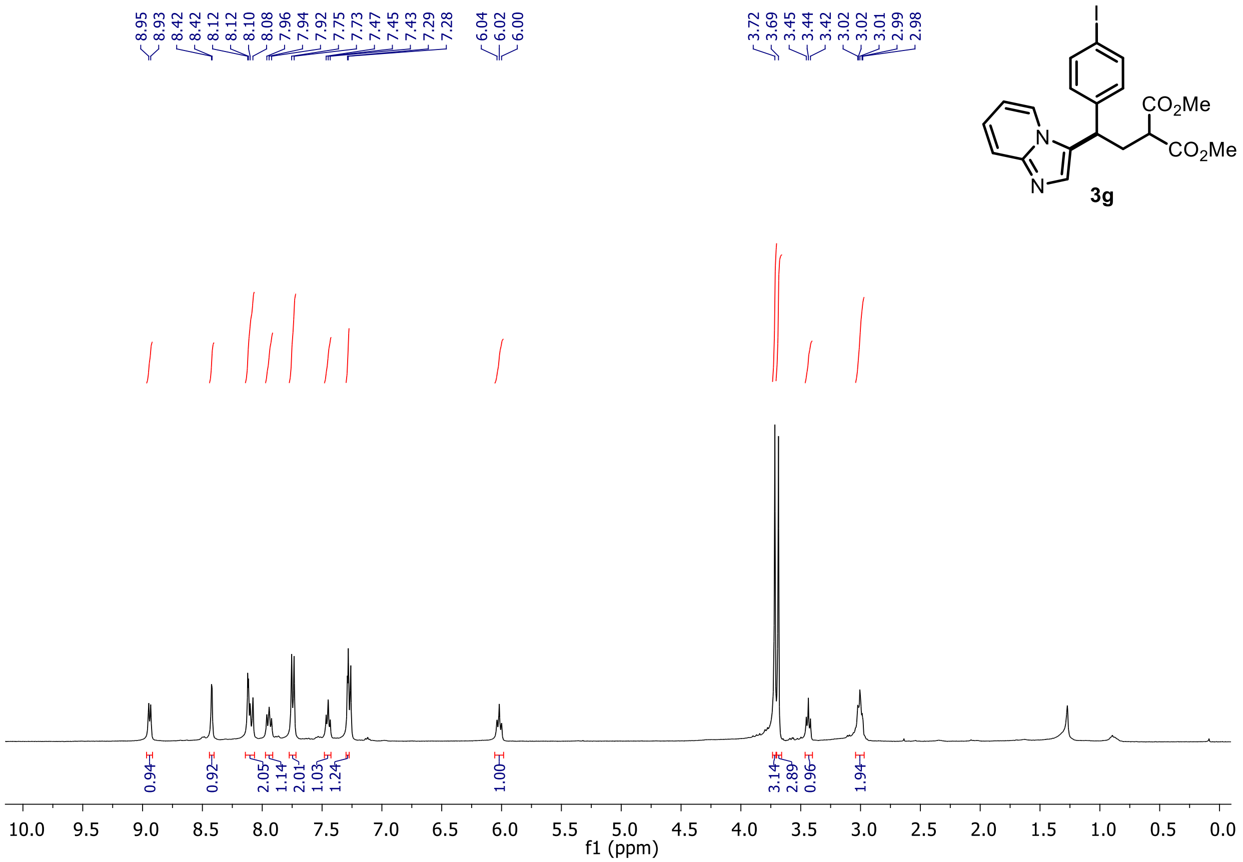

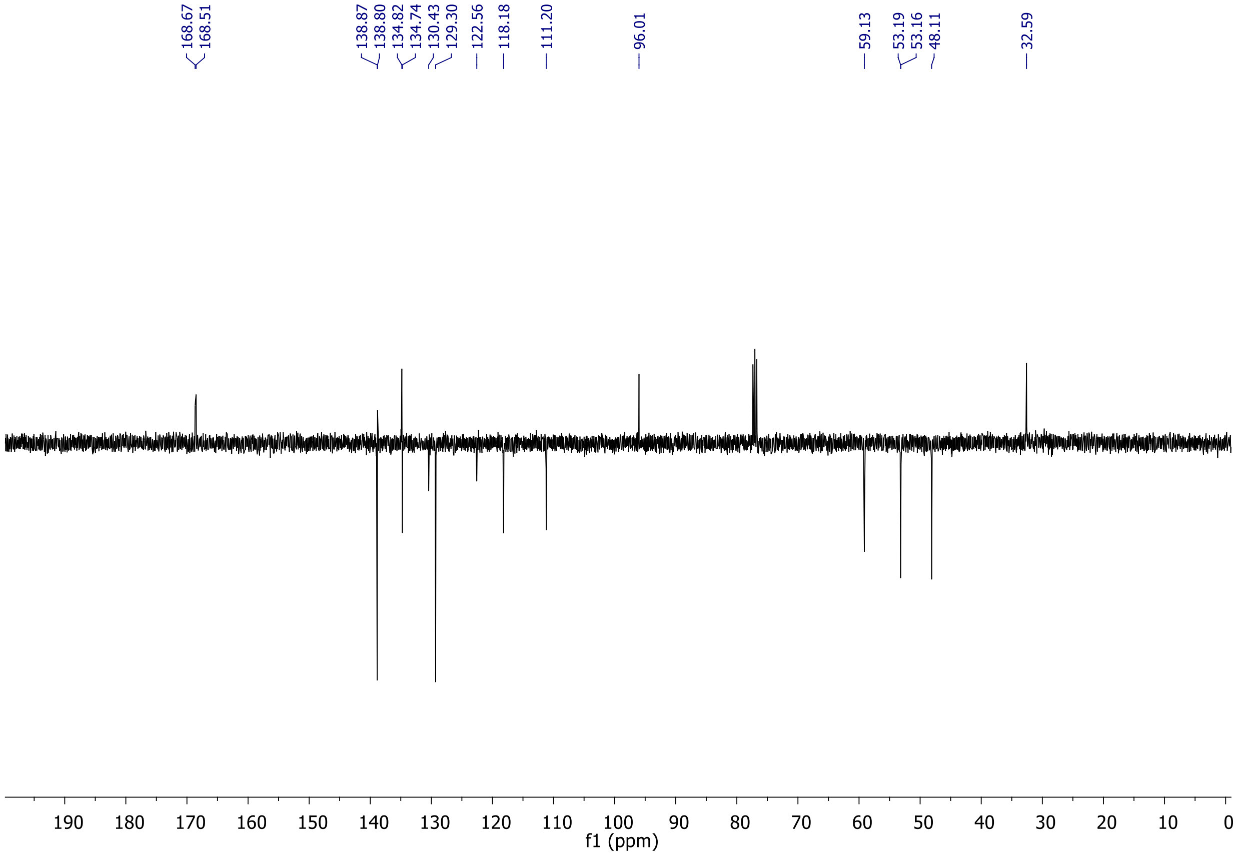

^{1}H NMR (400 MHz) and ^{13}C{^{1}H} APT NMR (100 MHz) spectra of **3g** ($CDCl_3$).

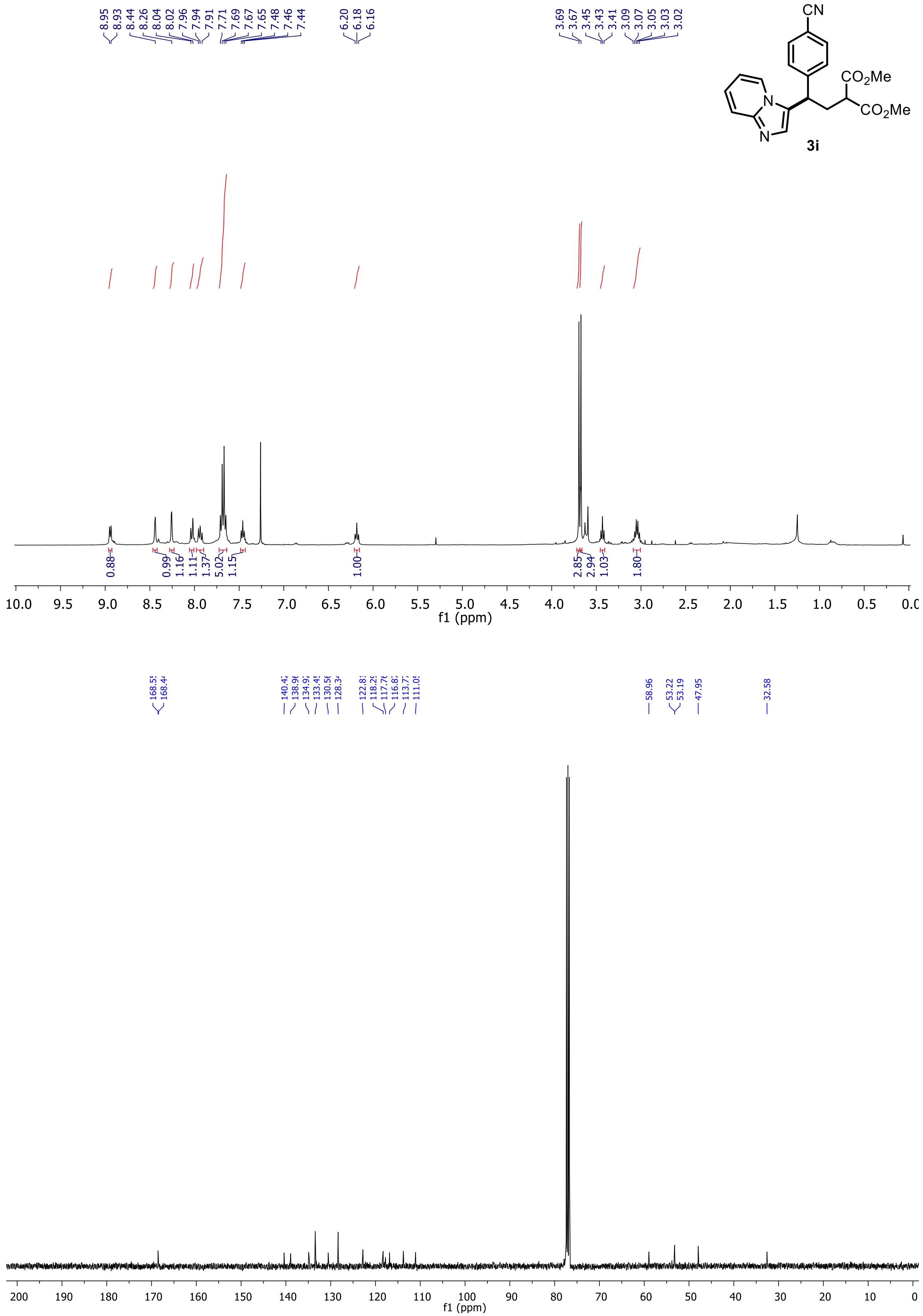

^{1}H NMR (400 MHz) and $^{13}C\{^{1}H\}$ NMR (100 MHz) spectra of **3i** ($CDCl_3$).

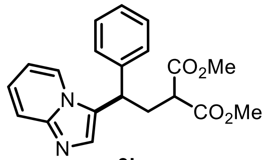

3j

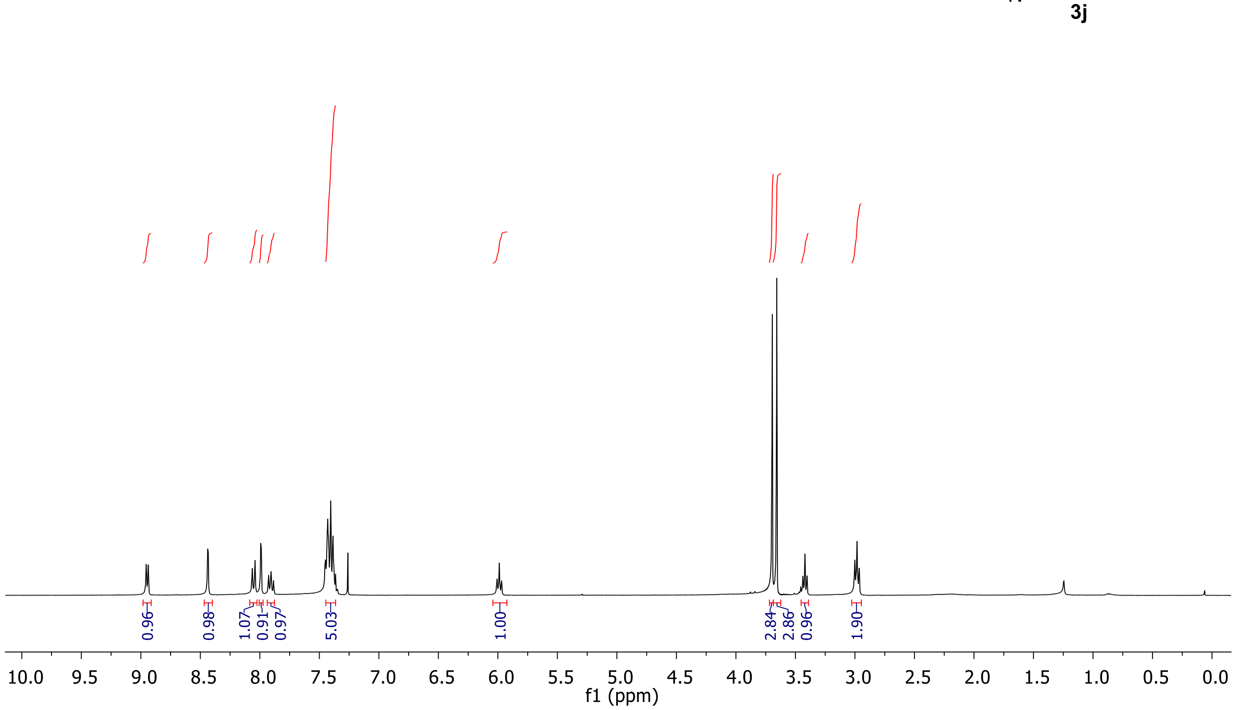

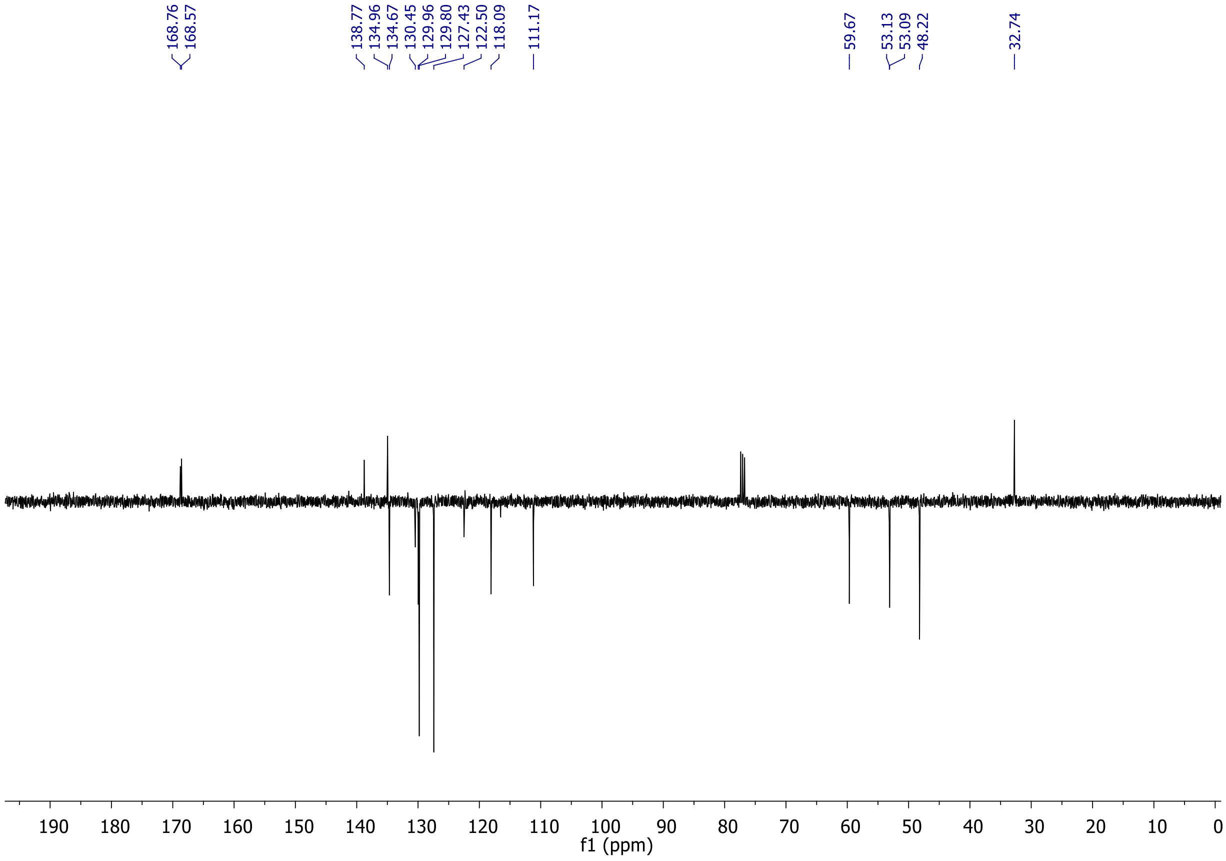

^{1}H NMR (400 MHz) and ^{13}C{^{1}H} APT NMR (100 MHz) spectra of **3j** ($CDCl_3$).

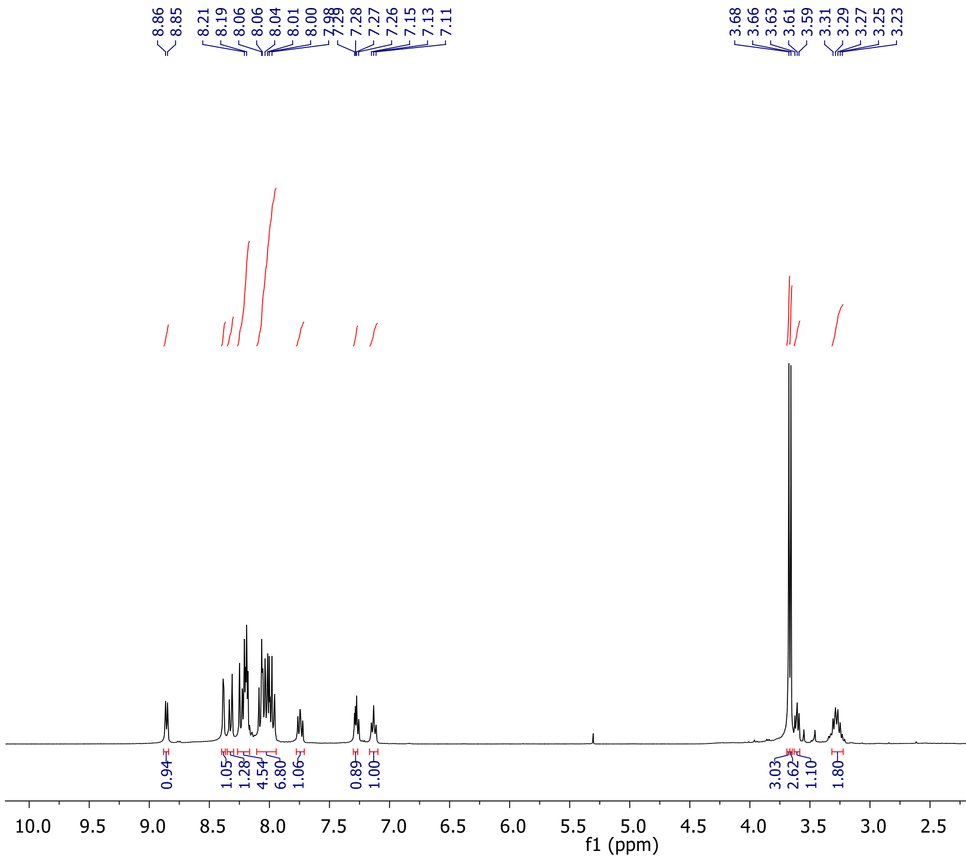

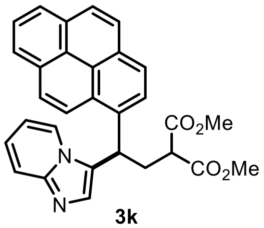

3k

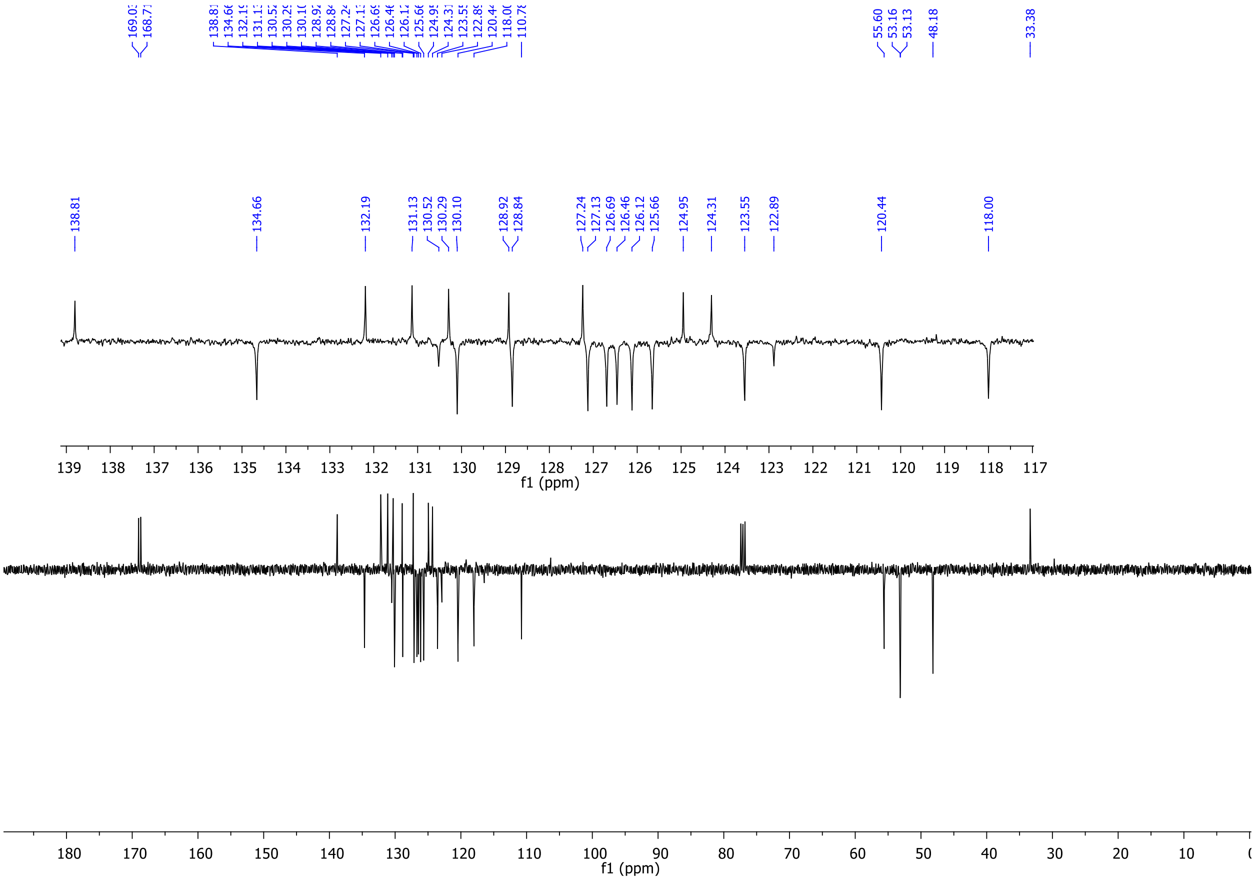

^{1}H NMR (400 MHz) and ^{13}C{^{1}H} APT NMR (100 MHz) spectra of **3k** ($CDCl_3$).

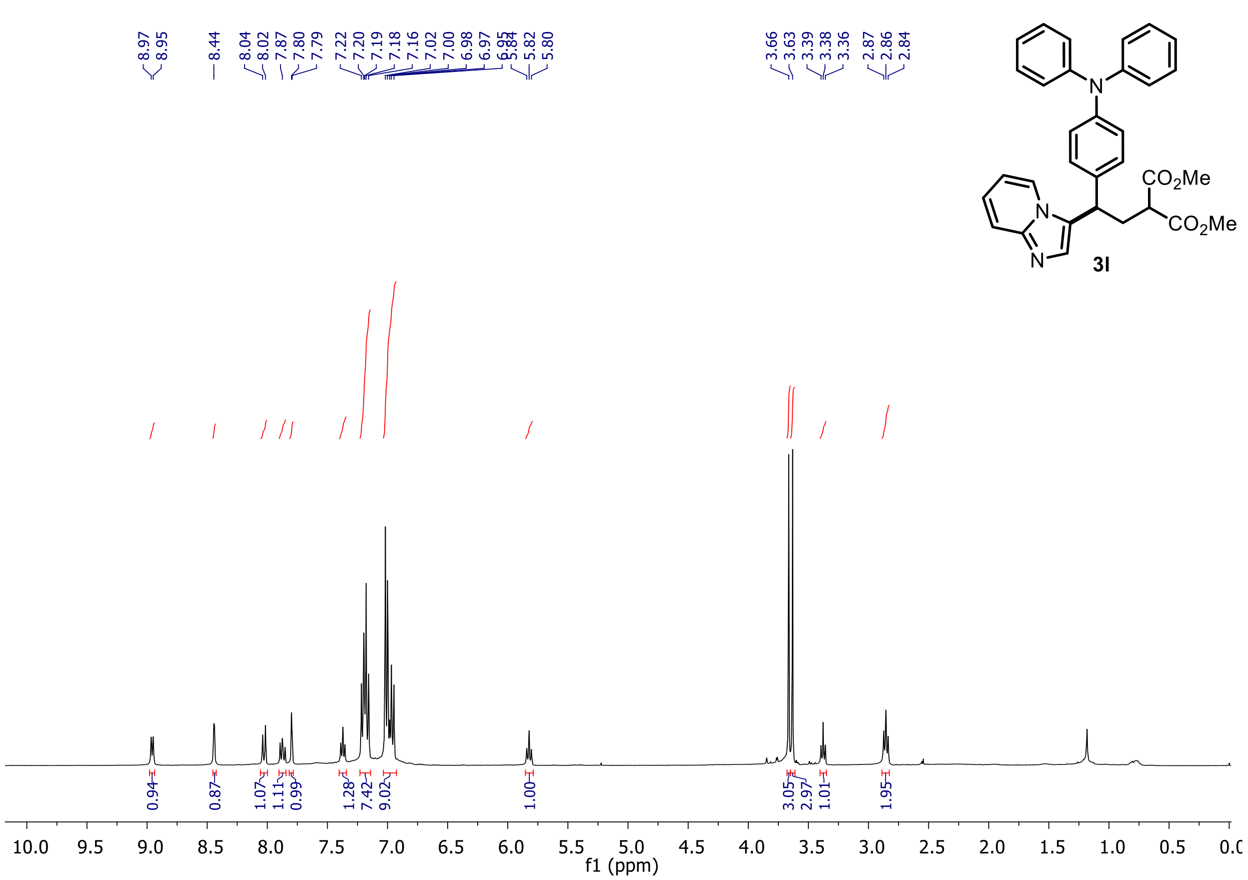

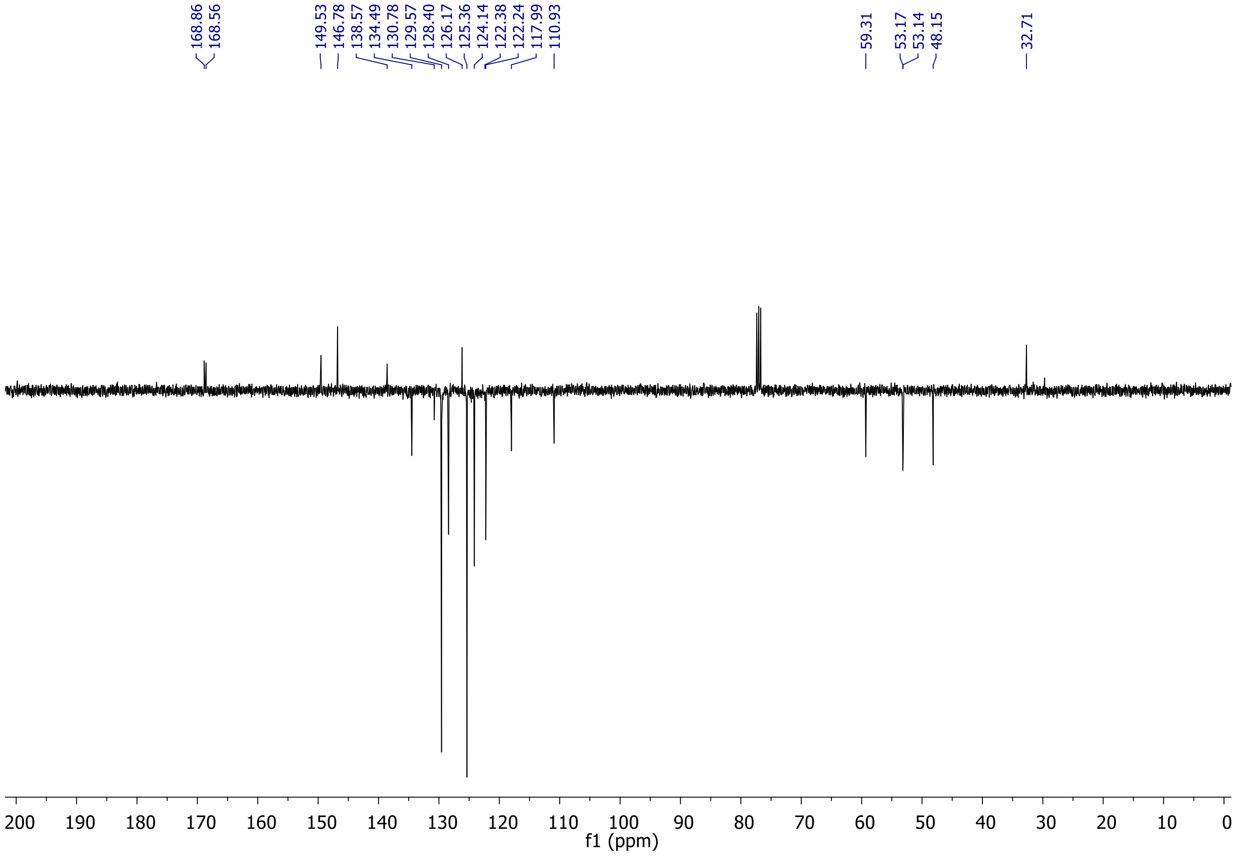

^{1}H NMR (400 MHz) and $^{13}C\{^{1}H\}$ APT NMR (100 MHz) spectra of **3l** ($CDCl_3$).

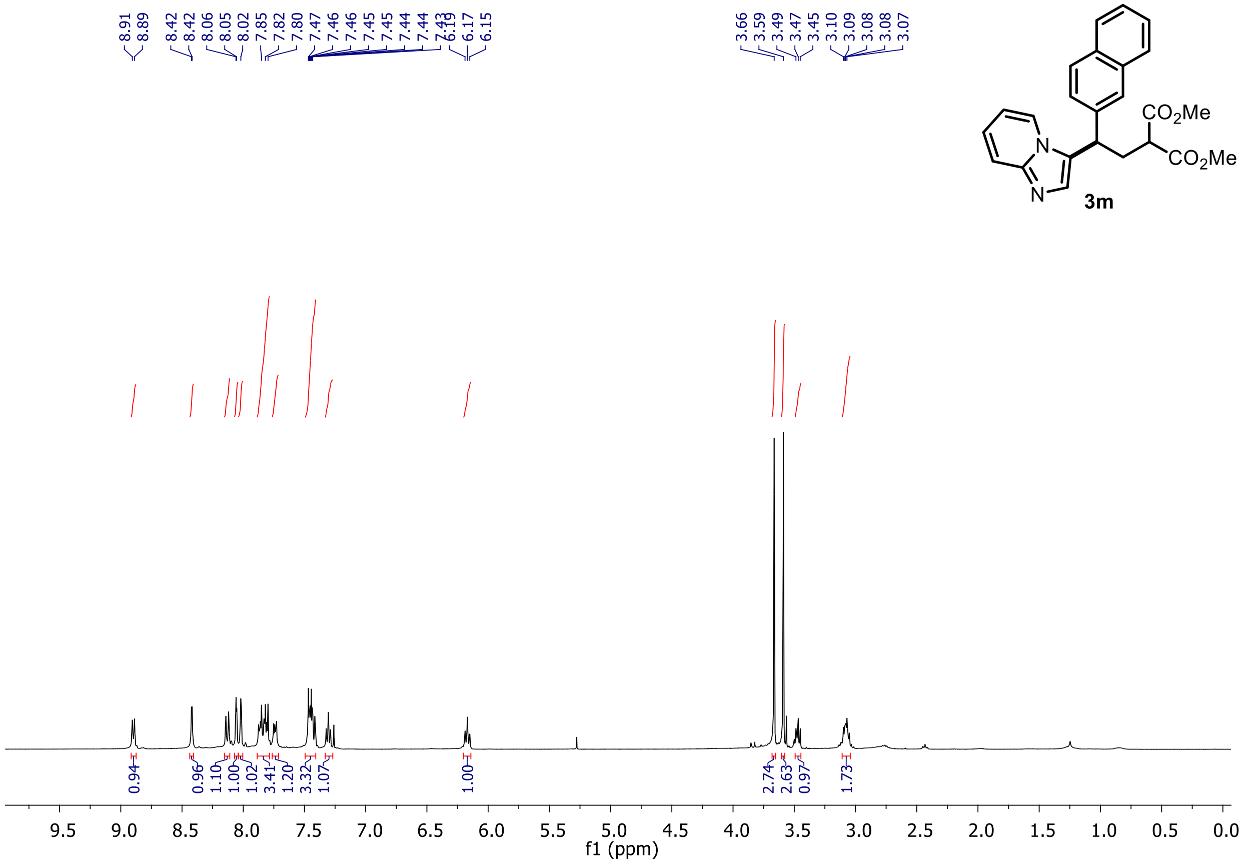

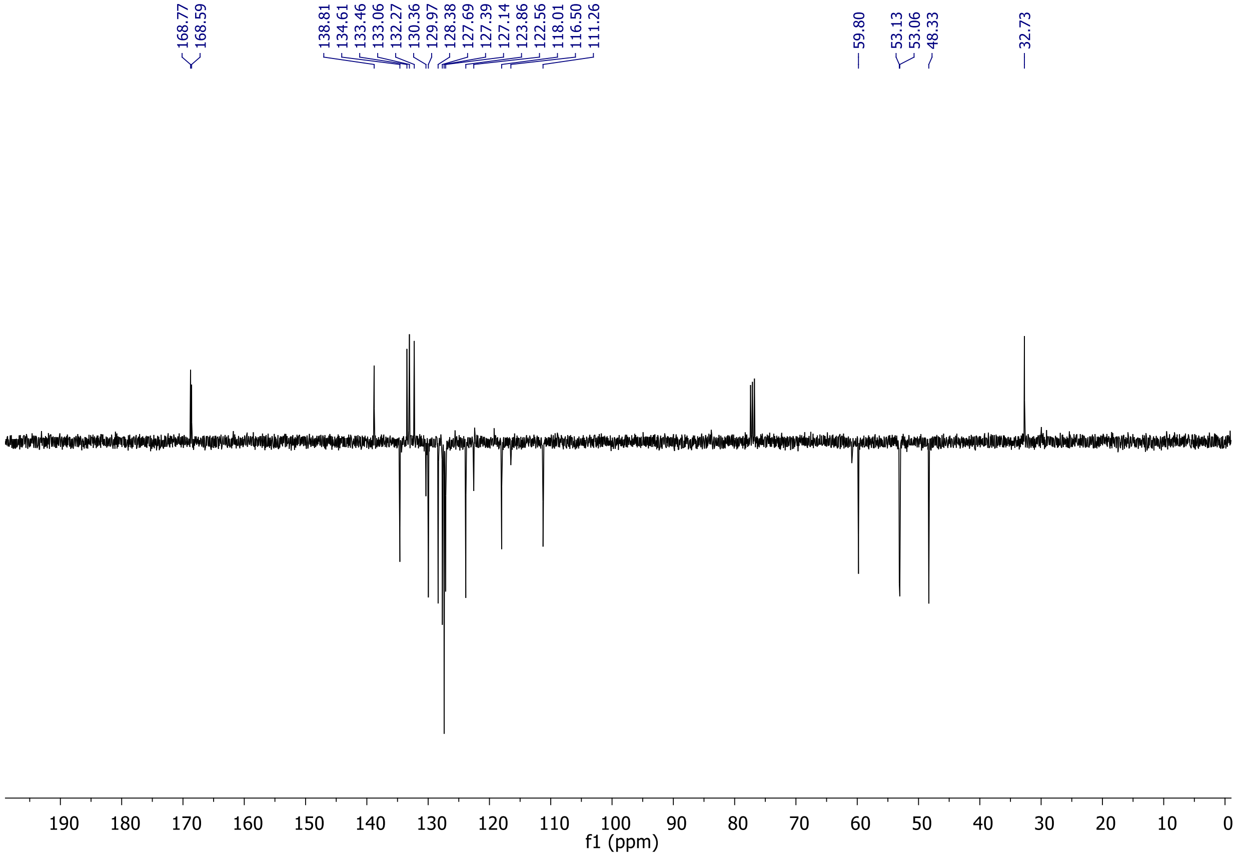

^1H NMR (400 MHz) and $^{13}C\{^1H\}$ APT NMR (100 MHz) spectra of **3m** ($CDCl_3$).

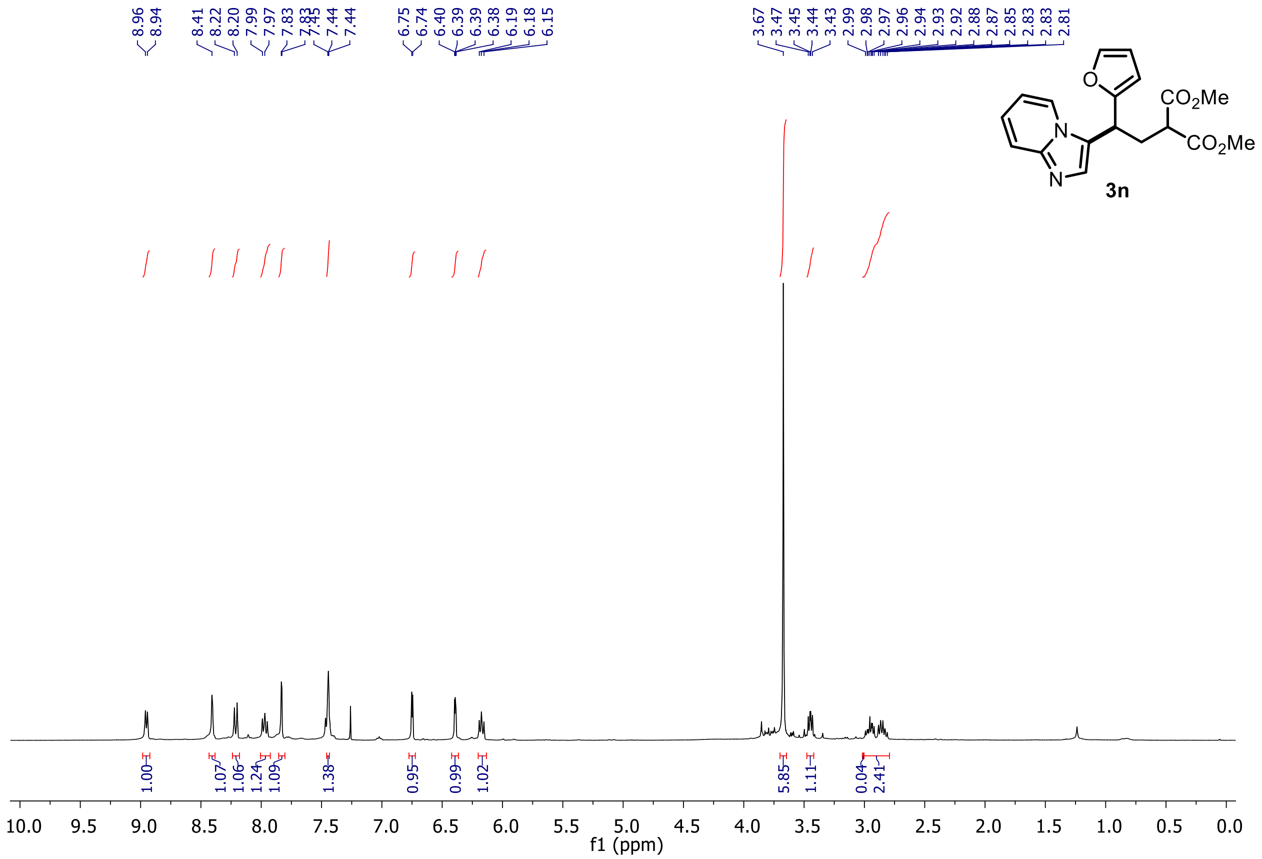

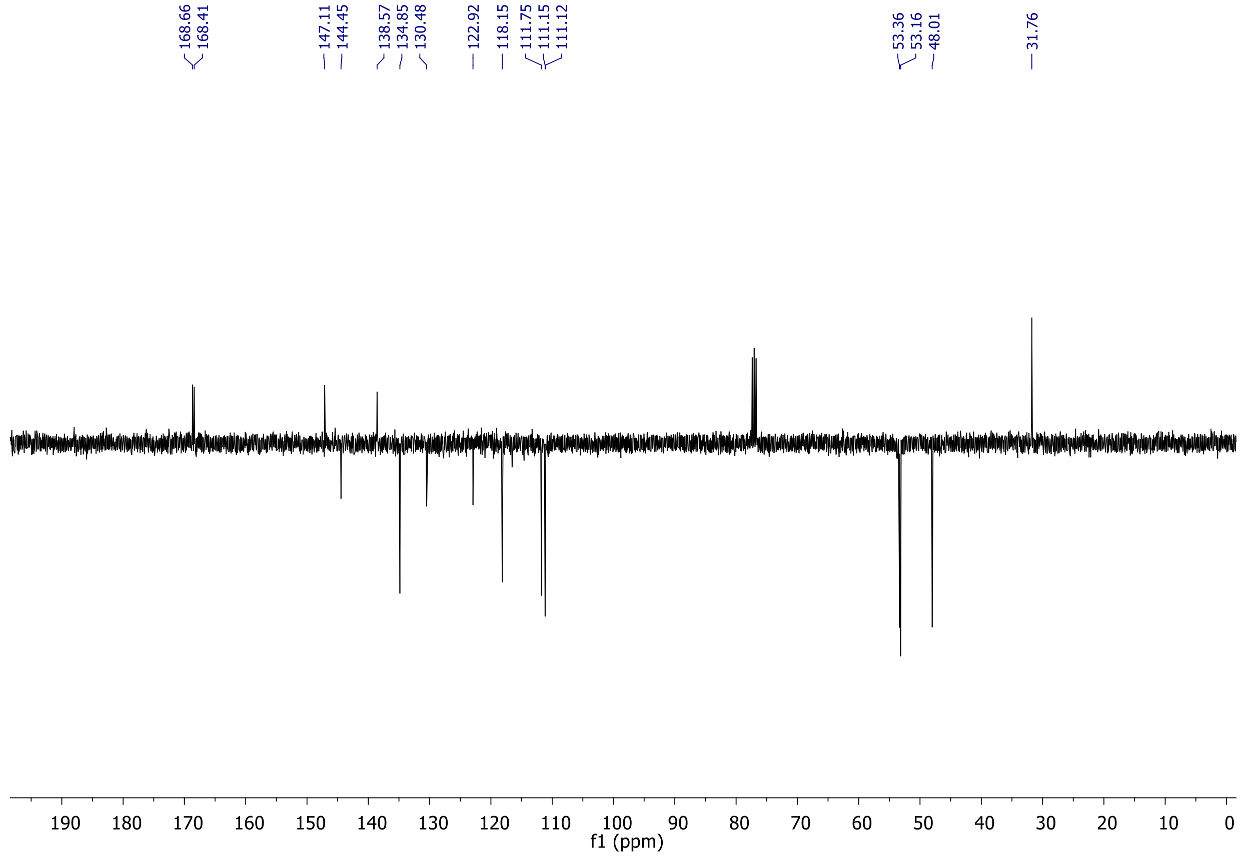

^{1}H NMR (400 MHz) and $^{13}C\{^{1}H\}$ APT NMR (100 MHz) spectra of **3n** ($CDCl_3$).

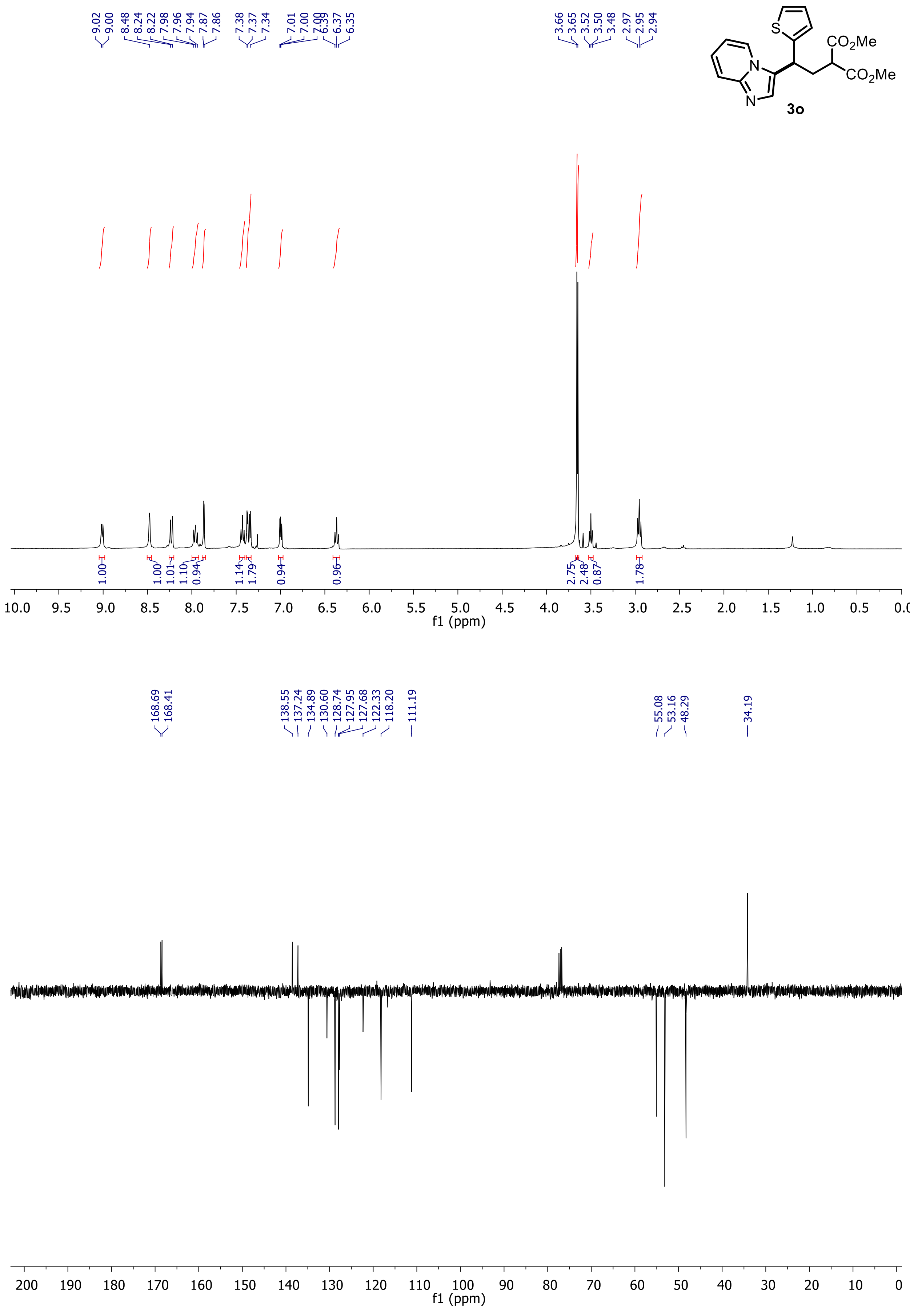

^{1}H NMR (400 MHz) and $^{13}C\{^{1}H\}$ APT NMR (100 MHz) spectra of **3o** ($CDCl_3$).

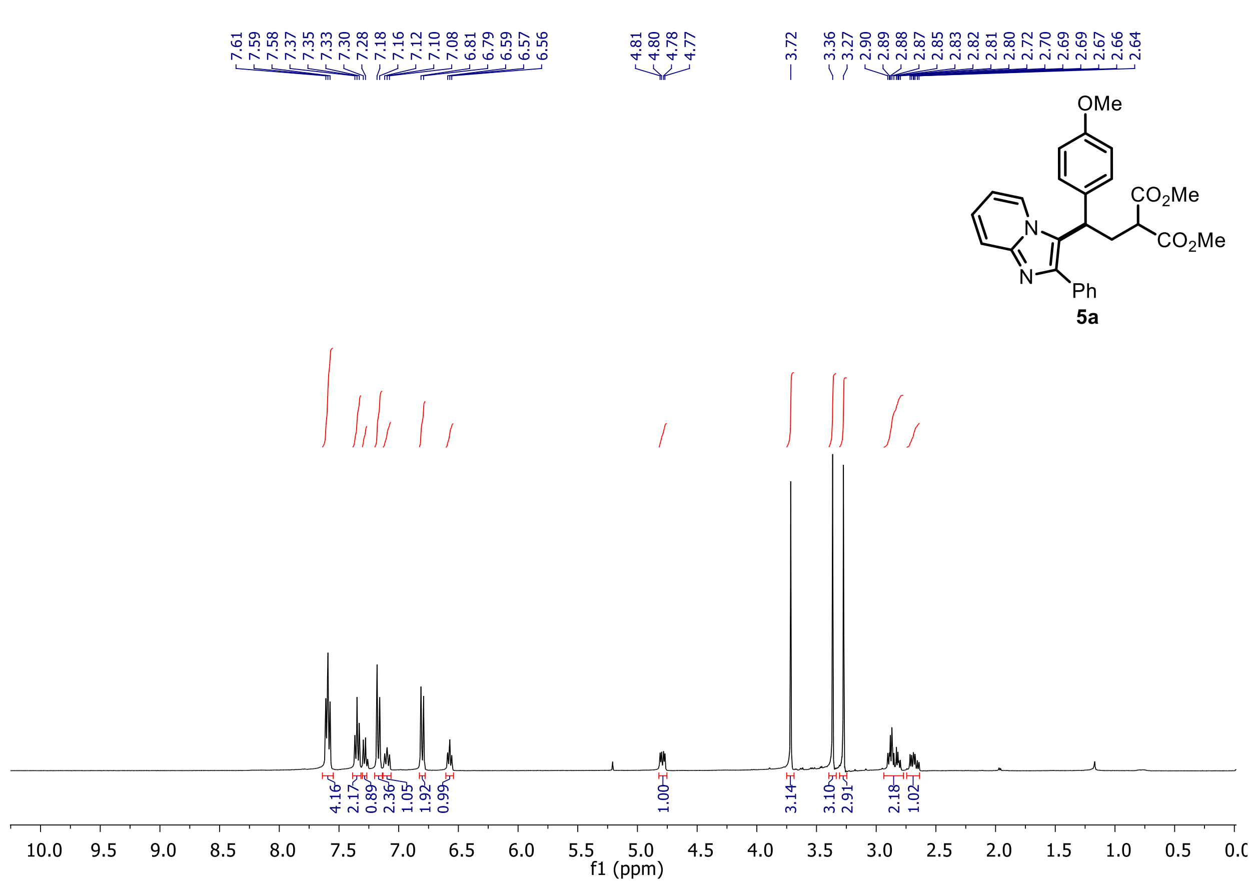

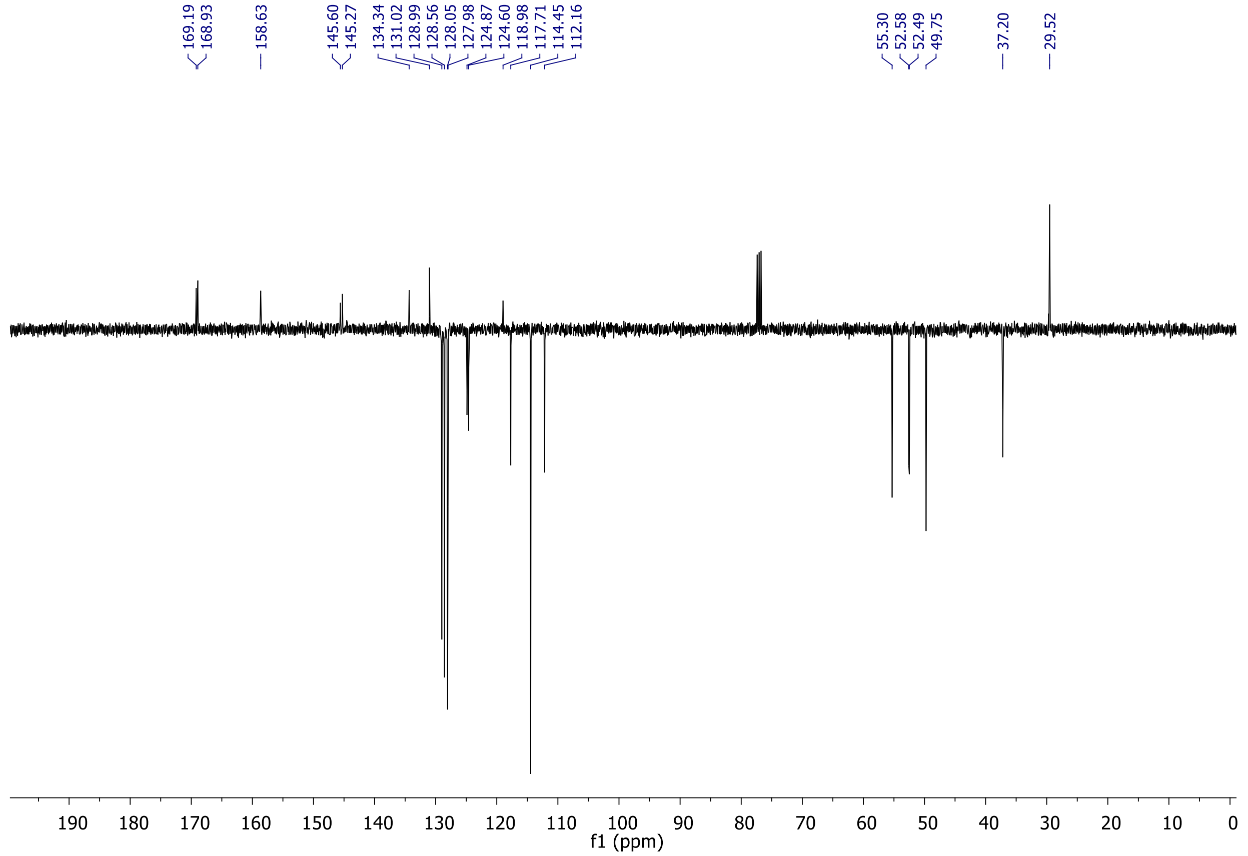

^{1}H NMR (400 MHz) and ^{13}C{^{1}H} APT NMR (100 MHz) spectra of **5a** ($CDCl_3$).

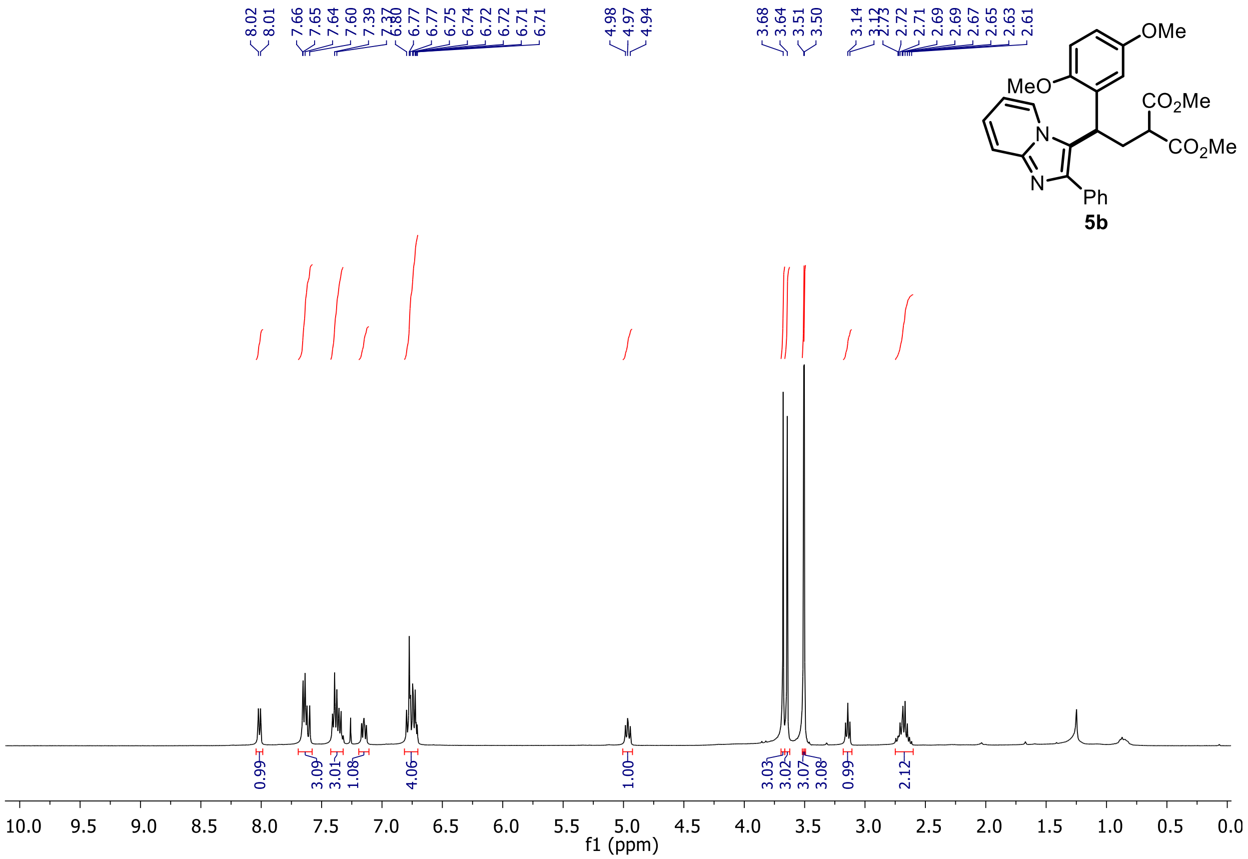

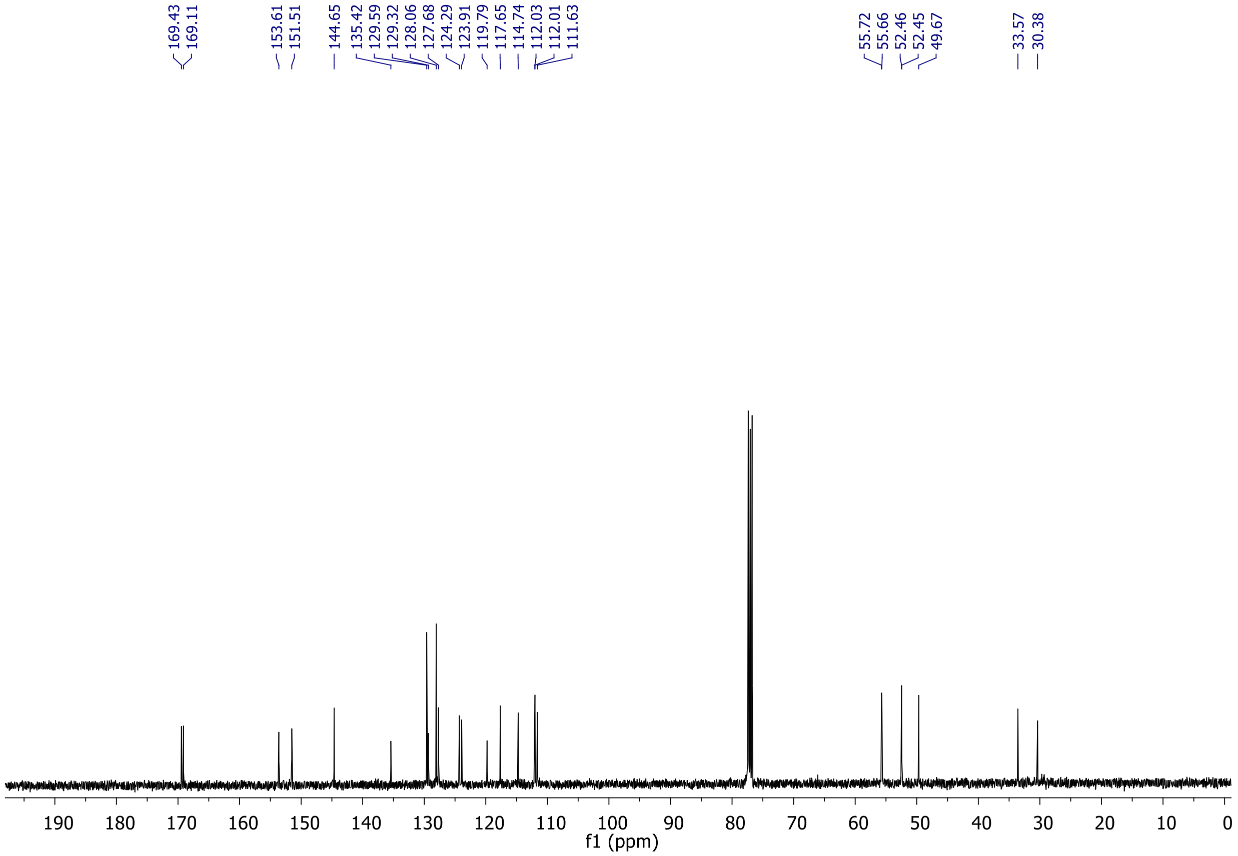

^{1}H NMR (400 MHz) and ^{13}C{^{1}H} NMR (100 MHz) spectra of **5b** ($CDCl_3$).

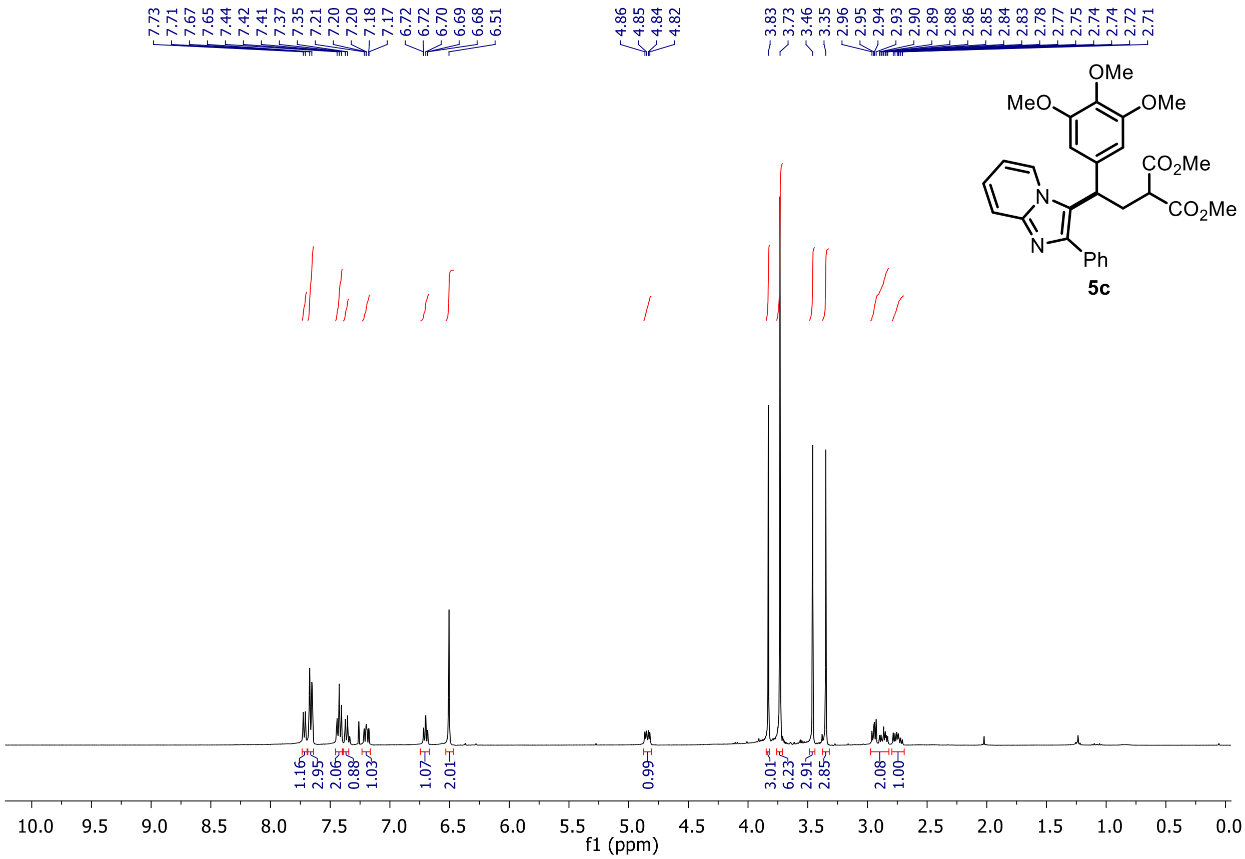

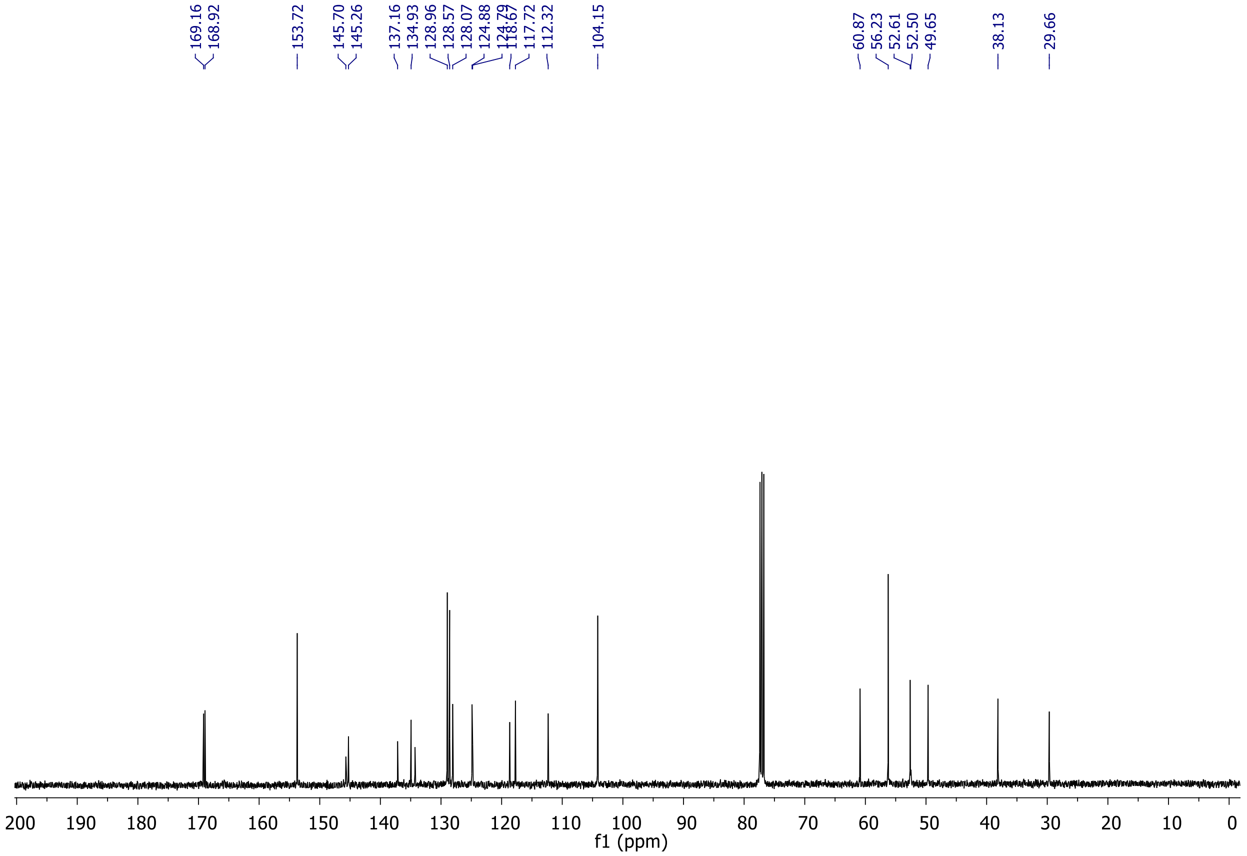

^{1}H NMR (400 MHz) and ^{13}C{^{1}H} NMR (100 MHz) spectra of **5c** ($CDCl_3$).

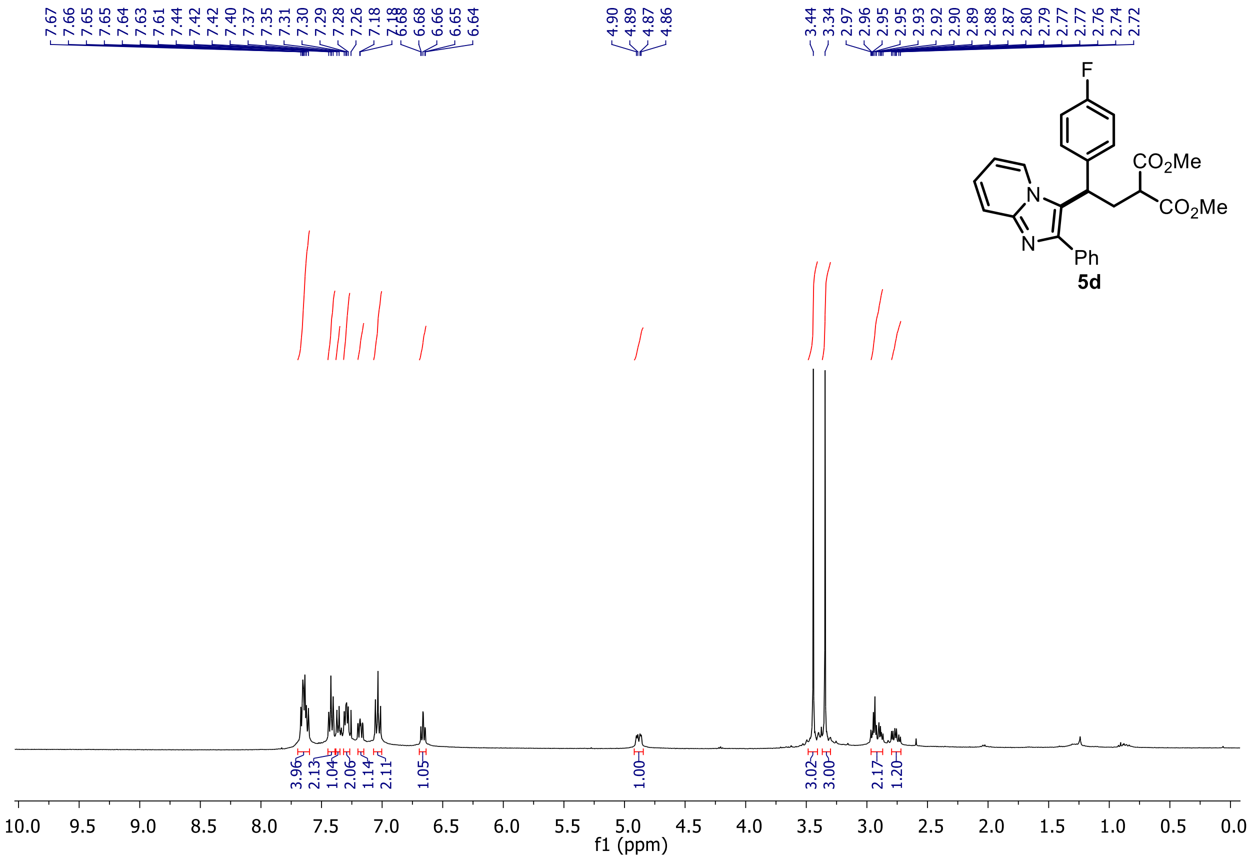

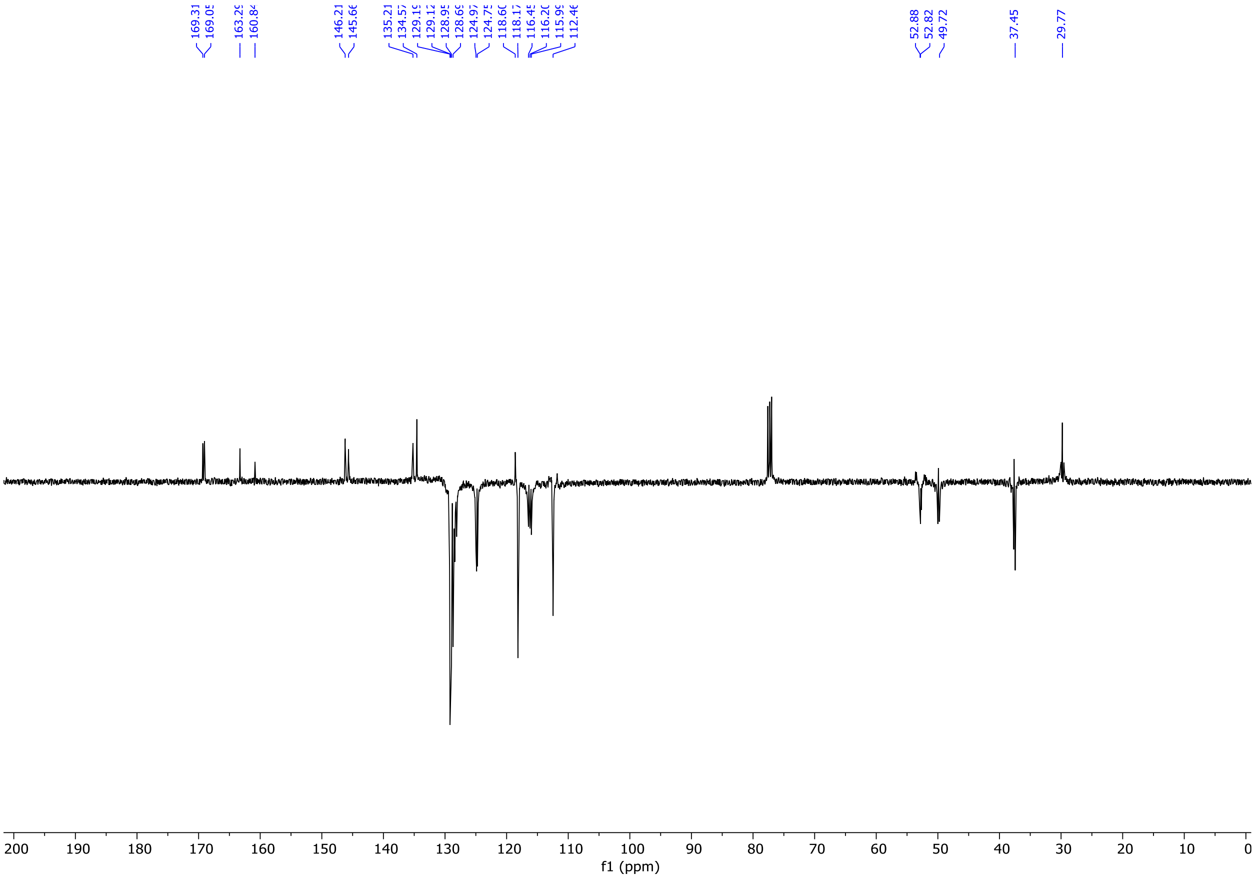

^{1}H NMR (400 MHz) and $^{13}C\{^{1}H\}$ APT NMR (100 MHz) spectra of **5d** ($CDCl_3$).

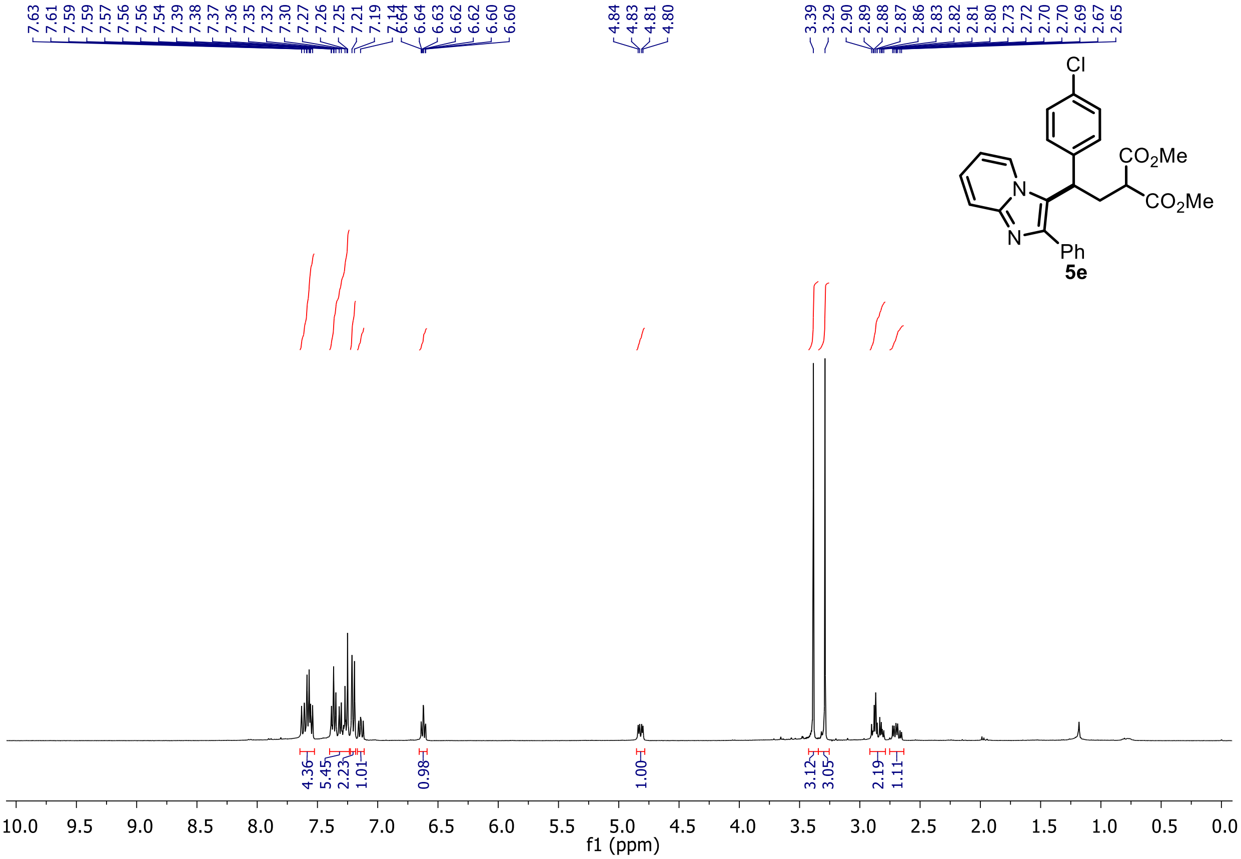

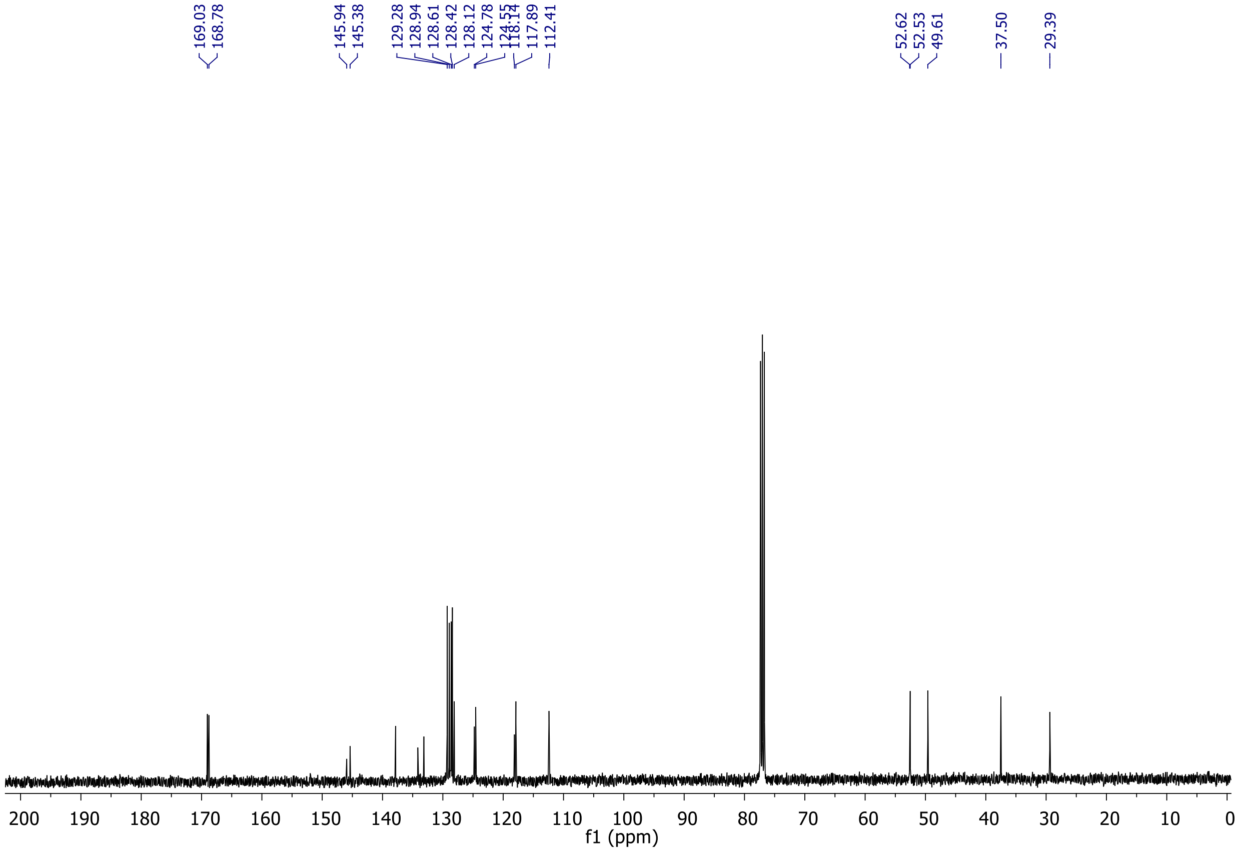

^{1}H NMR (400 MHz) and ^{13}C{^{1}H} NMR (100 MHz) spectra of **5e** ($CDCl_3$).

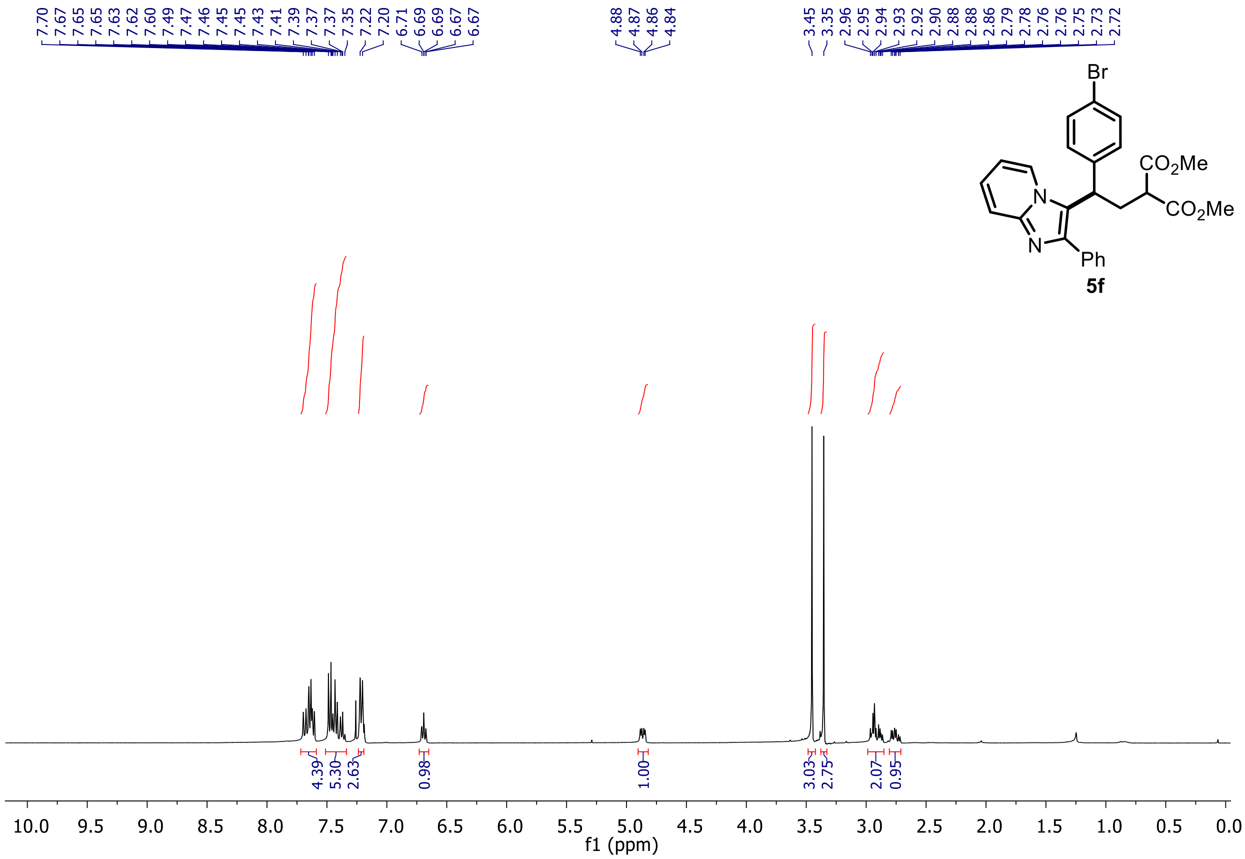

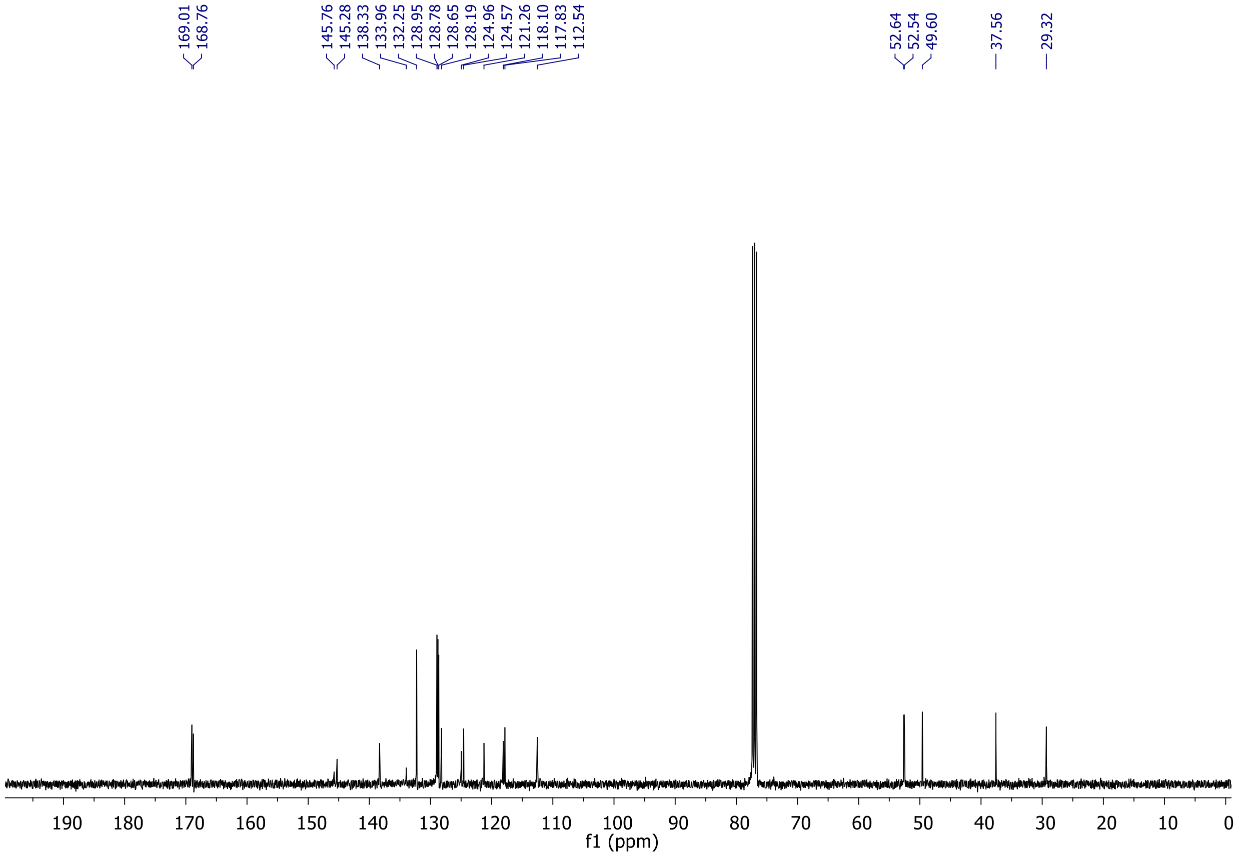

^{1}H NMR (400 MHz) and $^{13}C\{^{1}H\}$ NMR (100 MHz) spectra of **5f** ($CDCl_3$).

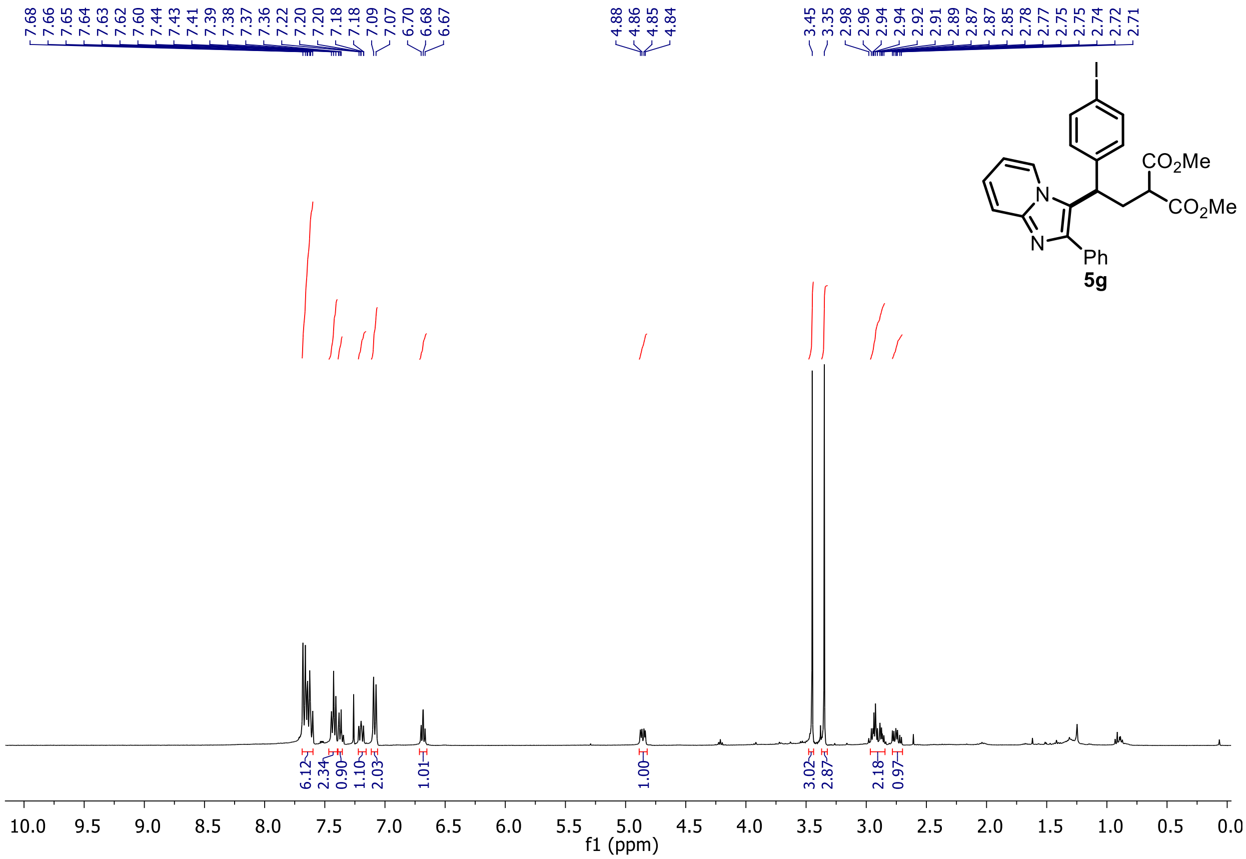

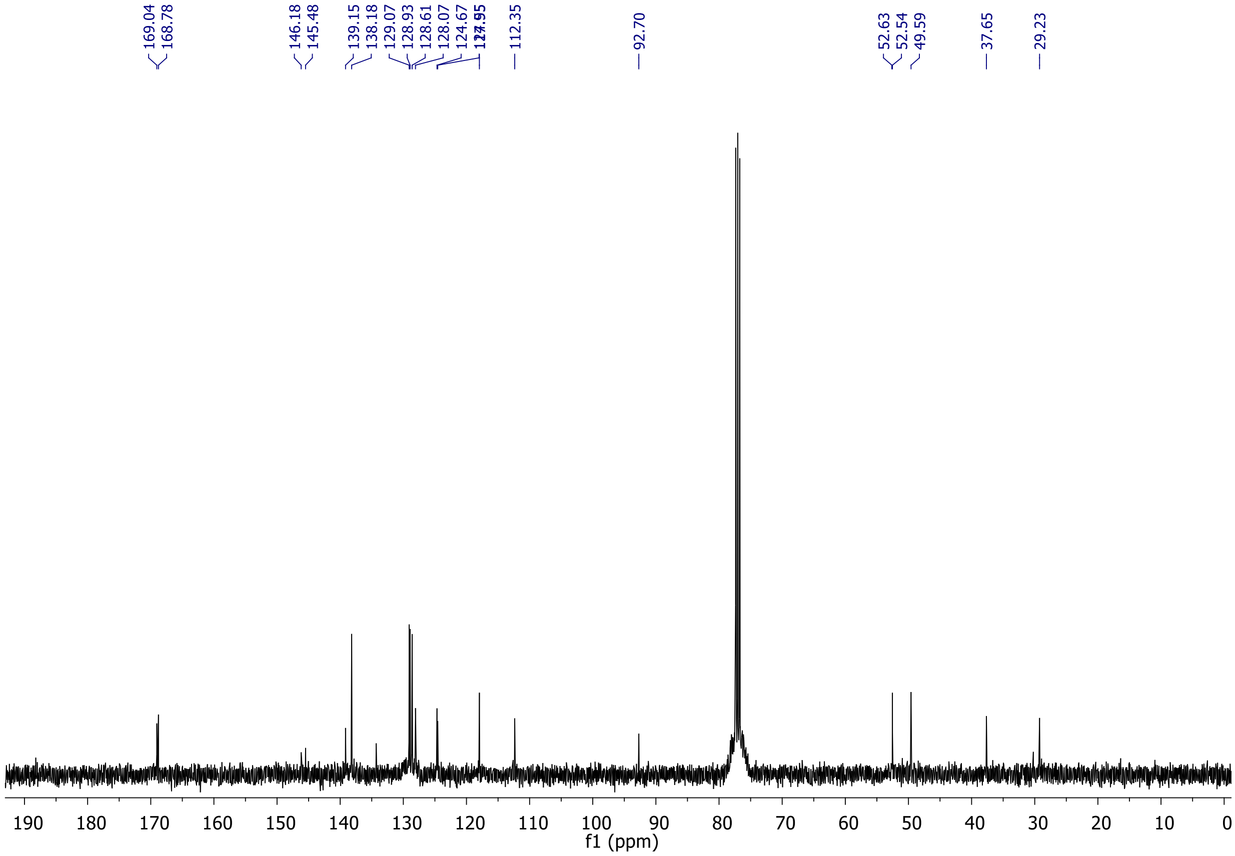

^{1}H NMR (400 MHz) and ^{13}C{^{1}H} NMR (100 MHz) spectra of **5g** ($CDCl_3$).

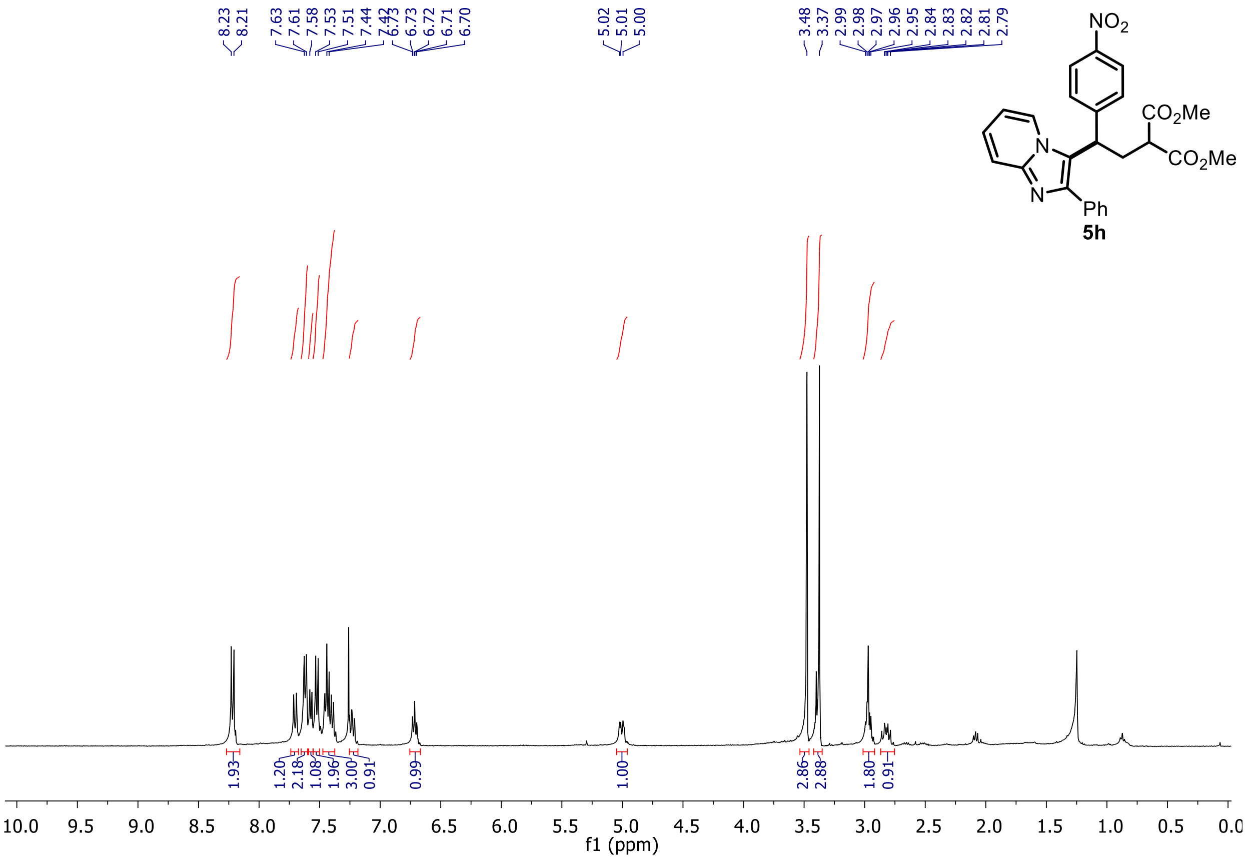

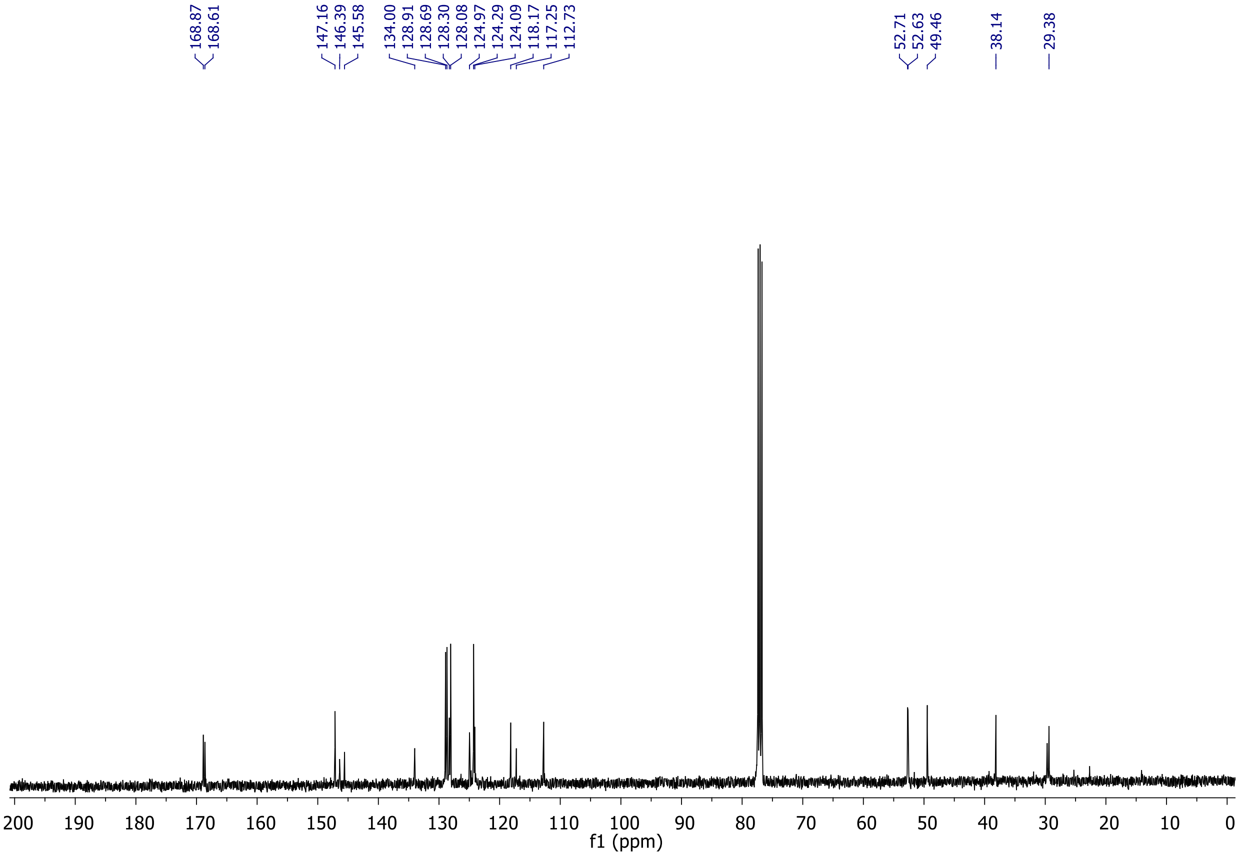

^{1}H NMR (400 MHz) and $^{13}C\{^{1}H\}$ NMR (100 MHz) spectra of **5h** ($CDCl_3$).

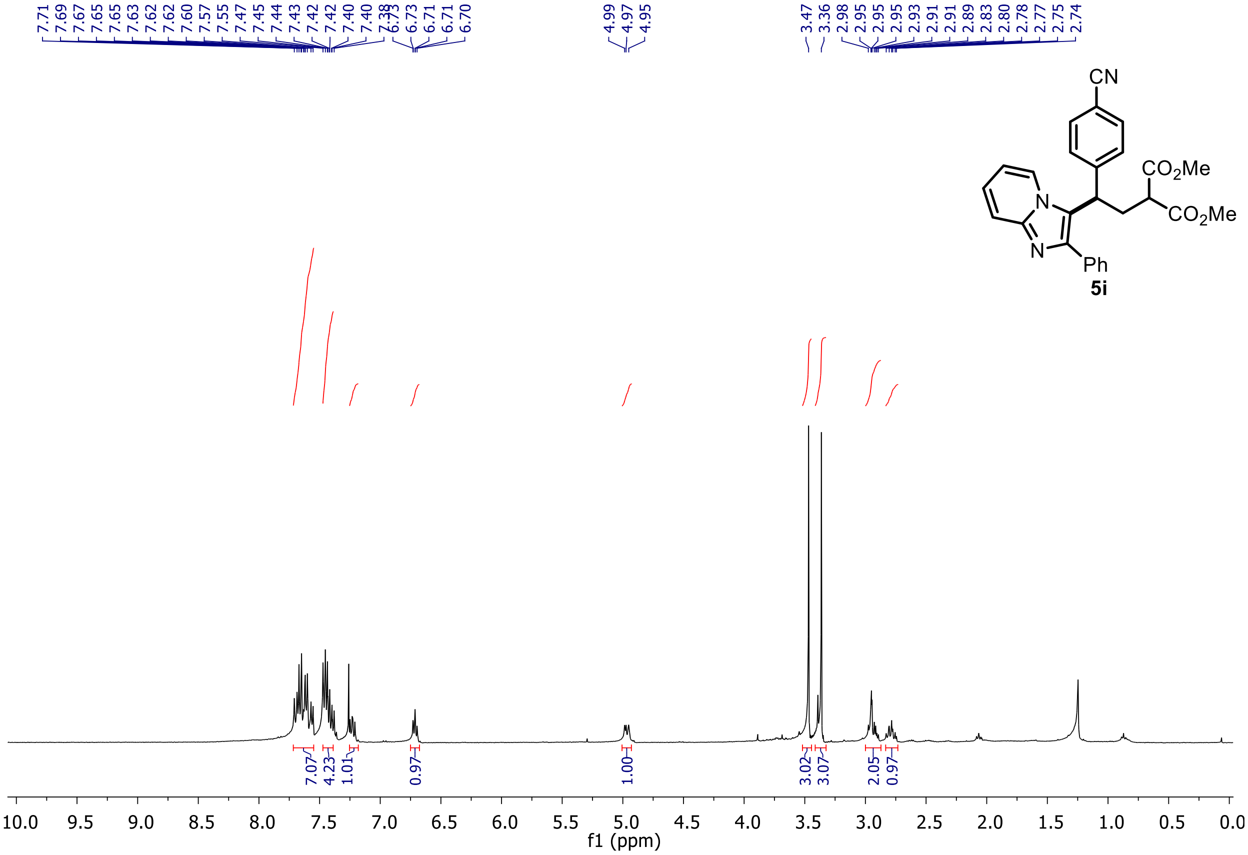

5i

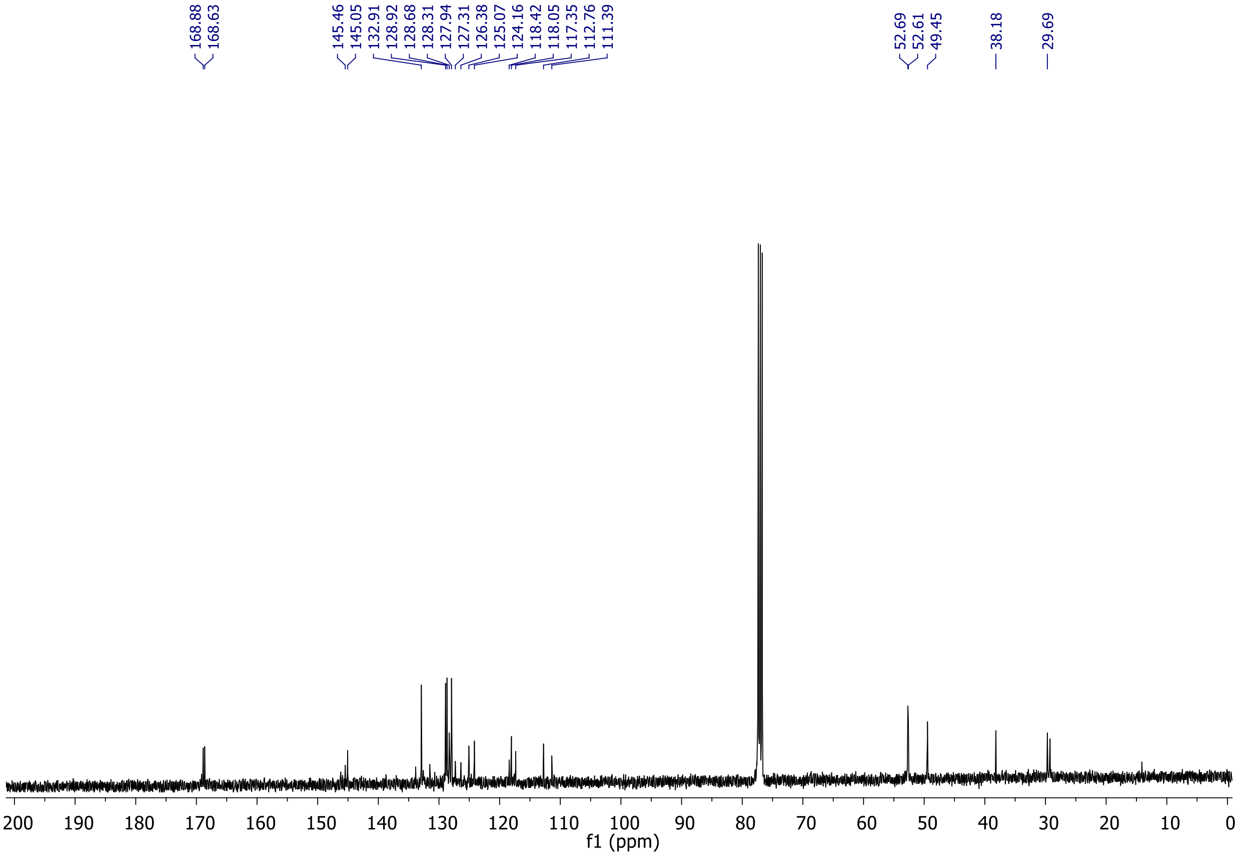

^{1}H NMR (400 MHz) and $^{13}C\{^{1}H\}$ NMR (100 MHz) spectra of **5i** ($CDCl_3$).

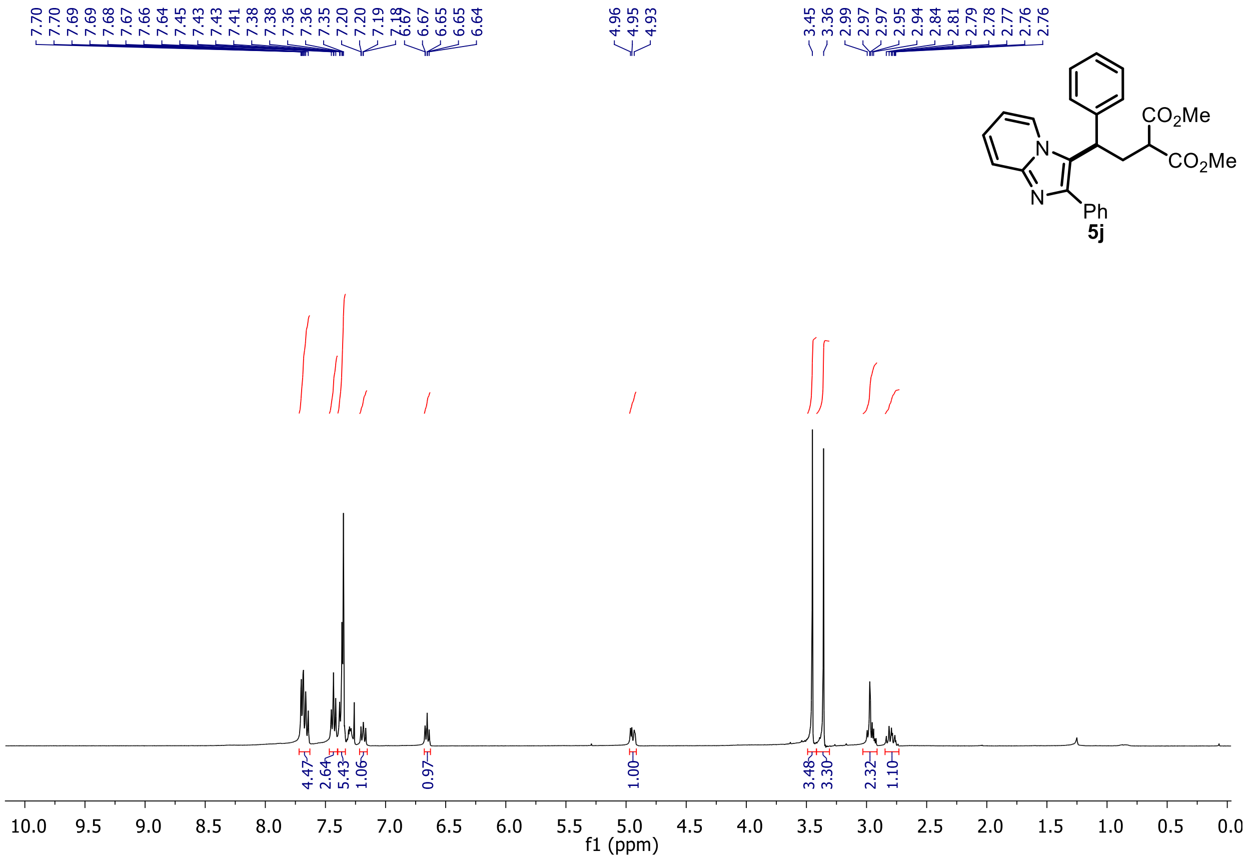

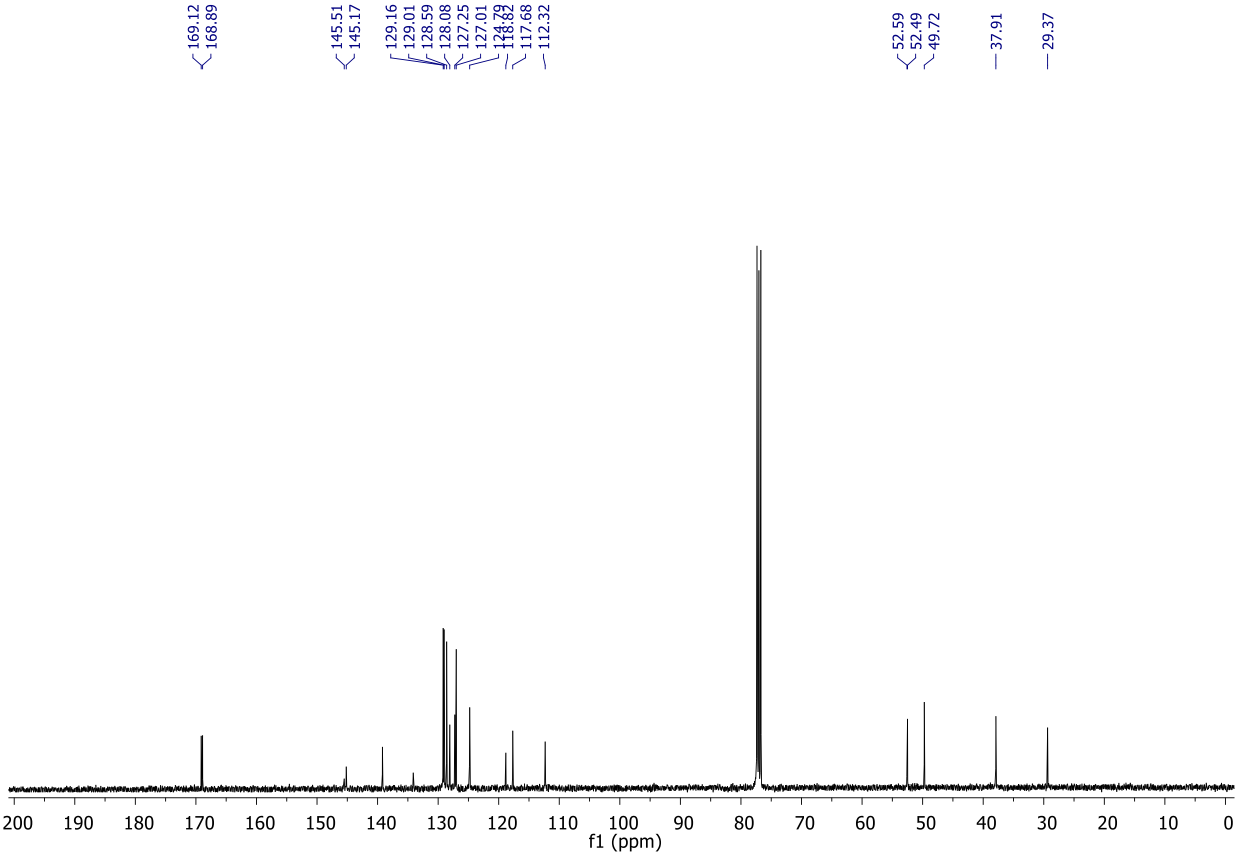

^{1}H NMR (400 MHz) and ^{13}C{^{1}H} NMR (100 MHz) spectra of **5j** ($CDCl_3$).

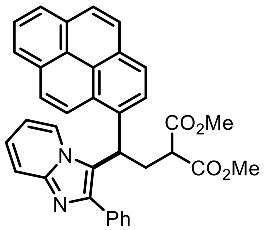

5k

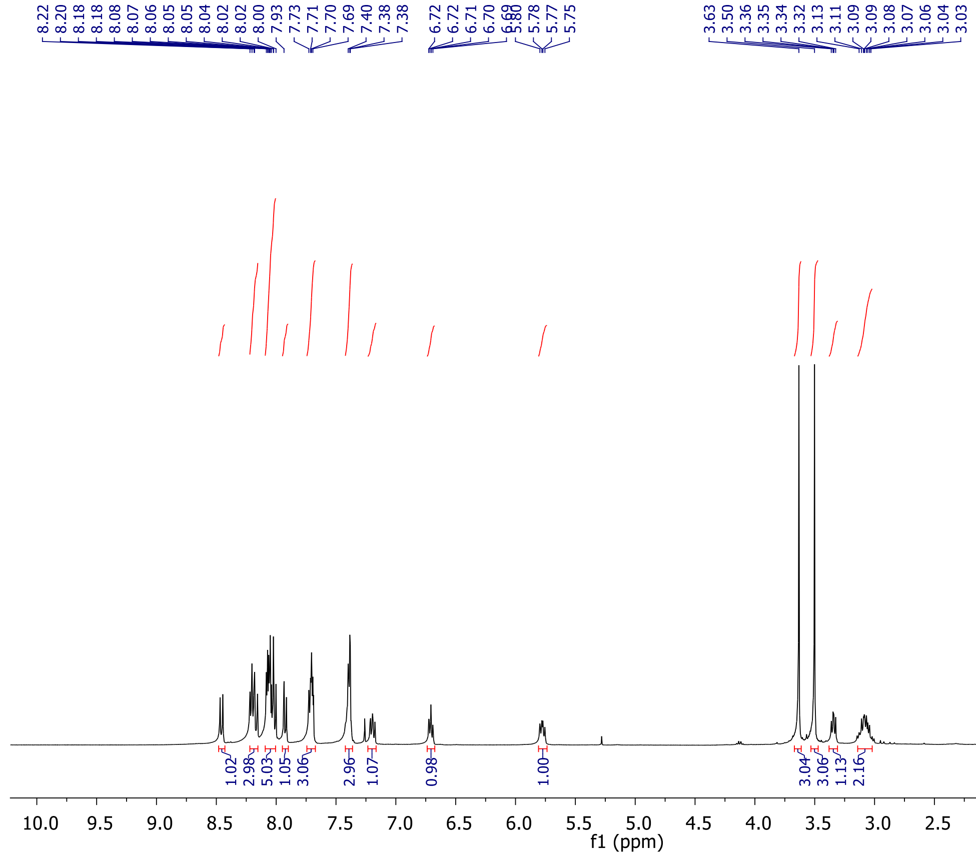

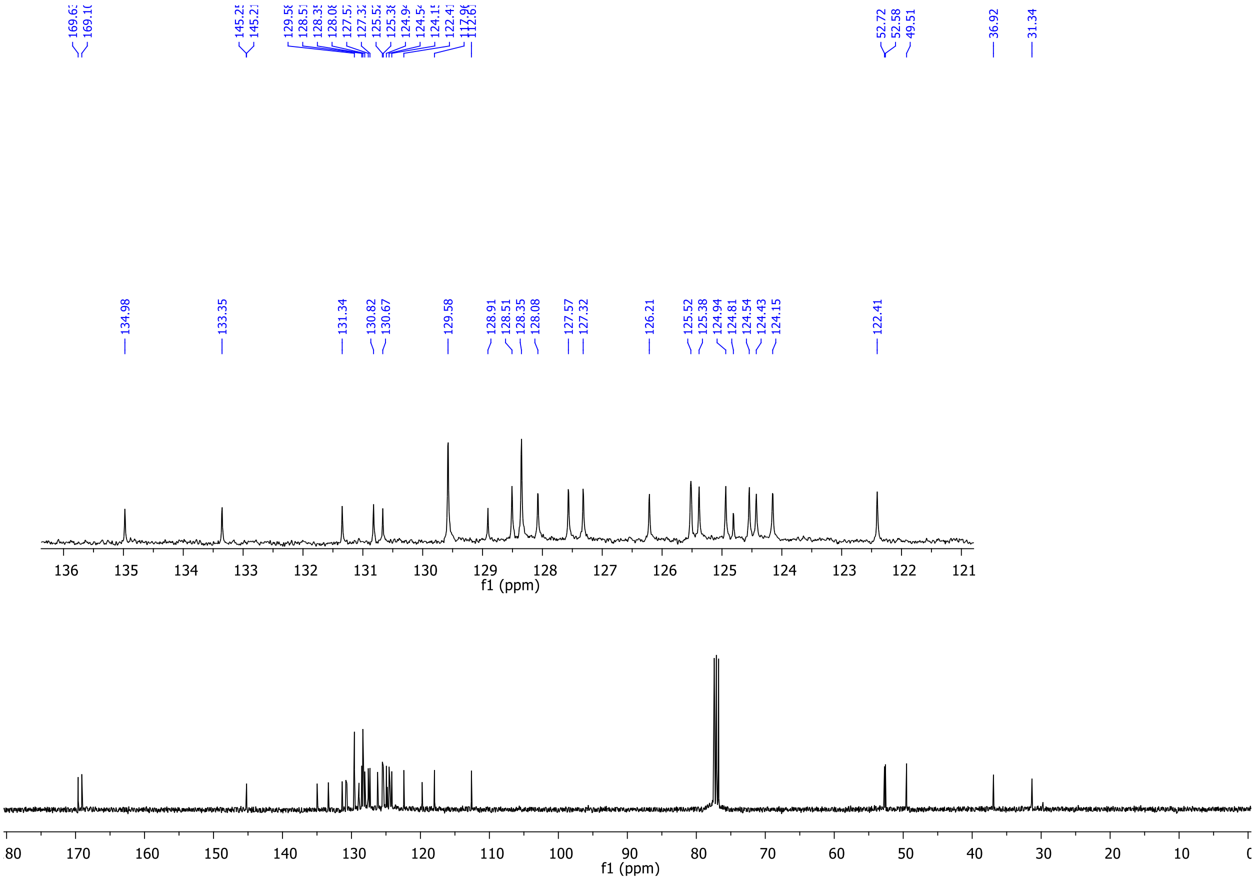

^{1}H NMR (400 MHz) and $^{13}C\{^{1}H\}$ NMR (100 MHz) spectra of **5k** ($CDCl_3$).

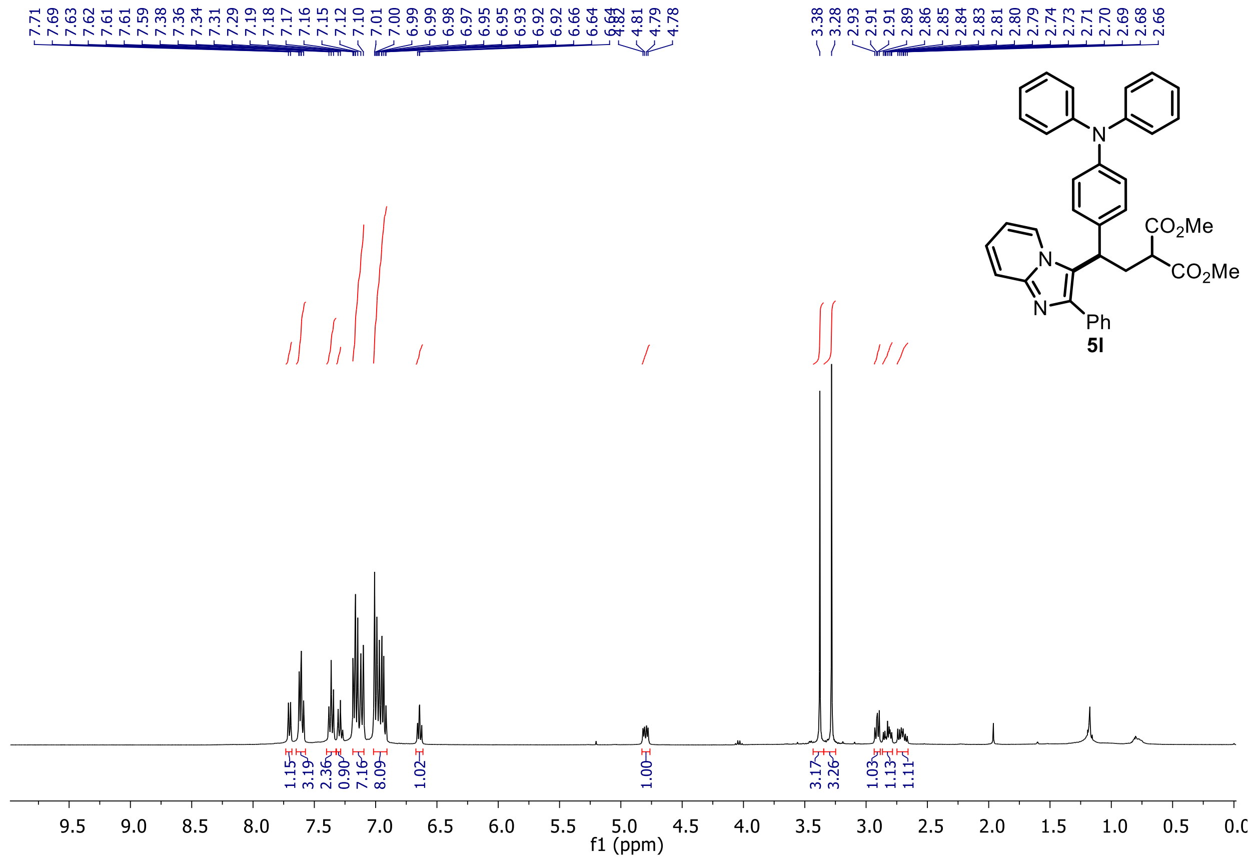

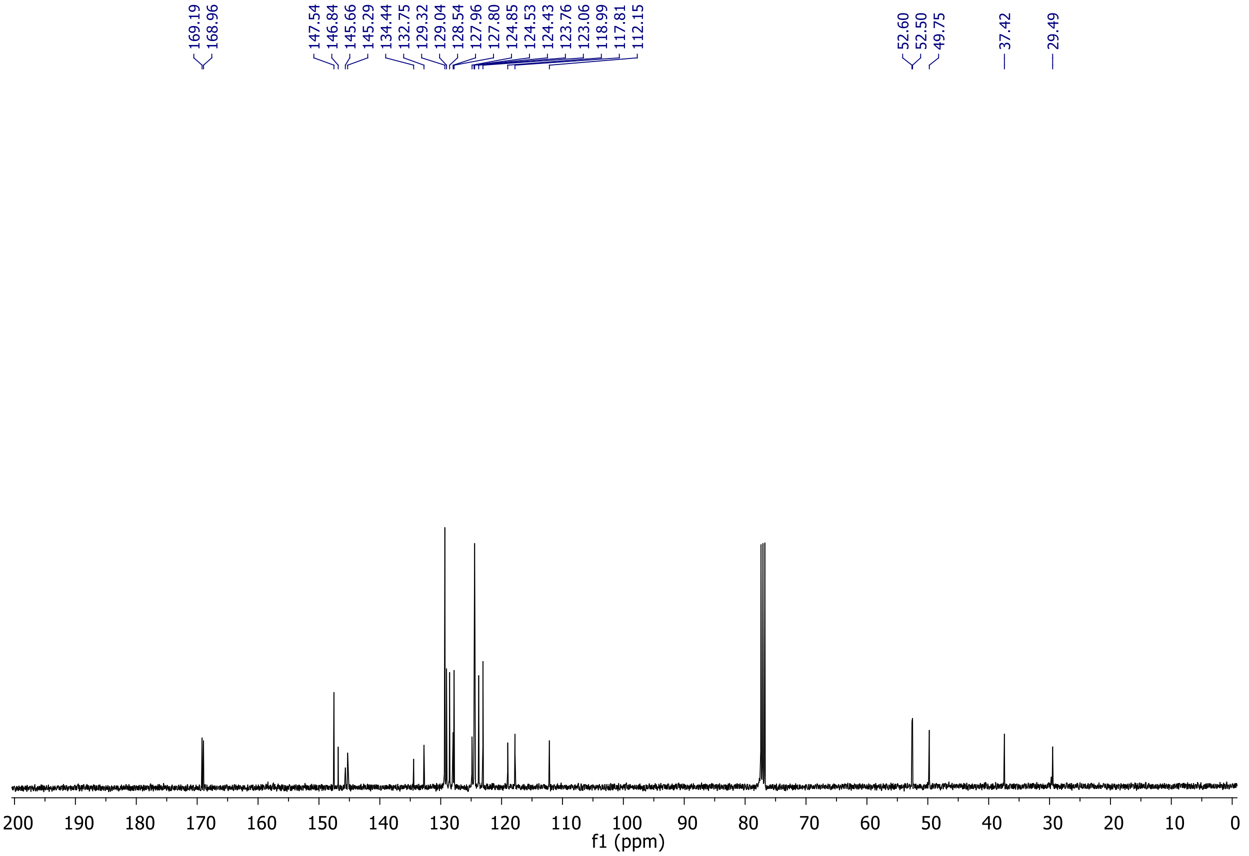

^{1}H NMR (400 MHz) and $^{13}C\{^{1}H\}$ NMR (100 MHz) spectra of **5l** ($CDCl_3$).

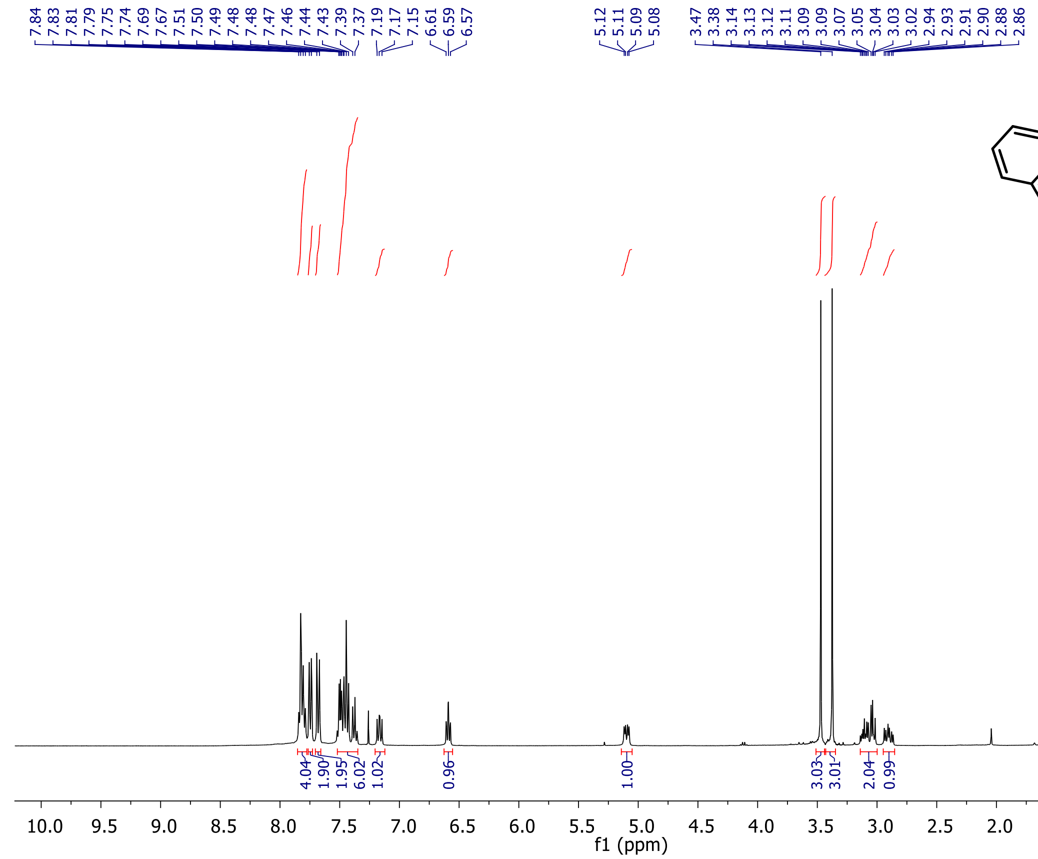

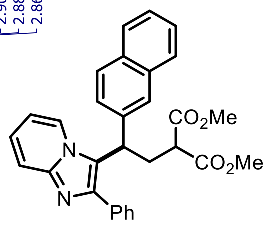

5m

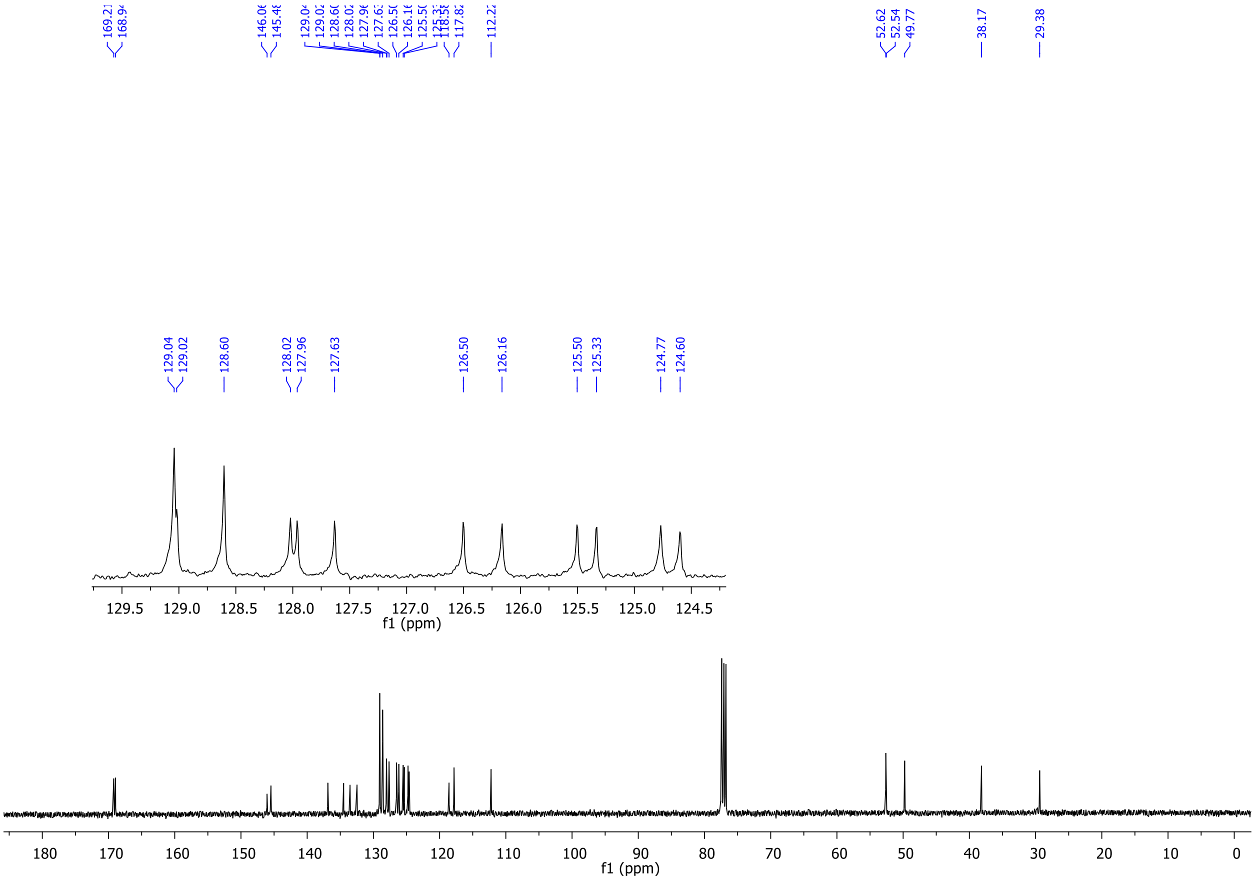

^{1}H NMR (400 MHz) and ^{13}C{^{1}H} NMR (100 MHz) spectra of **5m** ($CDCl_3$).

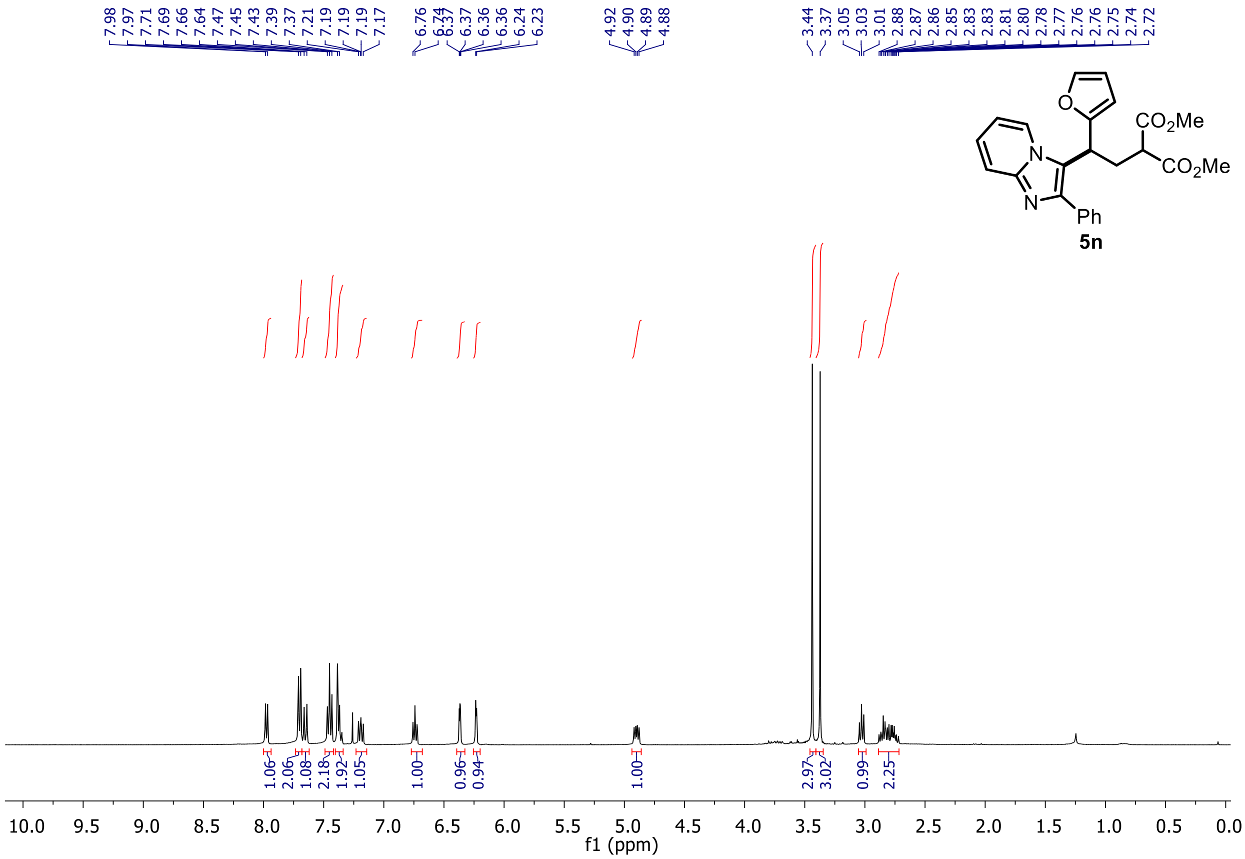

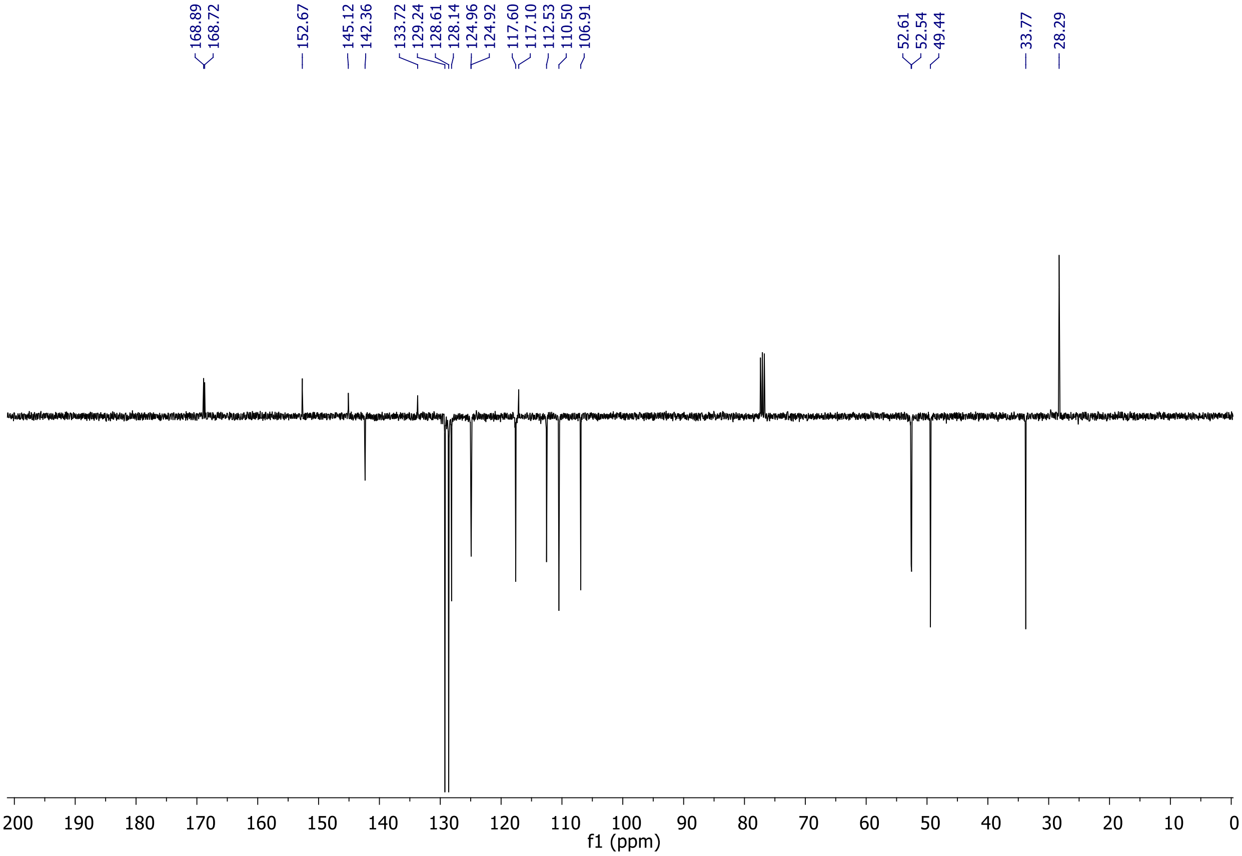

^{1}H NMR (400 MHz) and ^{13}C{^{1}H} APT NMR (100 MHz) spectra of **5n** ($CDCl_3$).

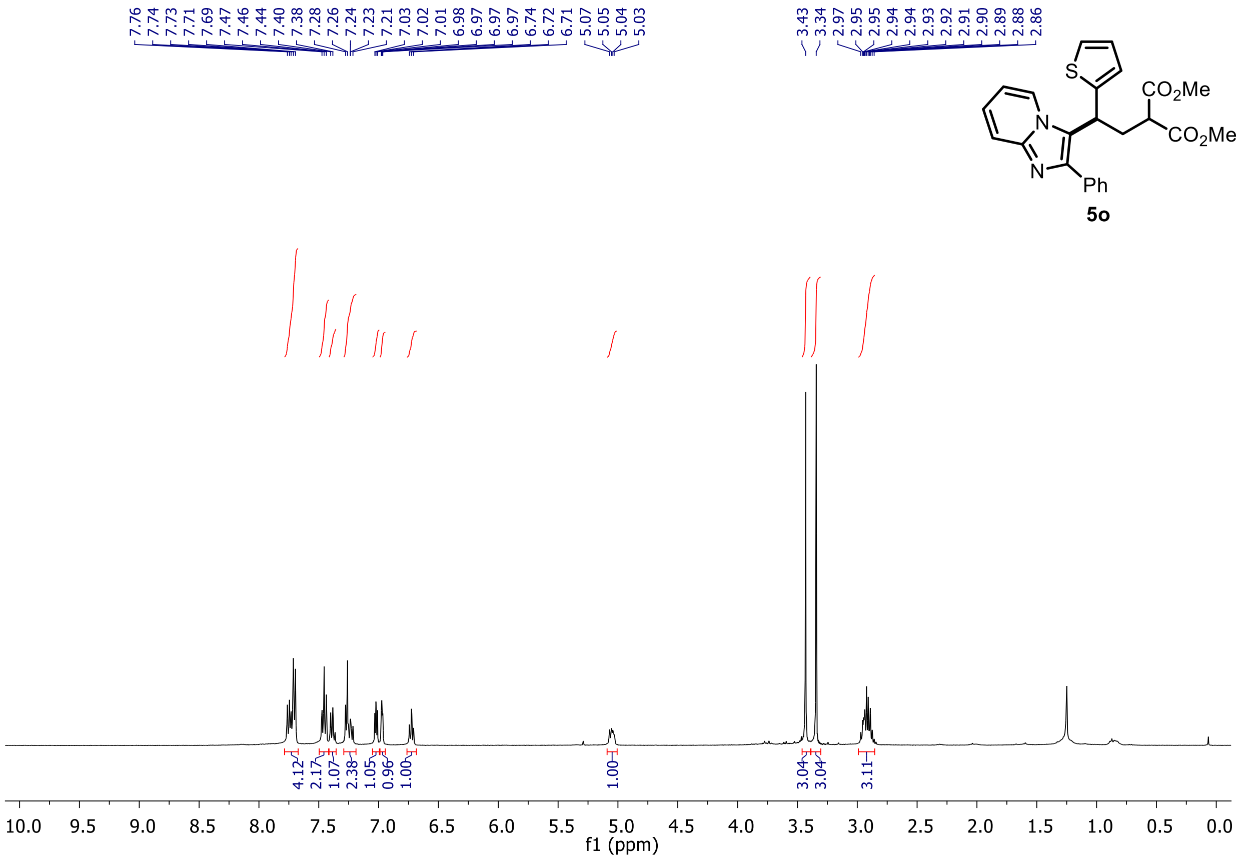

5o

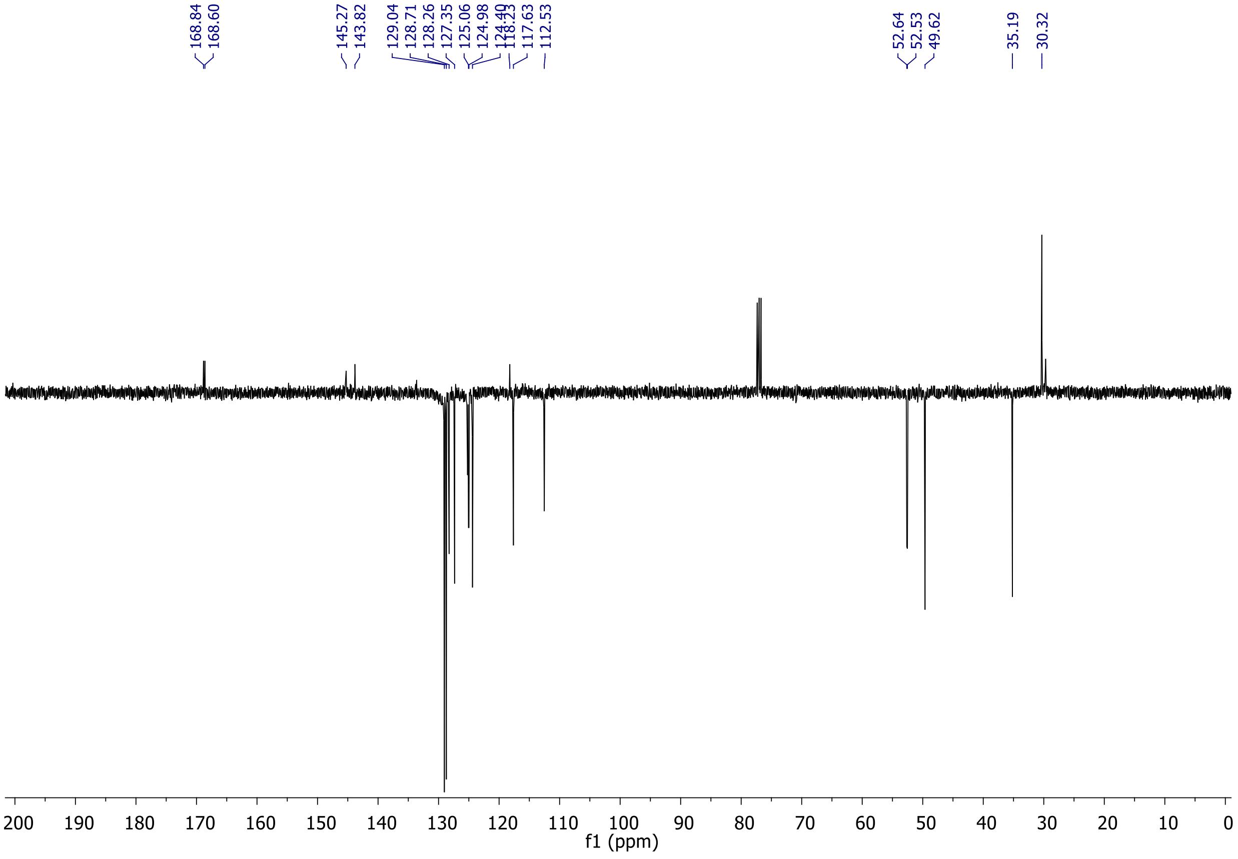

^{1}H NMR (400 MHz) and ^{13}C{^{1}H} APT NMR (100 MHz) spectra of **5o** ($CDCl_3$).

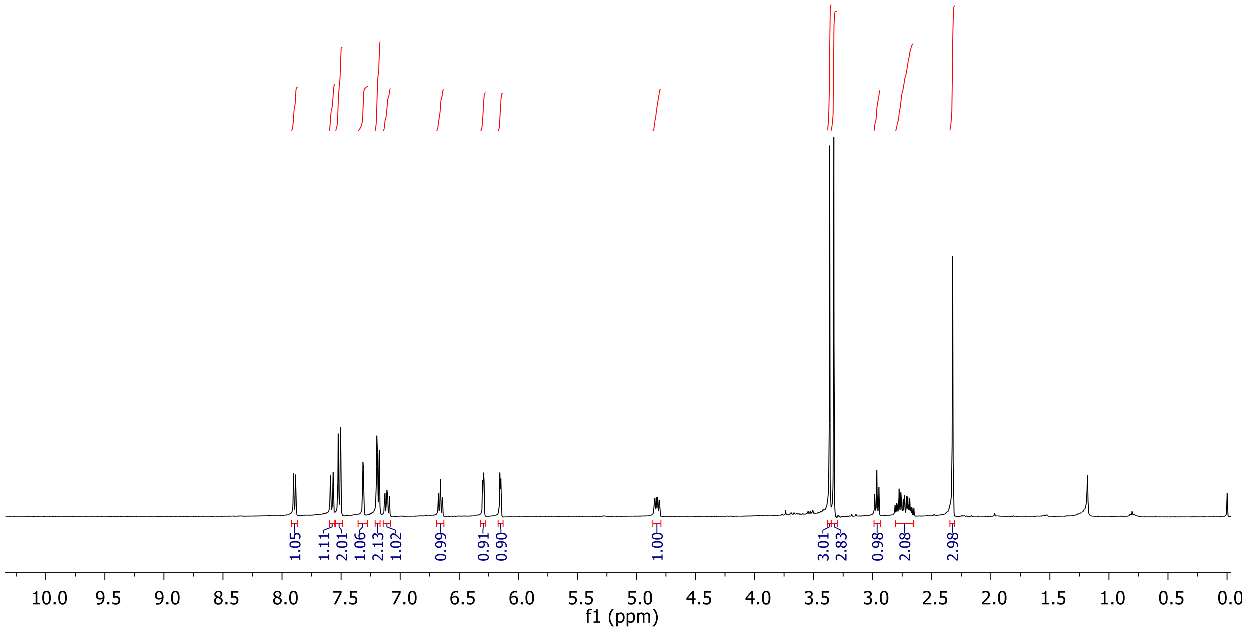

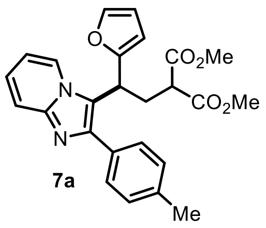

7a

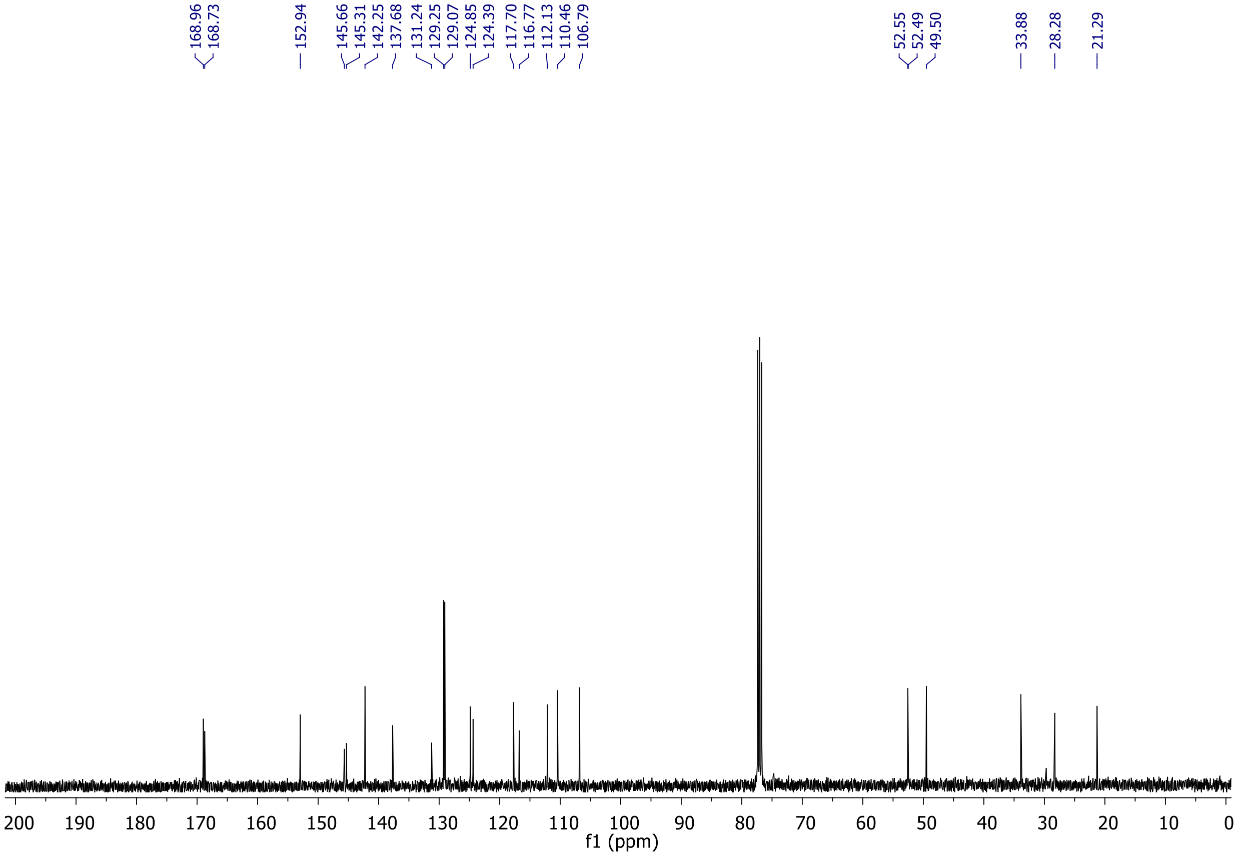

^{1}H NMR (400 MHz) and $^{13}C\{^{1}H\}$ NMR (100 MHz) spectra of **7a** ($CDCl_3$).

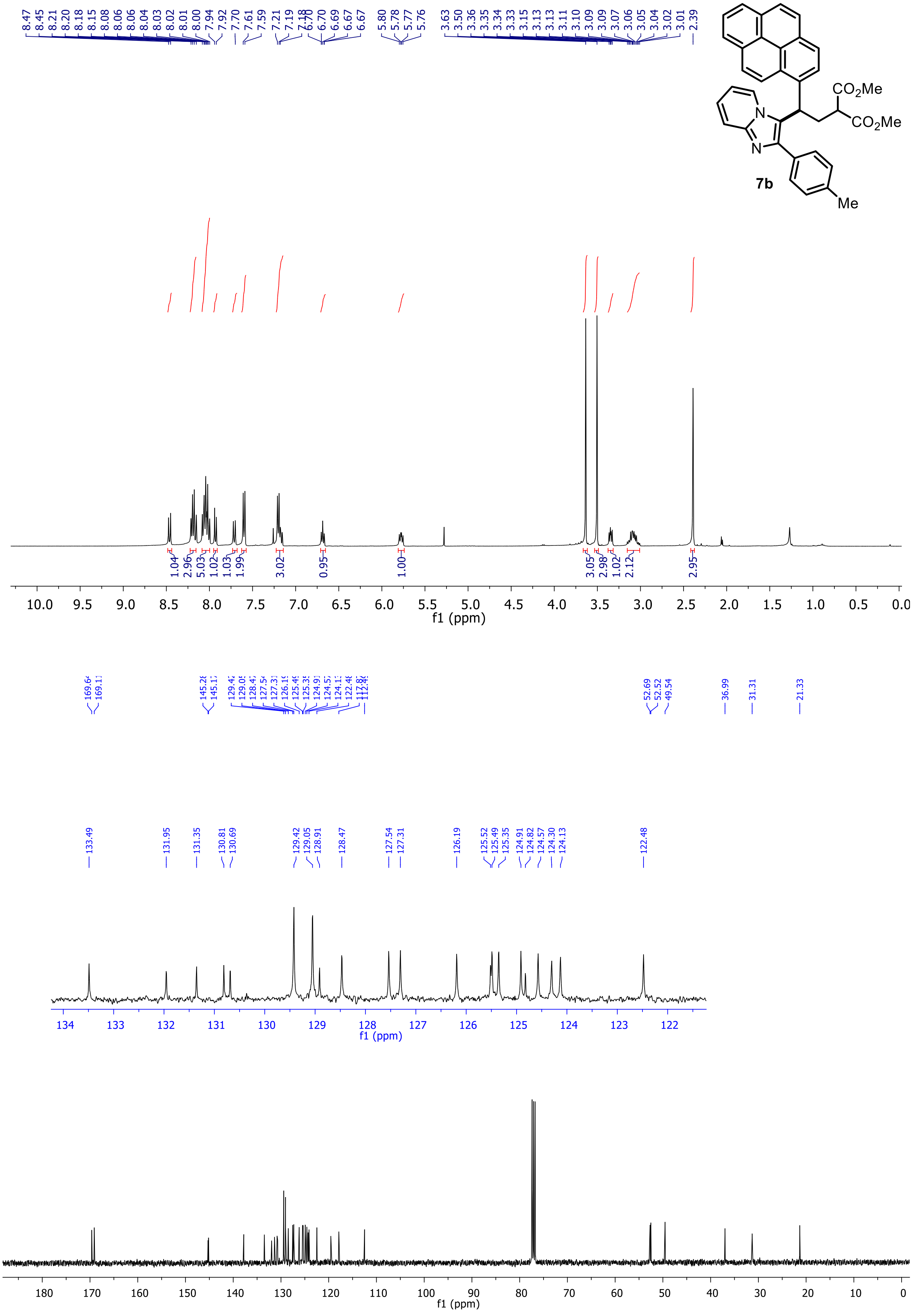

^{1}H NMR (400 MHz) and $^{13}C\{^{1}H\}$ NMR (100 MHz) spectra of **7b** ($CDCl_3$).

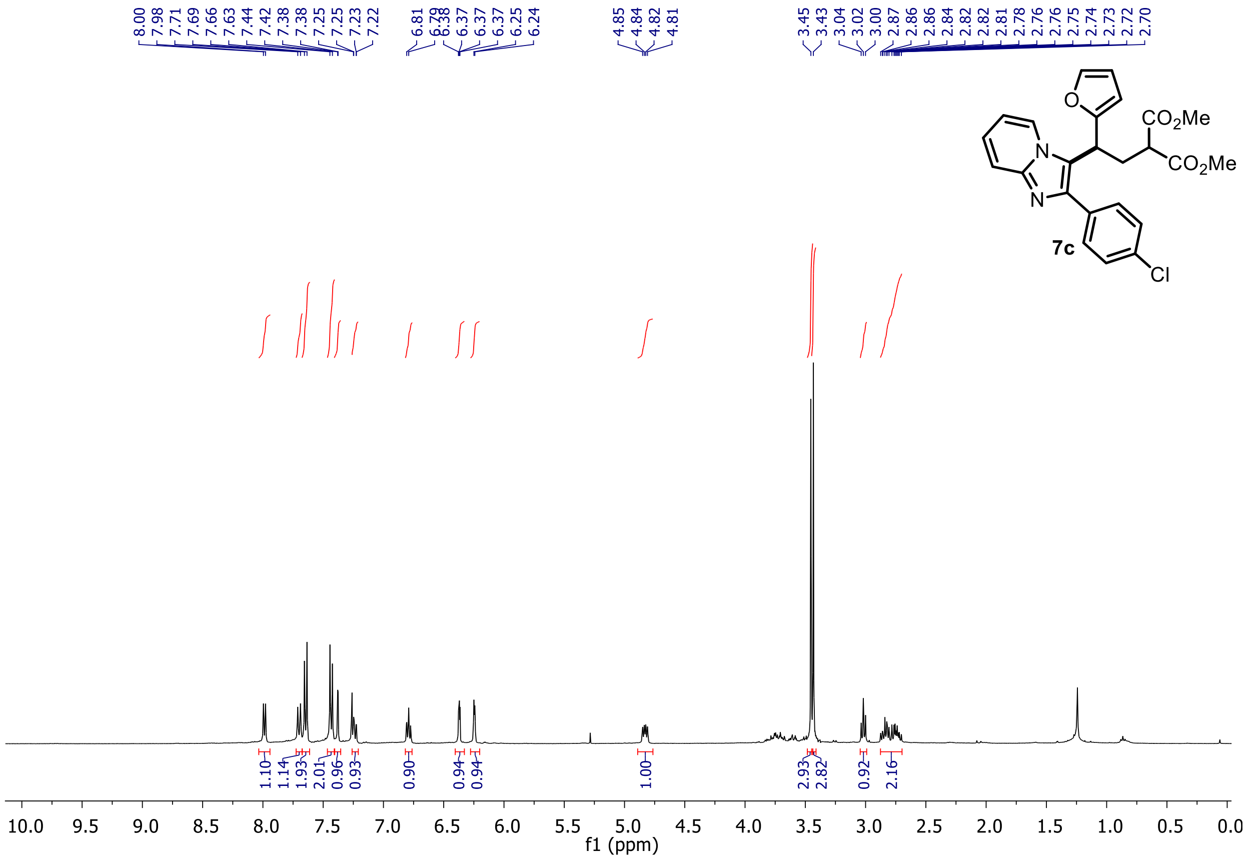

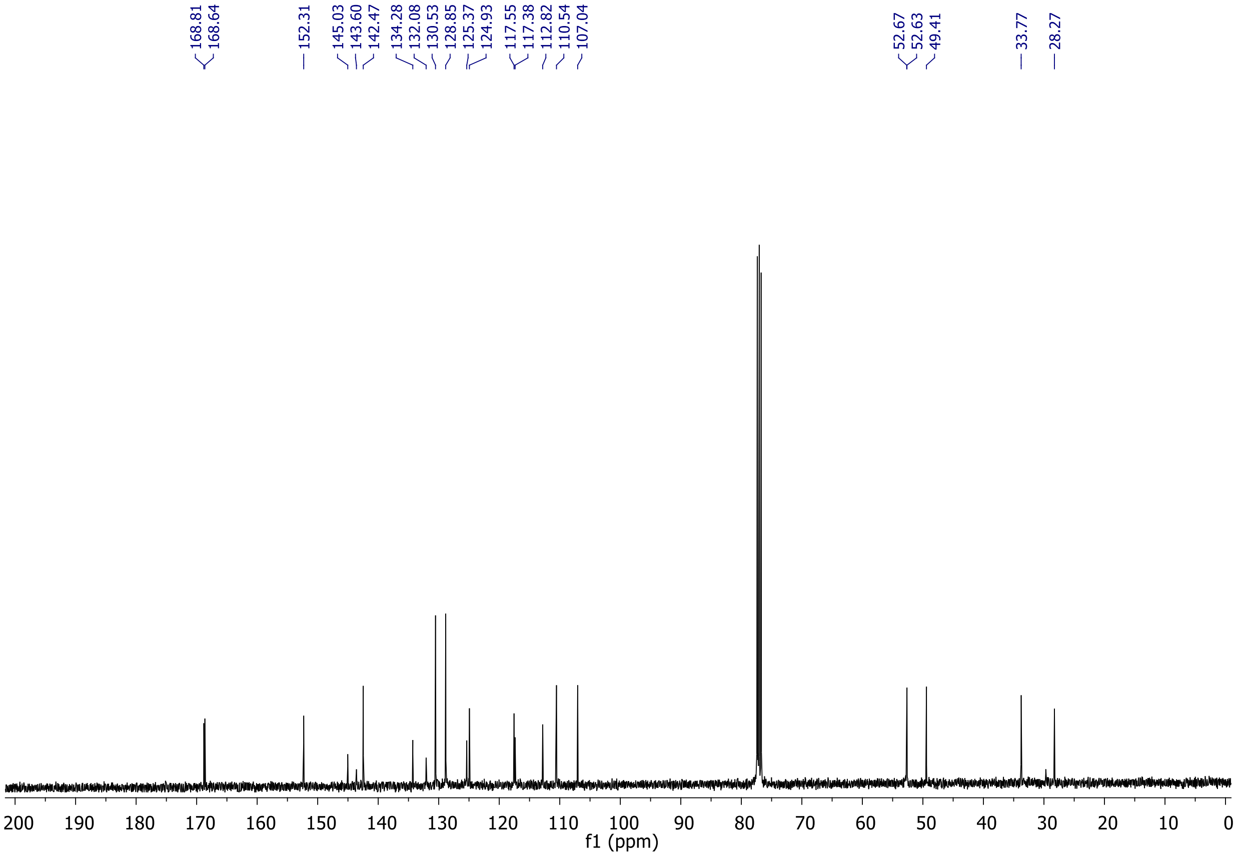

^{1}H NMR (400 MHz) and ^{13}C{^{1}H} NMR (100 MHz) spectra of **7c** ($CDCl_3$).

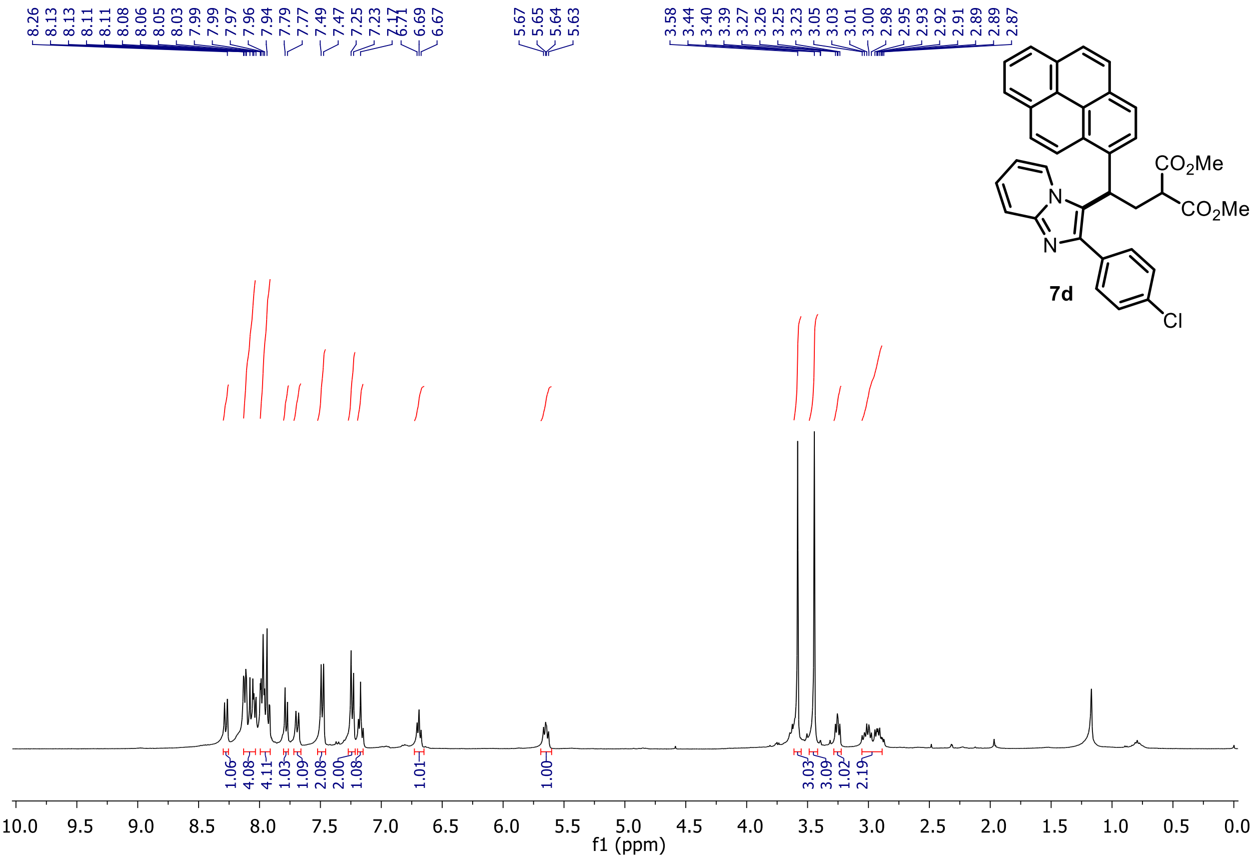

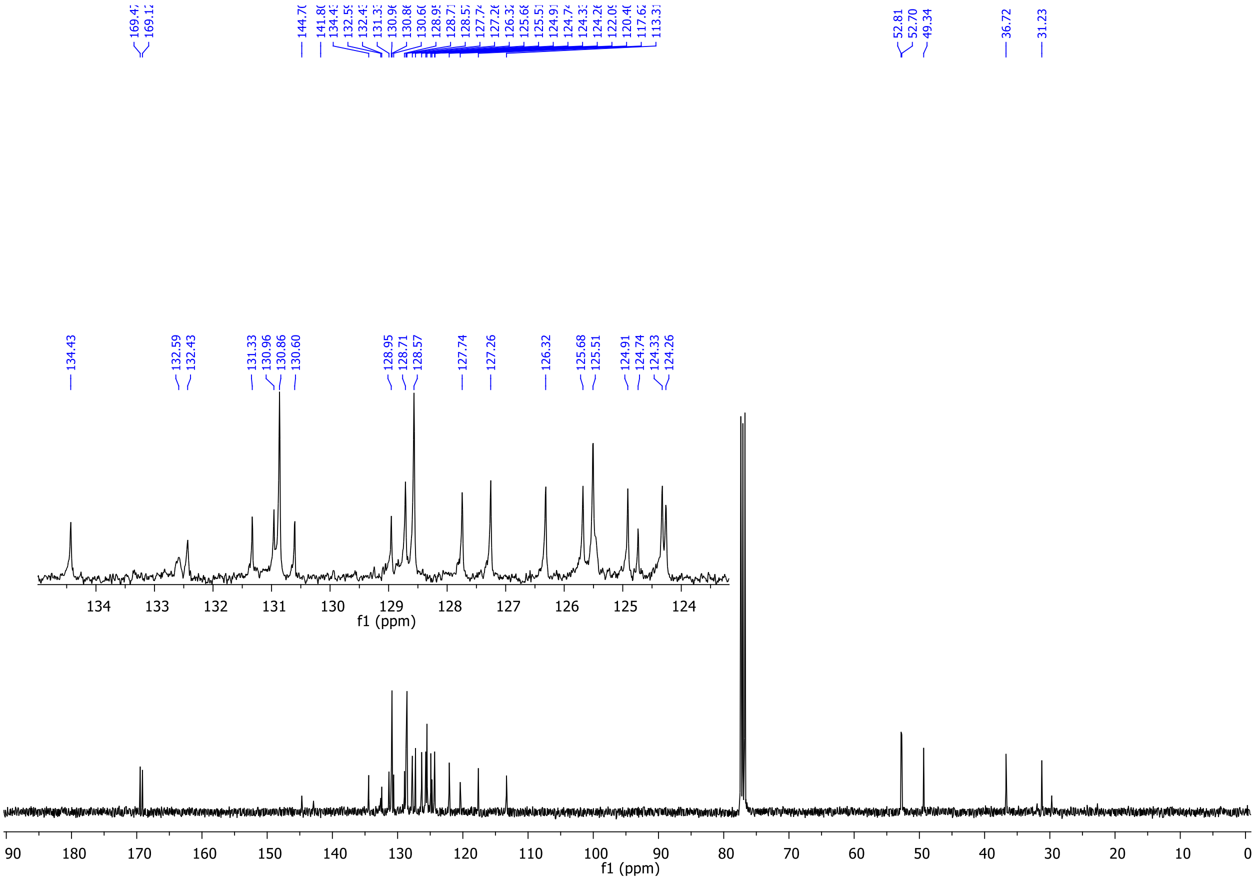

^{1}H NMR (400 MHz) and $^{13}C\{^{1}H\}$ NMR (100 MHz) spectra of **7d** ($CDCl_3$).

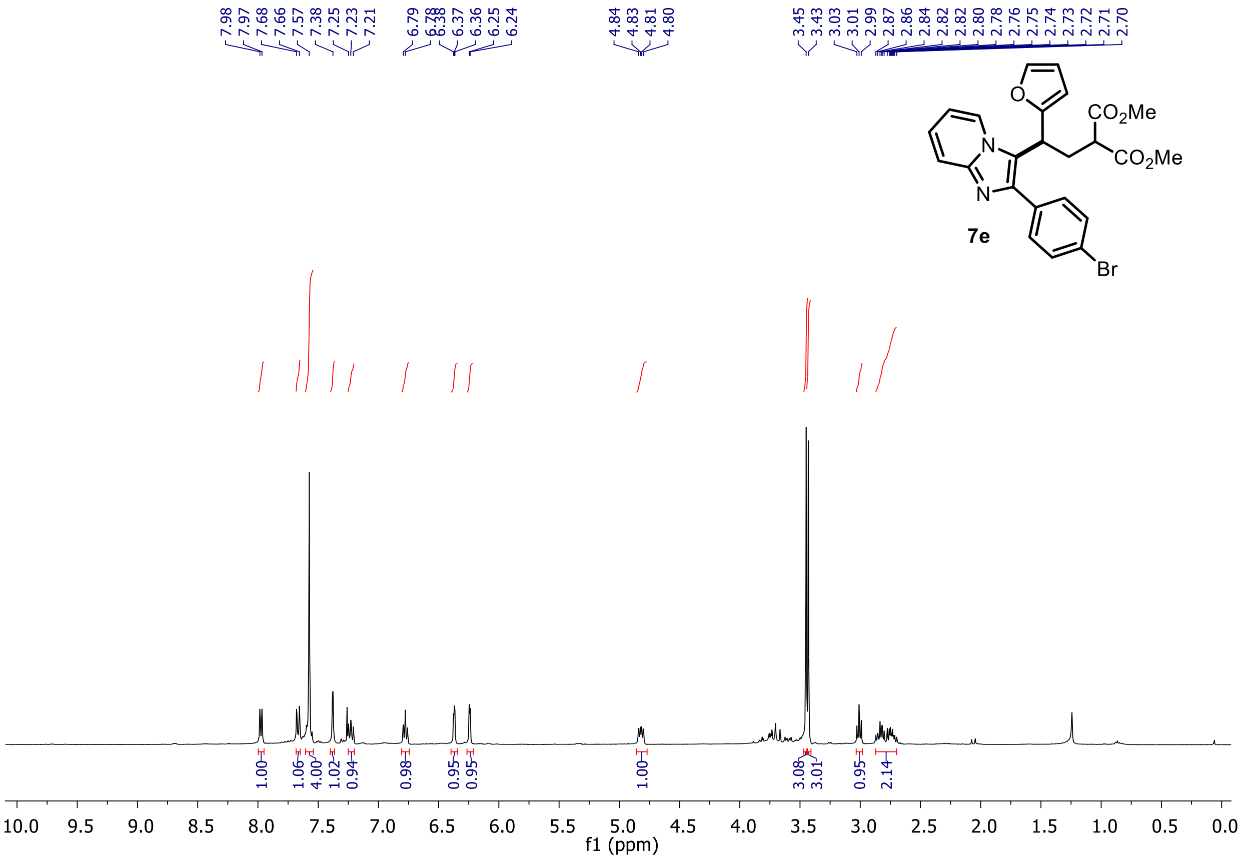

7e

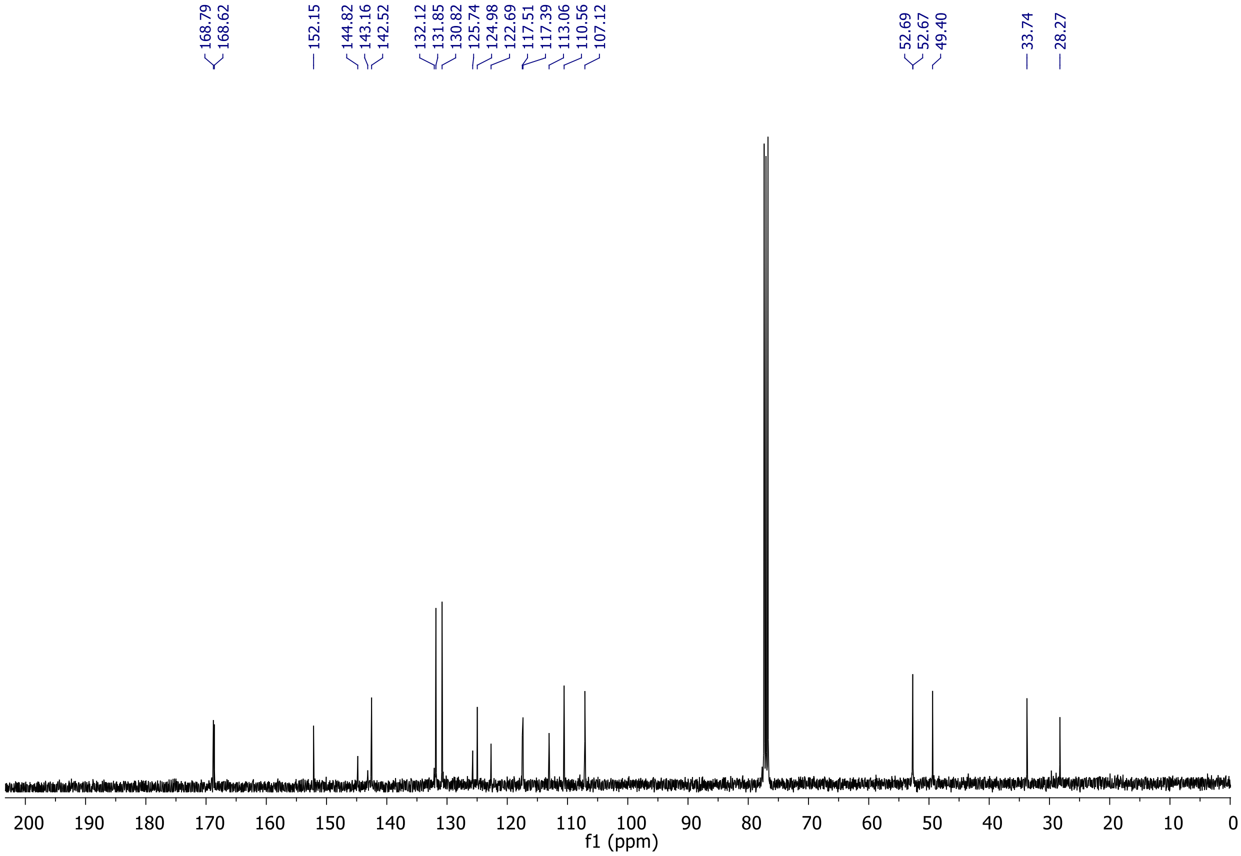

^{1}H NMR (400 MHz) and $^{13}C\{^{1}H\}$ NMR (100 MHz) spectra of **7e** ($CDCl_3$).

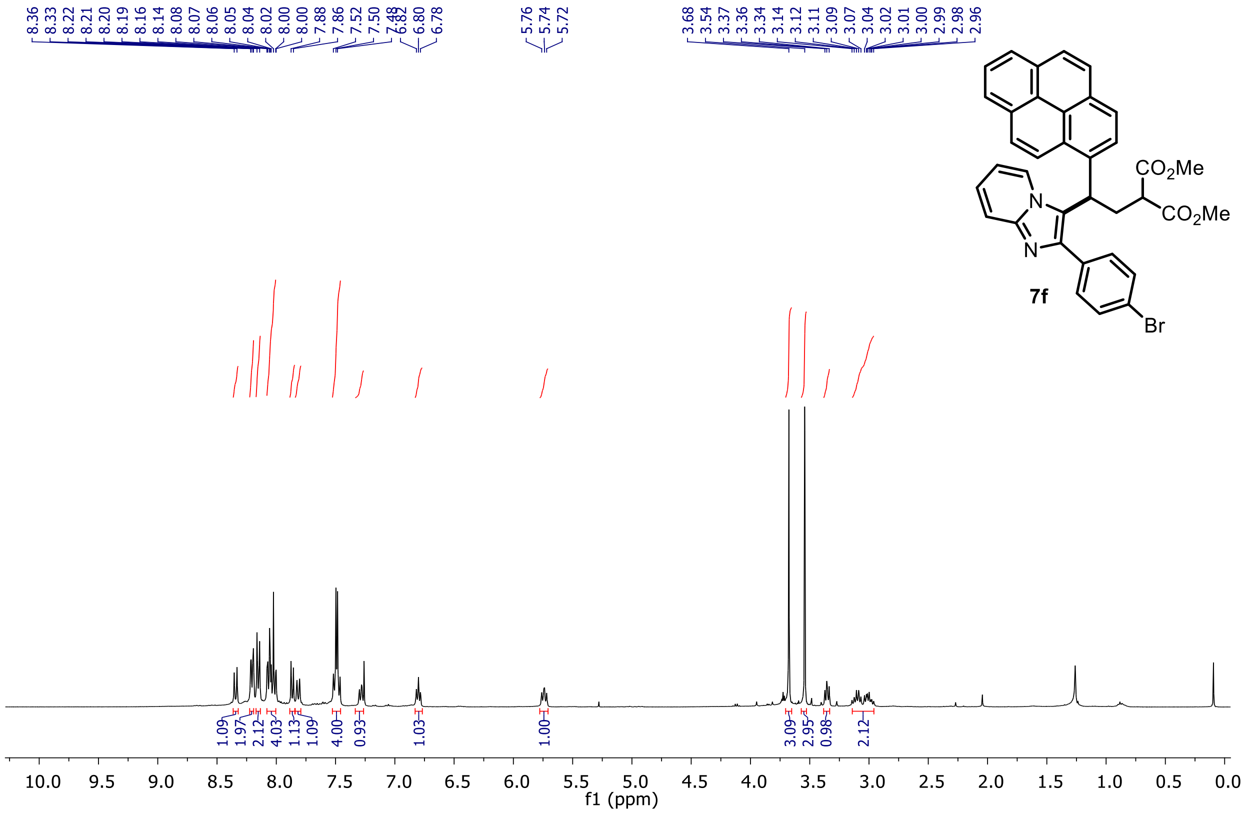

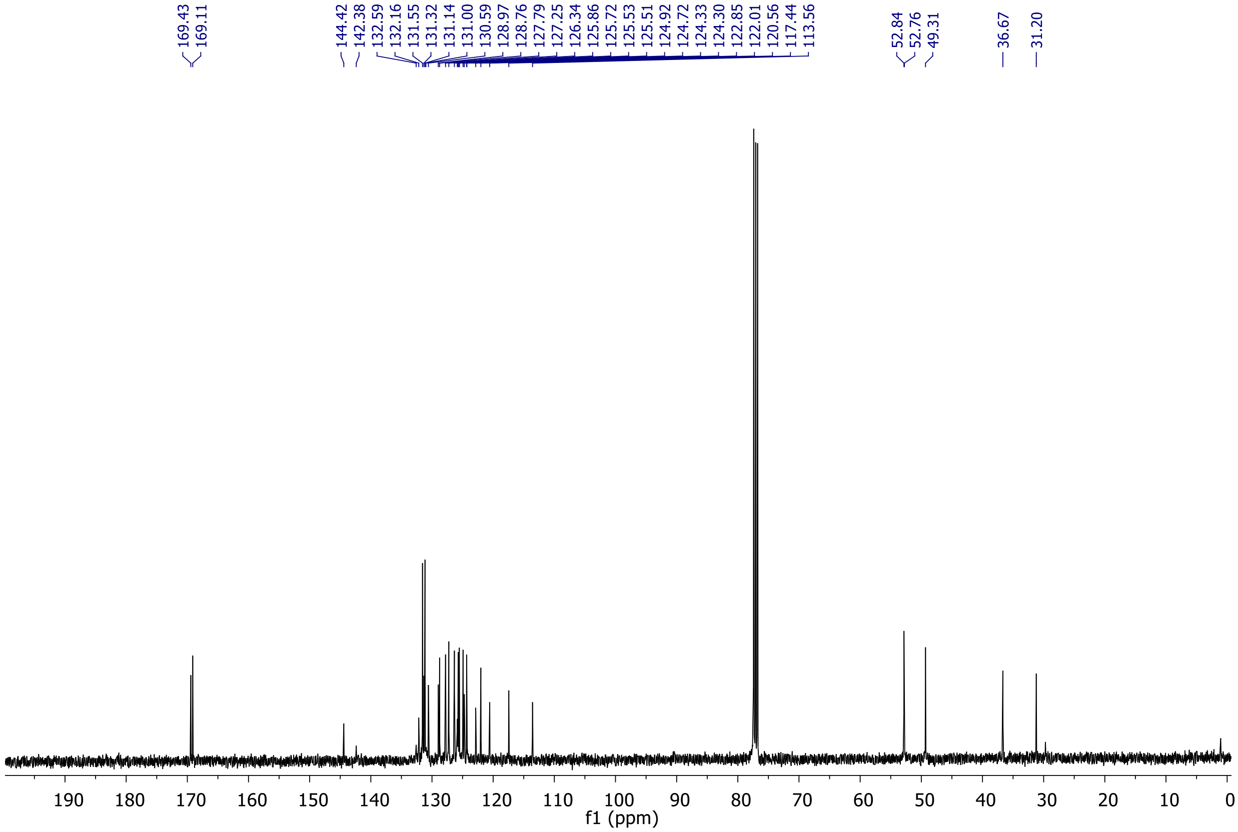

^{1}H NMR (400 MHz) and ^{13}C{^{1}H} NMR (100 MHz) spectra of **7f** ($CDCl_3$).

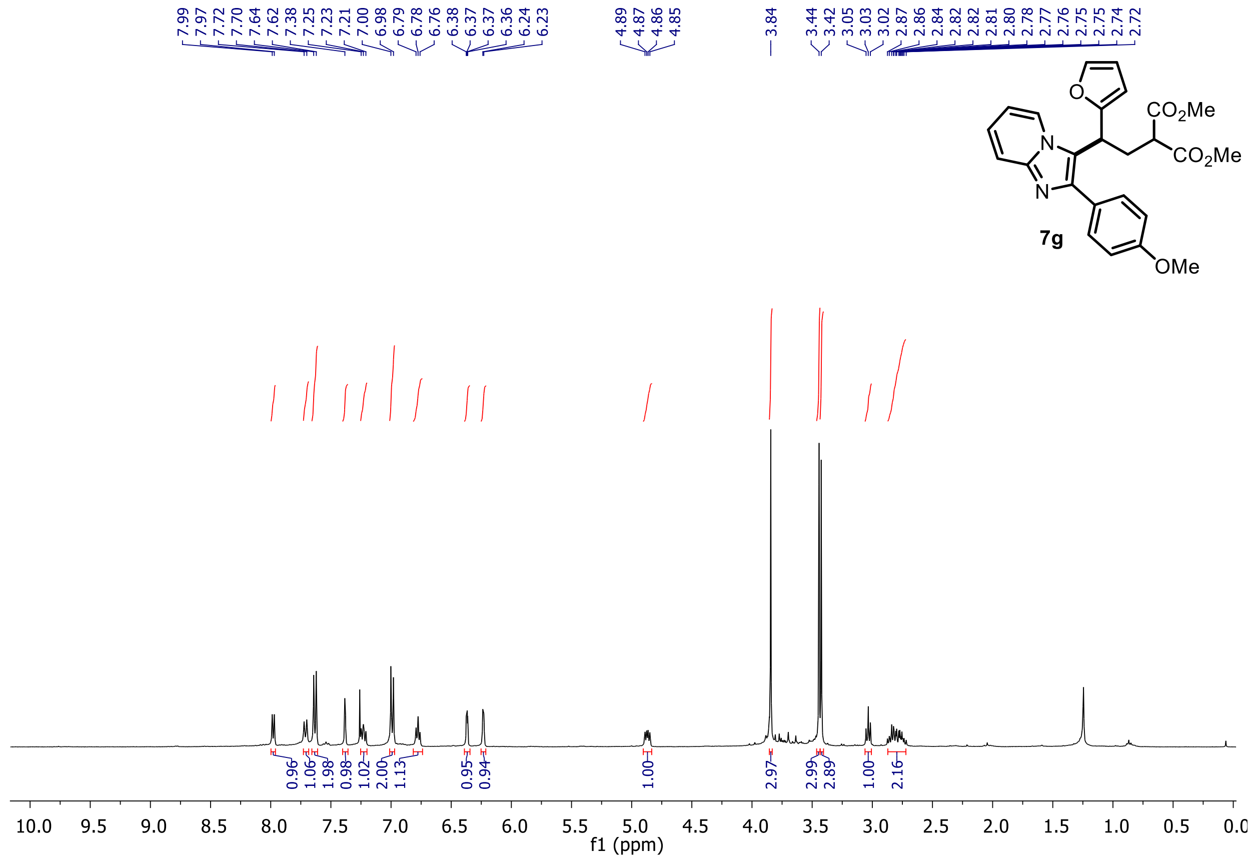

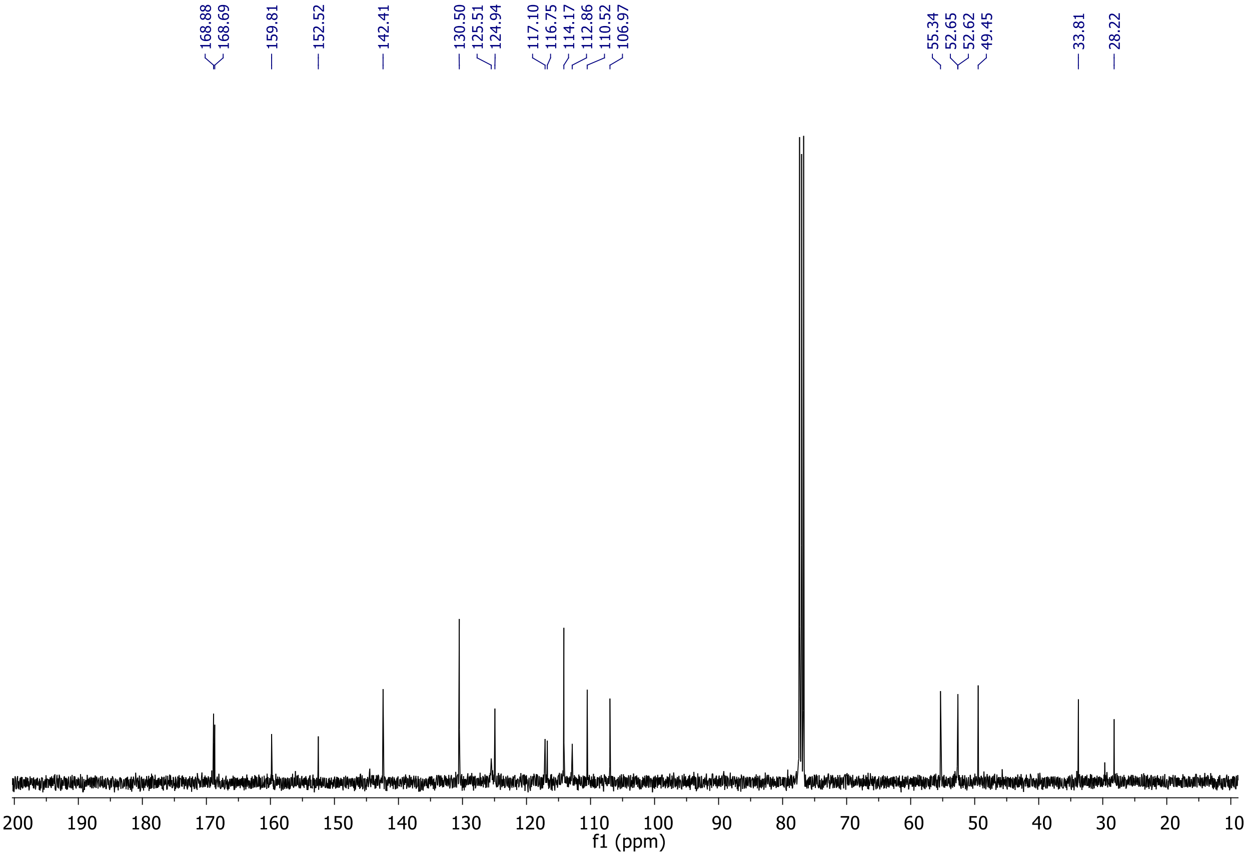

^{1}H NMR (400 MHz) and $^{13}C\{^{1}H\}$ NMR (100 MHz) spectra of **7g** ($CDCl_3$).

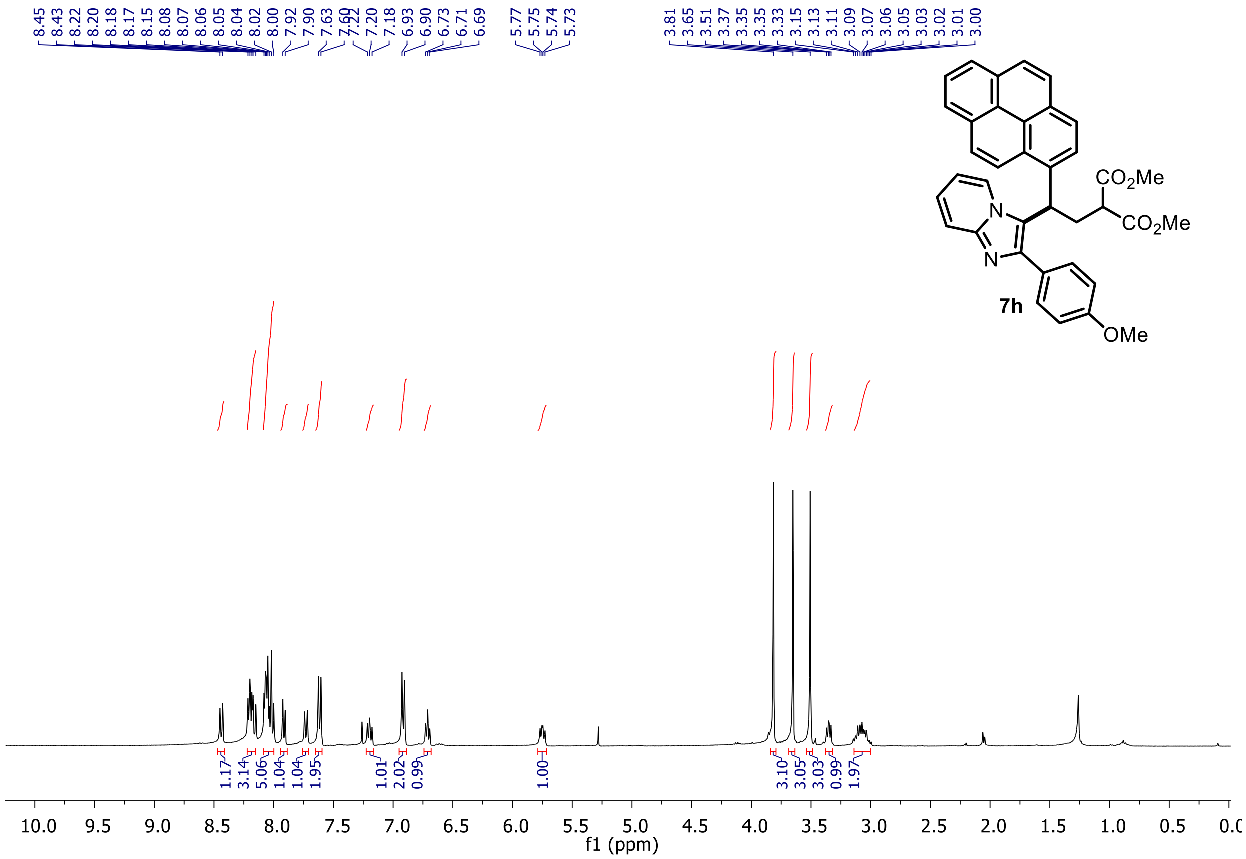

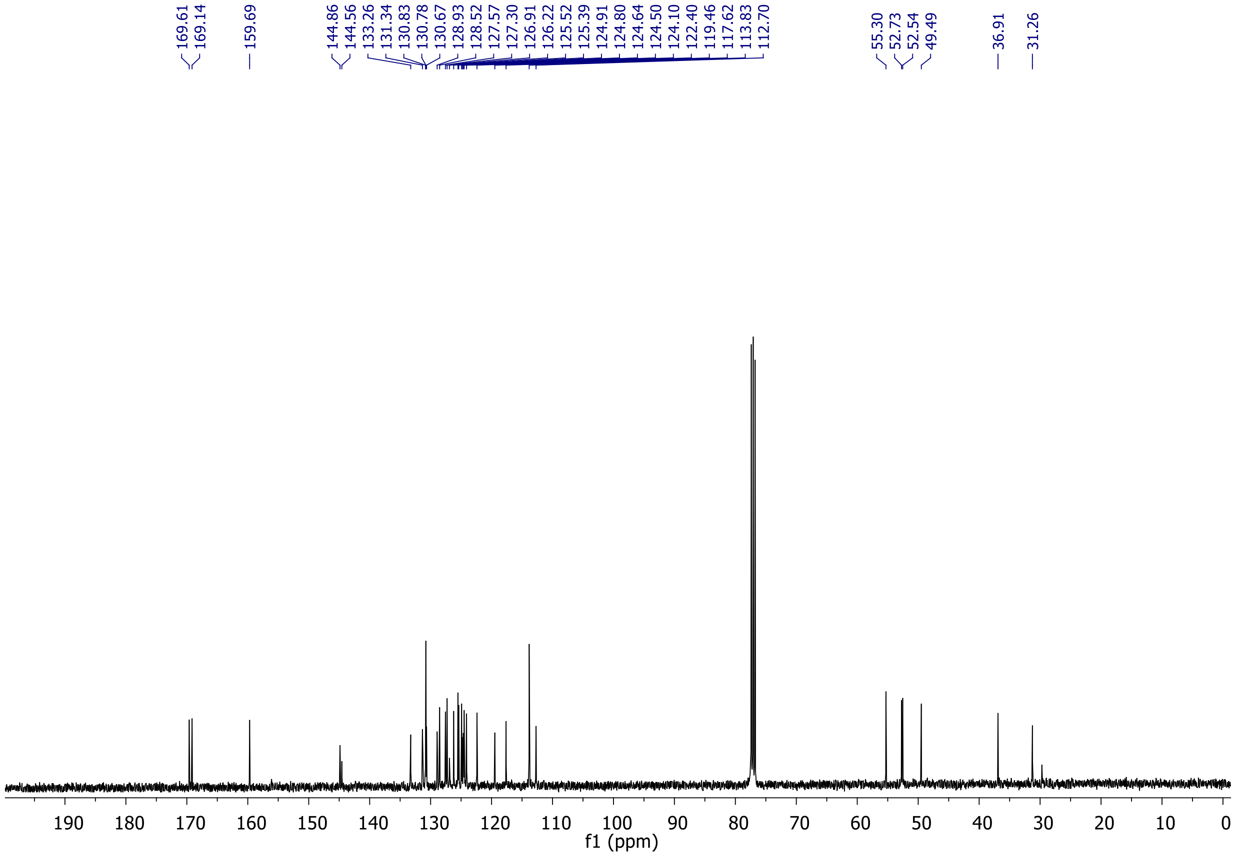

^{1}H NMR (400 MHz) and ^{13}C{^{1}H} NMR (100 MHz) spectra of **7h** ($CDCl_3$).

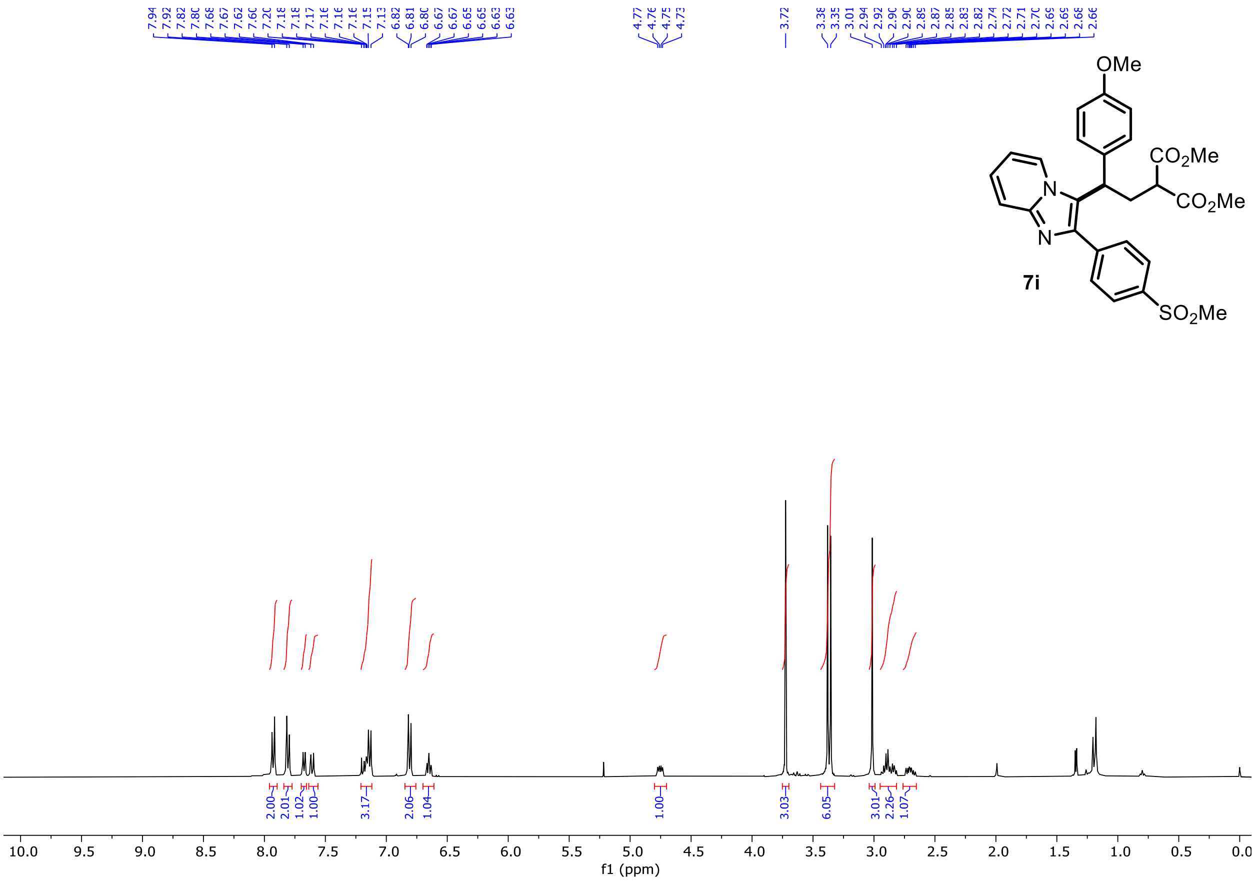

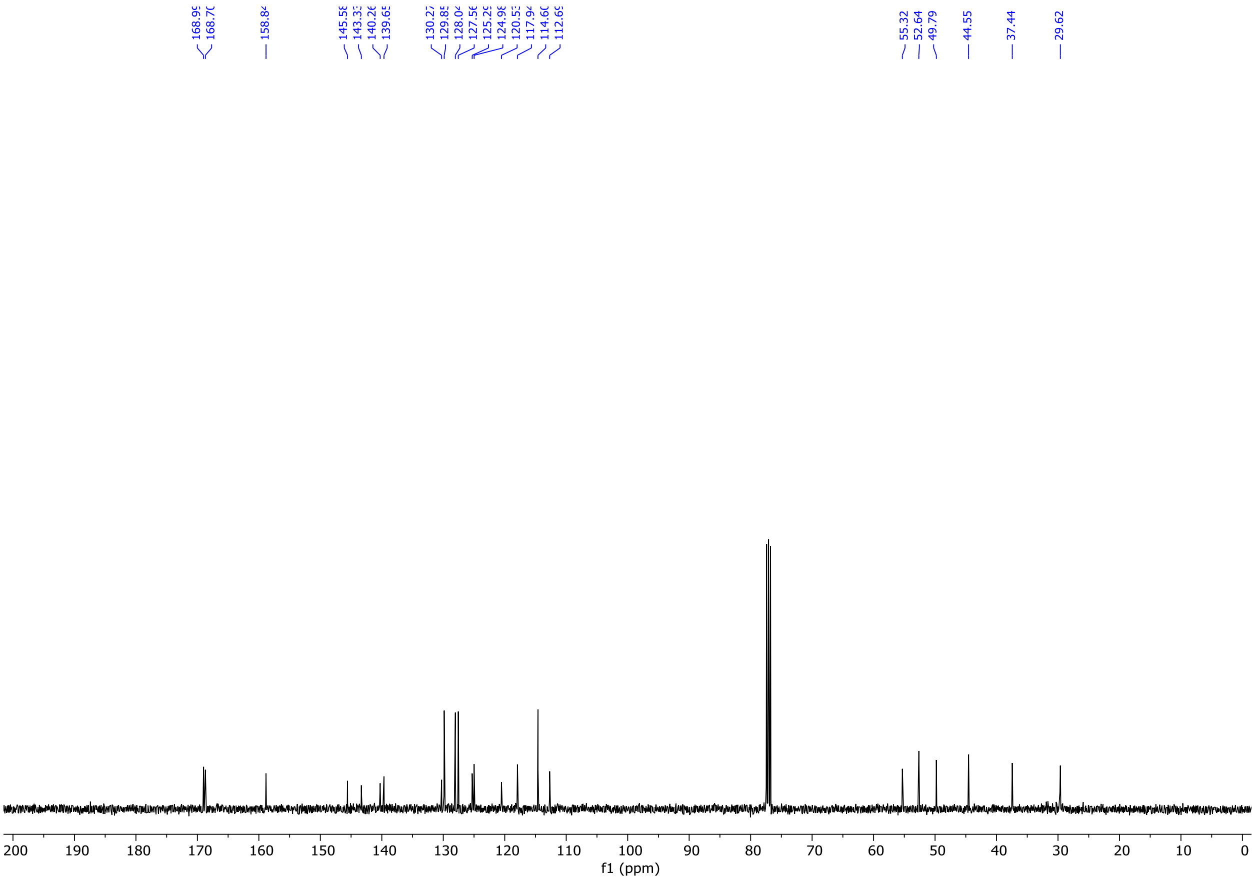

^{1}H NMR (400 MHz) and $^{13}C\{^{1}H\}$ NMR (100 MHz) spectra of **7i** ($CDCl_3$).

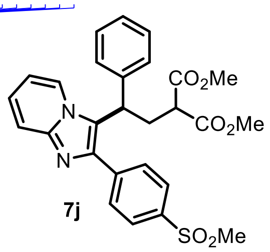

7j

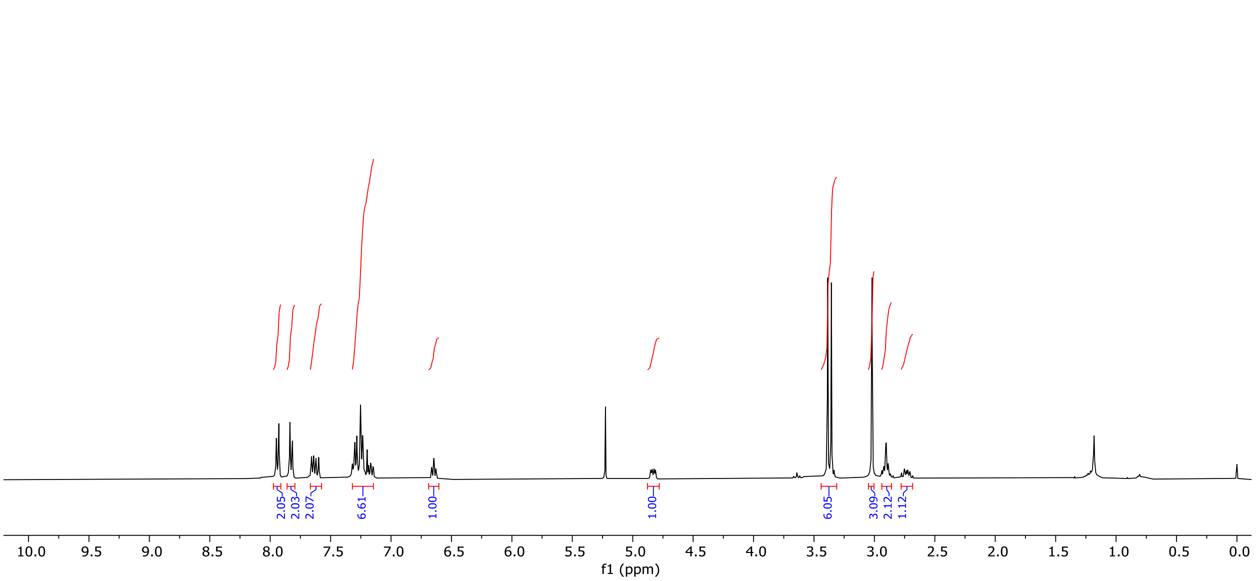

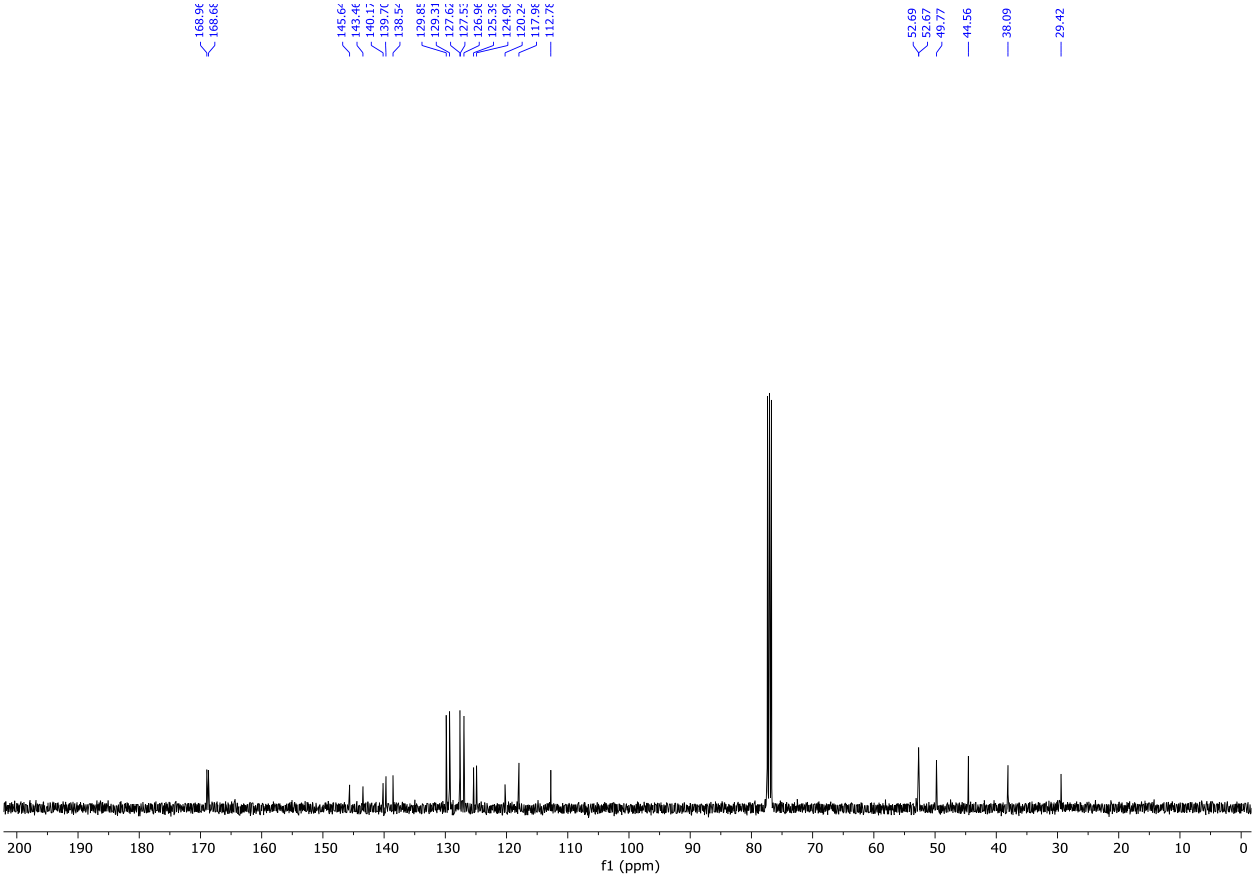

^{1}H NMR (400 MHz) and ^{13}C{^{1}H} NMR (100 MHz) spectra of **7j** ($CDCl_3$).

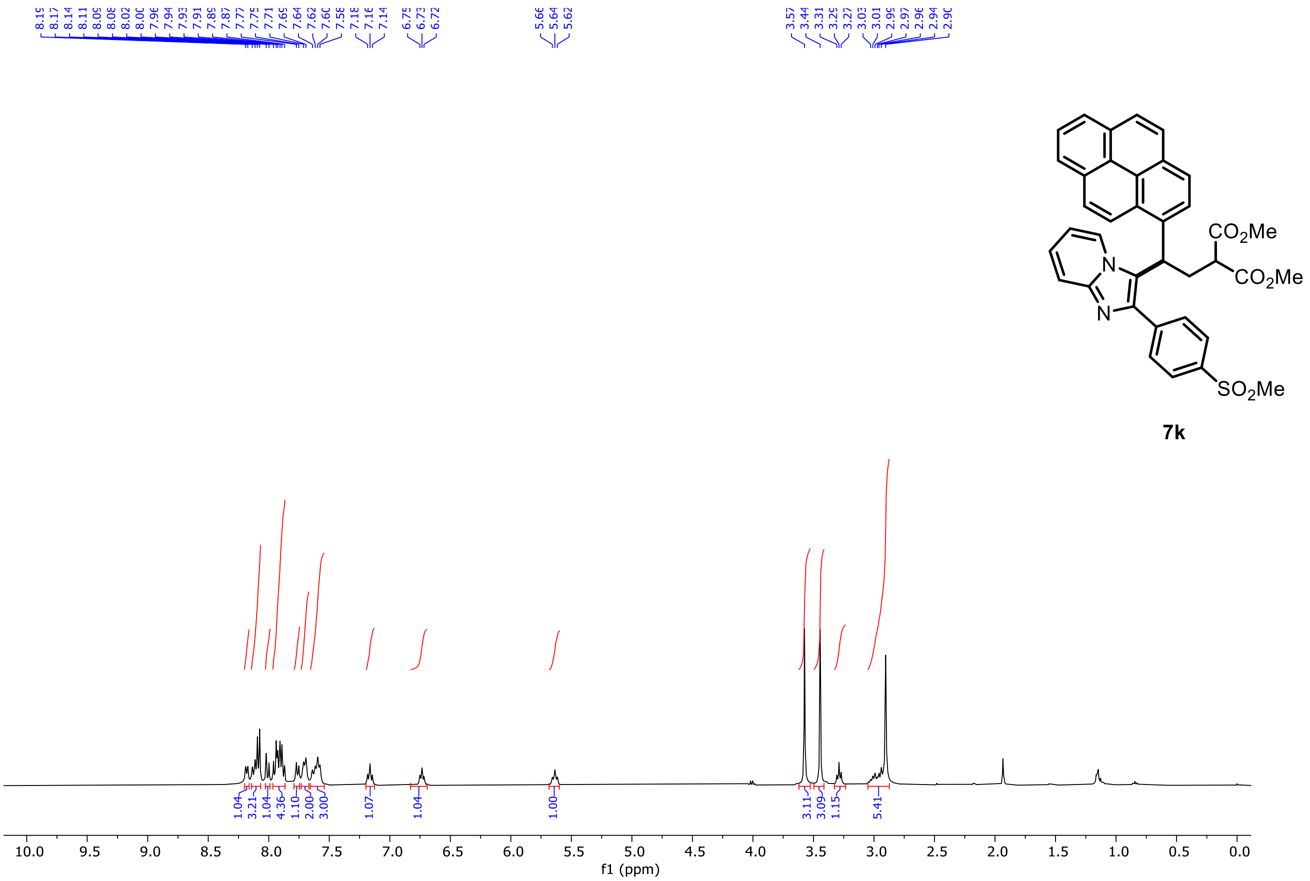

7k

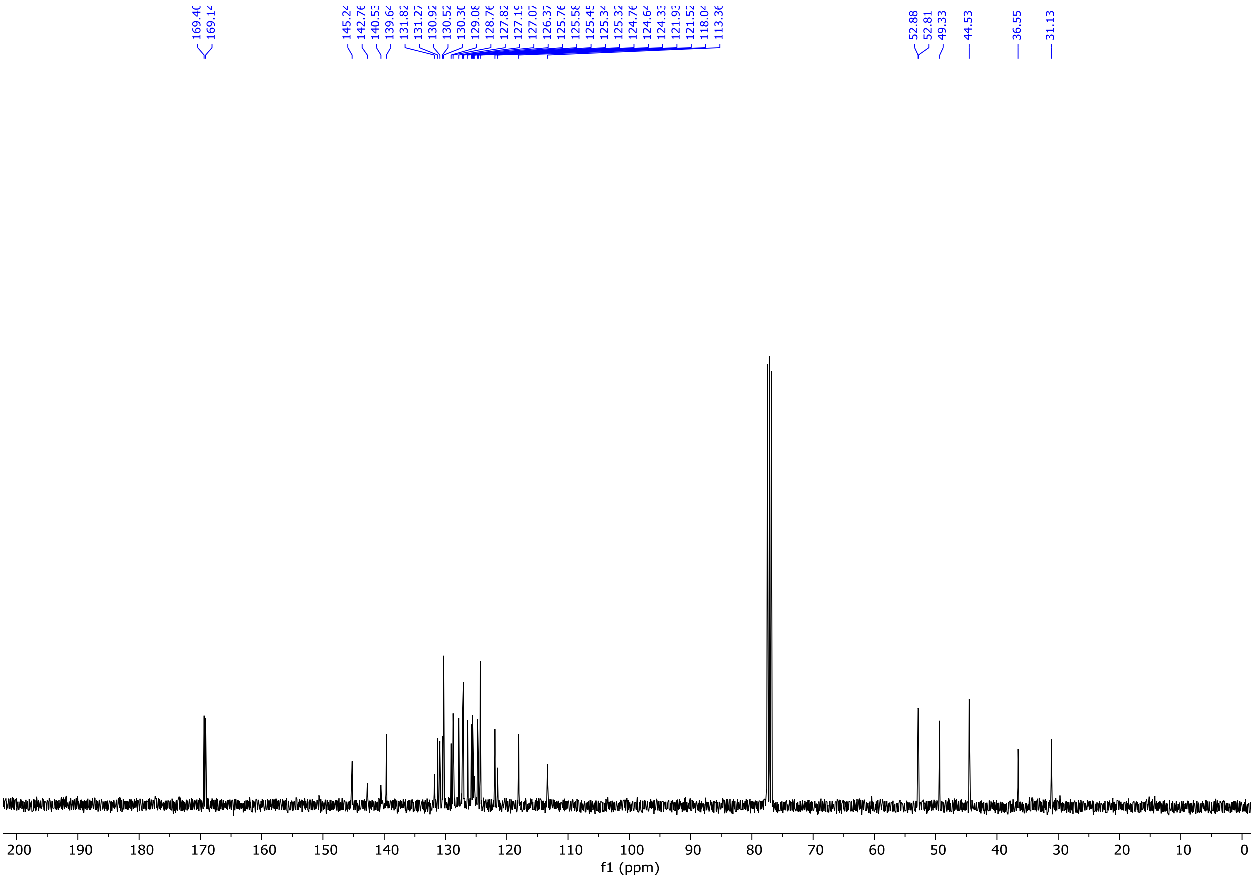

^{1}H NMR (400 MHz) and ^{13}C{^{1}H} NMR (100 MHz) spectra of **7k** ($CDCl_3$).

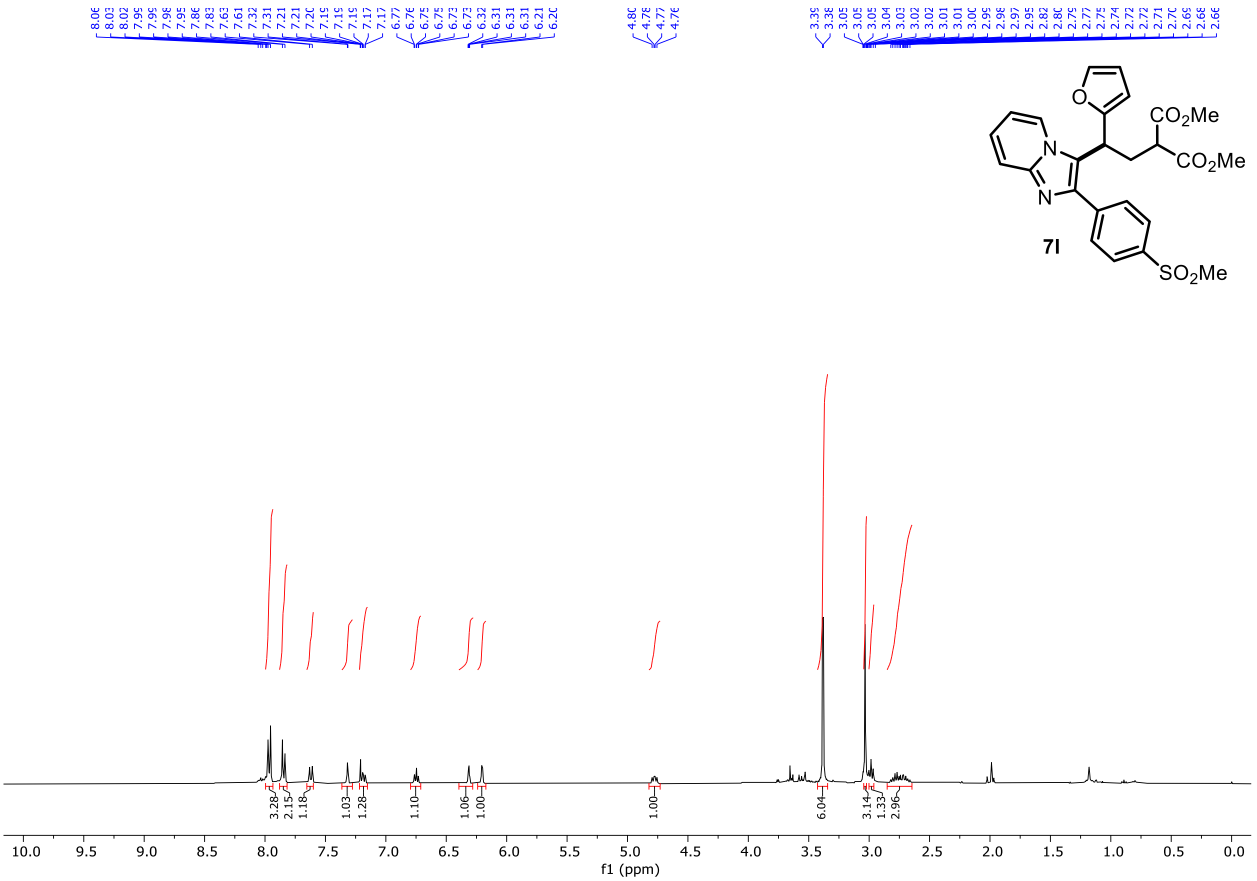

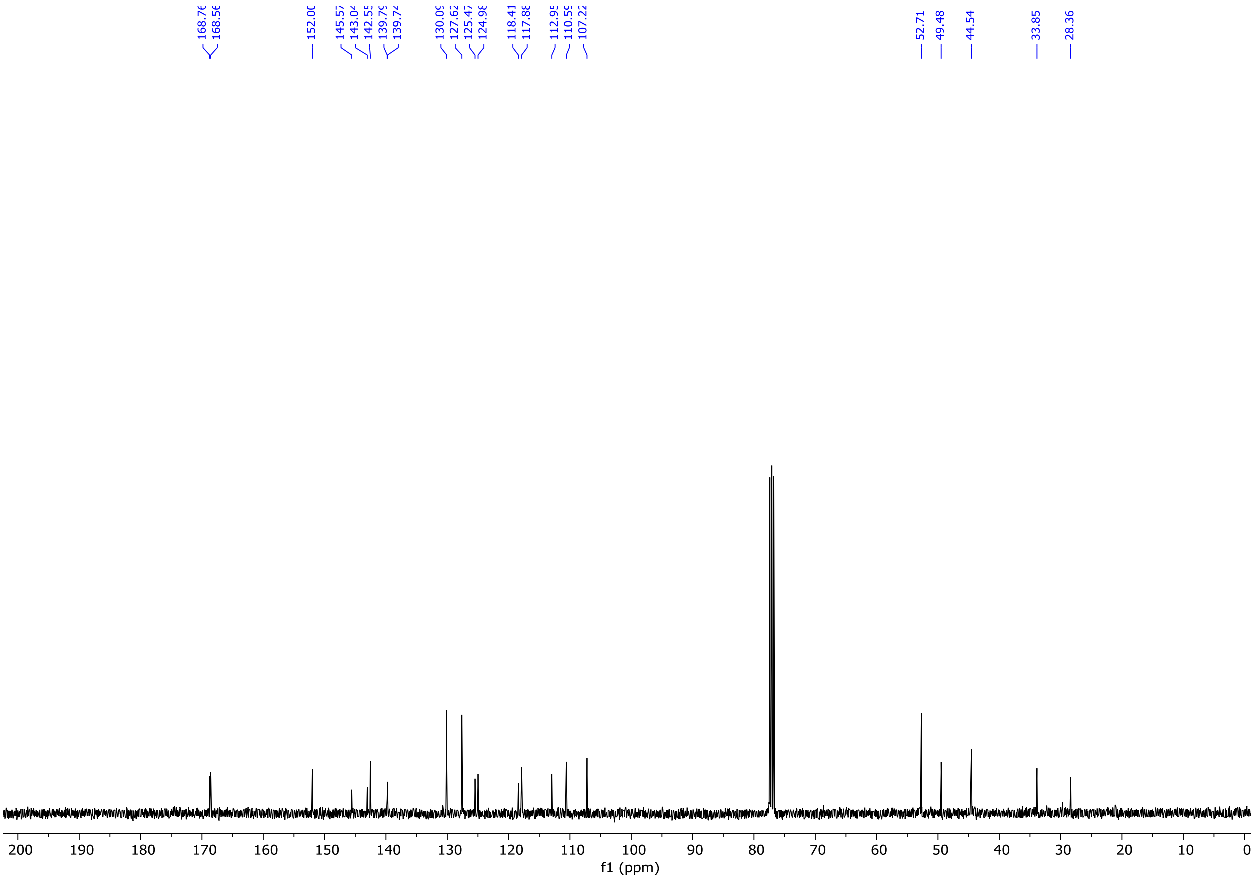

^{1}H NMR (400 MHz) and $^{13}C\{^{1}H\}$ NMR (100 MHz) spectra of **7l** ($CDCl_3$).

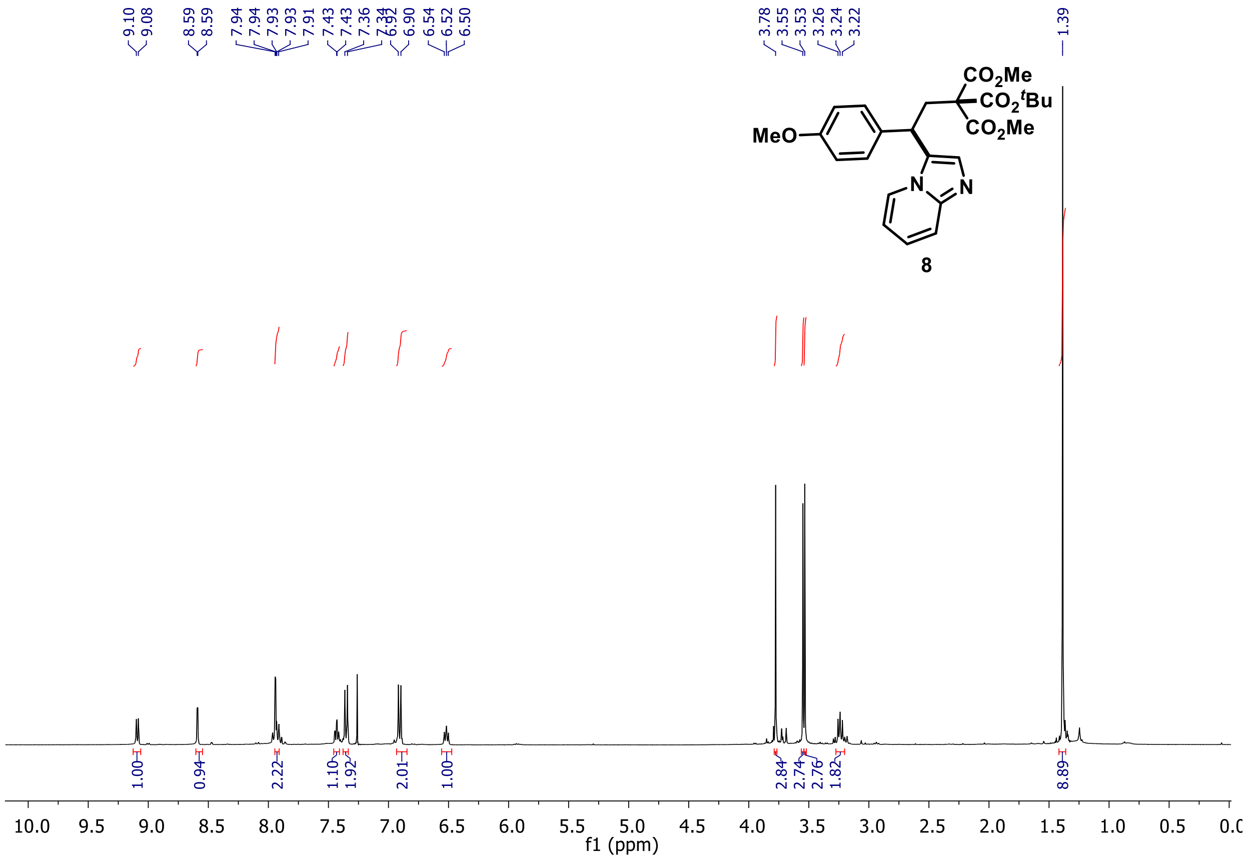

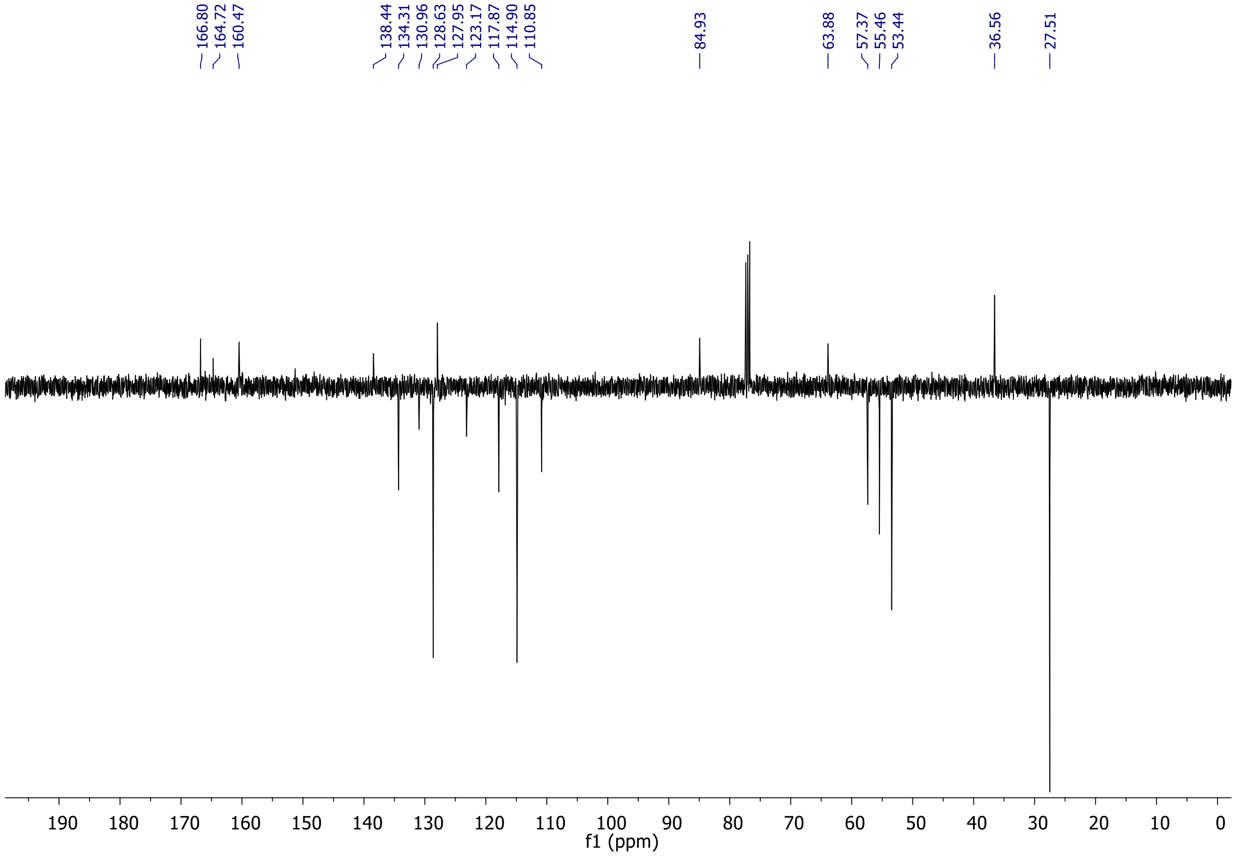

^{1}H NMR (400 MHz) and ^{13}C{^{1}H} APT NMR (100 MHz) spectra of **8** ($CDCl_3$).

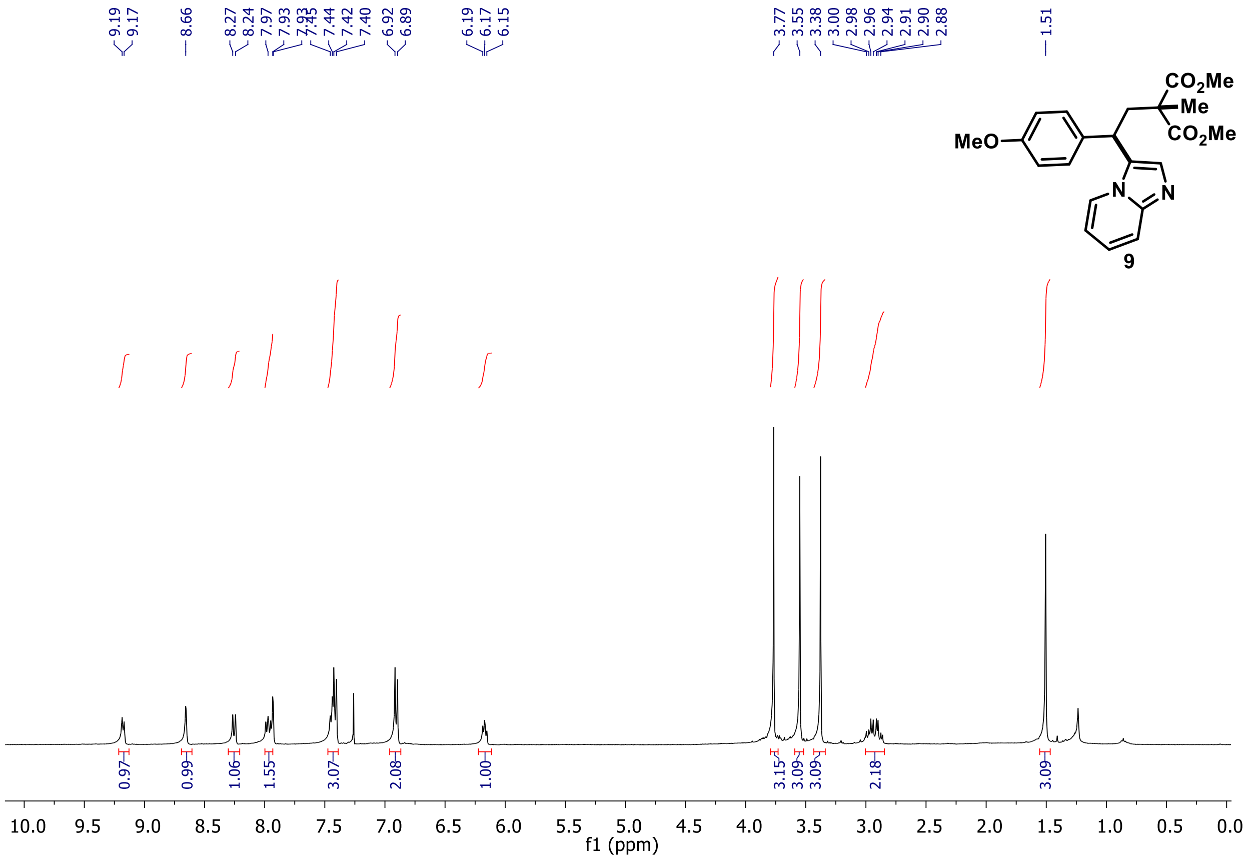

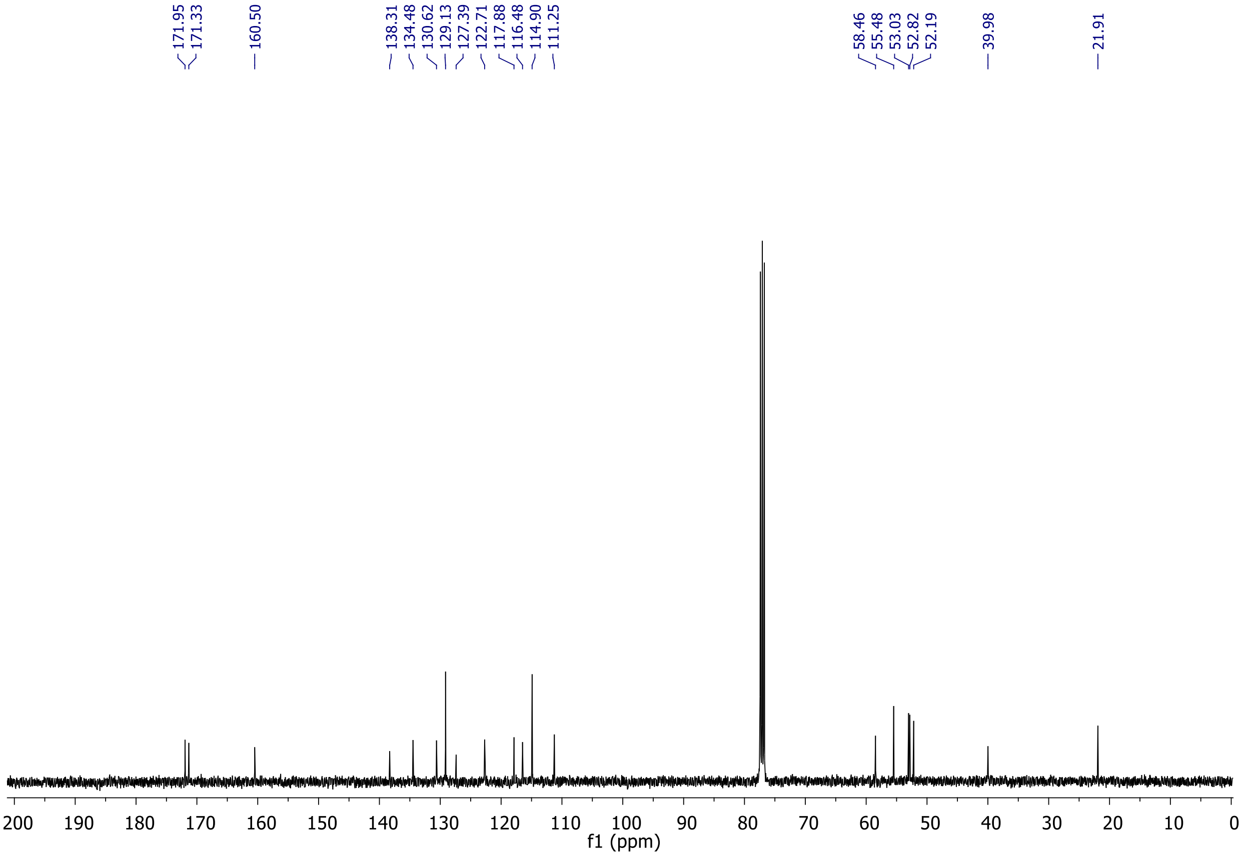

^{1}H NMR (400 MHz) and ^{13}C{^{1}H} NMR (100 MHz) spectra of **9** ($CDCl_3$).

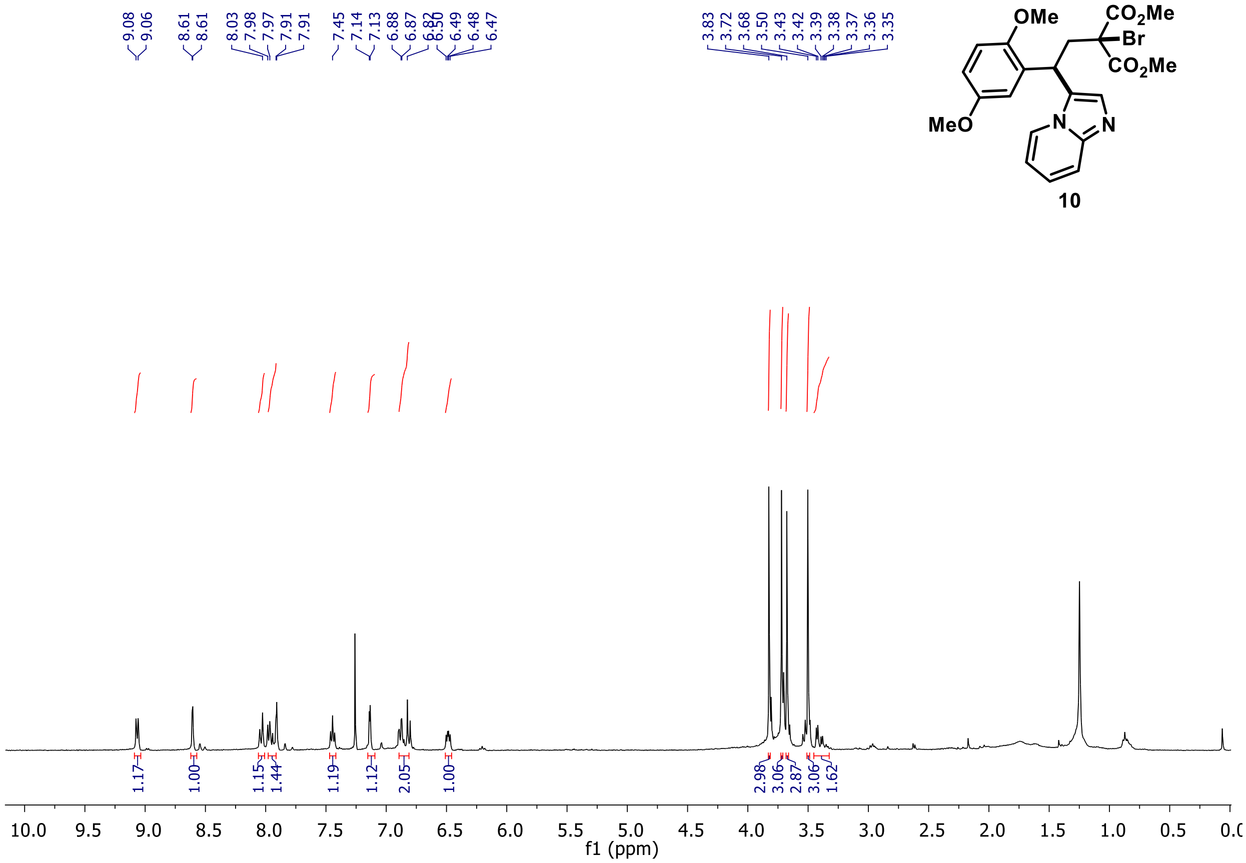

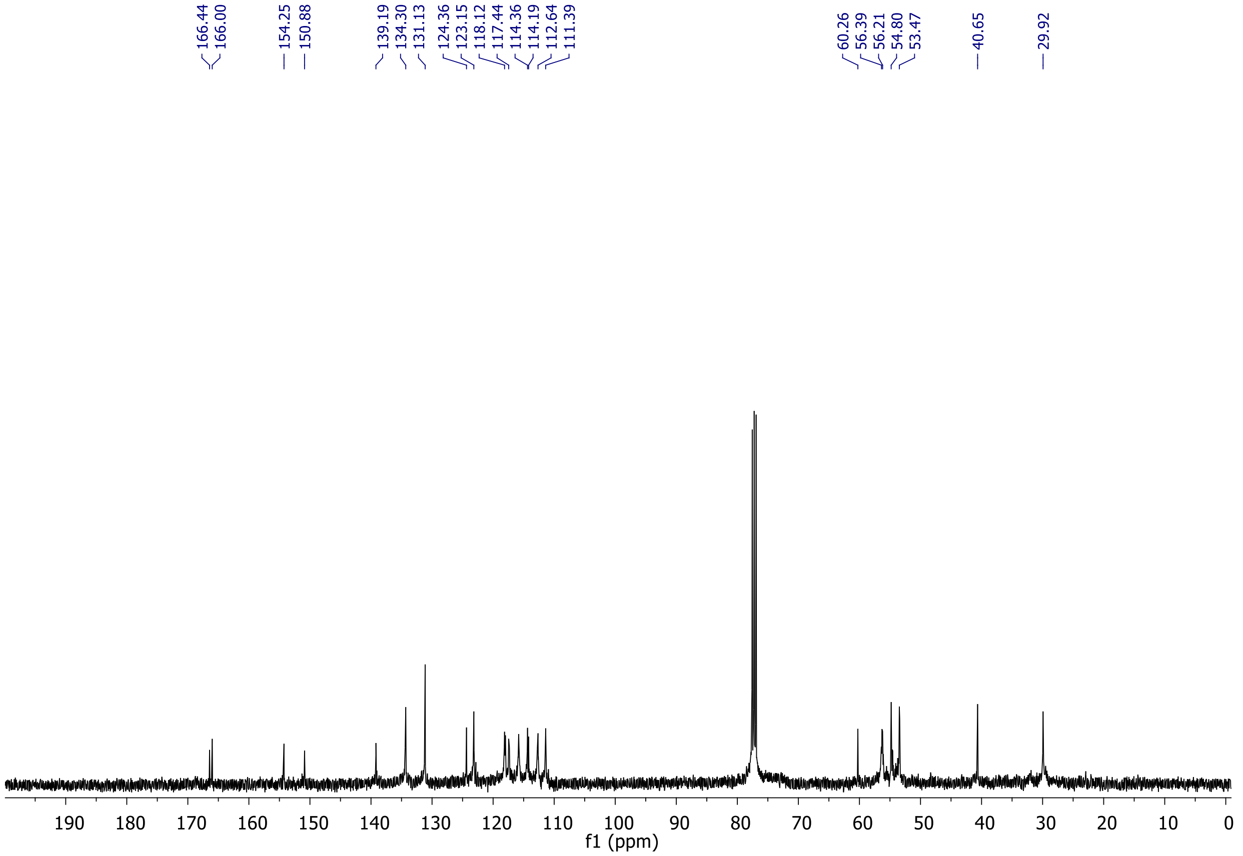

^{1}H NMR (400 MHz) and $^{13}C\{^{1}H\}$ NMR (100 MHz) spectra of **10** ($CDCl_3$).

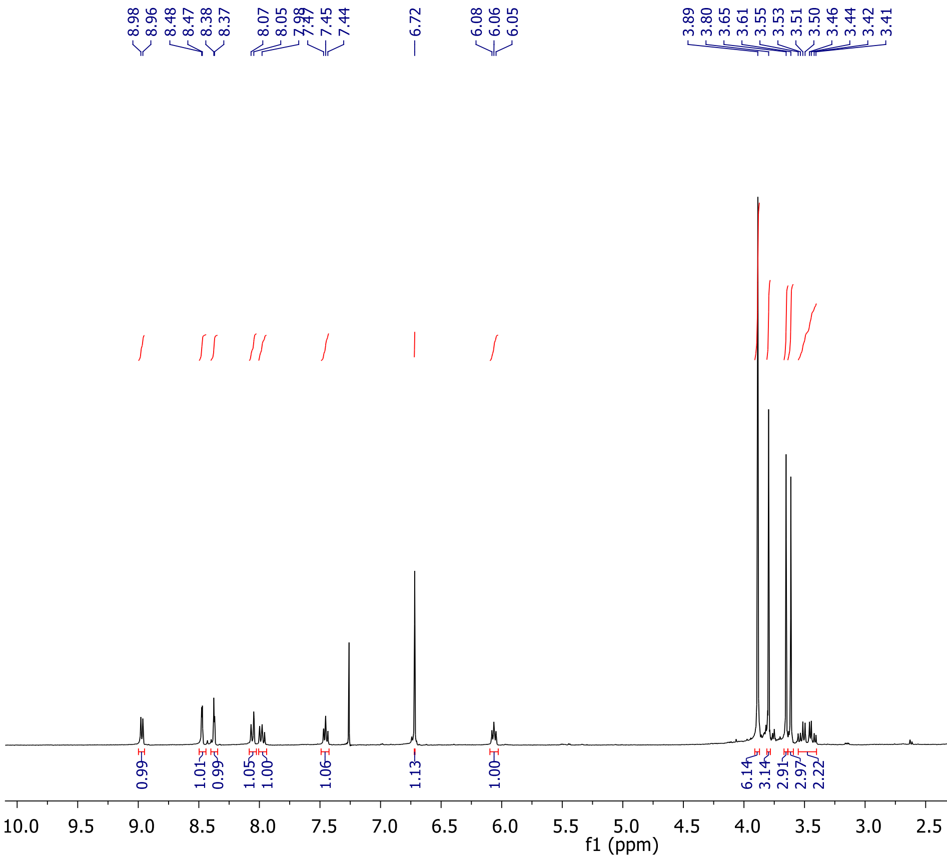

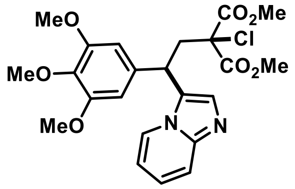

11

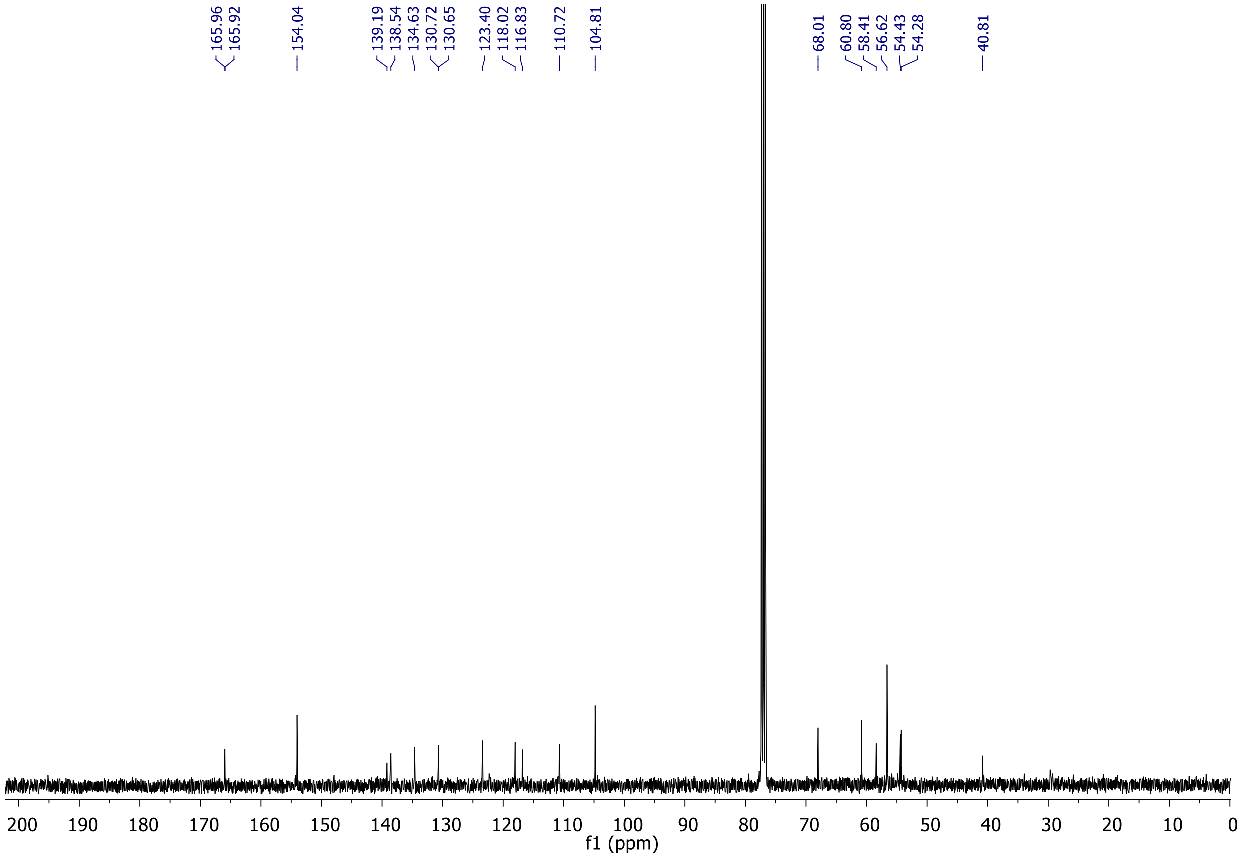

^{1}H NMR (400 MHz) and ^{13}C{^{1}H} NMR (100 MHz) spectra of **11** ($CDCl_3$).

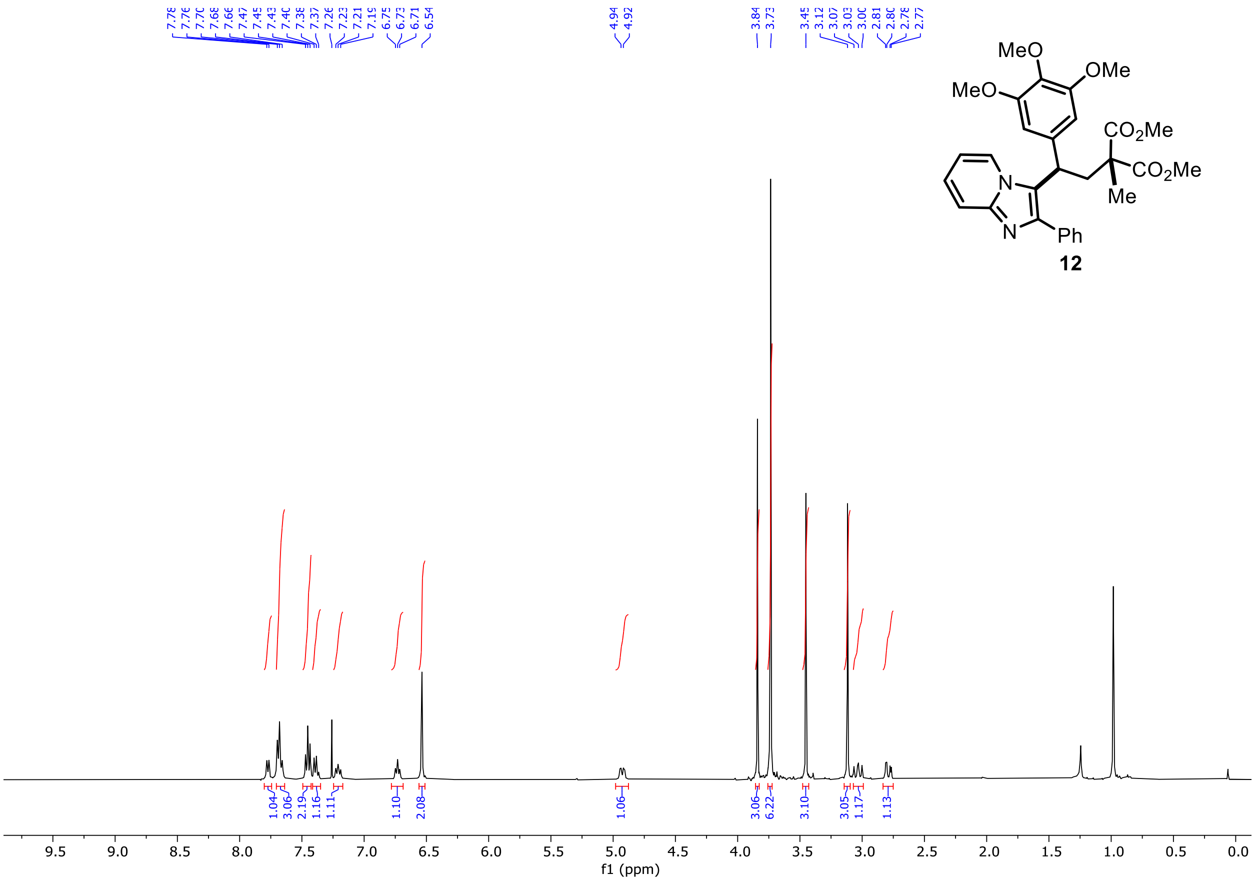

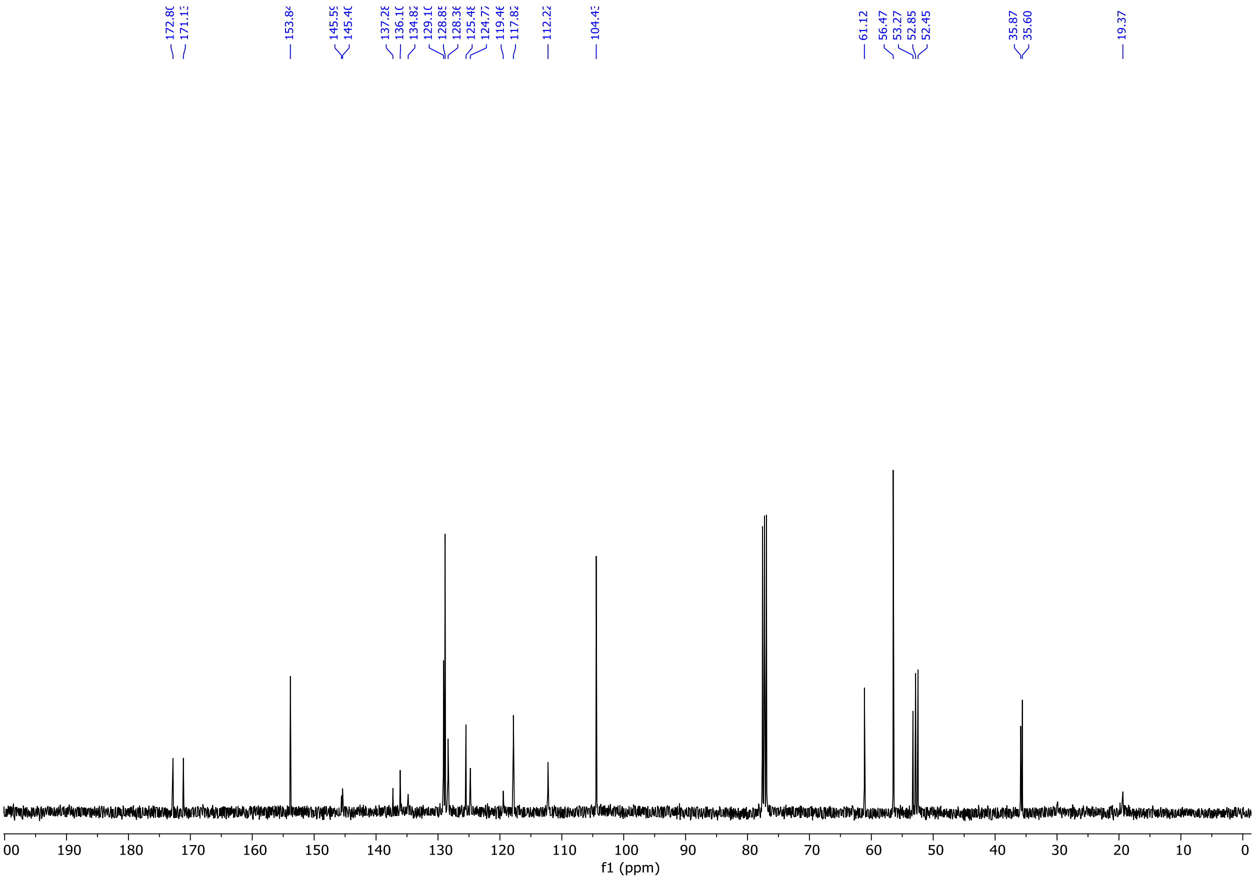

^{1}H NMR (400 MHz) and ^{13}C{^{1}H} NMR (100 MHz) spectra of **12** ($CDCl_3$).
